# Global impact of 10- and 13-valent pneumococcal conjugate vaccines on pneumococcal meningitis in all ages: The PSERENADE project

**DOI:** 10.1016/j.jinf.2025.106426

**Published:** 2025-03

**Authors:** Yangyupei Yang, Maria Deloria Knoll, Carly Herbert, Julia C. Bennett, Daniel R. Feikin, Maria Garcia Quesada, Marissa K. Hetrich, Scott L. Zeger, Eunice W. Kagucia, Melody Xiao, Adam L. Cohen, Mark van der Linden, Mignon du Plessis, Inci Yildirim, Brita A. Winje, Emmanuelle Varon, Maria Teresa Valenzuela, Palle Valentiner-Branth, Anneke Steens, J. Anthony Scott, Larisa Savrasova, Juan Carlos Sanz, Aalisha Sahu Khan, Kazunori Oishi, Néhémie Nzoyikorera, J. Pekka Nuorti, Jolita Mereckiene, Kimberley McMahon, Allison McGeer, Grant A. Mackenzie, Laura MacDonald, Shamez N. Ladhani, Karl G. Kristinsson, Jackie Kleynhans, James D. Kellner, Sanjay Jayasinghe, Pak-Leung Ho, Markus Hilty, Laura L. Hammitt, Marcela Guevara, Charlotte Gilkison, Ryan Gierke, Stefanie Desmet, Philippe De Wals, Ron Dagan, Edoardo Colzani, Pilar Ciruela, Urtnasan Chuluunbat, Guanhao Chan, Romina Camilli, Michael G. Bruce, Maria-Cristina C. Brandileone, Krow Ampofo, Katherine L. O’Brien, Kyla Hayford, The PSERENADE Team

**Affiliations:** a Johns Hopkins Bloomberg School of Public Health, Baltimore, MD 21205, United States; b UMass Chan Medical School, Worcester, MA 01655, United States; c Independent Consultant, 1296 Coppet, Switzerland; d Epidemiology and Demography Department, KEMRI-Wellcome Trust Research Programme, Centre for Geographic Medicine-Coast, P.O. Box 230-80108, Kilifi, Kenya; e World Health Organization, 1202 Geneva, Switzerland; f Reference Laboratory for Streptococci, Department of Medical Microbiology, University Hospital RWTH Aachen, 52074 Aachen, Germany; g Centre for Respiratory Diseases and Meningitis, National Institute for Communicable Diseases of the National Health Laboratory Service, Sandringham, 2192 Johannesburg, South Africa; h School of Pathology, Faculty of Health Sciences, University of the Witwatersrand, Braamfontein, 2000 Johannesburg, South Africa; i Department of Pediatrics, Yale New Haven Children’s Hospital, New Haven, CT 06504, United States; j Faculty of Health Sciences, Oslo Metropolitan University, 0130 Oslo, Norway; k National Reference Centre for Pneumococci, Centre Hospitalier Intercommunal de Créteil, 94000 Créteil, France; l Department of Public Health and Epidemiology, Faculty of Medicine, Universidad de los Andes, Metropolitan Region, Las Condes, Santiago, Chile; m Infectious Disease Epidemiology and Prevention, Statens Serum Institut, DK-2300 Copenhagen, Denmark; n Centre for Infectious Disease Control, National Institute for Public Health and the Environment, 3721 MA Bilthoven, the Netherlands; o Centre for Disease Prevention and Control of Latvia, Riga 1005, Latvia; p Doctoral Studies Department, Riga Stradiņš University, Riga 1007, Latvia; q Laboratorio Regional de Salud Pública, Dirección General de Salud Pública, Comunidad de Madrid, 28055 Madrid, Spain; r Ministry of Health and Medical Services, Suva, Fiji; s Toyama Institute of Health, Imizu, 939-0363 Toyama, Japan; t Higher Institute of Bioscience and Biotechnology, Mohammed VI University of Sciences and Health (UM6SS), Casablanca, Morocco; u Laboratory of Infectiology and Microbial Biotechnology Research, Mohammed VI Center for Research & Innovation (CM6), Rabat, Morocco; v National Reference Laboratory, Institut National de Santé Publique (INSP) du Burundi, Bujumbura, Burundi; w Department of Health Security, Finnish Institute for Health and Welfare, 00271 Helsinki, Finland; x Health Sciences Unit, Faculty of Social Sciences, Tampere University, 33100 Tampere, Finland; y Health Protection Surveillance Centre (HPSC), 25-27 Gardiner Street Middle, Dublin D01 A4A3, Ireland; z Centre for Disease Control, Department of Health and Community Services, Darwin City, NT 8000, Australia; aa Toronto Invasive Bacterial Diseases Network, and Department of Laboratory Medicine and Pathobiology, University of Toronto, Toronto, ON M5S 1A8, Canada; ab Department of Paediatrics, University of Melbourne, Parkville, Melbourne, Victoria, Australia; ac Faculty of Infectious and Tropical Diseases, London School of Hygiene & Tropical Medicine, Keppel St, London WC1E 7HT, United Kingdom; ad Medical Research Council Unit The Gambia at London School of Hygiene & Tropical Medicine, PO Box 273, Banjul, Gambia; ae New Vaccines Group, Murdoch Children’s Research Institute, Parkville, Melbourne, 3052 Victoria, Australia; af Public Health Scotland, Glasgow, United Kingdom; ag Immunisation and Countermeasures Division, UK Health Security Agency, NW9 5EQ London, United Kingdom; ah Department of Clinical Microbiology, Landspitali - The National University Hospital, Hringbraut, 101 Reykjavik, Iceland; ai School of Public Health, Faculty of Health Sciences, University of the Witwatersrand, Johannesburg, South Africa; aj Department of Pediatrics, University of Calgary, and Alberta Health Services, Calgary, Alberta T3B 6A8, Canada; ak National Centre for Immunisation Research and Surveillance and Discipline of Child and Adolescent Health, Children’s Hospital Westmead Clinical School, Faculty of Medicine and Health, University of Sydney, Westmead, 2145 NSW, Australia; al Department of Microbiology and Carol Yu Centre for Infection, Queen Mary Hospital, The University of Hong Kong, Hong Kong SAR; am Swiss National Reference Centre for invasive Pneumococci, Institute for Infectious Diseases, University of Bern, 3012 Bern, Switzerland; an CIBER Epidemiología y Salud Pública, (CIBERESP), 28029 Madrid, Spain; ao Instituto de Salud Pública de Navarra – IdiSNA, 31003 Pamplona, Navarra, Spain; ap Epidemiology Team, Institute of Environmental Science and Research, Porirua, 5022 Wellington, New Zealand; aq Division of Bacterial Diseases, National Center for Immunization and Respiratory Diseases, Centers for Disease Control and Prevention, Atlanta, GA 30329, United States; ar Department of Microbiology, Immunology and Transplantation, KU Leuven, BE-3000 Leuven, Belgium; as National Reference Centre for Streptococcus pneumoniae, University Hospitals Leuven, 3000 Leuven, Belgium; at Department of Social and Preventive Medicine, Laval University, Québec, Quebec G1V 0A6, Canada; au The Shraga Segal Dept. of Microbiology, Immunology and Genetics Faculty of Health Sciences of the Ben-Gurion University of the Negev, Beer-Sheva, Israel; av European Centre for Disease Prevention and Control, 169 73 Solna, Sweden; aw Surveillance and Public Health Emergency Response, Public Health Agency of Catalonia, 08005 Barcelona, Spain; ax National Center of Communicable Diseases (NCCD), Ministry of Health, Bayanzurkh District, 13336 Ulaanbaatar, Mongolia; ay Singapore Ministry of Health, Communicable Diseases Division, 308442, Singapore; az Department of Infectious Diseases, Italian National Institute of Health (Istituto Superiore di Sanità, ISS), 00161 Rome, Italy; ba Arctic Investigations Program, Division of Infectious Disease Readiness and Innovation, National Center for Emerging and Zoonotic Infectious Diseases, Centers for Disease Control and Prevention, Anchorage, AK 99508, United States; bb National Laboratory for Meningitis and Pneumococcal Infections, Center of Bacteriology, Institute Adolfo Lutz (IAL), São Paulo, 01246-902, Brazil; bc Division of Pediatric Infectious Diseases, Department of Pediatrics, University of Utah Health Sciences Center, Salt Lake City, UT 84132, United States

**Keywords:** Pneumococcal meningitis, Pneumococcal conjugate vaccines, Serotypes, Vaccine impact, Incidence, Serotype replacement, Indirect protection

## Abstract

**Background:**

Pneumococcal conjugate vaccines (PCVs) introduced in childhood national immunization programs lowered vaccine-type invasive pneumococcal disease (IPD), but replacement with non-vaccine-types persisted throughout the PCV10/13 follow-up period. We assessed PCV10/13 impact on pneumococcal meningitis incidence globally.

**Methods:**

The number of cases with serotyped pneumococci detected in cerebrospinal fluid and population denominators were obtained from surveillance sites globally. Site-specific meningitis incidence rate ratios (IRRs) comparing pre-PCV incidence to each year post-PCV10/13 were estimated by age (<5, 5–17 and ≥18 years) using Bayesian multi-level mixed effects Poisson regression, accounting for pre-PCV trends. All-site weighted average IRRs were estimated using linear mixed-effects regression stratified by age, product (PCV10 or PCV13) and prior PCV7 impact (none, moderate, or substantial). Changes in pneumococcal meningitis incidence were estimated overall and for product-specific vaccine-types and non-PCV13-types.

**Results:**

Analyses included 10,168 cases <5 y from PCV13 sites and 2849 from PCV10 sites, 3711 and 1549 for 5–17 y and 29,187 and 5653 for ≥18 y from 42 surveillance sites (30 PCV13, 12 PCV10, 2 PCV10/13) in 30 countries, primarily high-income (84%). Six years after PCV10/PCV13 introduction, pneumococcal meningitis declined 48–74% across products and PCV7 impact strata for children <5 y, 35–62% for 5–17 y and 0–36% for ≥18 y. Impact against PCV10-types at PCV10 sites, and PCV13-types at PCV13 sites was high for all age groups (<5 y: 96–100%; 5–17 y: 77–85%; ≥18 y: 73–85%). After switching from PCV7 to PCV10/13, increases in non-PCV13-types were generally low to none for all age groups.

**Conclusion:**

Pneumococcal meningitis declined in all age groups following PCV10/PCV13 introduction. Plateaus in non-PCV13-type meningitis suggest less replacement than for all IPD. Data from meningitis belt and high-burden settings were limited.

## Highlights

• PCV10 & PCV13 reduced pneumococcal meningitis 48-74% globally in children under 5.
• Indirect protection from PCV10 & PCV13 reduced pneumococcal meningitis in all ages.
• Replacement disease due to non-vaccine-type serotypes was minimal.
• Settings with serotype 19A meningitis likely benefit from PCVs targeting 19A.
• Increases in non-PCV13-type meningitis plateaued unlike for all invasive disease.

**Research in Context**

**Evidence before this study**

Widespread use of 10- and 13-valent pneumococcal conjugate vaccines (PCV10/13) in infant immunization programs has reduced vaccine-type invasive pneumococcal disease (IPD) across various age groups. The pathogenesis of meningitis is frequently different than other IPDs since in meningitis, bacteria can directly ascend from the nose to the meninges, especially in adults, and thus, serotype-specific invasiveness potential may also differ between syndromes. Consequently, the post-vaccination dynamics may also differ. We previously published estimates of the impact of PCV10/13 on vaccine-type, non-vaccine-type and all IPD as part of the PSERENADE project, a multi-site collaboration from 62 countries from all regions and income levels. We also previously demonstrated the impact of PCV10/13 on serotype 1 meningitis, which showed its near elimination in all ages by approximately six years after introduction into infant programs. Additionally, we showed that remaining pneumococcal meningitis in mature immunization programs (i.e., >5 years of PCV10/13 use with high uptake) are largely caused by non-vaccine types. A PubMed search for reviews evaluating long-term effects of PCV10/13 on pneumococcal meningitis incidence using the search terms “PCV”, “pneumococcal meningitis”, “impact OR effect*” and “NOT cost” resulted in only two publications that reviewed impact in multiple countries. Both studies evaluated predominantly short-term data in only five countries and observed heterogeneous effects across sites. Therefore, the impact of PCV10/13 on all pneumococcal meningitis incidence beyond serotype 1 remained insufficiently explored, including the impact of replacement with non-vaccine serotypes.

**Added value of this study**

This is, to our knowledge, the only globally representative study that estimates the full direct and indirect impact of PCV10/13 achieved after long-term impact of pediatric PCV10/13 programs on all pneumococcal meningitis in all ages. Utilizing data from 42 sites from 30 countries with global coverage, we observed an 83–99% decline in vaccine-type pneumococcal meningitis incidence relative to pre-PCV incidence across all age groups. Net declines in all pneumococcal meningitis incidence ranged between 48–74% for PCV10 and PCV13 groups among children aged <5 years, 35–62% among children 5–17 years and 0–36% among adults 18 years and older. Non-PCV13-type meningitis incidence increased approximately 2-fold six years after introduction relative to pre-PCV in children <5 years, but remained similar to pre-PCV incidence among older children and adults in most vaccine group strata. In contrast, serotype 19A increased more than 3-fold at PCV10 sites among both children <5 years and adults. No consistent patterns were seen for serotype 3 for either product. Analyses restricted to sites providing both meningitis and IPD data showed no consistent differences in impact between all pneumococcal meningitis and all IPD but data were sparse for PCV10 sites; for non-PCV13-serotype disease, IPD (all clinical pictures) generally increased more than meningitis.

**Implications of all the available evidence**

Overall, infant PCV programs reduced pneumococcal meningitis across all age groups, with no clear differences between PCV10 and PCV13 in net all-pneumococcal meningitis reductions. PCV10 and PCV13 impact differed primarily regarding serotype 19A meningitis, which increased among children <5 years and adults at PCV10 sites and was virtually eliminated at PCV13 sites in all ages. This indicates that sites with 19A meningitis could benefit from PCVs targeting serotype 19A. When comparing meningitis to all IPD, no consistent differences in net declines of all serotypes were observed between syndromes despite less replacement with non-PCV13-type disease for meningitis, suggesting they may have had differences in their serotype distributions pre-PCV. Higher valent PCVs may further impact pneumococcal meningitis. The limited availability of serotype-specific impact data from the highest meningitis burden countries, including the African meningitis belt, limits understanding of current pneumococcal meningitis disease burden in these countries, and poses challenges for policy-making for higher valent PCVs in those settings.

## Introduction

*Streptococcus pneumoniae* is a leading cause of bacterial meningitis, causing an estimated 83,900 cases and 37,900 deaths globally in 2015 in children <5 years of age with a 44% case-fatality rate.^1^ Pneumococcal meningitis is of especially high concern across the African meningitis belt, which experiences high endemic rates of meningitis and large seasonal outbreaks.^2^ In 2007, the World Health Organization (WHO) recommended inclusion of pneumococcal conjugate vaccines (PCVs) in infant immunization programs^3^ and PCVs have been introduced in over 166 countries worldwide.^4^ Beginning in 2009 and 2010, PCV immunization programs used 10-valent (PCV10; GlaxoSmithKline (GSK), Synflorix) or 13-valent PCV (PCV13; Pfizer, Prevnar13/Prevenar13).

The Pneumococcal Serotype Replacement and Distribution Estimation (PSERENADE) project was commissioned by WHO in 2018 to evaluate impact of PCV10/13 on invasive pneumococcal disease (IPD) incidence and serotype distribution. That analysis estimated that PCV10/13 reduced all serotype IPD by 58–74% in children <5 years of age and also reduced all IPD in older children and adults but to a lesser degree (62–65%, 4–62% for 5–17 years and above 18 years, respectively) through indirect protection.^5^ Large reductions in vaccine-type (VT) IPD were partially offset by replacement disease from non-vaccine-type (NVT) serotypes in all age groups.

However, PCV impact may differ between IPD causing meningitis and non-meningitis IPD (e.g., pneumonia and sepsis), either because of differences between serotypes’ ability to invade these tissues, host factors leading to different serotype-specific susceptibility or because vaccine effectiveness may differ between syndromes.^6^ Understanding the impact of PCV on pneumococcal meningitis is also critical to assess the progress of WHO’s road map to defeat meningitis by 2030, which aims to substantially reduce the number of meningitis cases globally.^7^ Here, we aimed to estimate the impact of long-term use of PCV10/13 in infant immunization programs on the incidence of pneumococcal meningitis in all ages.

## Methods

### Data collection and eligibility criteria

The systematic approach to identify sites eligible for the PSERENADE project was described in detail previously.^8^ Sites eligible for inclusion in analyses used PCV10 or PCV13 for at least one year, provided annual population denominators by age, had at least 50% uptake for the primary series at 12 months of age for at least one year post-PCV10/13 introduction, and observed serotype-specific meningitis cases for at least two years in at least one age group, excluding the year of introduction. Definitions of clinical meningitis differed across sites and some sites did not have syndromic characterization of cases (Table S1), so cases eligible for the primary analysis were confirmed positive for pneumococci in cerebrospinal fluid (CSF+) using *lytA*-based polymerase chain reaction (PCR), culture, or antigen testing. However, a sensitivity analysis was conducted additionally including clinically defined meningitis cases (i.e., blood-culture positive with CSF being negative or not tested), which added cases but not additional sites. Cases up to ten years before any PCV introduction and all years post-PCV introduction through 2018 were eligible. Sites with only post-PCV data contributed to analyses for the time period with data.

Two PSERENADE coordinators conducted a standardized data quality review for each site to evaluate changes in surveillance, pneumococcal identification methods, or other factors beyond PCV introduction that may have influenced incidence rates (IR) of all IPD or meningitis over time. After discussion with site investigators, potentially unstable site-year-age group data that might bias results were excluded.^8^ The PCV introduction year (‘year 0’) was defined as the year PCV7, PCV10, or PCV13 was universally introduced into national immunization programs (i.e., recommended for all age-eligible children); if PCV was introduced October to December, it was defined as the following calendar year. For sites where PCV was partially introduced (i.e., private market use prior to universal introduction), we considered the year of introduction to be the year used by the site for site-level analyses. For data submitted in epidemiologic years rather than calendar years, the introduction year was defined accordingly.

### Adjustments for missing data

Analyses of VT, NVT, and serotype-specific meningitis accounted for missing or unknown serotype data, as previously described in Supplemental Methods and published elsewhere.^5^ This includes cases that were “not serotyped” (serotyping was not attempted for any reason), “untypeable”, “typed, serotype not identified”, serogrouped-only (e.g., 6A/6B/6C/6D), undistinguished (e.g., 6A/6C), “serotype pool” or two serotypes reported. The serotype distribution of cases with missing or unknown serotype was assumed to be similar to that of serotyped cases. Cases were excluded from VT, NVT, and serotype-specific analyses for site-year-age group strata where evidence was insufficient to support this assumption (e.g., preferential selection of serotyping based upon severity), or when less than 50% of meningitis cases were serotyped in that stratum; these may still have been included in analyses of all-meningitis. To account for not serotyped cases in vaccine-type, non-vaccine-type, and serotype-specific analyses, population denominators were adjusted by the proportion of cases serotyped, as opposed to reapportioning unknown serotypes, to weight sites in the model on the basis of the actual serotype data reported. “Untypeable” and “typed, serotype not identified” were considered NVTs if the serotyping method tested for all VTs. Serogrouped (4.2%) or undistinguished (1.4%) cases were distributed using the serotype distribution of IPD cases because site-year-age group strata had too few meningitis cases for robust distribution estimation.

### Statistical analyses

Annual meningitis incidence rate ratios (IRR) comparing the period prior to universal introduction of any PCV (pre-PCV period) to each year post-PCV10/13 introduction were estimated by age group (<5 years, 5–17 years, and ≥18 years) for all serotype (all-ST) meningitis, PCV7 serotypes, the additional non-PCV7-STs in PCV10 (ST1, ST5, and ST7F), ST3, ST6A, ST19A, non-PCV13-ST and all PCV10-type and all PCV13-type meningitis. Unless otherwise stated, IRRs 6 years after PCV10/13 introduction were reported to describe percent decreases and fold increases relative to pre-PCV.

IRRs were estimated in a three-step process described elsewhere^5^ (Supplemental Methods). In brief, first, meningitis incidence rates (IR) were estimated for each site using Bayesian multi-level, mixed-effects Poisson regression using the MCMCglmm package in R,^9^ separately for sites that used PCV10 vs PCV13. Second, IRRs were estimated comparing pre-PCV incidence to each post-PCV10/13 year (reported as the mean of the Bayesian model posterior distribution of rate ratios) for each site. Credibility intervals (CIs, Bayesian confidence interval analog) were estimated using the 2.5 and 97.5 percentiles of the posterior distribution of the rate ratios. Third, all-site weighted average IRRs were estimated using linear mixed-effects regression stratified by product (PCV10 or PCV13) and degree of impact of prior PCV7 use on all IPD (either ‘no impact’ if PCV7 never used, ‘moderate impact’ if PCV7-type IPD IRR>0.05 among children <5 years in the last year of PCV7 use, or ‘substantial impact’ if PCV7-type IPD IRR ≤0.05).^5^ All analyses were conducted in R version 4.4.0.

Sensitivity analyses included excluding large sites one at a time, excluding sites lacking pre-PCV data and excluding sites switching between PCV10 and PCV13.

This activity was reviewed by the Johns Hopkins Institutional Review Board (IRB) and CDC, deemed research not involving human subjects and exempt from IRB oversite, and was conducted consistent with applicable federal law and CDC policy. The funders had no role in the design of the study; in the collection, analyses, or interpretation of data; in the writing of the manuscript; or in the decision to publish the results.

## Results

Of the 47 sites participating in PSERENADE with eligible IPD incidence data,^8^ 42 sites (including two Canadian sites contributing data to both products) from 30 countries were included in these meningitis analyses, contributing 52,891 meningitis cases occurring between 1991–2019 (median number per site: PCV13 sites n=222, range 8–17,610; PCV10 sites n=194, range 8–7381) (Table 1 and Table S1). Sites excluded from all analyses were due to concurrent PCV10 and PCV13 use (n=4), meningitis not distinguished from other IPD cases (n=2), clinically defined meningitis cases confirmed by blood culture only (n=1), or changes in meningitis surveillance or temporal events that could bias PCV impact estimations (n=4). Among included sites, data from one or two age groups were excluded from analyses from twelve sites (Table S1). Median proportion of cases fully serotyped among 12 PCV10 sites eligible for VT and NVT analyses was 93.3% (range: 82.8–96.9%) and 89.1% (range: 50.0–99.2) among the 28 eligible PCV13 sites (Table 1 and Supplementary Table 1).

**Table 1:** Characteristics of surveillance sites and surveillance data included in CSF+ pneumococcal meningitis analyses estimating change in incidence of pneumococcal meningitis post-PCV10/13 introduction.

Characteristic		<5 Years		5-17 Years		>18 Years		All Ages^a^	
		PCV10	PCV13	PCV10	PCV13	PCV10	PCV13	PCV10	PCV13
*A. Number of Surveillance Sites (Column %)*									
Total Sites		12	30	10	25	12	25	13	31
Sites included in VT/NVT analyses		11	27	9	22	8	24	12	28
Region^b^									
Asia		0 (0%)	3 (10.0%)	0 (0%)	2 (8.0%)	0 (0%)	3 (12.0%)	0 (0%)	4 (12.9%)
Europe		3 (25.0%)	13 (43.3%)	3 (30.0%)	12 (48.0%)	4 (33.3%)	12 (48.0%)	4 (30.8%)	13 (41.9%)
Latin America & the Caribbean		3 (25.0%)	0 (0%)	3 (30.0%)	0 (0%)	3 (25.0%)	0 (0%)	3 (23.1%)	0 (0%)
North America		2 (16.7%)	9 (30.0%)	2 (20.0%)	7 (28.0%)	2 (16.7%)	7 (28.0%)	2 (15.4%)	9 (29.0%)
Northern Africa & Western Asia		0 (0%)	2 (6.7%)	0 (0%)	2 (8.0%)	0 (0%)	1 (4.0%)	0 (0%)	2 (6.5%)
Oceania		3 (25.0%)	1 (3.3%)	1 (10.0%)	1 (4.0%)	2 (16.7%)	1 (4.0%)	3 (23.1%)	1 (3.2%)
Sub-Saharan Africa		1 (8.3%)	2 (6.7%)	1 (10.0%)	1 (4.0%)	1 (8.3%)	1 (4.0%)	1 (7.7%)	2 (6.5%)
World Bank Income Level^c^									
High		9 (75.0%)	26 (86.7%)	8 (80.0%)	23 (92.0%)	10 (83.3%)	24 (96.0%)	10 (76.9%)	27 (87.1%)
Upper Middle		1 (8.3%)	1 (3.3%)	0 (0%)	1 (4.0%)	0 (0%)	1 (4.0%)	1 (7.7%)	1 (3.2%)
Lower Middle		1 (8.3%)	2 (6.7%)	1 (10.0%)	1 (4.0%)	1 (8.3%)	0 (0%)	1 (7.7%)	2 (6.5%)
Low		0 (0%)	1 (3.3%)	0 (0%)	0 (0%)	0 (0%)	0 (0%)	0 (0%)	1 (3.2%)
Gavi Status^d^									
Gavi		1 (8.3%)	2 (6.7%)	1 (10.0%)	0 (0%)	1 (8.3%)	0 (0%)	1 (7.7%)	2 (6.5%)
Non-Gavi		10 (83.3%)	28 (93.3%)	8 (80.0%)	25 (100%)	10 (83.3%)	25 (100%)	11 (84.6%)	29 (93.5%)
Pre-PCV under 5 years pneumococcal disease burden (2000)^e^									
Low		7 (58.3%)	26 (86.7%)	6 (60.0%)	23 (92.0%)	7 (58.3%)	24 (96.0%)	7 (53.8%)	27 (87.1%)
Medium		2 (16.7%)	0 (0%)	2 (20.0%)	0 (0%)	3 (25.0%)	0 (0%)	3 (23.1%)	0 (0%)
High		2 (16.7%)	4 (13.3%)	1 (10.0%)	2 (8.0%)	1 (8.3%)	1 (4.0%)	2 (15.4%)	4 (12.9%)
Schedule									
3+0		2 (16.7%)	2 (6.7%)	1 (10.0%)	1 (4.0%)	1 (8.3%)	1 (4.0%)	2 (15.4%)	2 (6.5%)
2+1		5 (41.7%)	14 (46.7%)	5 (50.0%)	12 (48.0%)	5 (41.7%)	11 (44.0%)	5 (38.5%)	14 (45.2%)
3+1		3 (25.0%)	7 (23.3%)	2 (20.0%)	5 (20.0%)	3 (25.0%)	6 (24.0%)	3 (23.1%)	8 (25.8%)
2+1/3+1		2 (16.7%)	7 (23.3%)	2 (20.0%)	7 (28.0%)	3 (25.0%)	7 (28.0%)	3 (23.1%)	7 (22.6%)
PCV10/13 catch-up		2 (16.7%)	12 (40.0%)	3 (30.0%)	7 (28.0%)	3 (25.0%)	8 (32.0%)	3 (23.1%)	11 (35.5%)
Prior PCV7 use		6 (50.0%)	24 (80.0%)	5 (50.0%)	21 (84.0%)	5 (41.7%)	20 (80.0%)	6 (46.2%)	24 (77.4%)
Adult vaccine recommendation^f^									
PPSV23		8 (66.7%)	24 (80.0%)	7 (70.0%)	23 (92.0%)	8 (66.7%)	23 (92.0%)	8 (61.5%)	25 (80.6%)
PCV13		3 (25.0%)	13 (43.3%)	2 (20.0%)	12 (48.0%)	3 (25.0%)	11 (44.0%)	3 (23.1%)	13 (41.9%)
*B. Number of Surveillance Years per Site, Median (Range)*									
Total		14 (3−24)	13(5−27)	14 (6−24)	13 (5−27)	11 (4−24)	12 (2−27)	33 (3−72)	33 (2−81)
Pre-PCV period		6 (2−16)	3 (1−10)	7 (2−16)	3 (1−10)	6 (2−16)	3 (1−10)	14 (0−48)	6 (0−30)
PCV7 period		4 (2−9)	4 (1−10)	5 (2−9)	4 (1−10)	5 (2−9)	4 (1−10)	0 (0−27)	9 (0−30)
PCV10/13 period		6 (2−9)	8 (1−10)	6 (2−9)	8 (1−10)	6 (2−9)	8 (1−10)	6 (2−9)	8 (1−10)
*C. Number of CSF Cases*									
All years	Total	2849	10,168	1549	3711	5546	29,068	9944	42,947
	Median per site (range)	62 (5−2204)	70 (4−3771)	30 (2−1365)	28 (1−2058)	116 (1−3812)	227 (1−11,781)	194 (8−7381)	222 (8−17610)
Pre-PCV period	Total	1960	3954	988	1158	2256	6579	5204	11,691
	Median per site (range)	36 (0−1623)	34 (0−1879)	8 (0−917)	7 (0−812)	62 (0−1877)	85 (0−3617)	50 (0−4417)	10 (0−6308)
PCV7 period	Total	126	2894	36	994	319	7555	481	11,443
	Median per site (range)	26 (4−36)	28 (0−724)	7 (2−13)	12 (0−421)	31 (1−200)	92 (1−2295)	0 (0−249)	44 (0−3353)
PCV10/13 period	Total	763	3320	525	1559	2971	14,934	4259	19,813
	Median per site (range)	16 (0−581)	29 (1−1168)	4 (0−448)	13 (0−825)	72 (0−1935)	184 (0−5956)	64 (0−2964)	80 (1−7949)
*D. Proportion of children vaccinated with PCV10/13 across sites (uptake)*^g^									
Median (range)^i^		94.9 (86.8−99.9)	85.3 (67.8−99.8)	95.4 (86.8−99.9)	85.6 (67.8−99.8)	94.5 (84.6−99.9)	85.6 (67.8−99.8)	94.5 (84.6−99.9)	85.4 (67.8−99.8)
*E. Proportion VT pre-PCV across sites*^h^									
Median (range)		76.7 (64.7−90.3)	89.7 (75.0−100.0)	50.3 (25.0−100.0)	67.6 (25.0−100.0)	52.3 (0.0−100.0)	64.8 (16.7−78.7)	63.6 (55.4−90.3)	75.4 (53.8−84.6)
*F. Proportion of cases serotyped across sites*^i^									
Median (range)		93.3 (82.8−96.9)	89.1 (50.0−99.2)	95.1 (82.8−96.9)	90.0 (50.0−99.2)	94.8 (82.8−96.9)	89.1 (64.0−99.2)	93.3 (82.8−96.9)	89.1 (50.0−99.2)

a Quebec sites (Quebec-Nunavik and Quebec excluding Nunavik) contributed data to both PCV10 and PCV13 models for the respective years each vaccine was used, but their cases and years of data are counted only once in the total number of sites to avoid duplication.

b United Nations (UN) regions adapted from UN Statistics Division.^25^

c World Bank income level as of November 2020.^26^

d Gavi countries are those that are eligible or have graduated.

e Strata were defined as fewer than 300 cases per 100,000 children (low burden), 300 to fewer than 2000 cases per 100,000 children (medium burden), or 2000 or more cases per 100,000 children (high burden). Countries missing any or all incidence rates were categorized as “Unknown”.

f Any recommendation (high risk individuals only or universal) for any adult age group. Where data were available, adult PPSV23 and PCV13 uptake was generally low.

g Annual PCV uptake estimates provided by the surveillance site for PCV10/13 years of data included in analyses. Uptake is for the primary series of PCV by 12 months of age (if available, for some sites up to 15 months of age), excluding the year of vaccine rollout. If unavailable, annual PCV uptake estimates provided by the surveillance site for the primary series plus the booster dose by 23 months of age, excluding the year of vaccine rollout used. If PCV uptake data from the surveillance site unavailable, WHO and UNICEF Estimates of National Immunization Coverage (WUENIC) PCV uptake, excluding the year of vaccine rollout used (Table S3). Medians are generated using the median uptake for each site across years of PCV10/13 data included in the analyses, excluding the year of PCV10/13 introduction.

h PCV10 serotypes for PCV10 sites and PCV13 serotypes for PCV13 serotypes in the pre-any PCV period (i.e., prior to PCV7, if used).

i The number of fully serotyped cases (excluding not serotyped, untypeable, pool, serogroup-only, and undistinguished cases) / the total number of cases reported.

Although all regions were represented in analyses, few sites contributed data from Asia (125 cases, 4 sites), Africa (17,738 cases, 4 sites (2 meningitis-belt)), and Latin America and the Caribbean (7748 cases, 3 sites). Few sites were from Gavi-eligible (n=3), low-income (n=1), or middle-income countries (n=3 lower middle, n=2 upper middle). More data were available to evaluate PCV13 (31 sites, 42,947 cases) than PCV10 (13 sites, 9944 cases; Table 1). More PCV13 sites (77.4%) had previously used PCV7 than PCV10 sites (46.2%). Most infant PCV schedules included a booster dose (90.9%), and 31.8% of sites introduced PCV10/13 with a catch-up campaign. PCV10/13 uptake (excluding year of rollout) was high (PCV10 sites: median=94.5%, range 84.6–99.9%; PCV13 sites: 85.4%, range 67.8–99.8%). The median proportion of all IPD that was meningitis was 10.7% (IQR: 6.5–16.7%) among children aged <5 years, 8.9% (IQR: 5.9–17.2%) among children 5–17 years and 4.8% (IQR: 3.1–6.9%) among adults ≥18 years. Pre-PCV, the median proportion of meningitis that was VT in children <5 years was 76.7% (IQR 68.7–77.5%) PCV10-type at PCV10 sites and 89.7% (IQR 77.3–78.1%) PCV13-type at PCV13 sites.

### Impact among children <5 years of age

**Fig. 1:**
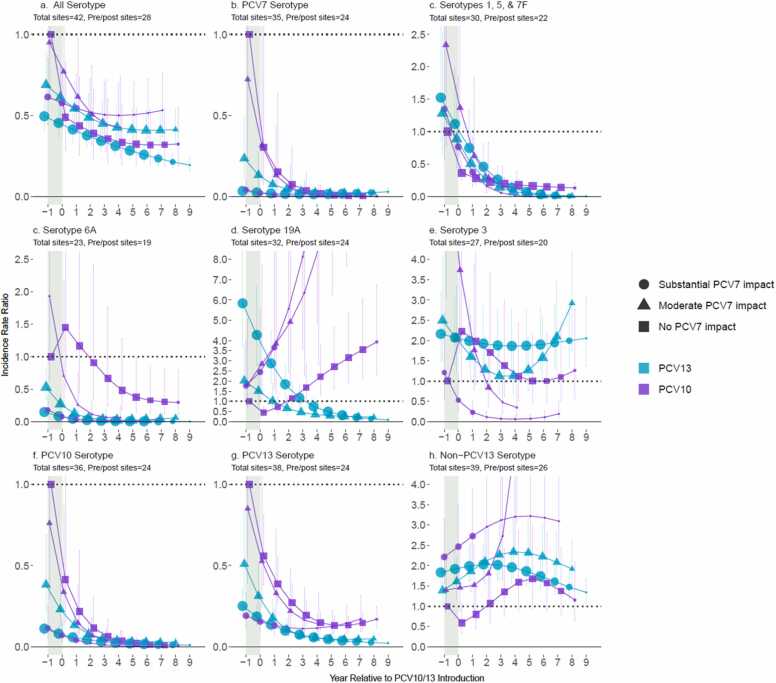
All-site weighted average incidence rate ratios for all serotype pneumococcal meningitis and vaccine/serotype-specific pneumococcal meningitis, comparing the annual post-PCV10/13 incidence rate to the average pre-PCV incidence rate, among children <5 years of age. Footnotes: Y-axis scales differ between figures. PCV10-types include serotypes 1, 4, 5, 6B, 7F, 9V, 14, 18C, 19F, and 23F; PCV13-types include PCV10-types plus serotypes 3, 6A and 19A. Shaded areas indicate the year of PCV10/13 rollout. Estimates at year 0 indicate the change in incidence after the first year of PCV10/13 use. The size of the symbols in the figures reflects the number of sites contributing data to each time point, with larger symbols representing more sites.

Analyses of children <5 years of age included 12 PCV10 sites with 2849 pneumococcal meningitis cases (median per site: 62.5, range 5–2204) and 30 PCV13 sites with 10,168 cases (median per site: 69.5, range 4–3771). After stratifying by degree of prior PCV7 impact (substantial, moderate, or none) analyses included 2–11 sites per stratum (Table S2). For PCV7-using sites, all non-PCV7-types increased prior to PCV10/13 introduction, except ST6A which decreased; ST19A increased more than other non-PCV7-types at sites that later switched to PCV13, but ST19A increases were similar to other NVTs at sites that later switched to PCV10 (Fig. 1, year=−1). Six years post-PCV10/13 introduction, All-ST meningitis had declined 48–74% relative to pre-PCV incidence across vaccine products and prior PCV7 impact strata, with no clear differences by product (Fig. 1a). PCV7-types and PCV10-non-PCV7-types were nearly eliminated (declines ranged between 97–99%), and ST6A declined at both PCV10 (67–98%) and PCV13 (97–100%) sites. PCV13-type meningitis declined more at PCV13 sites (96%) than at PCV10 sites (86–87%) due to ST19A, which declined 68–74% at PCV13 sites but increased over 3-fold at PCV10 sites. Serotypes 1, 5 and 7F (combined) fell faster than ST19A at PCV13 sites. ST19A took 3 years longer to decline below pre-PCV levels due to the higher degree of ST19A replacement disease following PCV7 use than for other NVTs, but was eventually also nearly eliminated in PCV13 sites (87–100% at year 8). No consistent trends were observed for ST3 for either product (Fig. 1e). Further increases in non-PCV13-type meningitis beyond post-PCV7 increases were observed after the switch to PCV10/13 for 3 of 4 strata that previously used PCV7; replacement generally peaked after approximately 6 years of any PCV use at 1.6–3.2-fold increase relative to pre-PCV across strata. Heterogeneity in non-PCV13-type IRRs across sites within a stratum was similar to the range across strata (IQR of site-specific modeled IRRs at year six: 1.5–3.0-fold increase).

### Impact among children 5-17 years of age

**Fig. 2:**
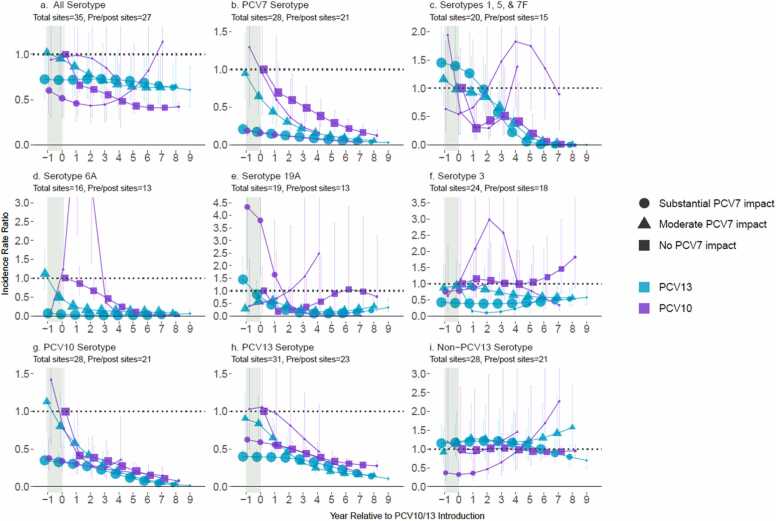
All-site weighted average incidence rate ratios for all serotype pneumococcal meningitis and vaccine/serotype-specific pneumococcal meningitis, comparing the annual post-PCV10/13 pneumococcal meningitis incidence rate to the average pre-PCV incidence rate, among children 5–17 years of age. Footnotes: Y-axis scales differ between figures. PCV10-types include serotypes 1, 4, 5, 6B, 7F, 9V, 14, 18C, 19F, and 23F; PCV13-types include PCV10-types plus serotypes 3, 6A and 19A. Shaded areas indicate the year of PCV10/13 rollout. Estimates at year 0 indicate the change in incidence after the first year of PCV10/13 use. The size of the symbols in the figures reflects the number of sites contributing data to each time point, with larger symbols representing more sites.

There were fewer pneumococcal meningitis cases among children aged 5–17 years compared to <5 years: 1549 from 10 PCV10 sites (median per site: 30, range 2–1365) and 3711 from 25 PCV13 sites (median: 28, range 1–2058; Table S2). Net declines in all pneumococcal meningitis six years post-PCV10/13 introduction were lower for children aged 5–17 years (range 35–62% across strata, Fig. 2) than <5 years (48–74%). Although patterns for VTs were generally similar between these age groups, rates of declines were slower among children aged 5–17 years. For NVTs, however, replacement disease was generally low or not observed (IRRs ranged 0.92–1.26) among children aged 5–17 years, including for ST19A at PCV10 sites for which two PCV10 strata had only one ST19A case post-PCV10 introduction (one in year 0 and one year 4) while the third PCV10 strata (no prior PCV7 use) had annual IRRs ≤1.05 through year 8 post-introduction (Supplementary Figure 22).

### Impact among adults ≥18 years of age

**Fig. 3:**
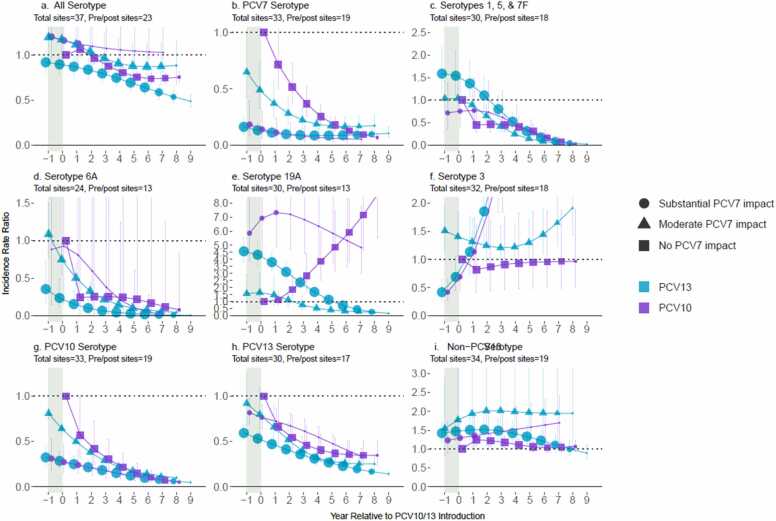
All-site weighted average incidence rate ratios for all serotype pneumococcal meningitis and vaccine/serotype-specific pneumococcal meningitis, comparing the annual post-PCV10/13 pneumococcal meningitis incidence rate to the average pre-PCV incidence rate, among adults ≥18 years of age. Footnotes: Y-axis scales differ between figures. PCV10-types include serotypes 1, 4, 5, 6B, 7F, 9V, 14, 18C, 19F, and 23F; PCV13-types include PCV10-types plus serotypes 3, 6A and 19A. Shaded areas indicate the year of PCV10/13 rollout. Estimates at year 0 indicate the change in incidence after the first year of PCV10/13 use. In adults, the model that best fit the data was the one with one year time lag as compared to the year of PCV roll out as for children aged <5 years. For sites with moderate or substantial PCV7 impact, the time lag started at PCV7 introduction (not shown). For sites with no PCV7 impact, the start of the time lag was from year −1, indicating the pre-PCV period (i.e., IRR=1) for children aged <5 years and from year 0 for all other age groups. The size of the symbols in the figures reflects the number of sites contributing data at each time point, with larger symbols representing more sites.

For adults ≥18 years of age, 5653 pneumococcal meningitis cases from 13 PCV10 sites and 29,187 cases from 26 PCV13 sites were included, most of whom were aged <50 years (age distribution: 18–49: 59%; 50–64: 26%; ≥65 years of age: 15%; Supplementary Figure 1). Declines in All-ST pneumococcal meningitis were smaller in adults (0-36%) than in children <5 or 5–17 years despite similar declines in VTs and low to no increases in non-PCV13-types as children 5–17 years, (Fig. 3). ST19A increased over 5-fold relative to pre-PCV levels in adults ≥18 years of age at PCV10 sites but did not increase further beyond post-PCV7 increases. Among sites that used PCV7, increases in non-PCV7-types (excluding ST6A) prior to PCV10/13 introduction were greater for adults than children 5–17 years and more similar to children <5 years.

### Sensitivity analyses

Sensitivity analyses that added clinically-defined blood culture-positive meningitis cases to the analyses, excluded one large site at a time (i.e., Active Bacterial Core Surveillance Network, USA; England, UK; South Africa; France; Brazil), excluded sites lacking pre-PCV data, or excluded sites that switched between PCV10 and PCV13 (i.e., Quebec excluding Nunavik and Quebec-Nunavik, Canada) did not produce meaningful differences (data not shown).

## Discussion

This study evaluated long-term direct and indirect effects of PCV10 or PCV13 used in national infant immunization programs on pneumococcal meningitis in all ages globally. By six years after PCV10/13 introduction, pneumococcal meningitis incidence declined 48–74% relative to pre-PCV among children aged <5 years, 35–62% among children 5–17 years and 0–36% among adults 18 years and older. Large (83–99%) declines in VT pneumococcal meningitis were observed across all age groups. Non-PCV13-type meningitis incidence increased approximately 2-fold relative to pre-PCV in children <5 years but did not increase in most strata among older children and adults. However, ST19A increased more than 3-fold at PCV10 sites among both children <5 years and adults.

Patterns of replacement disease were compared between meningitis and all-IPD,^5^ which represented primarily pneumonia and sepsis cases. Increases in non-PCV13-type meningitis were generally lower and more time-limited relative to all-IPD non-PCV13-type increases.^5^ Replacement with non-PCV13-type meningitis was absent for most strata for older children and adults, as reported previously for high-income countries,^10^, ^11^ in contrast to all IPD for which non-PCV13-types increased 1.5–2.5-fold post-PCV10/13.^5^ Among children aged <5 years, non-PCV13-type meningitis increased initially but peaked in most strata by 5 years post-PCV10/13 introduction, whereas non-PCV13-type all IPD continued increasing throughout the post-PCV10/13 period in most strata. In contrast, for ST19A at PCV10 sites, increases were larger for meningitis than all IPD (3-fold vs 1.6–2.3-fold) among children <5 years,^5^ but could be due to small case meningitis counts pre-PCV leading to instability as confidence intervals were wide and inconsistent among other age groups. Evidence from other studies comparing replacement disease between syndromes was limited and only described short-term (≤4 years post-PCV) impact.^6^, ^10^, ^12^ A study evaluating children aged <5 years in Israel found non-PCV13-type replacement was greater for meningitis than for non-meningitis,^6^ while a study in Germany evaluating non-PCV7-types during PCV7 use found the reverse for all age groups <16 years of age, although there was little PCV7-type meningitis to prevent and by definition replacement can only occur when VTs are eliminated.^12^ Less replacement disease for meningitis than for pneumonia may mean PCVs covering serotypes beyond PCV13 may make greater inroads in reducing pneumococcal meningitis in all ages.^13^

Other evidence that serotype-specific impact may differ between pneumococcal syndromes included greater declines in ST19A for meningitis (this study) than all IPD at PCV13 sites for all age groups (other PSERENADE results ^5^). Previous studies have identified serotype differences between pneumococcal syndromes with some (e.g., ST18C, ST12F, ST19F and ST6B) more likely to cause meningitis than non-meningitis IPD, whereas others (e.g., ST1) were more associated with non-meningitis IPD.^6^, ^14^, ^15^ A previous PSERENADE analysis of all ST1 IPD observed outbreaks persisting three years after PCV10/13 introduction in all age groups^16^ but ST1 meningitis cases were few and without evidence of outbreaks. However, data were limited from the meningitis belt where ST1 meningitis incidence is high and persistent ST1 meningitis has been observed post-PCV10/13,^17^ necessitating schedule changes in Burkina Faso to include a booster dose. Further long-term within-study comparisons between meningitis and non-meningitis IPD would help clarify differences.

While sites using PCV13 had the largest net impact on all pneumococcal meningitis in both children <5 years and adults, we observed no consistent patterns for all pneumococcal meningitis favoring PCV10 vs PCV13. Differences between strata at the time of PCV10/13 introduction as indicated by the degree of prior PCV7 impact indicate likely differences in serotype distribution between sites that we could not account for. These serotype distribution differences may have affected the choice of PCV10 vs PCV13. At PCV10 sites, increasing ST19A meningitis with little to no increases in non-PCV13-type meningitis after the PCV7 period and ST19A being a top serotype at mature PCV10 sites^13^ suggest that switching to a ST19A-containing higher-valency PCV could further reduce pneumococcal meningitis. Availability of several ST19A-containing PCVs, including Serum Institute of India’s (PNEUMOSIL) 10-valent PCV and other higher valency PCVs, provide options. However, vaccine choice and access are anticipated to be more limited for low- and middle-income countries; ensuring they have access to PCVs that protect against ST19A and serotypes beyond PCV13-types will be important for equity and reducing global pneumococcal mortality and disease burden. A separate analysis evaluating the distribution of remaining serotypes in mature settings estimated that after excluding ST3, PCV15 covered 17–35% of remaining pneumococcal meningitis in all ages and 20- to 25-valent PCVs covered approximately 40–50%.^13^

Although the serotype distribution of remaining meningitis cases <5 years of age after long-term PCV10/13 use showed ST3 accounted for a lower percentage at PCV13 sites (4%, ranked eighth most common remaining serotype) than at PCV10 sites (7.4%, ranked third),^13^ we found no consistent changes in ST3 meningitis incidence post-PCV10/13, similar to all IPD,^5^ suggesting no protection with PCV13 and no replacement with PCV10 use. At PCV10 sites, ST6A meningitis declined nearly to the level observed at PCV13 sites in all ages, attributed to cross-protection from ST6B. We were unable to estimate cross-protection from ST6A to ST6C due to few ST6C cases at PCV13 sites; however, ST6C was less common at PCV13 sites (≤2.2% of remaining pneumococcal meningitis) than at PCV10 sites (approximately 10%) where it was the second or third most common serotype in all ages,^13^ suggesting PCV13 prevents ST6C meningitis but PCV10 does not.

This study has several limitations. First, the trend in pneumococcal meningitis incidence (e.g., whether cases were increasing or decreasing) prior to PCV introduction was needed to estimate what would have occurred had PCV not been introduced (the counterfactual), but was difficult to model due to small annual case counts, especially for serotype-specific meningitis incidence. Therefore, trends pre-PCV were estimated using all IPD cases. This may bias impact estimates if pre-PCV trends differed between blood culturing and CSF testing practices or between syndromes. However, its impact would likely be minimal since data from sites with suspected changes in surveillance systems that could produce sizeable errors in the counterfactual were excluded and no notable differences between all pneumococcal meningitis incidence and all IPD were observed pre-PCV at sites included in analyses. Additionally, when vaccine effects are large, such as those occurring after 6 years of PCV use, the magnitude of any remaining differences between counterfactuals would be exceeded by vaccine effects, thus having minimal influence, and averaging across multiple sites further minimizes potential bias. A second limitation is that, although we tried to incorporate all available data globally and our analysis included data from 42 surveillance sites representing all global regions, there were few data from Asia, Africa, meningitis-belt countries, and sites with 3+0 schedules so we could not provide estimates stratified by region or schedule. Still, Burkina Faso, a country that did not participate, found similar results to ours.^18^ Third, we were unable to account for the many potentially influential factors that likely contribute to heterogeneity between sites, including changes in adult immunization, prevalence of comorbidities such as HIV and other risk factors, pre-PCV serotype distribution, degree of prior PCV7 influence within the moderate impact strata, PCV schedule, PCV catch-up programs, PCV uptake (beyond eligibility threshold), disease incidence, lab methods and under-five mortality rate. However, despite heterogeneity in these factors between sites, there was homogeneity across sites within strata in the directionality of long-term results. Furthermore, explorations into the factors where possible revealed no major influence on long-term directionality or fully explained the heterogeneity between sites (data not shown). Others have hypothesized global heterogeneity is caused by differential surveillance and clinical practices, transmission dynamics, prevalence and degree of changes in population risk factors, pathogen evolution, and pre-PCV serotype distributions.^19^, ^20^, ^21^, ^22^ Finally, analyses directly comparing meningitis versus non-meningitis IPD were not done due to resource constraints; excluding meningitis cases from all IPD and synthesizing within-site comparisons may have reduced potential confounding by site and further distinguished syndromic differences.

The main strengths of this study were the amount of long-term, well-characterized meningitis surveillance data from across the globe and the application of standardized analytic methods for each site. This facilitated comparisons between products, robust serotype-specific analyses and analyses by age group and syndrome that would not be feasible for most sites individually. Having results after long-term PCV10/13 use enabled estimation of their full effects and understanding the dynamics of replacement disease.

Our findings underscore the important role of PCVs in reducing pneumococcal meningitis burden globally across all age groups. Since data collection for this study occurred there have been other changes, including global introduction of other PCV products; COVID-19 pandemic disruptions of vaccination programs, IPD surveillance functioning and pneumococcal epidemiology^23^; increases in adult vaccination; and a switch to a novel 1+1 schedule in the UK.^24^ As such, the landscape of pneumococcal meningitis is expected to continue to evolve and ongoing surveillance will be critical to help evaluate and optimize use of PCVs.

## Disclaimer

The findings and conclusions in this report are those of the authors and do not necessarily represent the official position of the Centers for Disease Control and Prevention or the World Health Organization (WHO).

## Funding

The PSERENADE project is funded by the 10.13039/100000865Bill and Melinda Gates Foundation as part of the World Health Organization Pneumococcal Vaccines Technical Coordination Project, grant number INV-010429/OPP1189065.

## Author contributions

Conceptualization: KLO, MDK, DRF, KH.

Methodology: MDK, SLZ, DRF, KH, YY, JCB.

Software and Analysis: YY, JCB, SLZ.

Visualization: YY, MX.

Writing—Original Draft: YY, MDK, CH.

Writing—Review and Editing: JCB, KH, MKH, DRF, ALC, and all site authors who reviewed site-specific and meta results and draft manuscripts.

Supervision: KLO, MDK, KH, ALC.

Project Administration: JCB, MGQ, CH.

Funding Acquisition: KLO, MDK, ALC, KH.

Data Curation: JCB, MGQ, MKH, YY, EWK, and all site authors who shared prepared datasets for the project.

All authors provided input for the analytic methodology and critically reviewed results. All authors have read and agreed to the published version of this manuscript.

## Declaration of Competing Interest

The authors declare the following financial interests/personal relationships which may be considered as potential competing interests: KH conducted the study and analyses while working at the Johns Hopkins School of Public Health but became an employee at Pfizer, Inc. on 26 October 2020. MDK reports grants from Merck, personal fees from Merck, and grants from Pfizer, outside the submitted work. JAS reports grants from the Bill & Melinda Gates Foundation, the Wellcome Trust, the UK MRC, National Institute of Health Research, outside the submitted work. MCB reports lectures fee from MSD outside from submitted work. ML has been a member of advisory boards and has received speakers honoraria from Pfizer and Merck. German pneumococcal surveillance has been supported by Pfizer and Merck. SD reports grant from Pfizer, outside the submitted work. KA reports a grant from Merck, outside the submitted work. AM reports research support to her institution from Pfizer and Sanofi and personal fees for advisory board membership and webinar presentations from AstraZeneca, GSK, Merck, Moderna, Novavax Pfizer, Seqirus. SNL performs contract research for GSK, Pfizer, Sanofi Pasteur on behalf of St. George’s University of London, but receives no personal remuneration. IY stated she was a member of mRNA-1273 study group and has received funding to her institution to conduct clinical research from BioFire, MedImmune, Regeneron, PaxVax, Pfizer, GSK, Merck, Novavax, Sanofi-Pasteur, and Micron. RD has received grants/research support from Pfizer, Merck Sharp & Dohme and Medimmune; has been a scientific consultant for Pfizer, MeMed, Merck Sharp & Dohme, Biondvax and GSK; had served on advisory boards of Pfizer, Merck Sharp & Dohme Biondvax and GXRD has received grants/research support from Pfizer, Merck Sharp & Dohme and Medimmune; has been a scientific consultant for Pfizer, MeMed, Merck Sharp & Dohme, Biondvax and GSK; and has been a speaker for Pfizer, Astra-Zeneca, GSK. LLH reports research grants to her institution from GSK, Pfizer and Merck. JDK has received an unrestricted grant-inaid from Pfizer Canada that supports, in part, the CASPER invasive pneumococcal disease surveillance project. MH received an educational grant from Pfizer AG for partial support of this project. However, Pfizer AG had no role in the data analysis and content of the manuscript. JCS reports had received assistance from Pfizer for attending to scientific meetings outside the submitted work. EV reports grants from French public health agency, during the conduct of the study; grants from Pfizer, grants from Merck, outside the submitted work. KGK reports grants from GlaxoSmithKline Biologicals SA, outside of the submitted work. KA reports a grant from Merck, outside the submitted work. SCGA received travel grant from Pfizer. All other authors declare no competing interests.

## Figures and Tables

**Table A1:** PSERENADE Team.

Name	Affiliation
Anne von Gottberg	Centre for Respiratory Diseases and Meningitis, National Institute for Communicable Diseases of the National Health Laboratory Service, Sandringham, 2192 Johannesburg, South Africa; School of Pathology, Faculty of Health Sciences, University of the Witwatersrand, Braamfontein 2000, Johannesburg, South Africa
Nina M. van Sorge	Medical Microbiology and Infection Prevention, Netherlands Reference Laboratory for Bacterial Meningitis, Amsterdam UMC, location AMC, University of Amsterdam, 1105 AZ Amsterdam, The Netherlands
Mignon du Plessis	Centre for Respiratory Diseases and Meningitis, National Institute for Communicable Diseases of the National Health Laboratory Service, Sandringham, 2192 Johannesburg, South Africa
Sara de Miguel	Epidemiology Department, Dirección General de Salud Pública, 28009 Madrid, Spain; CIBER de Enfermedades Respiratoris (CIBERES), 28029 Madrid, Spain
Linda de Gouveia	Centre for Respiratory Diseases and Meningitis, National Institute for Communicable Diseases of the National Health Laboratory Service, Sandringham, 2192 Johannesburg, South Africa
Khalid Zerouali	Department of Microbiology, Faculty of Medicine and Pharmacy, Hassan II University of Casablanca, Casablanca 20000, Morocco; Bacteriology-Virology and Hospital Hygiene Laboratory, Ibn Rochd University Hospital Centre, Casablanca 20250, Morocco
Delphine Viriot	Santé Publique France, the French National Public Health Agency, 94410 Saint- Maurice, France
Didrik F. Vestrheim	Department of Public Health, Ministry of Health and Care Services, Oslo, Norway
Maija Toropainen	Department of Health Security, Finnish Institute for Health and Welfare, 00271 Helsinki, Finland
Koh Cheng Thoon	KK Women’s and Children’s Hospital, 229899, Singapore
Catherine G. Sutcliffe	Johns Hopkins Bloomberg School of Public Health, Baltimore, MD 21205, United States
Shigeru Suga	Infectious Disease Center and Department of Clinical Research, National Hospital Organization Mie Hospital, Tsu, Mie 514−0125, Japan
Kevin J. Scott	Bacterial Respiratory Infection Service, Scottish Microbiology Reference Laboratory, NHS GG&C, G31 2ER Glasgow, United Kingdom
Heidi M. Soeters	World Health Organization, 1202 Geneva, Switzerland
Leah J. Ricketson	Department of Pediatrics, University of Calgary, Calgary Alberta T3B 6A8, Canada
Rita Reyburn	Murdoch Children’s Research Institute, Parkville VIC 3052, Australia
Marie-Cecile Ploy	University Hospital Centre Limoges, Regional Observatories for Pneumococci, 87000 Limoges, France
Tamara Pilishvili	National Center for Immunizations and Respiratory Diseases, Centers for Disease Control and Prevention, Atlanta, GA 30329, USA
Kate Pennington	Communicable Disease Epidemiology and Surveillance Section, Office of Health Protection, Australian Government Department of Health, 2606 Canberra ACT, Australia
Stephen I. Pelton	Boston University Schools of Medicine and Public Health, Boston, MA 02118, United States
Carmen Muñoz-Almagro	Molecular Microbiology Department, Hospital Sant Joan de Déu Research Institute, 08950 Esplugues de Llobregat, Barcelona, Spain; Medicine Department, Universitat Internacional de Catalunya, 08017 Barcelona, Spain; CIBER Epidemiología y Salud Pública, (CIBERESP), 28029 Madrid, Spain
Tuya Mungun	National Center of Communicable Diseases (NCCD), Ministry of Health, Bayanzurkh district, 13336 Ulaanbaatar, Mongolia
Kimberley McMahon	Centre for Disease Control, Department of Health and Community Services, Darwin City NT 8000, Australia
Brigitte Lefebvre	Laboratoire de Santé Publique du Québec, Sainte-Anne-de-Bellevue, Quebec H9X 3R5, Canada
Miwako Kobayashi	National Center for Immunizations and Respiratory Diseases, Centers for Disease Control and Prevention, Atlanta, GA 30329, USA
Ilias Hossain	Medical Research Council Unit The Gambia at London School of Hygiene & Tropical Medicine, PO Box 273, Banjul, The Gambia
Yvonne Galloway	Epidemiology Team, Institute of Environmental Science and Research, Porirua, 5022 Wellington, New Zealand
Helga Erlendsdottir	Department of Clinical Microbiology, Landspitali - The National University Hospital, Hringbraut, 101 Reykjavik, Iceland
Janepsy Díaz	Instituto de Salud Pública de Chile, 7780050 Santiago, Santiago Metropolitan, Chile
Elina Dimina	Centre for disease prevention and control of Latvia, Riga, 1005, Latvia
Martina Del Manso	Department of Infectious Diseases, Italian National Institute of Health (Istituto Superiore di Sanità, ISS), 00161 Rome, Italy
Geneviève Deceuninck	Quebec University Hospital Research Centre, Québec, QC G1V 4G2, Canada
Tine Dalby	Infectious Disease Epidemiology and Prevention, Statens Serum Institut, DK−2300 Copenhagen S, Denmark
	Department of Bacteria, Parasites and Fungi, Statens Serum Institut, Copenhagen, Denmark
Mary Corcoran	Irish Meningitis and Sepsis Reference Laboratory, Children’s Health Ireland at Temple Street, Temple Street, Dublin 1, D01 YC76, Ireland; Royal College of Surgeons in Ireland, Dublin, Ireland
Kin-Hung Chow	Department of Microbiology and Carol Yu Centre for Infection, Queen Mary Hospital, The University of Hong Kong, Hong Kong SAR, China
Lucia Pastore Celentano	European Centre for Disease Prevention and Control, 169 73 Solna, Sweden
Jesús Castilla	Instituto de Salud Pública de Navarra - IdiSNA, 31003 Pamplona, Navarra, Spain; CIBER Epidemiología y Salud Pública, (CIBERESP), 28029 Madrid, Spain
Carrie L. Byington	University of Utah Department of Pediatrics (emeritus), Salt Lake City, UT 84108, United States; University of California Health System, Oakland, CA 94607, United States
Dana Bruden	Arctic Investigations Program, Division of Preparedness and Emerging Infections, National Center for Emerging and Zoonotic Infectious Diseases, Centers for Disease Control and Prevention, Anchorage, AK 99508, United States
Rita Born	Federal Office of Public Health, 3097 Liebefeld, Switzerland
Michelle Ang	National Public Health Laboratory, National Centre for Infectious Diseases, Singapore 308442, Singapore
Zahin Amin-Chowdhury	Immunisation and Countermeasures Division, UK Health Security Agency, NW9 5EQ, London, United Kingdom
Samanta C. G. Almeida	National Laboratory for Meningitis and Pneumococcal Infections, Center of Bacteriology, Institute Adolfo Lutz (IAL), São Paulo, 01246−902, Brazil
Pedro Alarcon	Instituto de Salud Pública de Chile, 7780050 Santiago, Santiago Metropolitan, Chile
Toronto Invasive Bacterial Diseases Network	C/O Sinai Health, 600 University Avenue, Toronto, Ontario, M5G 1×5, Canada

**Table A2:** Acknowledgment List.

**PSERENADE Technical Advisory Group**
Thomas Cherian
William P. Hausdorff
Marc Lipsitch
Shabir A. Madhi
Elizabeth Miller
Catherine Satzke
Cynthia G. Whitney
Ron Dagan
**World Health Organization**
Jenny A. Walldorf
**Johns Hopkins University**
Meagan E. Peterson
Jenna N. Sinkevitch
Yunfeng Cao
Peggy Gross
Donna Hesson
Ananya Kumar
Kate Perepezko
Francesca Schiaffino Salazar
Daniel Stephens
Anju Ogyu
**Dirección General de Salud Pública, Comunidad de Madrid, Spain**
Luis García Comas
Maria Ordobás Gavín
**Department of Infectious Diseases, Italian National Institute of Health (Istituto Superiore di Sanità, ISS), Rome, Italy**
Flavia Riccardo
**Mohammed VI University of Sciences and Health, Mohammed VI Higher Institute of Biosciences and Biotechnologies (UM6SS), Casablanca, Morocco**
Idrissa Diawara
**Surveillance and Public Health Emergency Response, Public Health Agency of Catalonia, Barcelona, Spain**
Sonia Broner
Conchita Izquierdo
Desiree Henares
**Epidemiology Department, Epiconcept, France**
Camelia Savulescu
**Australian National Notifiable Diseases Surveillance data were provided by the Office of Health Protection, Australian Government Department of Health, on behalf of the Communicable Diseases Network Australia and the Enhanced Invasive Pneumococcal Disease Surveillance Working Group.**
**Child Health Research Foundation, Bangladesh**
**National Public Health Organisation, Greece**
**Malawi Liverpool Wellcome Programme, Malawi**
**National Reference Centre for Bacterial Meningitis, National Medicines Institute, Poland**
**National Reference Centre for Pneumococcal and Haemophilus Diseases, Regional Authority of Public Health, Slovak Republic**
**Communicable Diseases Centre, National Institute of Public Health, Slovenia**
**Vaccine Study Center, Kaiser Permanente, United States**

## Data Availability

Restrictions apply to the availability of these data. Data were obtained under data-sharing agreements from contributing surveillance sites and can only be shared by contributing organizations with their permission.

## Footnotes

Appendix B Supplementary data associated with this article can be found in the online version at doi:10.1016/j.jinf.2025.106426.
