# Supplementary material for: Global impact of 10- and 13-valent pneumococcal conjugate vaccines on pneumococcal meningitis in all ages: The PSERENADE project
Source: J Infect. Author manuscript; Available in PMC 2025 Mar 12. (PMC11879884; doi:10.1016/j.jinf.2025.106426)
Supplement: Supplement [file NIHMS2058692-supplement-Supplement.docx]

Supplementary Materials

Table of Contents

[Supplementary Methods 9](#_Toc174092466)

[Supplementary Table 1. Sites in PSERENADE evaluated for inclusion in the CSF+ meningitis incidence analyses by age group 11](#_Toc174092467)

[Supplementary Table 2. Description of surveillance data included in analyses by PCV product and PCV7-impact strata 13](#_Toc174092468)

[Supplementary Table 3. Description of surveillance data included in CSF+ meningitis analyses by site 15](#_Toc174092469)

[Supplementary Table 4. All-site CSF+ meningitis weighted average incidence rate ratios comparing 6 years post-PCV10/13 incidence rate to the average pre-PCV incidence rate 21](#_Toc174092470)

[Supplementary Figure 1. Age distribution of CSF+ meningitis cases by PCV product, region, and age group 23](#_Toc174092471)

[Supplementary Figure 2. Proportion of CSF+ vs. non-CSF+ clinical meningitis cases by site 24](#_Toc174092472)

[Appendix 1: Clinically-defined meningitis all-site weighted average incidence rate ratios comparing the annual post-PCV10/13 incidence rate to the average pre-PCV incidence rate 25](#_Toc174092473)

[Supplementary Figure 3. Children <5 years 25](#_Toc174092474)

[Supplementary Figure 4. Children 5-17 years 26](#_Toc174092475)

[Supplementary Figure 5. Adults 18 years and older 27](#_Toc174092476)

[Appendix 2: All-site IPD vs CSF+ meningitis weighted average incidence rate ratios comparing the annual post-PCV10/13 incidence rate to the average pre-PCV incidence rate 28](#_Toc174092477)

[Supplementary Figure 7. IPD vs CSF+ meningitis, children <5 years 29](#_Toc174092478)

[Supplementary Figure 8. IPD vs CSF+ meningitis, children 5-17 years 30](#_Toc174092479)

[Supplementary Figure 9. IPD vs CSF+ meningitis, adults 18 years and older 31](#_Toc174092480)

[Appendix 3: All-site CSF+ meningitis weighted average incidence rate ratios comparing the annual post-PCV10/13 incidence rate to the average pre-PCV incidence rate 32](#_Toc174092481)

[Supplementary Figure 10. All meningitis, children <5 years 32](#_Toc174092482)

[Supplementary Figure 11. All meningitis, children 5-17 years. 33](#_Toc174092483)

[Supplementary Figure 12. All meningitis, adults >18 years. 34](#_Toc174092484)

[Supplementary Figure 13. PCV7-type, children <5 years. 35](#_Toc174092485)

[Supplementary Figure 14. PCV7-type , children 5-17 years. 36](#_Toc174092486)

[Supplementary Figure 15. PCV7-type, adults >18 years. 37](#_Toc174092487)

[Supplementary Figure 16. ST1, 5, 7F all, children <5 years. 38](#_Toc174092488)

[Supplementary Figure 17. ST1, 5, 7F , children 5-17 years. 39](#_Toc174092489)

[Supplementary Figure 18. ST1, 5, 7F, adults >18 years. 40](#_Toc174092490)

[Supplementary Figure 19. PCV10-type, children <5 years. 41](#_Toc174092491)

[Supplementary Figure 20. PCV10-type, children 5-17 years. 42](#_Toc174092492)

[Supplementary Figure 21. PCV10-type all, adults >18 years. 43](#_Toc174092493)

[Supplementary Figure 22. ST6A, children <5 years. 44](#_Toc174092494)

[Supplementary Figure 23. ST6A, children 5-17 years. 45](#_Toc174092495)

[Supplementary Figure 24. ST6A, adults >18 years. 46](#_Toc174092496)

[Supplementary Figure 25. ST19A, children <5 years. 47](#_Toc174092497)

[Supplementary Figure 26. ST19A, children 5-17 years. 48](#_Toc174092498)

[Supplementary Figure 27. ST19A, adults >18 years. 49](#_Toc174092499)

[Supplementary Figure 28. ST3, children <5 years. 50](#_Toc174092500)

[Supplementary Figure 29. ST3, children 5-17 years. 51](#_Toc174092501)

[Supplementary Figure 30. ST3, adults >18 years. 52](#_Toc174092502)

[Supplementary Figure 31. PCV13-type, children <5 years. 53](#_Toc174092503)

[Supplementary Figure 32. PCV13-type, children 5-17 years. 54](#_Toc174092504)

[Supplementary Figure 33. PCV13-type, adults >18 years. 55](#_Toc174092505)

[Supplementary Figure 34. Non-PCV13 ST, children <5 years. 56](#_Toc174092506)

[Supplementary Figure 35. Non-PCV13 ST, children 5-17 years. 57](#_Toc174092507)

[Supplementary Figure 36. Non-PCV13 ST, adults >18 years. 58](#_Toc174092508)

[Appendix 3: All-site CSF+ meningitis weighted average incidence rate ratios comparing the annual post-PCV10/13 incidence rate to the average pre-PCV incidence rate 59](#_Toc174092509)

[Supplementary Figure 37. All serotype with PCV13 use and substantial PCV7 impact for children <5 years. 59](#_Toc174092510)

[Supplementary Figure 38. All serotype with PCV13 use and moderate PCV7 impact for children <5 years. 60](#_Toc174092511)

[Supplementary Figure 39. All serotype with PCV13 use and no PCV7 impact for children <5 years. 61](#_Toc174092512)

[Supplementary Figure 40. All serotype with PCV10 use and substantial PCV7 impact for children <5 years. 62](#_Toc174092513)

[Supplementary Figure 41. All serotype with PCV10 use and moderate PCV7 impact for children <5 years. 63](#_Toc174092514)

[Supplementary Figure 42. All serotype with PCV10 use and no PCV7 impact for children <5 years. 64](#_Toc174092515)

[Supplementary Figure 43. All serotype with PCV13 use and substantial PCV7 impact for individuals 5-17 years. 65](#_Toc174092516)

[Supplementary Figure 44. All serotype with PCV13 use and moderate PCV7 impact for individuals 5-17 years. 66](#_Toc174092517)

[Supplementary Figure 45. All serotype with PCV13 use and no PCV7 impact for individuals 5-17 years. 67](#_Toc174092518)

[Supplementary Figure 46. All serotype with PCV10 use and substantial PCV7 impact for individuals 5-17 years. 68](#_Toc174092519)

[Supplementary Figure 47. All serotype with PCV10 use and moderate PCV7 impact for individuals 5-17 years. 69](#_Toc174092520)

[Supplementary Figure 48. All serotype with PCV10 use and no PCV7 impact for individuals 5-17 years. 70](#_Toc174092521)

[Supplementary Figure 49. All serotype with PCV13 use and substantial PCV7 impact for adults >18 years. 71](#_Toc174092522)

[Supplementary Figure 50. All serotype with PCV13 use and moderate PCV7 impact for adults >18 years. 72](#_Toc174092523)

[Supplementary Figure 51. All serotype with PCV10 use and substantial PCV7 impact for adults >18 years. 73](#_Toc174092524)

[Supplementary Figure 52. All serotype with PCV13 use and no PCV7 impact for adults >18 years. 74](#_Toc174092525)

[Supplementary Figure 53. PCV7-type with PCV13 use and substantial PCV7 impact for children <5 years. 75](#_Toc174092526)

[Supplementary Figure 54. PCV7-type with PCV13 use and moderate PCV7 impact for children <5 years. 76](#_Toc174092527)

[Supplementary Figure 55. PCV7-type with PCV13 use and no PCV7 impact for children <5 years. 77](#_Toc174092528)

[Supplementary Figure 56. PCV7-type with PCV10 use and substantial PCV7 impact for children <5 years. 78](#_Toc174092529)

[Supplementary Figure 57. PCV7-type with PCV10 use and moderate PCV7 impact for children <5 years. 79](#_Toc174092530)

[Supplementary Figure 58. PCV7-type with PCV10 use and no PCV7 impact for children <5 years. 80](#_Toc174092531)

[Supplementary Figure 59. PCV7-type with PCV13 use and substantial PCV7 impact for individuals 5-17 years. 81](#_Toc174092532)

[Supplementary Figure 60. PCV7-type with PCV13 use and moderate PCV7 impact for individuals 5-17 years. 82](#_Toc174092533)

[Supplementary Figure 61. PCV7-type with PCV13 use and no PCV7 impact for individuals 5-17 years. 83](#_Toc174092534)

[Supplementary Figure 62. PCV7-type with PCV10 use and substantial PCV7 impact for individuals 5-17 years. 84](#_Toc174092535)

[Supplementary Figure 63. PCV7-type with PCV10 use and moderate PCV7 impact for individuals 5-17 years. 85](#_Toc174092536)

[Supplementary Figure 64. PCV7-type with PCV10 use and no PCV7 impact for individuals 5-17 years. 86](#_Toc174092537)

[Supplementary Figure 65. PCV7-type with PCV13 use and substantial PCV7 impact for adults >18 years. 87](#_Toc174092538)

[Supplementary Figure 66. PCV7-type with PCV13 use and moderate PCV7 impact for adults >18 years. 88](#_Toc174092539)

[Supplementary Figure 67. PCV7-type with PCV10 use and substantial PCV7 impact for adults >18 years. 89](#_Toc174092540)

[Supplementary Figure 68. PCV7-type with PCV10 use and no PCV7 impact for adults >18 years. 90](#_Toc174092541)

[Supplementary Figure 69. ST1, 5, 7F with PCV13 use and substantial PCV7 impact for children <5 years. 91](#_Toc174092542)

[Supplementary Figure 70. ST1, 5, 7F with PCV13 use and moderate PCV7 impact for children <5 years. 92](#_Toc174092543)

[Supplementary Figure 71. ST1, 5, 7F with PCV13 use and no PCV7 impact for children <5 years. 93](#_Toc174092544)

[Supplementary Figure 72. ST1, 5, 7F with PCV10 use and substantial PCV7 impact for children <5 years. 94](#_Toc174092545)

[Supplementary Figure 73. ST1, 5, 7F with PCV10 use and moderate PCV7 impact for children <5 years. 95](#_Toc174092546)

[Supplementary Figure 74. ST1, 5, 7F with PCV10 use and no PCV7 impact for children <5 years. 96](#_Toc174092547)

[Supplementary Figure 75. ST1, 5, 7F with PCV13 use and substantial PCV7 impact for individuals 5-17 years. 97](#_Toc174092548)

[Supplementary Figure 76. ST1, 5, 7F with PCV13 use and moderate PCV7 impact for individuals 5-17 years. 98](#_Toc174092549)

[Supplementary Figure 77. ST1, 5, 7F with PCV13 use and no PCV7 impact for individuals 5-17 years. 99](#_Toc174092550)

[Supplementary Figure 78. ST1, 5, 7F with PCV10 use and substantial PCV7 impact for individuals 5-17 years. 100](#_Toc174092551)

[Supplementary Figure 79. ST1, 5, 7F with PCV10 use and moderate PCV7 impact for individuals 5-17 years. 101](#_Toc174092552)

[Supplementary Figure 80. ST1, 5, 7F with PCV10 use and no PCV7 impact for individuals 5-17 years. 102](#_Toc174092553)

[Supplementary Figure 81. ST1, 5, 7F with PCV13 use and substantial PCV7 impact for adults >18 years. 103](#_Toc174092554)

[Supplementary Figure 82. ST1, 5, 7F with PCV13 use and moderate PCV7 impact for adults >18 years. 104](#_Toc174092555)

[Supplementary Figure 83. ST1, 5, 7F with PCV10 use and substantial PCV7 impact for adults >18 years. 105](#_Toc174092556)

[Supplementary Figure 84. ST1, 5, 7F with PCV10 use and no PCV7 impact for adults >18 years. 106](#_Toc174092557)

[Supplementary Figure 85. Serotype 6A with PCV13 use and substantial PCV7 impact for children <5 years. 107](#_Toc174092558)

[Supplementary Figure 86. Serotype 6A with PCV13 use and moderate PCV7 impact for children <5 years. 108](#_Toc174092559)

[Supplementary Figure 87. Serotype 6A with PCV13 use and no PCV7 impact for children <5 years. 109](#_Toc174092560)

[Supplementary Figure 88. Serotype 6A with PCV10 use and substantial PCV7 impact for children <5 years. 110](#_Toc174092561)

[Supplementary Figure 89. Serotype 6A with PCV10 use and moderate PCV7 impact for children <5 years. 111](#_Toc174092562)

[Supplementary Figure 90. Serotype 6A with PCV10 use and no PCV7 impact for children <5 years. 112](#_Toc174092563)

[Supplementary Figure 91. Serotype 6A with PCV13 use and substantial PCV7 impact for individuals 5-17 years. 113](#_Toc174092564)

[Supplementary Figure 92. Serotype 6A with PCV13 use and moderate PCV7 impact for individuals 5-17 years. 114](#_Toc174092565)

[Supplementary Figure 93. Serotype 6A with PCV13 use and no PCV7 impact for individuals 5-17 years. 115](#_Toc174092566)

[Supplementary Figure 94. Serotype 6A with PCV10 use and moderate PCV7 impact for individuals 5-17 years. 116](#_Toc174092567)

[Supplementary Figure 95. Serotype 6A with PCV10 use and no PCV7 impact for individuals 5-17 years. 117](#_Toc174092568)

[Supplementary Figure 96. Serotype 6A with PCV13 use and substantial PCV7 impact for adults >18 years. 118](#_Toc174092569)

[Supplementary Figure 97. Serotype 6A with PCV13 use and moderate PCV7 impact for adults >18 years. 119](#_Toc174092570)

[Supplementary Figure 98. Serotype 6A with PCV10 use and substantial PCV7 impact for adults >18 years. 120](#_Toc174092571)

[Supplementary Figure 99. Serotype 6A with PCV10 use and no PCV7 impact for adults >18 years. 121](#_Toc174092572)

[Supplementary Figure 100. Serotype 19A with PCV13 use and substantial PCV7 impact for children <5 years. 122](#_Toc174092573)

[Supplementary Figure 101. Serotype 19A with PCV13 use and moderate PCV7 impact for children <5 years. 123](#_Toc174092574)

[Supplementary Figure 102. Serotype 19A with PCV13 use and no PCV7 impact for children <5 years. 124](#_Toc174092575)

[Supplementary Figure 103. Serotype 19A with PCV10 use and substantial PCV7 impact for children <5 years. 125](#_Toc174092576)

[Supplementary Figure 104. Serotype 19A with PCV10 use and moderate PCV7 impact for children <5 years. 126](#_Toc174092577)

[Supplementary Figure 105. Serotype 19A with PCV10 use and no PCV7 impact for children <5 years. 127](#_Toc174092578)

[Supplementary Figure 106. Serotype 19A with PCV13 use and substantial PCV7 impact for individuals 5-17 years. 128](#_Toc174092579)

[Supplementary Figure 107. Serotype 19A with PCV13 use and moderate PCV7 impact for individuals 5-17 years. 129](#_Toc174092580)

[Supplementary Figure 108. Serotype 19A with PCV13 use and no PCV7 impact for individuals 5-17 years. 130](#_Toc174092581)

[Supplementary Figure 109. Serotype 19A with PCV10 use and substantial PCV7 impact for individuals 5-17 years. 131](#_Toc174092582)

[Supplementary Figure 110. Serotype 19A with PCV10 use and moderate PCV7 impact for individuals 5-17 years. 132](#_Toc174092583)

[Supplementary Figure 111. Serotype 19A with PCV10 use and no PCV7 impact for individuals 5-17 years. 133](#_Toc174092584)

[Supplementary Figure 112. Serotype 19A with PCV13 use and substantial PCV7 impact for adults >18 years. 134](#_Toc174092585)

[Supplementary Figure 113. Serotype 19A with PCV13 use and moderate PCV7 impact for adults >18 years. 135](#_Toc174092586)

[Supplementary Figure 114. Serotype 19A with PCV10 use and substantial PCV7 impact for adults >18 years. 136](#_Toc174092587)

[Supplementary Figure 115. Serotype 19A with PCV10 use and no PCV7 impact for adults >18 years. 137](#_Toc174092588)

[Supplementary Figure 116. Serotype 3 with PCV13 use and substantial PCV7 impact for children <5 years. 138](#_Toc174092589)

[Supplementary Figure 117. Serotype 3 with PCV13 use and moderate PCV7 impact for children <5 years. 139](#_Toc174092590)

[Supplementary Figure 118. Serotype 3 with PCV13 use and no PCV7 impact for children <5 years. 140](#_Toc174092591)

[Supplementary Figure 119. Serotype 3 with PCV10 use and substantial PCV7 impact for children <5 years. 141](#_Toc174092592)

[Supplementary Figure 120. Serotype 3 with PCV10 use and moderate PCV7 impact for children <5 years. 142](#_Toc174092593)

[Supplementary Figure 121. Serotype 3 with PCV10 use and no PCV7 impact for children <5 years. 143](#_Toc174092594)

[Supplementary Figure 122. Serotype 3 with PCV13 use and substantial PCV7 impact for individuals 5-17 years. 144](#_Toc174092595)

[Supplementary Figure 123. Serotype 3 with PCV13 use and moderate PCV7 impact for individuals 5-17 years. 145](#_Toc174092596)

[Supplementary Figure 124. Serotype 3 with PCV13 use and no PCV7 impact for individuals 5-17 years. 146](#_Toc174092597)

[Supplementary Figure 125. Serotype 3 with PCV10 use and substantial PCV7 impact for individuals 5-17 years. 147](#_Toc174092598)

[Supplementary Figure 126. Serotype 3 with PCV10 use and moderate PCV7 impact for individuals 5-17 years. 148](#_Toc174092599)

[Supplementary Figure 127. Serotype 3 with PCV10 use and no PCV7 impact for individuals 5-17 years. 149](#_Toc174092600)

[Supplementary Figure 128. Serotype 3 with PCV13 use and substantial PCV7 impact for adults >18 years. 150](#_Toc174092601)

[Supplementary Figure 129. Serotype 3 with PCV13 use and moderate PCV7 impact for adults >18 years. 151](#_Toc174092602)

[Supplementary Figure 130. Serotype 3 with PCV10 use and substantial PCV7 impact for adults >18 years. 152](#_Toc174092603)

[Supplementary Figure 131. Serotype 3 with PCV10 use and no PCV7 impact for adults >18 years. 153](#_Toc174092604)

[Supplementary Figure 132. Non-PCV13 serotype with PCV13 use and substantial PCV7 impact for children <5 years. 154](#_Toc174092605)

[Supplementary Figure 133. Non-PCV13 serotype with PCV13 use and moderate PCV7 impact for children <5 years. 155](#_Toc174092606)

[Supplementary Figure 134. Non-PCV13 serotype with PCV13 use and no PCV7 impact for children <5 years. 156](#_Toc174092607)

[Supplementary Figure 135. Non-PCV13 serotype with PCV10 use and substantial PCV7 impact for children <5 years. 157](#_Toc174092608)

[Supplementary Figure 136. Non-PCV13 serotype with PCV10 use and moderate PCV7 impact for children <5 years. 158](#_Toc174092609)

[Supplementary Figure 137. Non-PCV13 serotype with PCV10 use and no PCV7 impact for children <5 years. 159](#_Toc174092610)

[Supplementary Figure 138. Non-PCV13 serotype with PCV13 use and substantial PCV7 impact for individuals 5-17 years. 160](#_Toc174092611)

[Supplementary Figure 139. Non-PCV13 serotype with PCV13 use and moderate PCV7 impact for individuals 5-17 years. 161](#_Toc174092612)

[Supplementary Figure 140. Non-PCV13 serotype with PCV13 use and no PCV7 impact for individuals 5-17 years. 162](#_Toc174092613)

[Supplementary Figure 141. Non-PCV13 serotype with PCV10 use and substantial PCV7 impact for individuals 5-17 years. 163](#_Toc174092614)

[Supplementary Figure 142. Non-PCV13 serotype with PCV10 use and moderate PCV7 impact for individuals 5-17 years. 164](#_Toc174092615)

[Supplementary Figure 143. Non-PCV13 serotype with PCV10 use and no PCV7 impact for individuals 5-17 years. 165](#_Toc174092616)

[Supplementary Figure 144. Non-PCV13 serotype with PCV13 use and substantial PCV7 impact for adults >18 years. 166](#_Toc174092617)

[Supplementary Figure 145. Non-PCV13 serotype with PCV13 use and moderate PCV7 impact for adults >18 years. 167](#_Toc174092618)

[Supplementary Figure 146. Non-PCV13 serotype with PCV10 use and substantial PCV7 impact for adults >18 years. 168](#_Toc174092619)

[Supplementary Figure 147. Non-PCV13 serotype with PCV10 use and no PCV7 impact for adults >18 years. 169](#_Toc174092620)

[Supplementary Figure 148. PCV10-type with PCV13 use and substantial PCV7 impact for children <5 years. 170](#_Toc174092621)

[Supplementary Figure 149. PCV10-type with PCV13 use and moderate PCV7 impact for children <5 years. 171](#_Toc174092622)

[Supplementary Figure 150. PCV10-type with PCV13 use and no PCV7 impact for children <5 years. 172](#_Toc174092623)

[Supplementary Figure 151. PCV10-type with PCV10 use and substantial PCV7 impact for children <5 years. 173](#_Toc174092624)

[Supplementary Figure 152. PCV10-type with PCV10 use and moderate PCV7 impact for children <5 years. 174](#_Toc174092625)

[Supplementary Figure 153. PCV10-type with PCV10 use and no PCV7 impact for children <5 years. 175](#_Toc174092626)

[Supplementary Figure 154. PCV13-type with PCV13 use and substantial PCV7 impact for children <5 years. 176](#_Toc174092627)

[Supplementary Figure 155. PCV13-type with PCV13 use and moderate PCV7 impact for children <5 years. 177](#_Toc174092628)

[Supplementary Figure 156. PCV13-type with PCV13 use and no PCV7 impact for children <5 years. 178](#_Toc174092629)

[Supplementary Figure 157. PCV13-type with PCV10 use and substantial PCV7 impact for children <5 years. 179](#_Toc174092630)

[Supplementary Figure 158. PCV13-type with PCV10 use and moderate PCV7 impact for children <5 years. 180](#_Toc174092631)

[Supplementary Figure 159. PCV13-type with PCV10 use and no PCV7 impact for children <5 years 181](#_Toc174092632)

# Supplementary Methods

*Adjustments for missing data*

Cases without a specific serotypes identified were grouped into four categories: “not serotyped”, “untypeable”, “typed, serotype not identified”, “serogrouped only”.

“Not serotyped” meningitis cases (where serotyping was not attempted for any reason) had population denominators adjusted by the proportion of all meningitis cases that were serotyped (i.e., annual denominator * percent of cases that were serotyped in that year) for each site-year-age group strata. For cases with unknown age, the population denominators were adjusted by the proportion of cases with known age (i.e., annual denominator * percent of cases with known age in that year) for each year and age group.

“Untypeable cases” (comprehensive serotyping methodology was performed but a serotype was not identified (e.g., non-encapsulated strain prohibiting serotyping or an isolate that produces less capsule under lab conditions so it cannot be serotyped)) were counted as NVTs and excluded from serotype-specific analyses.

“Typed, serotype not identified” (serotyping was performed with a method that does not assess all serotypes and a specific serotype was not identified) were counted as NVTs if the serotyping method tested for all VTs. For serotype-specific analyses or if the serotyping method did not include all VTs, the case was treated as a “not serotyped” case as described above. Cases reported as Quellung Pool-only were counted as VT or NVT, if possible, and otherwise treated as a “not serotyped” case as described above and excluded from serotype-specific analyses.

Cases with two serotypes reported (two specimens taken from one case or two serotypes identified from one specimen), were counted as VT or NVT if both were of the same category, but if one was VT and the other was NVT then treated as a “not serotyped” case as described above. Both were included in relevant serotype-specific analyses as applicable.

For “serogrouped-only” and undistinguished cases, cases were redistributed according to the distribution of fully serotyped cases within the serogroup by site, age group, and year first. If the data did not adequately represent the serotype distribution (i.e., >50% of cases undistinguished or fewer than 10 distinguished cases in the site, age group, year stratum), redistribution was done within the geographic region and time period relative to PCV introduction, then within the time period relative to PCV introduction using the pooled global serotype distribution. The established serotype distribution was applied to serogrouped and undistinguished meningitis and non-meningitis cases.

*Statistical Analysis*

*Step 1*: First, IR curves were estimated over years of available data for each site using a Bayesian multi-level, mixed-effects Poisson regression using the MCMCglmm package in R for PCV10 and PCV13-using sites separately ^1^. The model included data from all PCV10- or PCV13-using sites with an offset for population denominator and random effects for all of the site-specific regression coefficients, which allows for heterogeneity among sites in the shapes of their incidence curves. The regression identified commonalities within and across sites in the direction of change over time and smoothed out observed annual variability. Data points from the same site were treated as repeated measures over time and sites with small case counts or few years of data had less influence on the estimated average curve than sites with larger case counts and many years of data.

The regression modeled the pre-PCV (pre-any PCV, including PCV7 if used) IRs with a linear slope across the pre-PCV period. For each site, a non-linear break (allowing an abrupt hinge in the curve) was included in the model one year prior to PCV7 (if used) and one year prior to PCV10/13 introduction to capture the change from the year prior to introduction to the year of PCV introduction. Cubic splines knots (allowing a smooth change in the slope) were included for each site at years +1 and +3 post PCV7 (if used) and PCV10/13 (the second and fourth year of PCV use) to allow for flexibility in the IR over time for each site following PCV introduction. Site-specific modeled IR curves were visually inspected for the reasonableness of model fit and approved by site investigators with expertise in invasive pneumococcal disease (IPD) surveillance at each site.

*Step 2*: Second, a counterfactual IR (i.e., an expected IR in any given post-PCV year in the absence of PCV introduction) was estimated for sites with both pre- and post-PCV data by extending the pre-PCV IR slope for three years post-PCV10/13 introduction before flattening to a slope of zero at the achieved level. The site-specific modeled IR and counterfactual IR were used to estimate site-specific annual IRRs in each post-PCV year (reported as the mean of the posterior distribution of rate ratios) for each site. Site-specific IRRs were not generated for sites without pre-PCV years of data. Credibility intervals (CIs, Bayesian confidence interval analog) were estimated using the 2.5 and 97.5 percentiles of the posterior distribution of the IRs.

*Step 3*: Third, modeled site-specific IRRs were used to estimate all-site weighted average IRRs in each post-PCV year using a linear mixed-effects regression where site-specific IRRs were regressed on a three-way interaction term for year since PCV10/13 introduction, prior PCV7 impact, and product (PCV10 or PCV13) with a random intercept for site to account for approximately exchangeable correlation among repeated years for a given site. The model was weighted to give more influence to sites whose IRR standard errors were smaller. Prior PCV7 impact strata were defined by the modeled site-specific reduction in PCV7-serotype IPD among children <5 years in the last year of PCV7 use (i.e., the year prior to PCV10/13 introduction) (no PCV7 impact (PCV7 not used), moderate (PCV7-serotype IRR for children <5 years >0.05 in the last year of PCV7 use, or substantial (IRR ≤0.05).

*Comparison between PCV10/13 impact on IPD and CSF*

The results for IPD in this analysis differs from previously published PSERENADE analyses that evaluated the global impact of PCV10/13 on IPD^2^. Specifically, this analysis was limited to sites that provided both IPD and meningitis data, excluding sites that only provided IPD data or only meningitis data. This approach allowed us to compare the differences in the impact of PCV10/13 on IPD and cerebrospinal fluid (CSF) infections across all serotypes, vaccine-type/non-vaccine-type (VT/NVT) categories, and specific serotypes, stratified by prior PCV7 impact categories.

Reference:

1. Hadfield, J. D. MCMC Methods for Multi-Response Generalized Linear Mixed Models: The MCMCglmm R Package. *Journal of Statistical Software* **33**, 1–22 (2010).

2. Bennett, J. *et al.* Global impact of 10- and 13-valent pneumococcal conjugate vaccines on invasive pneumococcal disease in all ages: the PSERENADE project.

# Supplementary Table 1. Sites in PSERENADE evaluated for inclusion in the CSF+ meningitis incidence analyses by age group

Y= Yes, included; N= No, excluded. The table lists all sites initially considered for inclusion in the analysis. Some sites were later excluded after applying data eligibility criteria or having insufficient data.

| Site | <5 years | | 5-17 years | | ≥18 years | |
| --- | --- | --- | --- | --- | --- | --- |
|  | All-ST | VT/NVT | All-ST | VT/NVT | All-ST | VT/NVT |
| Australia (Non-Indigenous) | Y | Y | Y | Y^1^ | Y | Y |
| Northern Territory, Australia | Y | Y^1,2,3^ | Y | Y^1,3^ | Y | Y^1,2,3,5^ |
| Mirzapur, Bangladesh | N^4^ | N^4^ | N^4^ | N^4^ | -- | -- |
| Belgium | Y | Y | Y | Y^3^ | -- | -- |
| Brazil | Y | Y | Y | Y | Y | Y |
| Alberta, Canada | Y | Y^5^ | Y | Y^1,5^ | Y | Y |
| Ontario, Canada | Y | N | Y | N | Y | N |
| Quebec (excluding Nunavik), Canada (PCV13) | Y | Y^1^ | Y | Y^1,5^ | Y | Y^1^ |
| Quebec (excluding Nunavik), Canada (PCV10) | Y | Y | Y | N^7^ | Y | Y^1^ |
| Quebec-Nunavik, Canada (PCV13) | Y | Y | Y | N^7^ | Y | N^7^ |
| Quebec-Nunavik, Canada (PCV10) | Y | Y^1,2,5,6^ | Y | N^7^ | Y | N^7^ |
| Metropolitan Region, Chile | Y | Y | Y | Y | Y | Y |
| Non-Metropolitan Regions, Chile | Y | Y^1,5^ | Y | Y^1^ | Y | Y |
| Czech Republic | N^8^ | N^8^ | N^8^ | N^8^ | N^8^ | N^8^ |
| Denmark | Y | Y | Y | Y | Y | Y |
| Fiji | Y | N^7^ | N^4^ | N^4^ | N^4^ | N^4^ |
| Finland | Y | Y | Y | Y^1,5^ | Y | Y |
| France | Y | N | Y | N | Y | N |
| Basse, The Gambia | Y | N | N^4^ | N^4^ | N^4^ | N^4^ |
| Germany | Y | Y | Y | Y | Y | Y |
| Greece | N^9^ | N^9^ | N^9^ | N^9^ | N^9^ | N^9^ |
| Hong Kong | Y | Y^1,2,3,5^ | Y | Y^1,3,5,6^ | Y | Y^1.5^ |
| Iceland | Y | Y^1,2^ | Y | Y^1,2,3^ | Y | Y |
| Ireland | Y | Y^1,2^ | Y | Y^1,3^ | Y | Y |
| Israel | Y | Y | Y | Y | Y | Y |
| Italy | Y | Y | Y | Y^1^ | Y | Y |
| Japan | N^4^ | N^4^ | N^4^ | N^4^ | Y | Y^1,3,5^ |
| Asembo, Kenya | N^10^ | N^10^ | N^10^ | N^10^ | N^10^ | N^10^ |
| Kibera, Kenya | N^10^ | N^10^ | N^10^ | N^10^ | N^10^ | N^10^ |
| Kilifi, Kenya | Y | Y^3^ | Y | Y^3^ | Y | Y^1,2^ |
| Latvia | N^4^ | N^4^ | N^4^ | N^4^ | Y | Y |
| Blantyre District, Malawi | N^4^ | N^4^ | N^4^ | N^4^ | N^4^ | N^4^ |
| Mongolia | Y | N^5^ | -- | -- | -- | -- |
| Morocco | Y | Y | Y | Y^2,3^ | Y | N^7^ |
| Netherlands | Y | Y | Y | Y^1^ | Y | Y |
| New Zealand | N^4^ | N^4^ | N^4^ | N^4^ | Y | Y |
| Norway | Y | Y^2^ | Y | Y^3^ | Y | Y |
| Poland | N^8^ | N^8^ | N^8^ | N^8^ | N^8^ | N^8^ |
| Singapore | Y | Y^1,2,3,5^ | Y | N^7^ | Y | Y^3^ |
| Slovakia | N^8^ | N^8^ | N^8^ | N^8^ | N^8^ | N^8^ |
| Slovenia | N^4^ | N^4^ | N^4^ | N^4^ | N^4^ | N^4^ |
| South Africa | Y | Y | Y | Y | Y | Y |
| Catalonia, Spain | Y | Y | Y | Y | Y | Y |
| Madrid, Spain | Y | Y | Y | Y^2,5^ | Y | Y |
| Navarra, Spain | Y | Y^1,5^ | Y | Y^2,3^ | Y | Y^1^ |
| Sweden | N^8^ | N^8^ | N^8^ | N^8^ | N^8^ | N^8^ |
| Switzerland | N^4^ | N^4^ | N^4^ | N^4^ | Y | Y |
| England & Wales, UK | Y | Y | Y | Y | Y | Y |
| Scotland, UK | Y | Y^2,3,5^ | Y | Y^1,3,5,6^ | Y | Y^1,5,6^ |
| Active Bacterial Core surveillance (ABCs), USA | Y | Y | Y | Y | Y | Y |
| Alaska, USA | Y | Y^2^ | Y | Y | Y | Y |
| California, USA | N^4^ | N^4^ | N^4^ | N^4^ | N^4^ | N^4^ |
| Massachusetts, USA | Y | N | -- | -- | -- | -- |
| Southwest, USA (Indigenous) | Y | Y^1^ | Y | Y^2,3^ | Y | Y |
| Utah, USA | Y | N | -- | -- | -- | -- |

^1^ Zero ST6A cases in all years

^2^ Zero ST3 cases in all years

^3^ Zero ST19A cases in all years

^4^ Temporal changes in CSF+ meningitis surveillance system over time or other non-PCV changes that could not be accounted for in analyses

^5^ Zero ST1, 5, & 7F cases in all years

^6^ Zero PCV7 cases in all years

^7^ Low proportion of cases serotyped (<50%)

^8^ Concurrent PCV10/13 use

^9^ Population-based surveillance data only available for clinically-defined pneumococcal meningitis cases

^10^ Population-based surveillance data only available for site-defined IPD cases

-- Data not provided or not available

Supplementary Table 2. Description of surveillance data included in analyses by PCV product and PCV7-impact strata

|  | Total Number (Median, Range across sites) | | | | |
| --- | --- | --- | --- | --- | --- |
|  | Sites with both pre- and post-PCV data^1^ | | | Sites with post-PCV data only^2^ | Total |
|  | No PCV7 Impact | Moderate PCV7 Impact | Substantial PCV7 Impact^3^ |  |  |
| PCV10 Sites | | | | | |
| *<5 years* | | | | | |
| Surveillance Sites | 4 | 2 | 4 | 2 | 12 |
| Countries | 4 | 2 | 3 | 2 | 10 |
| Cases | 2346  (62.5, 17-2204) | 198  (99.0, 76-122) | 256  (67.0, 5-117) | 49  (24.5, 12-37) | 2849  (62.5, 5-2204) |
| Surveillance Years | 74  (18.0, 14-24) | 26  (13.0, 12-14) | 55  (13.0, 11-18) | 9  (4.5, 3-6) | 164  (14.0, 3-24) |
| *5-17 years* | | | | | |
| Surveillance Sites | 4 | 1 | 4 | 1 | 10 |
| Countries | 4 | 1 | 3 | 1 | 8 |
| Cases | 1412  (21.0, 5-1365) | 32  (32.0, 32-32) | 70  (17.0, 2-34) | 35  (35.0, 35-35) | 1549  (29.5, 2-1365) |
| Surveillance Years | 74  (18.0, 14-24) | 14  (14.0, 14-14) | 55  (13.0, 11-18) | 6  (6.0, 6-6) | 149  (14.5, 6-24) |
| *≥18 years* | | | | | |
| Surveillance Sites | 4 | 1 | 4 | 3 | 13 |
| Countries | 4 | 1 | 3 | 3 | 11 |
| Cases | 4181  (177.5, 14-3812) | 228  (228.0, 228-228) | 805 (114.0, 1-576) | 429 (101.0, 72-256) | 5653 (101.0, 1-3812) |
| Surveillance Years | 67 (16, 10-24) | 14 (14, 14-14) | 55 (13, 11-18) | 18 (6, 5-7) | 161 (11, 5-24) |
| PCV13 Sites | | | | | |
| *<5 years* | | | | | |
| Surveillance Sites | 2 | 6 | 11 | 10 | 30 |
| Countries | 2 | 6 | 6 | 8 | 20 |
| Cases | 54  (27.0, 14-40) | 6531  (517.0, 81-3771) | 2873  (55.0, 5-1630) | 706  (50.0, 5-184) | 10168  (69.5, 4-3771) |
| Surveillance Years | 10  (5.0, 5-5) | 106  (15.0, 11-19) | 200  (18.0, 12-27) | 100  (10.0, 5-16) | 416  (14.0, 5-27) |
| *5-17 years* | | | | | |
| Surveillance Sites | 1 | 5 | 11 | 7 | 25 |
| Countries | 1 | 5 | 6 | 6 | 17 |
| Cases | 7  (7.0, 7-7) | 2834  (220.0, 59-2058) | 688  (12.0, 2-296) | 181  (26.0, 1-55) | 3711  (28.0, 1-2058) |
| Surveillance Years | 5  (5.0, 5-5) | 80  (16.0, 14-19) | 195  (18.0, 10-27) | 68  (10.0, 5-12) | 359  (14.0, 5-27) |
| *≥18 years* | | | | | |
| Surveillance Sites | 1 | 4 | 10 | 10 | 26 |
| Countries | 1 | 4 | 6 | 9 | 18 |
| Cases | 54  (54, 54-54) | 18456  (3166.0, 343-11781) | 6634  (148.0, 1-2959) | 4021  (200.0, 32-1359) | 29187  (211.5, 1-11781) |
| Surveillance Years | 5  (5, 5-5) | 65  (16.0, 14-19) | 177  (18.0, 10-27) | 86  (9.0, 5-12) | 344  (12.0, 5-27) |

^1^ Data included in analysis steps 1-3 (site-specific modeling of IR and IRR curves and estimation of all-site weighted average IRRs)

^2^ Data included only in analysis step 1 (site-specific modeling of post-PCV IR curves)

^3^ Pre-PCV and PCV7 years of data from Quebec sites included in both PCV10 and PCV13 models

# Supplementary Table 3. Description of surveillance data included in CSF+ meningitis analyses by site

| Site |  | | | | | | | | | No. of surveillance years (Pre, PCV7, PCV10/13) | | | | | | | | | No. of CSF+ cases | | |  |
| --- | --- | --- | --- | --- | --- | --- | --- | --- | --- | --- | --- | --- | --- | --- | --- | --- | --- | --- | --- | --- | --- | --- |
|  | PCV Product^1^ | PCV7-Impact Strata^2^ | PCV10/13 Schedule^1^ | PCV  10/13 catch-up | Mean PCV 10/13 Uptake^3^ | Proportion VT in pre- Period^4^ (%) | | Cases serotyped (n) | Proportion of cases serotyped^5^ (%) |  |  |  |  |  |  |  |  |  |  |  |  |  |
|  |  |  |  |  |  |  |  |  |  | <5y | | | 5-17y | | | ≥18y | | | <5y | 5-17y | ≥18y | Definition for meningitis cases without pneumococcus detected in CSF^6^ |
| Kilifi, Kenya | PCV10 | No impact | 3+0 | Y | 82 | | 71.9 | 111 | 100 | 11, | 0, | 6 | 11, | 0, | 6 | 4, | 0, | 6 | 67 | 29 | 14 |  |
| Finland | PCV10 | No impact | 2+1 | N | 95 | | 84.4 | 367 | 95.7 | 6, | 0, | 8 | 6, | 0, | 8 | 6, | 0, | 8 | 58 | 13 | 313 | Spn in blood & diagnosis from ICD-10 codes |
| Iceland | PCV10 | No impact | 2+1 | N | 89* | | 86.7 | 48 | 74.5 | 16, | 0, | 8 | 16, | 0, | 8 | 16, | 0, | 8 | 17 | 5 | 42 | Spn in blood & abnormal CSF |
| Brazil | PCV10 | No impact | 3+1/2+1 | Y | 84 | | 76.5 | 7063 | 95.7 | 10, | 0, | 9 | 10, | 0, | 9 | 10, | 0, | 9 | 2204 | 1365 | 3812 | Spn in blood & (abnormal CSF or diagnosis from ICD-10 codes) |
| Metropolitan Region, Chile | PCV10 | Moderate | 2+1 | N | 97 | | 66.7 | 269 | 80.3 | 7, | 2, | 5 | 7, | 2, | 5 | 7, | 2, | 5 | 76 | 32 | 228 |  |
| New Zealand | PCV10 | Moderate | 3+1 | N | 92 | | 90.3 | 143 | 74.2 | 6, | 3, | 3 | 0, | 0, | 0, | 0, | 2, | 3 | 122 | 0 | 72 | Spn in blood & (abnormal CSF or clinical diagnosis (not from ICD-10 codes)) |
| Quebec (excluding Nunavik), Canada | PCV10 | Substantial | 2+1 | N | 97 | | 80.1 | 192 | 52.5 | 5, | 4, | 2 | 5, | 4, | 2 | 5, | 4, | 2 | 112 | 34 | 220 | Spn in blood & clinical diagnosis |
| Northern Territory, Australia | PCV10 | Substantial | 3+1 | Y | 80 | | 64.7 | 24 | 70.5 | 7, | 9, | 2, | 7, | 9, | 2 | 7, | 9, | 2 | 22 | 4 | 8 | Spn in blood & abnormal CSF |
| Quebec-Nunavik, Canada | PCV10 | Substantial | 3+1 | N | 97 | | - | 8 | 100 | 2, | 7, | 2 | 2, | 7, | 2 | 2, | 7, | 2 | 5 | 2 | 1 | Spn in blood & clinical diagnosis |
| Netherlands | PCV10 | Substantial | 2+1/3+1 | N | 95 | | 76.9 | 717 | 99.2 | 2, | 5, | 8 | 2, | 5, | 8 | 2, | 5, | 8 | 117 | 30 | 576 | *Only CSF+ cases reported* |
| Fiji | PCV10 | -- | 3+0 | N | 88 | | - | - | - | 0, | 0, | 3 | 0, | 0, | 0 | 1, | 0, | 6 | 12 | 0 | 10 | Spn in blood & abnormal CSF |
| Non-Metropolitan Region, Chile | PCV10 | -- | 2+1 | N | 97 | | - | 252 | 76.8 | 0, | 0, | 6 | 0, | 0, | 6 | 0, | 0, | 6 | 37 | 35 | 256 | Spn in blood & clinical diagnosis |
| Latvia | PCV10 | -- | 2+1/3+1 | N | 90 | | - | 73 | 72.3 | 0, | 0, | 0 | 0, | 0, | 0 | 0, | 0, | 7 | 0 | 0 | 101 | *Only CSF+ cases reported* |
| Grand Casablanca, Morocco | PCV13 | No impact | 2+1 | N | 82 | | 91.2 | 54 | 53.8 | 4, | 0, | 1 | 4, | 0, | 1 | 4, | 0, | 1 | 40 | 7 | 54 | Spn in blood & abnormal CSF |
| Mongolia | PCV13 | No impact | 2+1 | Y | 92 | | - | - | - | 4, | 0, | 1 | 0, | 0, | 0 | 0, | 0, | 0 | 14 | 0 | 0 | Spn in blood & (abnormal CSF or clinical diagnosis (not from ICD-10 codes)) |
| Denmark | PCV13 | Moderate | 2+1 | N | 90 | | 90.7 | 1313 | 90.2 | 8, | 2, | 1 | 8, | 2, | 1 | 8, | 2, | 1 | 232 | 59 | 1165 | *Only CSF+ cases reported* |
| Ireland | PCV13 | -- | 2+1 | N | 90 | | - | 141 | 62.8 | 0, | 2, | 1 | 0, | 2, | 1 | 0, | 2, | 1 | 42 | 18 | 165 | Spn in blood & (abnormal CSF or clinical diagnosis (not from ICD-10 codes)) |
| Israel | PCV13 | Moderate | 2+1 | N | 95 | | 83.2 | 616 | 94.5 | 7, | 1, | 1 | 7, | 1, | 1 | 0, | 1, | 1 | 360 | 65 | 227 | Spn in blood & abnormal CSF |
| Singapore | PCV13 | -- | 2+1 | Y | 73 | | NA | 19 | 69.6 | 1, | 2, | 1 | 1, | 2, | 1 | 1, | 2, | 1 | 4 | 1 | 22 | *Only CSF+ cases reported* |
| South Africa | PCV13 | Moderate | 2+1 | Y | 67 | | 83.5 | 11023 | 62.6 | 4, | 2, | 1 | 4, | 2, | 1 | 4, | 2, | 1 | 3771 | 2058 | 11781 | Spn in blood & clinical diagnosis |
| Switzerland | PCV13 | Moderate | 2+1 | Y | 55 | | 78.3 | 352 | 83.1 | 3, | 5, | 1 | 0, | 0, | 0 | 3, | 5, | 1 | 81 | 0 | 343 | Spn in blood & (abnormal CSF or clinical diagnosis (not from ICD-10 codes)) |
| France | PCV13 | Moderate | 2+1/3+1 | N | 81 | | 90.9 | 5672 | 81.0 | 2, | 7, | 1 | 2, | 7, | 1 | 2, | 7, | 1 | 1413 | 423 | 5167 | Spn in blood & abnormal CSF |
| Germany | PCV13 | Moderate | 2+1/3+1 | N | 83* | | 83.9 | 1728 | 98.7 | 2, | 3, | 1 | 2, | 3, | 1 | 0, | 0, | 1 | 674 | 229 | 848 | Spn in blood & clinical diagnosis |
| Australia (Non-Indigenous) | PCV13 | Substantial | 3+0 | Y | 91 | | 89.7 | 782 | 77.8 | 3, | 6, | 1 | 3, | 6, | 1 | 3, | 6, | 1 | 306 | 85 | 614 | Spn in blood & abnormal CSF |
| England, UK | PCV13 | Substantial | 2+1 | N | 93 | | 88.6 | 3810 | 78.0 | 6, | 4, | 1 | 6, | 4, | 1 | 6, | 4, | 1 | 1630 | 296 | 2959 | Spn in blood & clinical diagnosis |
| Norway | PCV13 | Substantial | 2+1 | N | 92 | | 89.7 | 552 | 89.3 | 2, | 5, | 1 | 2, | 5, | 1 | 2, | 5, | 1 | 103 | 28 | 487 | Spn in blood & clinical diagnosis (not from ICD-10 codes) |
| Quebec (excluding Nunavik), Canada | PCV13 | Substantial | 2+1 | N | 97 | | - | 116 | 52.8 | 5, | 4, | 3 | 5, | 4, | 1 | 5, | 4, | 1 | 113 | 33 | 196 | Spn in blood & clinical diagnosis |
| Scotland, UK | PCV13 | Substantial | 2+1 | N | 97 | | 0.0 | 202 | 59.1 | 0, | 0, | 1 | 0, | 0, | 1 | 0, | 0, | 1 | 35 | 12 | 173 | Spn in blood & clinical diagnosis (not from ICD-10 codes) |
| ABCs, USA | PCV13 | Substantial | 3+1 | Y | 80 | | 92.1 | 2541 | 89.6 | 2, | 10, | 1 | 2, | 10, | 1 | 2, | 10, | 1 | 541 | 198 | 2097 | Spn in blood & clinical diagnosis (not from ICD-10 codes) |
| Alaska, USA | PCV13 | Substantial | 3+1 | Y | 75 | | 93.5 | 130 | 85.6 | 10, | 9, | 1 | 10, | 9, | 1 | 10, | 9, | 1 | 55 | 10 | 87 | Spn in blood & clinical diagnosis |
| Quebec-Nunavik, Canada | PCV13 | Substantial | 3+1 | N | 97 | | - | 8 | 100 | 2, | 7, | 3 | 2, | 7, | 1 | 2, | 7, | 1 | 5 | 2 | 1 | Spn in blood & clinical diagnosis |
| Southwest, USA (Indigenous) | PCV13 | Substantial | 3+1 | Y | 71 | | 75.0 | 81 | 91.1 | 5, | 10, | 1 | 5, | 10, | 1 | 5, | 10, | 1 | 44 | 11 | 34 | Spn in blood & clinical diagnosis |
| Alberta, Canada | PCV13 | Substantial | 2+1/3+1 | N | 89 | | 80.0 | 92 | 100 | 2, | 8, | 1 | 2, | 8, | 1 | 2, | 8, | 1 | 25 | 8 | 59 | Spn in blood & abnormal CSF |
| Navarra, Spain | PCV13 | Substantial | 2+1/3+1 | N | 54 | | 100.0 | 91 | 75.2 | 3, | 6, | 1 | 3, | 6, | 1 | 3, | 6, | 1 | 16 | 5 | 100 | Spn in blood & abnormal CSF |
| Basse, The Gambia | PCV13 | -- | 3+0 | N | 64 | | - | - | - | 0, | 2, | 1 | 0, | 0, | 0 | 0, | 0, | 0 | 18 | 0 | 0 |  |
| Belgium | PCV13 | -- | 2+1 | N | 80 | | - | 192 | 86.3 | 0, | 5, | 5 | 0, | 5, | 4 | 0, | 0, | 0 | 184 | 38 | 0 | Spn in blood & clinical diagnosis (not from ICD-10 codes) |
| Italy | PCV13 | -- | 2+1 | N | 84 | | - | 757 | 50.0 | 0, | 0, | 1 | 0, | 0, | 1 | 0, | 0, | 1 | 100 | 55 | 1359 | Spn in blood & clinical diagnosis (not from ICD-10 codes) |
| Hong Kong | PCV13 | -- | 3+1 | N | 98 | | - | 36 | 93.9 | 0, | 0, | 5 | 0, | 0, | 5 | 0, | 0, | 5 | 5 | 1 | 32 | Spn in blood & (abnormal CSF or diagnosis (not from ICD-10 codes)) |
| Japan | PCV13 | -- | 3+1 | N | 98* | | - | 104 | 93.5 | 0, | 0, | 0 | 0, | 0, | 0 | 0, | 0, | 1 | 0 | 0 | 111 | Spn in blood & clinical diagnosis (not from ICD-10 codes) |
| Massachusetts, USA | PCV13 | -- | 3+1 | Y | 89 | | - | - | - | 0, | 8, | 1 | 0, | 0, | 0 | 0, | 0, | 0 | 58 | 0 | 0 | *Only CSF+ cases reported* |
| Utah, USA | PCV13 | -- | 3+1 | Y | 88 | | - | - | - | 0, | 0, | 1 | 0, | 0, | 0 | 0, | 0, | 0 | 11 | 0 | 0 | Spn in blood & abnormal CSF |
| Catalonia, Spain | PCV13 | -- | 2+1/3+1 | N | 64 | | - | 670 | 79.4 | 0, | 4, | 1 | 0, | 4, | 1 | 0, | 4, | 1 | 166 | 36 | 643 | Spn in blood & abnormal CSF |
| Madrid, Spain | PCV13 | -- | 2+1/3+1 | N | 95 | | - | 361 | 72.4 | 0, | 3, | 1 | 0, | 3, | 1 | 0, | 3, | 1 | 108 | 26 | 365 | Spn in blood & abnormal CSF |
| Ontario, Canada | PCV13 | -- | 2+1/3+1 | Y | 72 | | - | - | - | 0, | 3, | 8 | 0, | 3, | 8 | 0, | 3, | 8 | 14 | 7 | 98 | Spn in blood & (abnormal CSF or diagnosis (not from ICD-10 codes)) |

PCV: Pneumococcal conjugate vaccines.

VT: Vaccine-serotype.

-: Data not provided, not available, or excluded from analyses.

1 PCV product and schedule used during years of data included in analyses. For sites that used PCV10/PCV13 sequentially, the first PCV10/13 product introduced was included in the analysis. Quebec sites are included in PCV10 models for first two years of PCV10 use and PCV13 models for years of data ≥5-7 years after switch from PCV10 to PCV13.

2 Prior PCV7 impact strata defined by modeled site-specific reduction in PCV7-serotype IPD among children <5 years in the last year of PCV7 use (i.e., the year prior to PCV10/13 introduction) for sites with pre-PCV VT data: no PCV7 impact (i.e., PCV7 not used), substantial (PCV7-type IRR ≤0.05), or moderate (all others with pre-PCV VT data). “--" indicates site had no pre-PCV IR data or inadequate serotyping (<50%) in the pre-PCV period for assessing the impact of PCV7 for categorizing PCV7-impact strata.

3 Annual PCV uptake estimates provided by the surveillance site for PCV10/13 years of data included in analyses. Uptake is for the primary series of PCV by 12 months of age (if available, for some sites up to 15 months of age), excluding the year of vaccine rollout. If unavailable, annual PCV uptake estimates provided by the surveillance site for the primary series plus the booster dose by 23 months of age, excluding the year of vaccine rollout used (N=7 sites: Alberta, Belgium, Denmark, Italy, South Africa, Switzerland, and Ontario). If PCV uptake data from the surveillance site unavailable, WHO and UNICEF Estimates of National Immunization Coverage (WUENIC) PCV uptake, excluding the year of vaccine rollout used (N=3 sites: Germany, Iceland, and Japan).

4 Proportion of serotyped cases due to VT CSF+ (PCV10 serotypes for PCV10 sites and PCV13 serotypes for PCV13 sites) among children <5 years in the pre-PCV period. “--" indicates site had no pre-PCV data.

5 Proportion of CSF+ meningitis cases fully serotyped across all age groups; cases not fully serotyped include undistinguished serotypes (e.g., ST6B/6C), Quellung pool and not further distinguished, etc. “--" indicates site only included in all serotype meningitis analyses (i.e., excluded from all VT/NVT analyses).

6 All sites defined pneumococcus detected from CSF as pneumococcal meningitis. Listed here are any additional definitions of pneumococcal meningitis. CSF was not systematically collected in all meningitis cases across all sites. The sterile site was assumed to be blood for 8 sites that did not further specify.

# Supplementary Table 4. All-site CSF+ meningitis weighted average incidence rate ratios comparing 6 years post-PCV10/13 incidence rate to the average pre-PCV incidence rate

Data are only provided for estimates that were available at 6 years post introduction; some groups had data that terminated earlier (e.g., PCV10 <5y with moderate PCV7 impact prior to PCV10 introduction).

|  |  |  | **<5 years of age** | | | **5-17 years of age** | | | **18+ years of age** | | |
| --- | --- | --- | --- | --- | --- | --- | --- | --- | --- | --- | --- |
| **Serotype** | Vaccine | PCV7 impact group | Number of Sites* | IRR | 95% CI | Number of Sites | IRR | 95% CI | Number of Sites | IRR | 95% CI |
| **All CSF** | PCV10 | None | 3 | 0.32 | (0.19, 0.54) | 3 | 0.41 | (0.37, 0.46) | 3 | 0.74 | (0.69, 0.80) |
|  | PCV10 | Substantial | 1 | 0.52 | (0.37, 0.73) | 1 | 0.82 | (0.45, 1.48) | 1 | 1.03 | (0.83, 1.29) |
|  | PCV13 | Moderate | 6 | 0.41 | (0.31, 0.53) | 6 | 0.63 | (0.40, 1.00) | 5 | 0.87 | (0.67, 1.13) |
|  | PCV13 | Substantial | 10 | 0.26 | (0.21, 0.32) | 11 | 0.69 | (0.49, 0.96) | 10 | 0.64 | (0.56, 0.73) |
| **PCV7** | PCV10 | None | 3 | 0.01 | (0.00, 0.02) | 3 | 0.22 | (0.14, 0.34) | 3 | 0.13 | (0.09, 0.18) |
|  | PCV10 | Substantial | 1 | 0.00 | (0.00, 0.00) | 1 | 0.04 | (0.02, 0.07) | 1 | 0.06 | (0.02, 0.13) |
|  | PCV13 | Moderate | 5 | 0.02 | (0.01, 0.04) | 4 | 0.09 | (0.06, 0.14) | 4 | 0.15 | (0.10, 0.24) |
|  | PCV13 | Substantial | 10 | 0.02 | (0.01, 0.03) | 8 | 0.06 | (0.04, 0.08) | 9 | 0.08 | (0.05, 0.13) |
| **1, 5, 7F** | PCV10 | None | 3 | 0.15 | (0.10, 0.23) | 2 | 0.06 | (0.02, 0.16) | 3 | 0.17 | (0.11, 0.26) |
|  | PCV10 | Substantial | 1 | 0.00 | (0.00, 0.00) | 1 | 1.35 | (0.46, 3.97) | 1 | 0.17 | (0.08, 0.37) |
|  | PCV13 | Moderate | 5 | 0.03 | (0.01, 0.05) | 4 | 0.05 | (0.03, 0.08) | 4 | 0.11 | (0.06, 0.21) |
|  | PCV13 | Substantial | 7 | 0.03 | (0.02, 0.06) | 5 | 0.01 | (0.01, 0.02) | 9 | 0.10 | (0.07, 0.14) |
| **6A** | PCV10 | None | 2 | 0.33 | (0.12, 0.87) | 1 | 0.04 | (0.03, 0.06) | 3 | 0.17 | (0.02, 1.79) |
|  | PCV10 | Substantial | 1 | 0.02 | (0.01, 0.05) |  |  |  | 1 | 0.05 | (0.01, 0.27) |
|  | PCV13 | Moderate | 5 | 0.03 | (0.02, 0.04) | 4 | 0.10 | (0.07, 0.16) | 4 | 0.08 | (0.05, 0.13) |
|  | PCV13 | Substantial | 7 | 0.00 | (0.00, 0.01) | 5 | 0.02 | (0.01, 0.06) | 6 | 0.04 | (0.02, 0.08) |
| **19A** | PCV10 | None | 3 | 3.16 | (1.85, 5.40) | 2 | 1.05 | (0.26, 4.36) | 3 | 5.92 | (3.84, 9.14) |
|  | PCV10 | Substantial | 1 | 18.11 | (7.71, 42.52) | 1 | 0.01 | (0.00, 0.03) | 1 | 5.31 | (2.90, 9.72) |
|  | PCV13 | Moderate | 5 | 0.26 | (0.14, 0.48) | 4 | 0.18 | (0.10, 0.32) | 4 | 0.41 | (0.31, 0.56) |
|  | PCV13 | Substantial | 10 | 0.32 | (0.20, 0.50) | 6 | 0.11 | (0.07, 0.18) | 9 | 1.07 | (0.81, 1.41) |
| **3** | PCV10 | None | 2 | 1.01 | (0.47, 2.19) | 2 | 1.20 | (0.59, 2.45) | 3 | 0.96 | (0.49, 1.86) |
|  | PCV10 | Substantial | 1 | 0.11 | (0.04, 0.27) | 1 | 0.53 | (0.26, 1.10) | 1 | 5.92 | (3.84, 9.13) |
|  | PCV13 | Moderate | 5 | 1.58 | (0.96, 2.58) | 4 | 0.58 | (0.31, 1.07) | 4 | 1.40 | (1.11, 1.78) |
|  | PCV13 | Substantial | 8 | 1.9 | (1.29, 2.80) | 7 | 0.44 | (0.28, 0.71) | 9 | 5.92 | (3.84, 9.13) |
|  | PCV13 | None |  |  |  | 1 | 0.59 | (0.17, 2.05) |  |  |  |
| **PCV10** | PCV10 | None | 3 | 0.01 | (0.01, 0.04) | 3 | 0.15 | (0.11, 0.21) | 3 | 0.11 | (0.06, 0.18) |
|  | PCV10 | Substantial | 1 | 0.00 | (0.00, 0.00) | 1 | 0.17 | (0.10, 0.28) | 1 | 0.09 | (0.05, 0.16) |
|  | PCV13 | Moderate | 5 | 0.03 | (0.02, 0.05) | 4 | 0.09 | (0.06, 0.14) | 4 | 0.14 | (0.11, 0.18) |
|  | PCV13 | Substantial | 10 | 0.02 | (0.01, 0.02) | 8 | 0.08 | (0.06, 0.10) | 9 | 0.07 | (0.05, 0.10) |
| **PCV13** | PCV10 | None | 3 | 0.13 | (0.09, 0.19) | 3 | 0.31 | (0.26, 0.37) | 3 | 0.35 | (0.24, 0.52) |
|  | PCV10 | Substantial | 1 | 0.14 | (0.08, 0.26) | 1 | 0.24 | (0.15, 0.37) | 1 | 0.41 | (0.33, 0.51) |
|  | PCV13 | Moderate | 5 | 0.04 | (0.03, 0.07) | 4 | 0.18 | (0.13, 0.24) | 4 | 0.27 | (0.21, 0.35) |
|  | PCV13 | Substantial | 11 | 0.04 | (0.03, 0.05) | 10 | 0.23 | (0.18, 0.28) | 9 | 0.23 | (0.19, 0.27) |
| **NVT13** | PCV10 | None | 3 | 1.58 | (0.88, 2.81) | 3 | 0.92 | (0.81, 1.05) | 3 | 1.04 | (0.91, 1.18) |
|  | PCV10 | Substantial | 1 | 3.18 | (2.19, 4.62) | 1 | 1.69 | (0.78, 3.64) | 1 | 1.64 | (1.15, 2.33) |
|  | PCV13 | Moderate | 5 | 2.22 | (1.63, 3.03) | 4 | 1.30 | (0.76, 2.21) | 4 | 1.92 | (1.26, 2.91) |
|  | PCV13 | Substantial | 11 | 1.74 | (1.41, 2.15) | 9 | 1.00 | (0.70, 1.42) | 9 | 1.24 | (1.02, 1.51) |

*The number of sites shown is the number with data out to year 6 (i.e., those with 7 or more years of PCV 10/13 use); more sites may have contributed to the projection (i.e., those with data <6 years still shape the trend line to arrive at these estimates).

# Supplementary Figure 1. Age distribution of CSF+ meningitis cases by PCV product, region, and age group


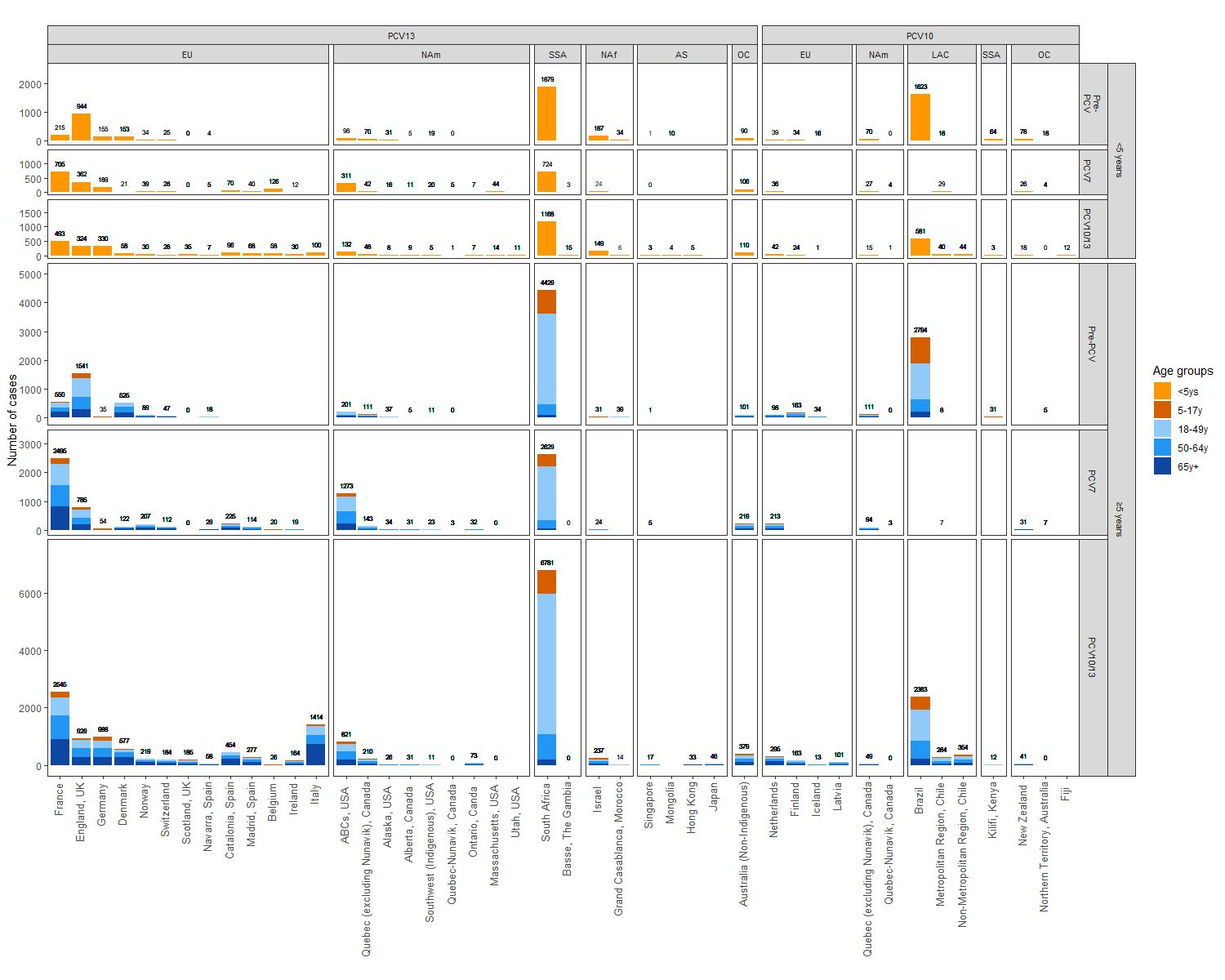


Supplementary Figure 2. Proportion of CSF+ vs. non-CSF+ clinical meningitis cases by site


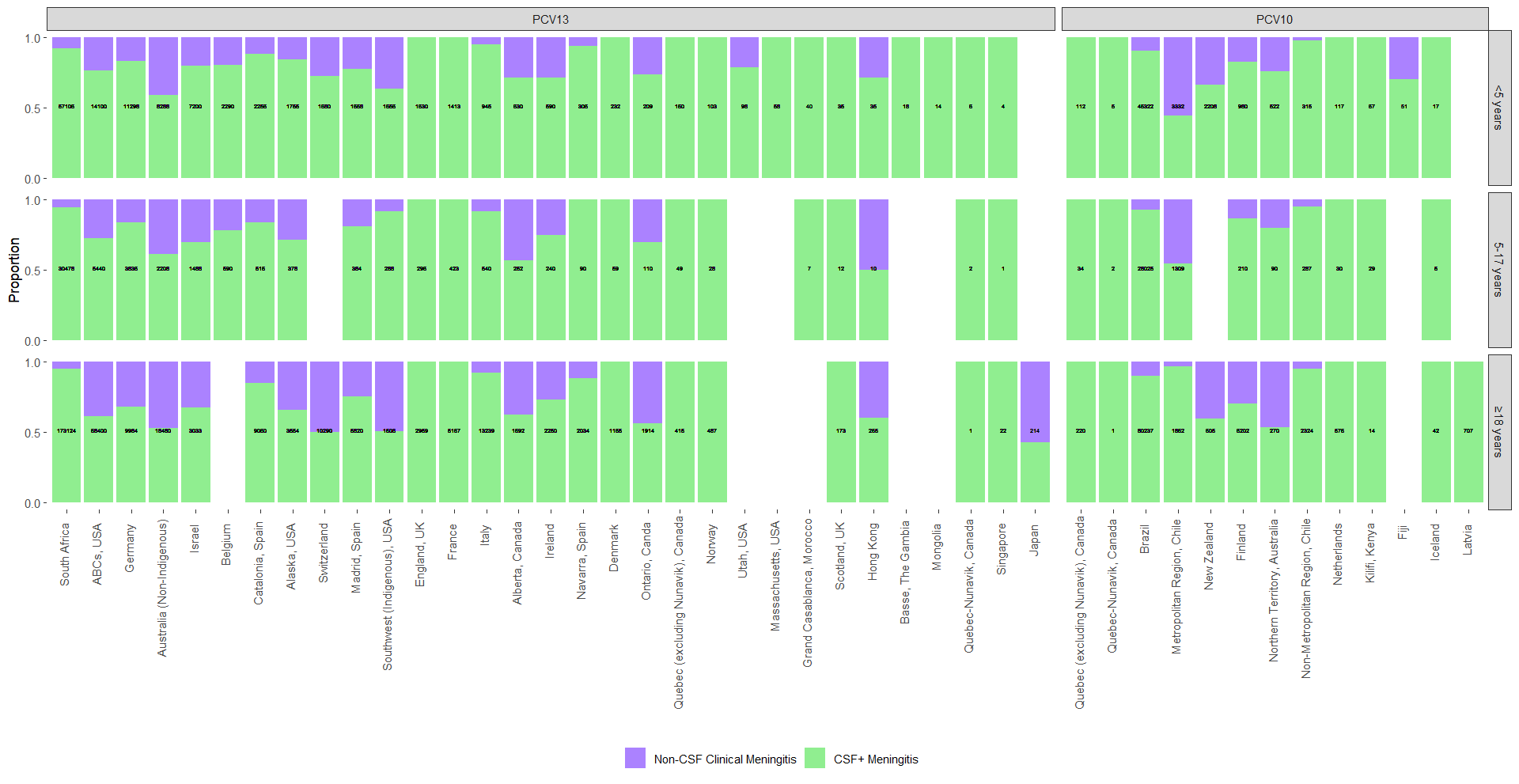


#

# Appendix 1: Clinically-defined meningitis all-site weighted average incidence rate ratios comparing the annual post-PCV10/13 incidence rate to the average pre-PCV incidence rate

Clinically-defined meningitis was defined as cases where the cerebrospinal fluid (CSF) test is positive, or when blood cultures are positive with the CSF being negative or not tested.

## Supplementary Figure 3. Children <5 years


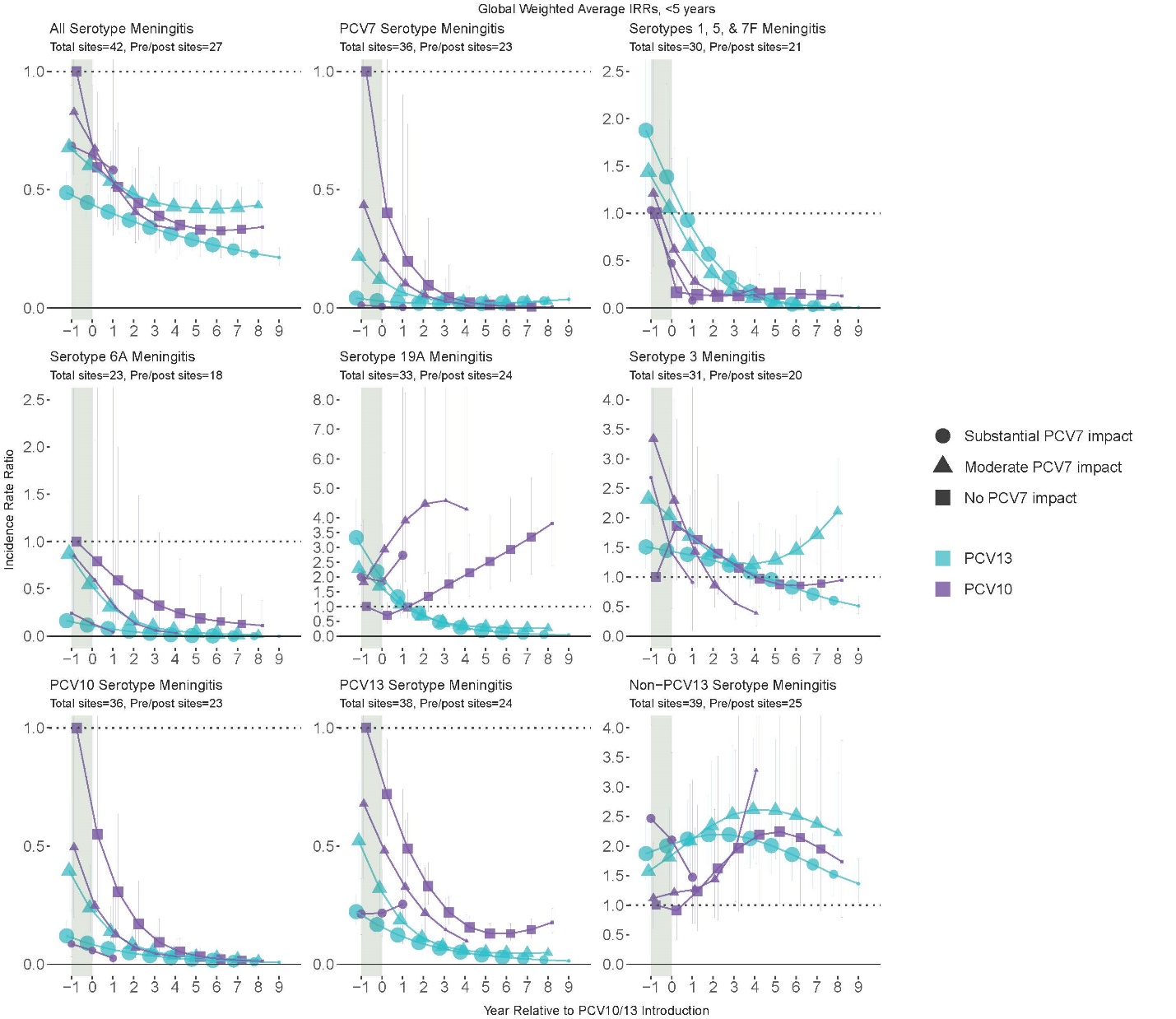


## Supplementary Figure 4. Children 5-17 years


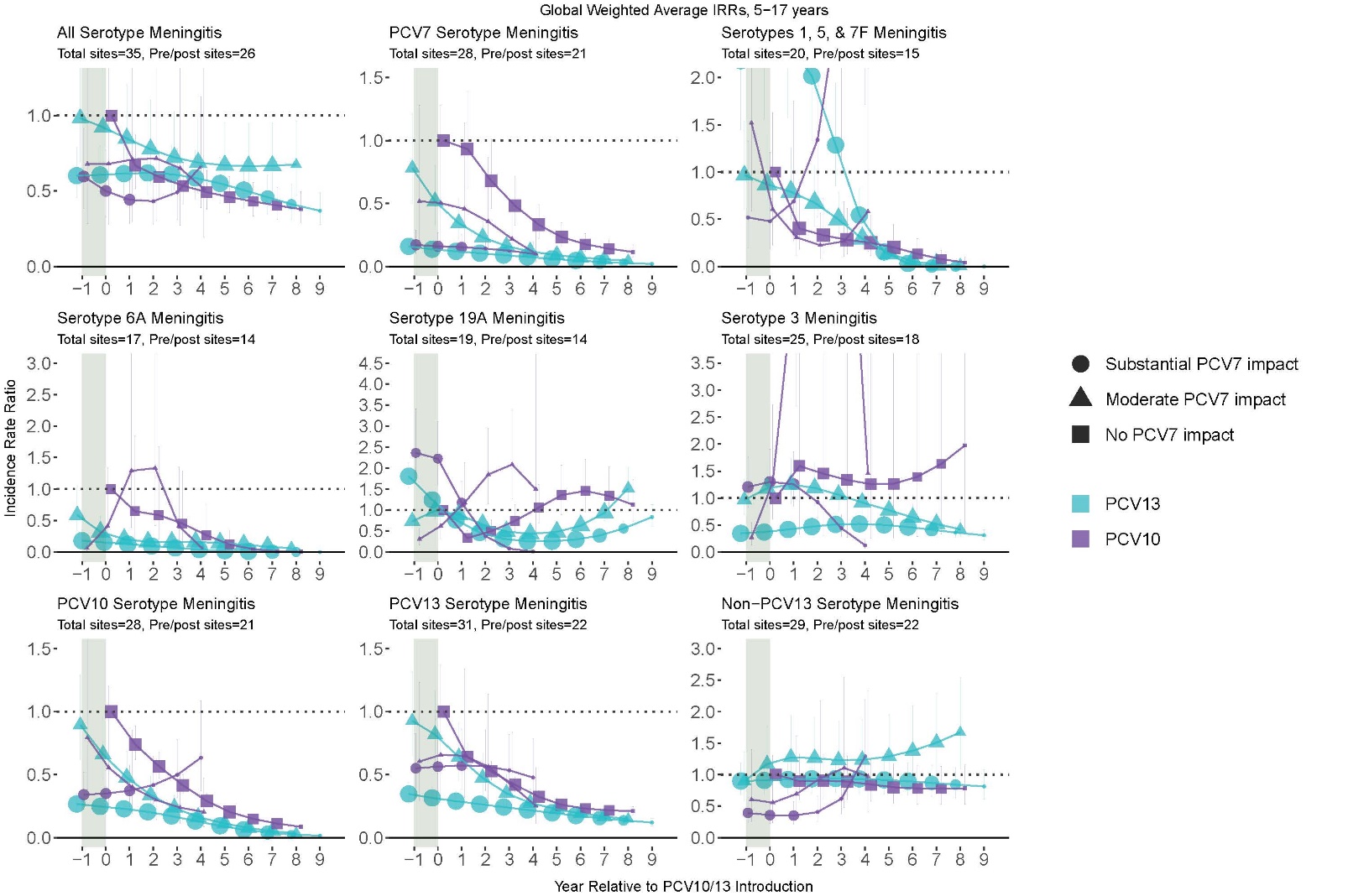


## Supplementary Figure 5. Adults 18 years and older


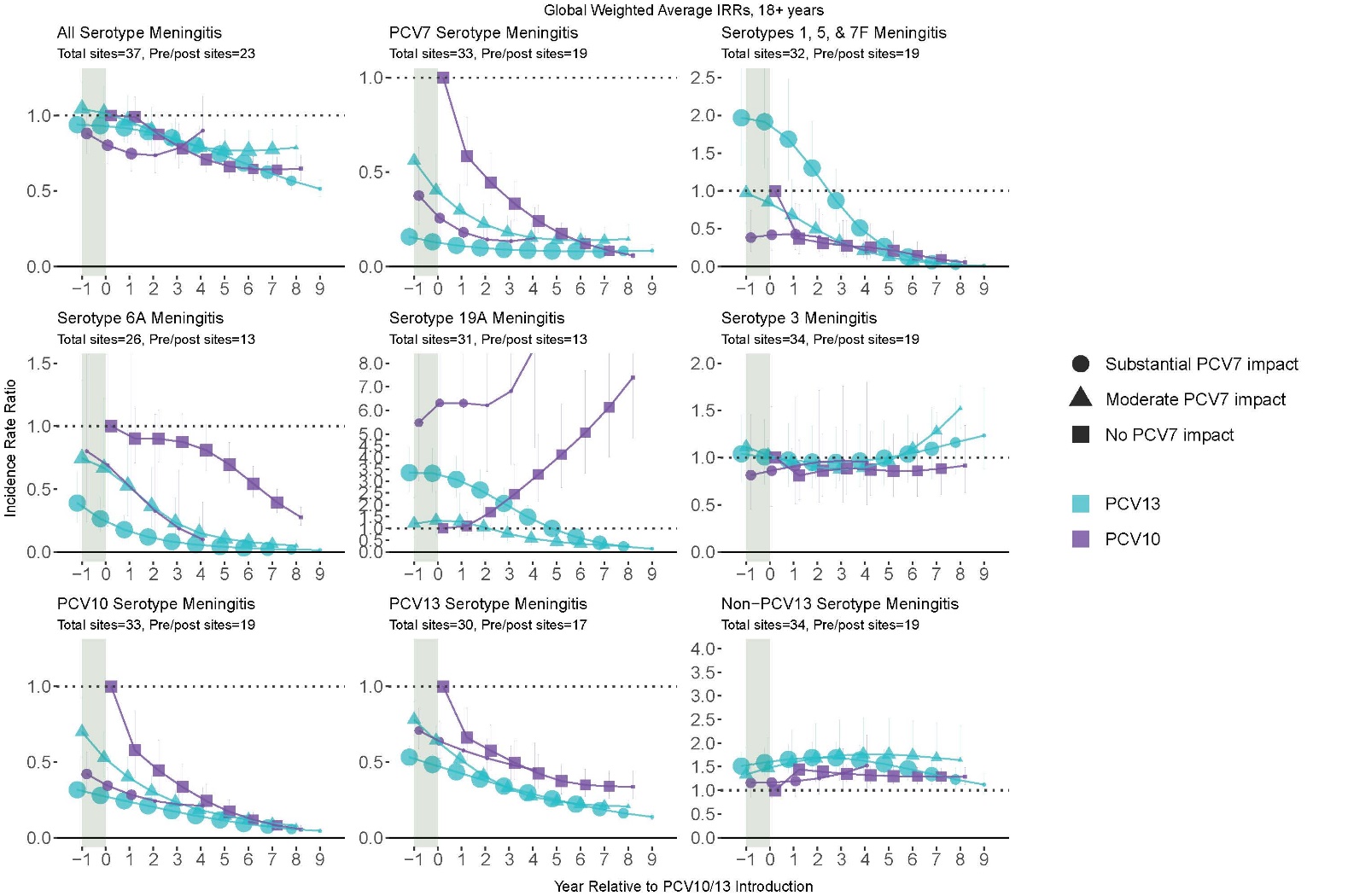


Appendix 2: All-site IPD vs CSF+ meningitis weighted average incidence rate ratios comparing the annual post-PCV10/13 incidence rate to the average pre-PCV incidence rate

## Supplementary Figure 7. IPD vs CSF+ meningitis, children <5 years


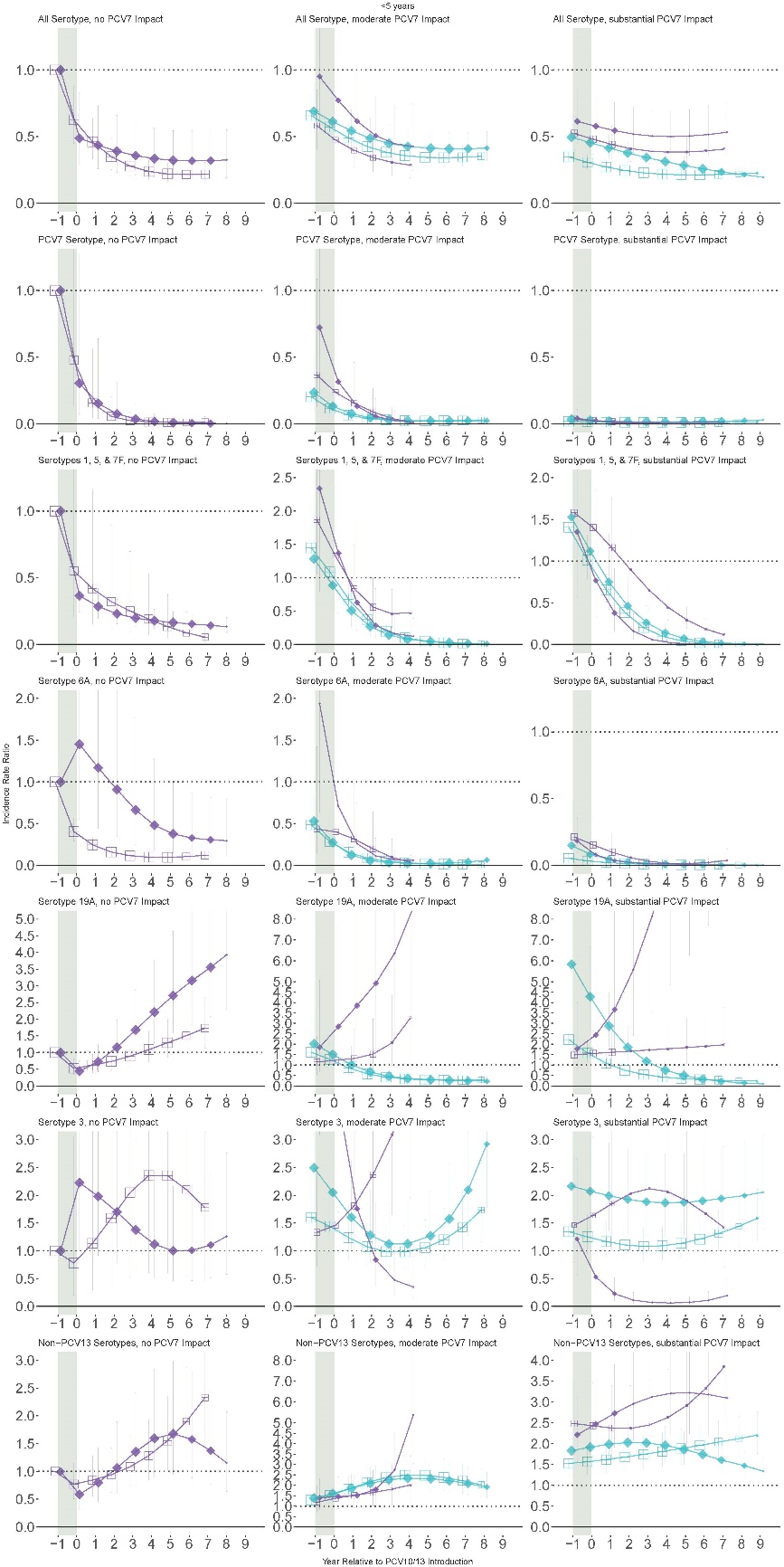

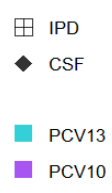


## Supplementary Figure 8. IPD vs CSF+ meningitis, children 5-17 years


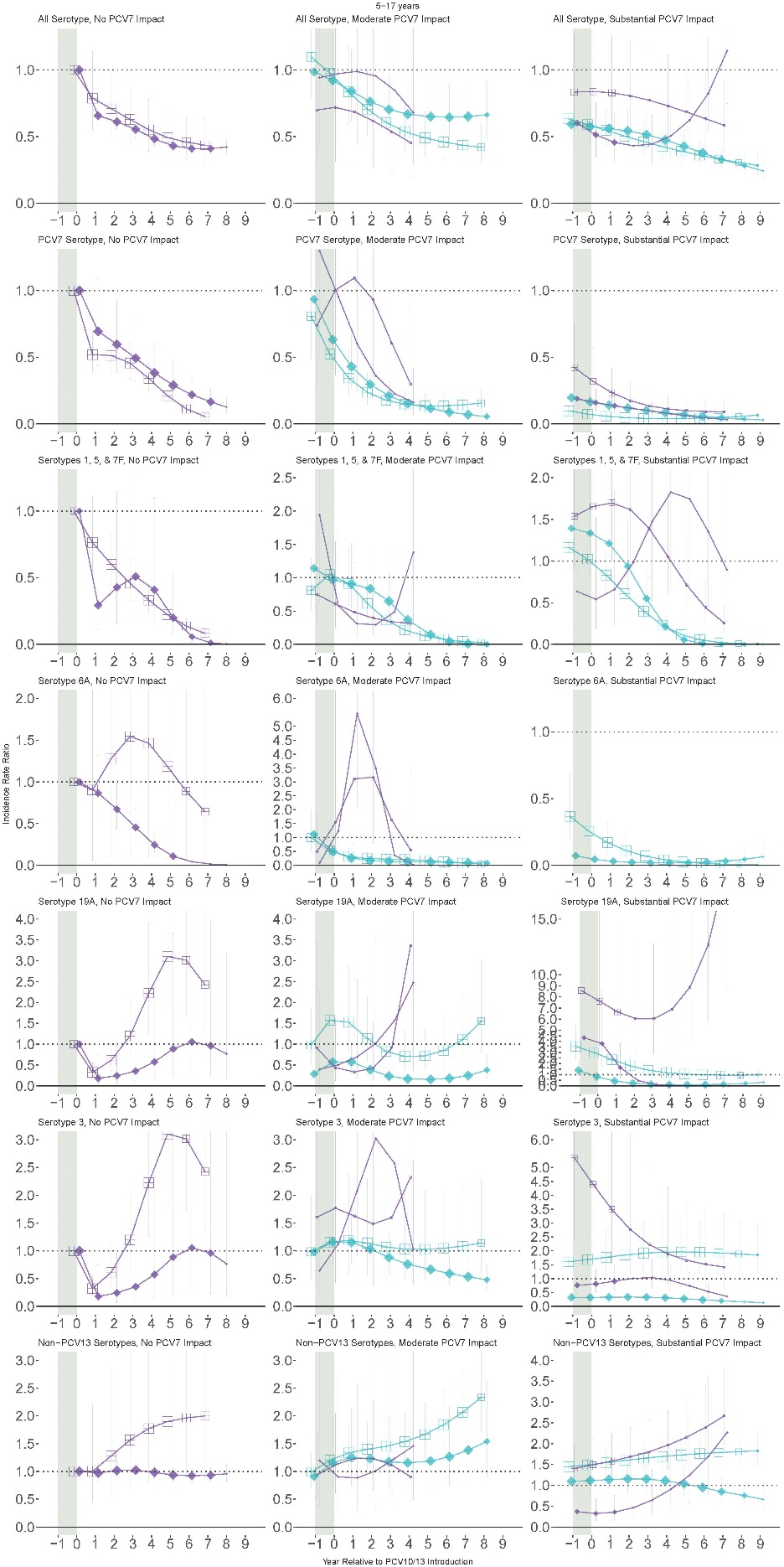

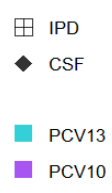


## Supplementary Figure 9. IPD vs CSF+ meningitis, adults 18 years and older


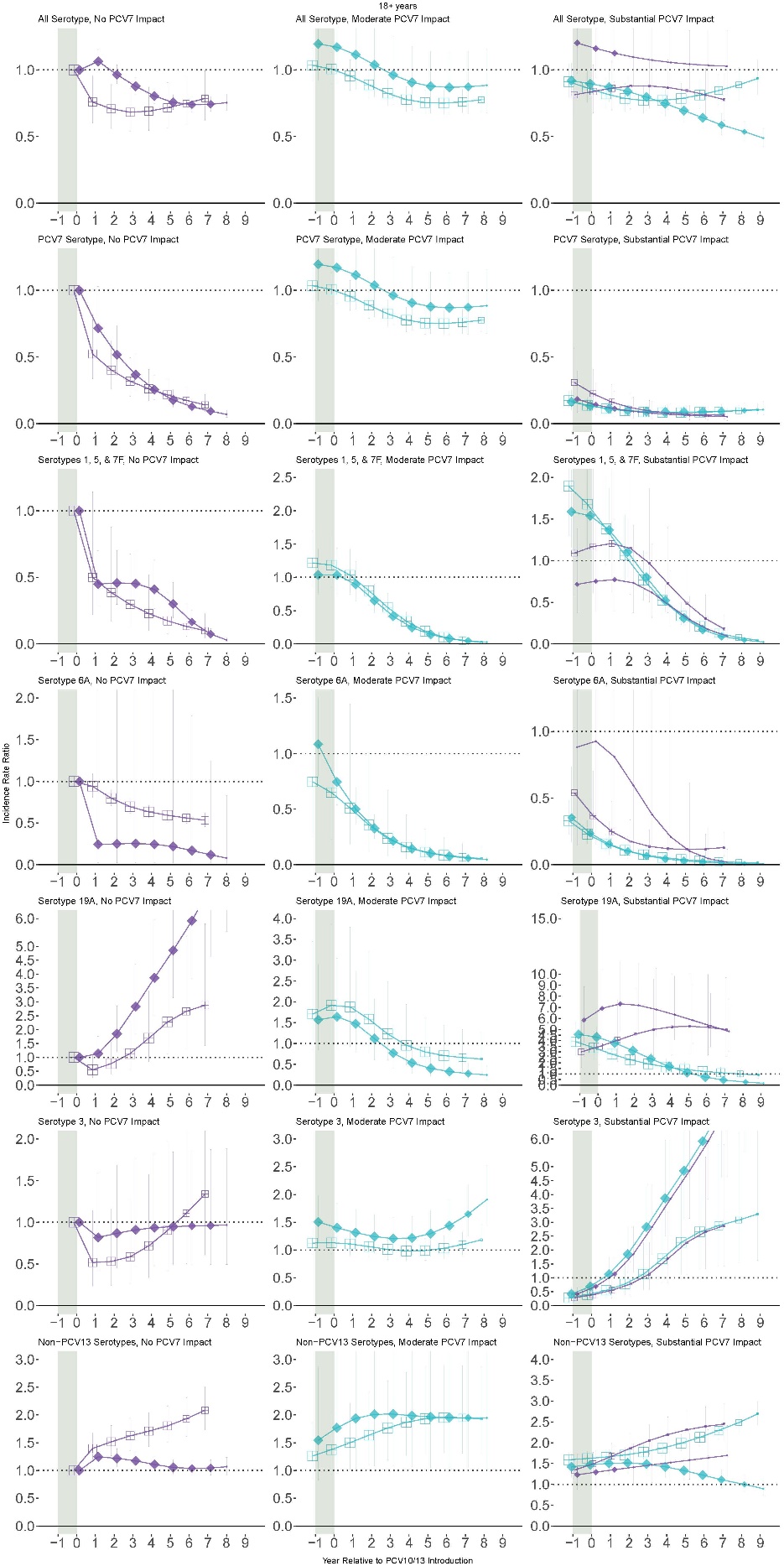

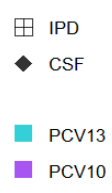


Appendix 3: All-site CSF+ meningitis weighted average incidence rate ratios comparing the annual post-PCV10/13 incidence rate to the average pre-PCV incidence rate

## Supplementary Figure 10. All meningitis, children <5 years


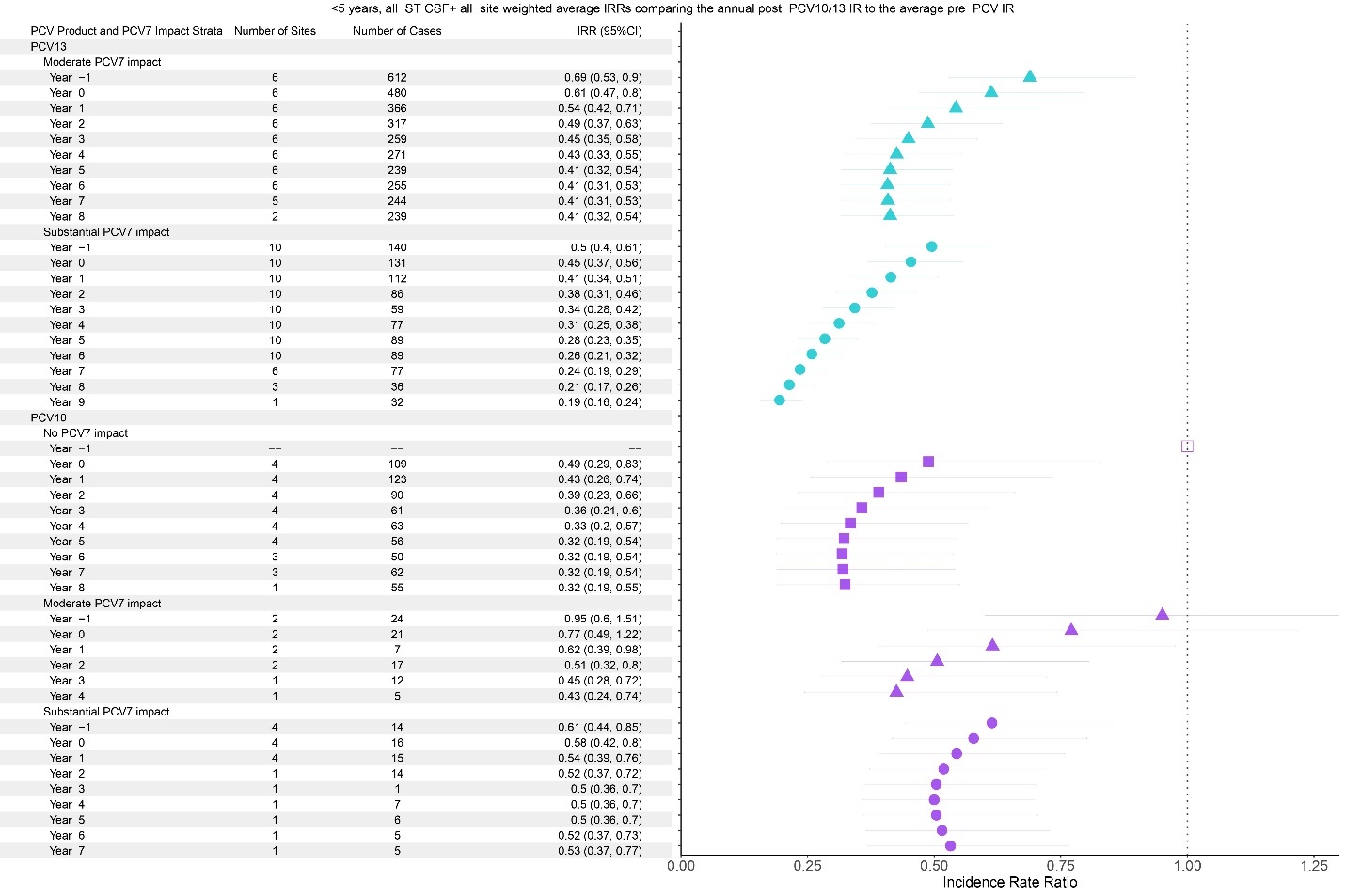


Supplementary Figure 11. All meningitis, children 5-17 years.


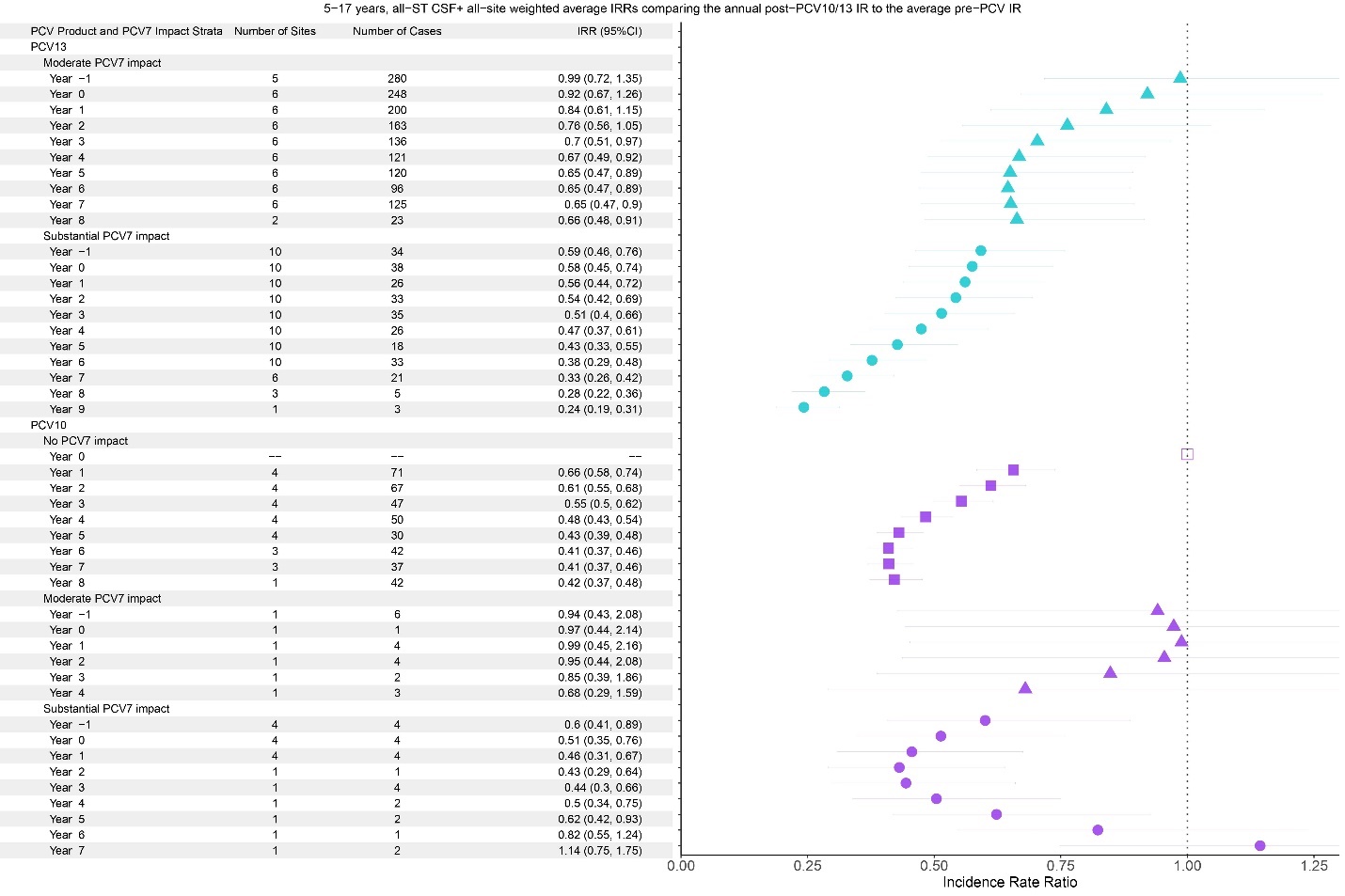


Supplementary Figure 12. All meningitis, adults >18 years.


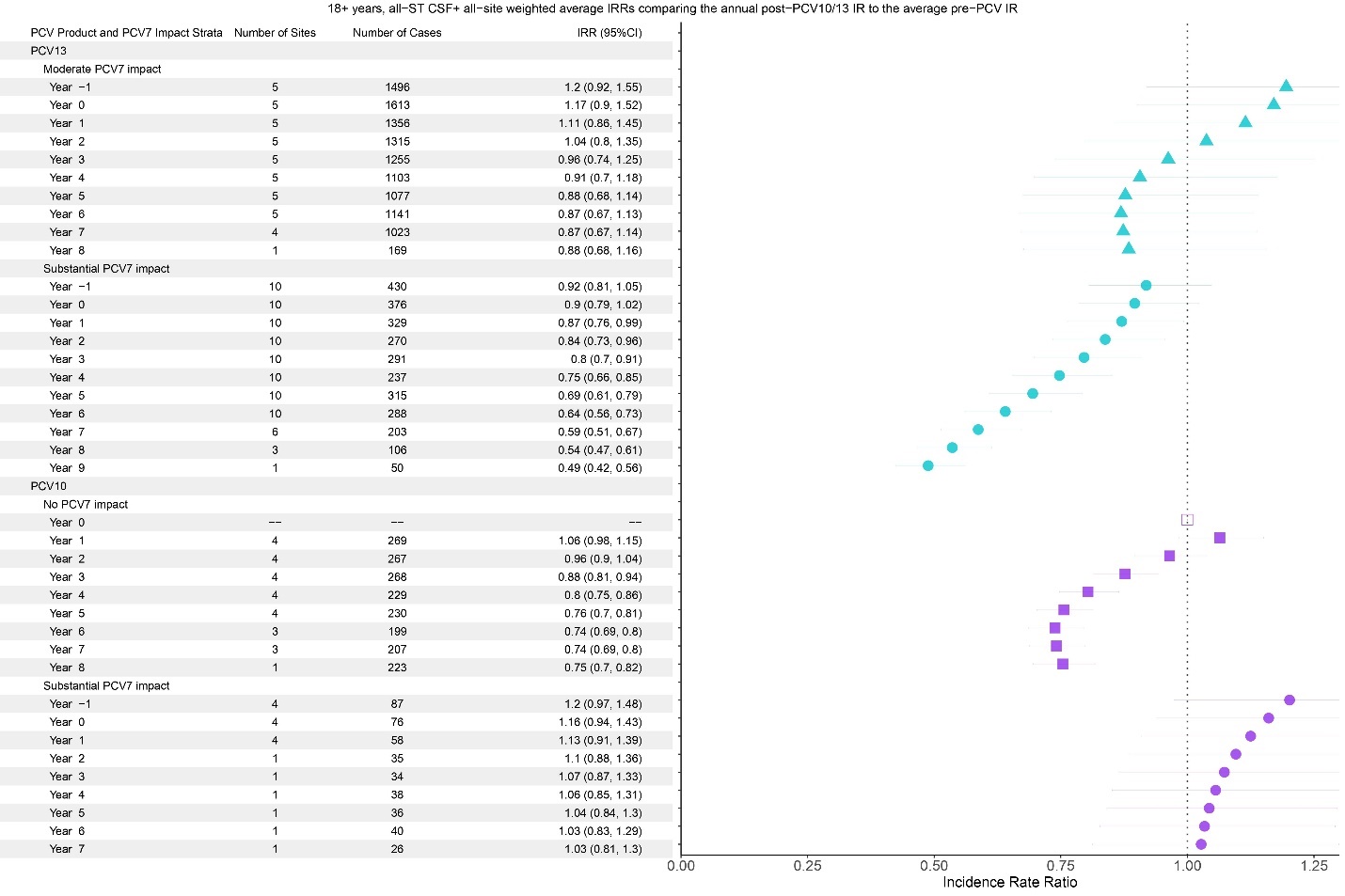


## Supplementary Figure 13. PCV7-type, children <5 years.


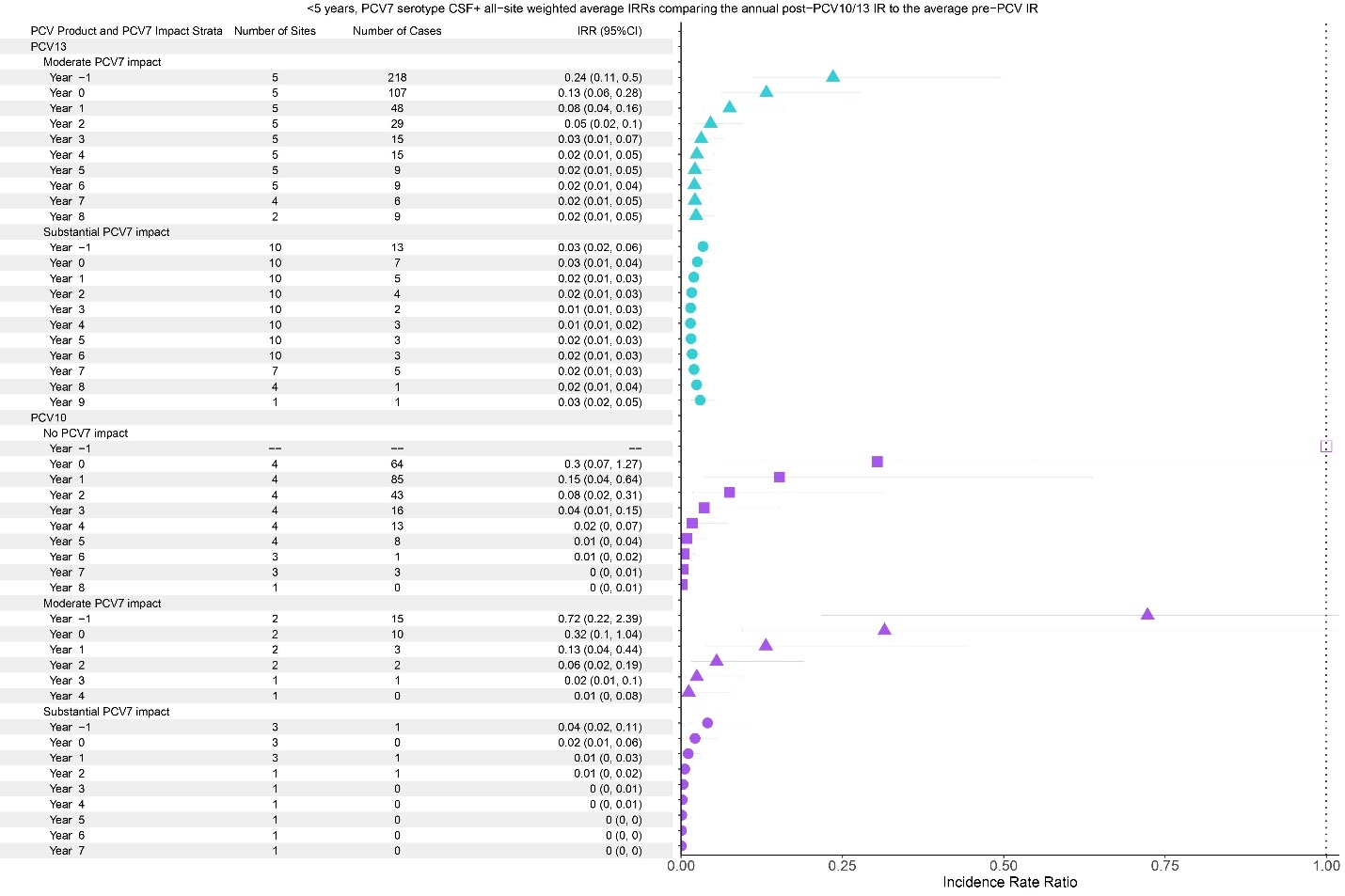


## Supplementary Figure 14. PCV7-type , children 5-17 years.


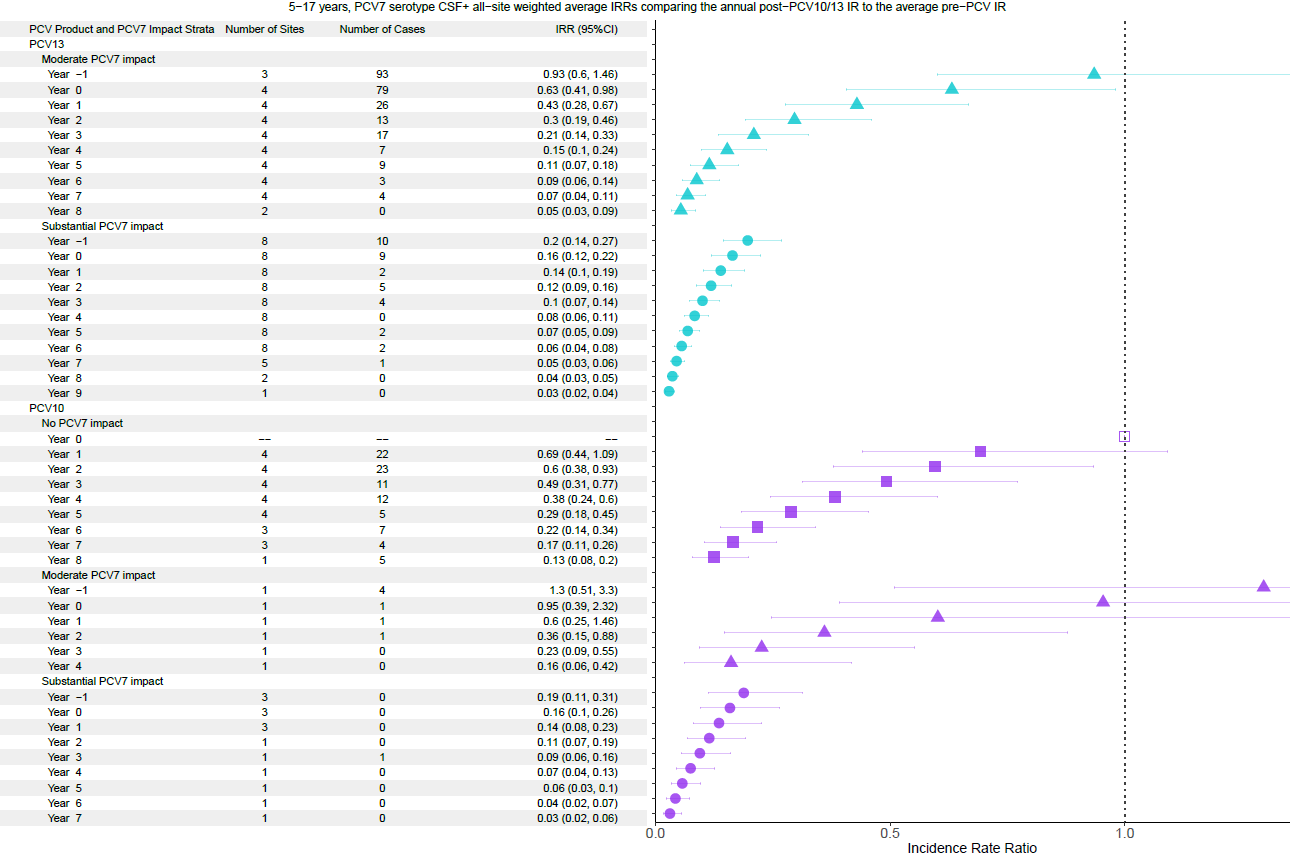


## Supplementary Figure 15. PCV7-type, adults >18 years.


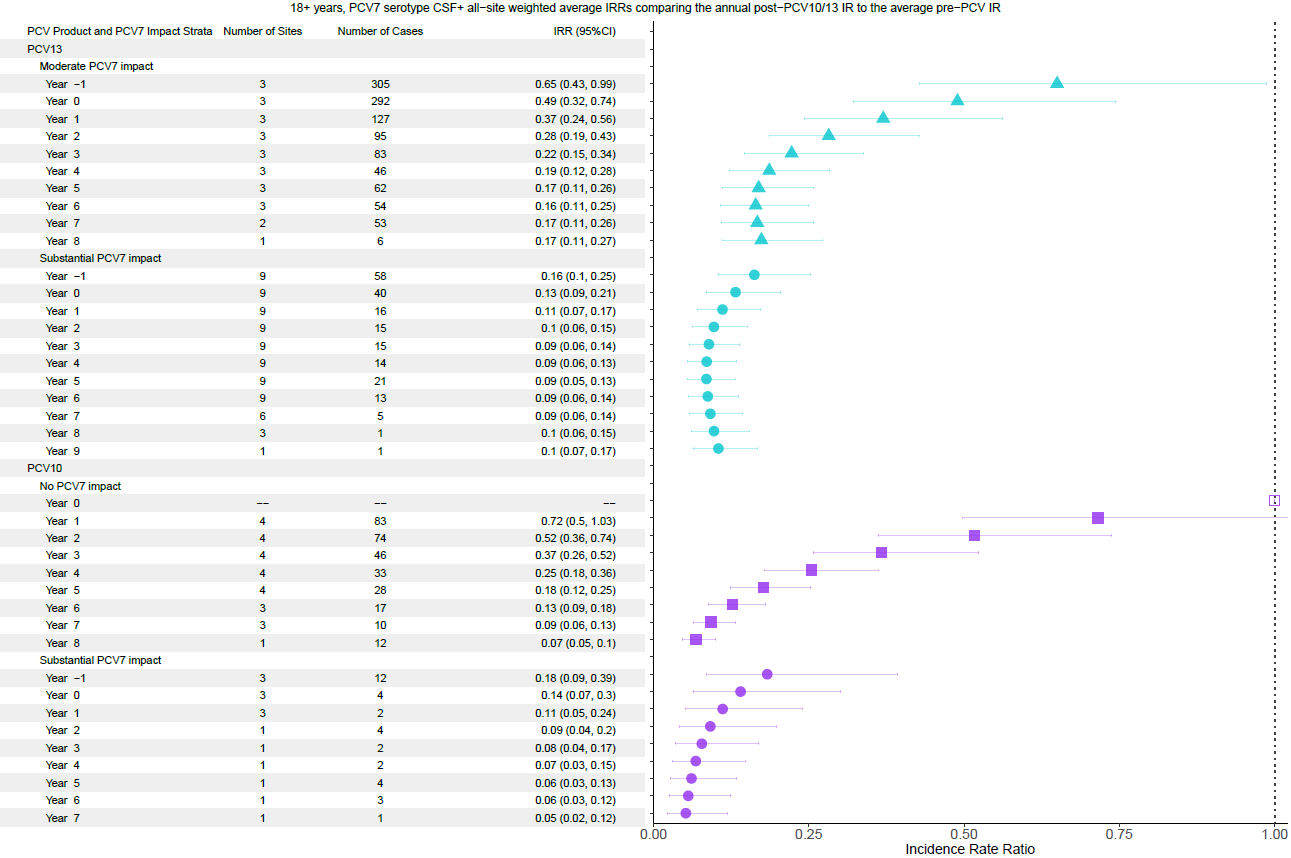


Supplementary Figure 16. ST1, 5, 7F all, children <5 years.
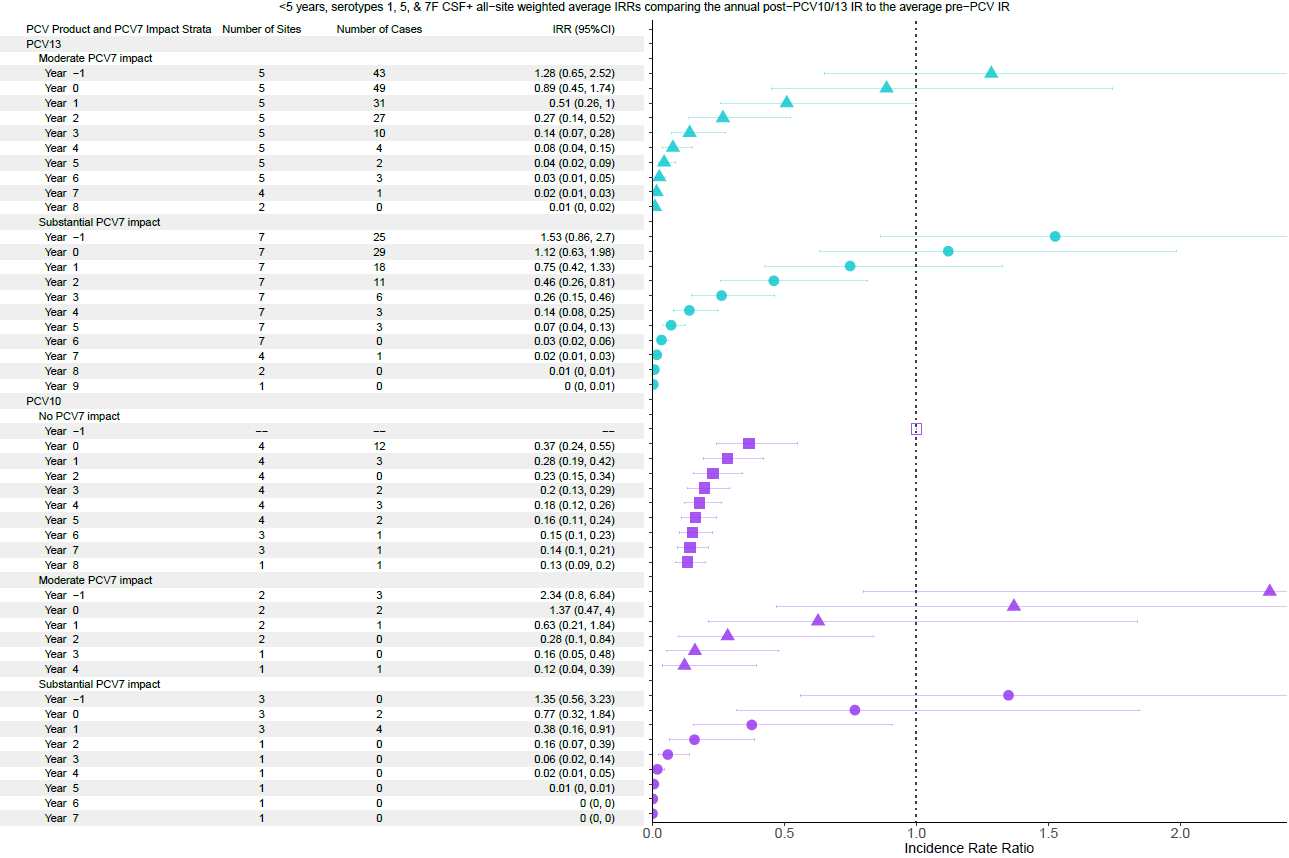


## Supplementary Figure 17. ST1, 5, 7F , children 5-17 years.


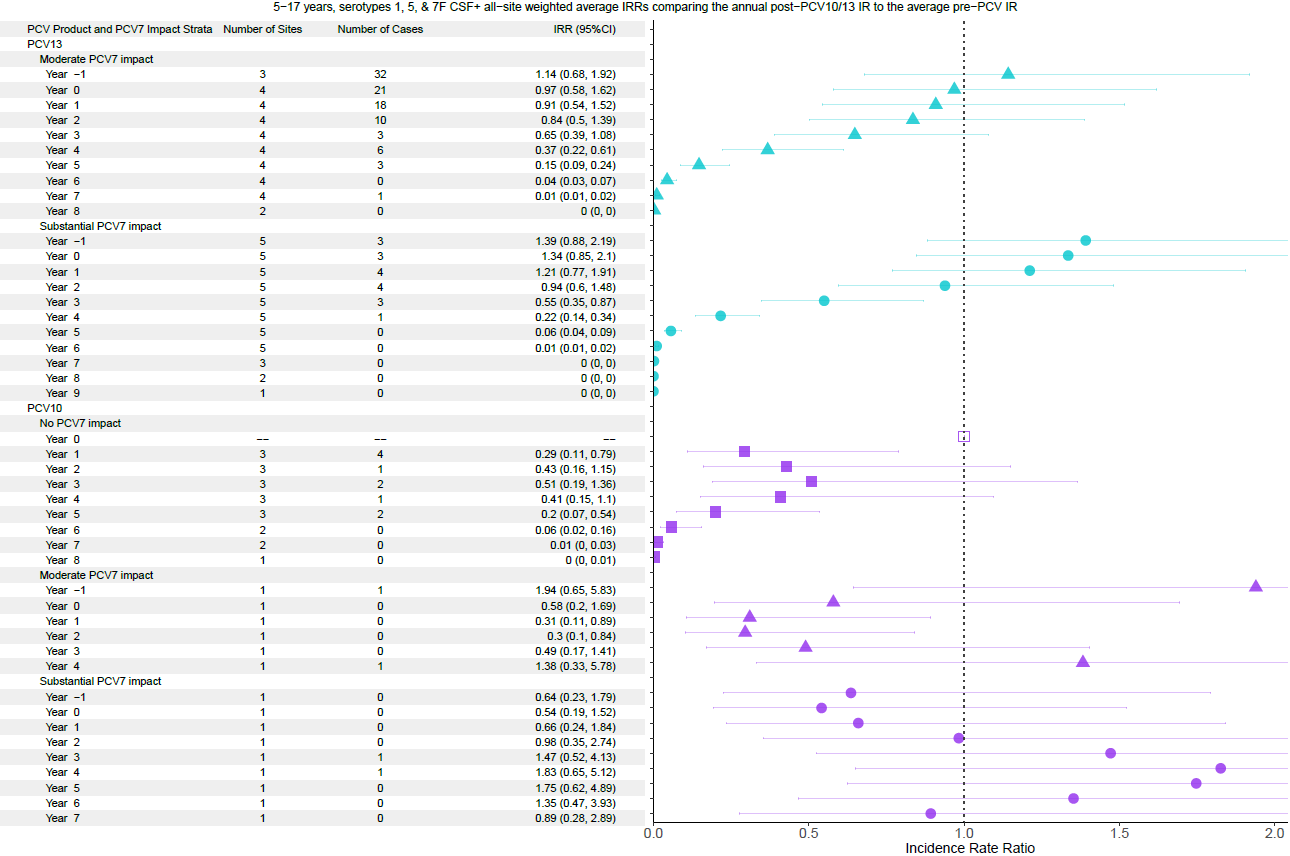


## Supplementary Figure 18. ST1, 5, 7F, adults >18 years.


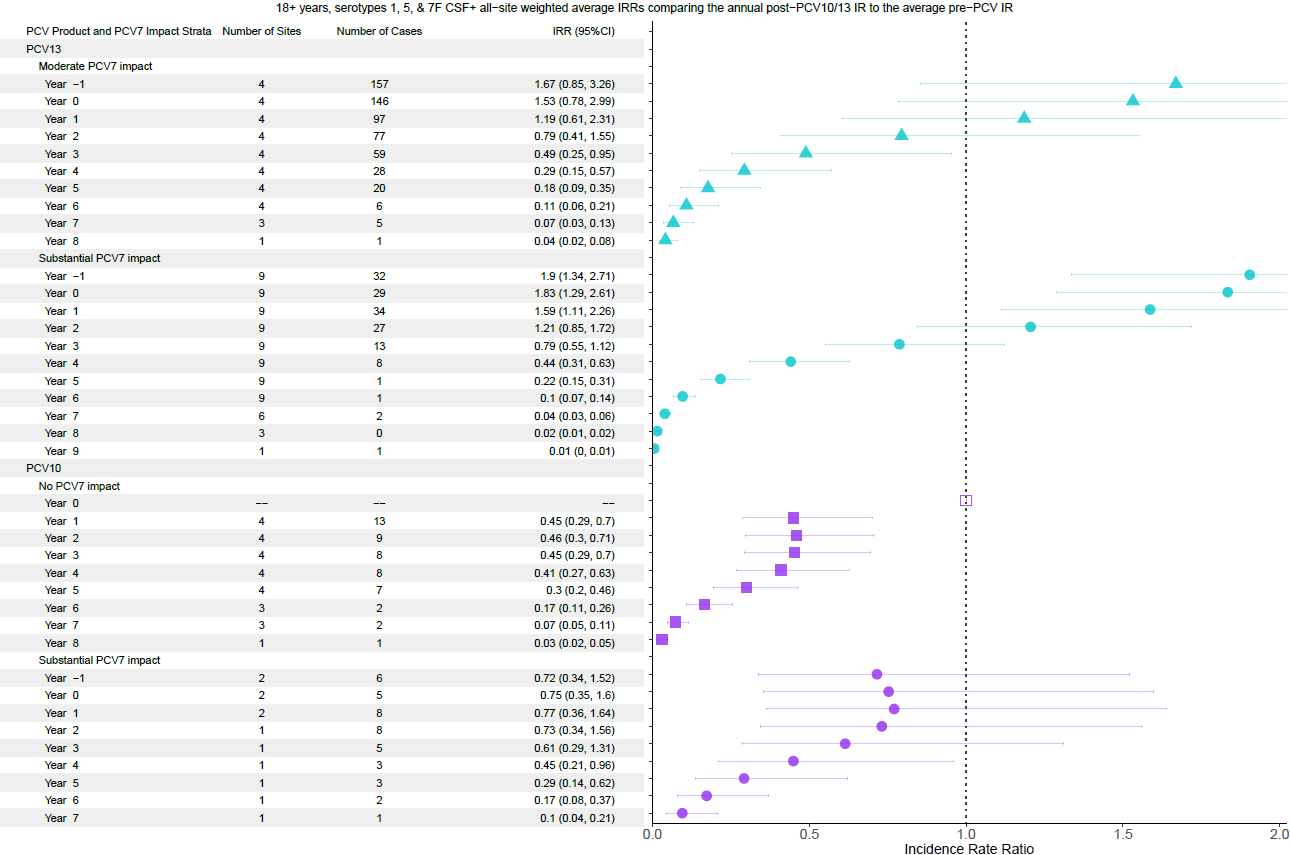


## Supplementary Figure 19. PCV10-type, children <5 years.


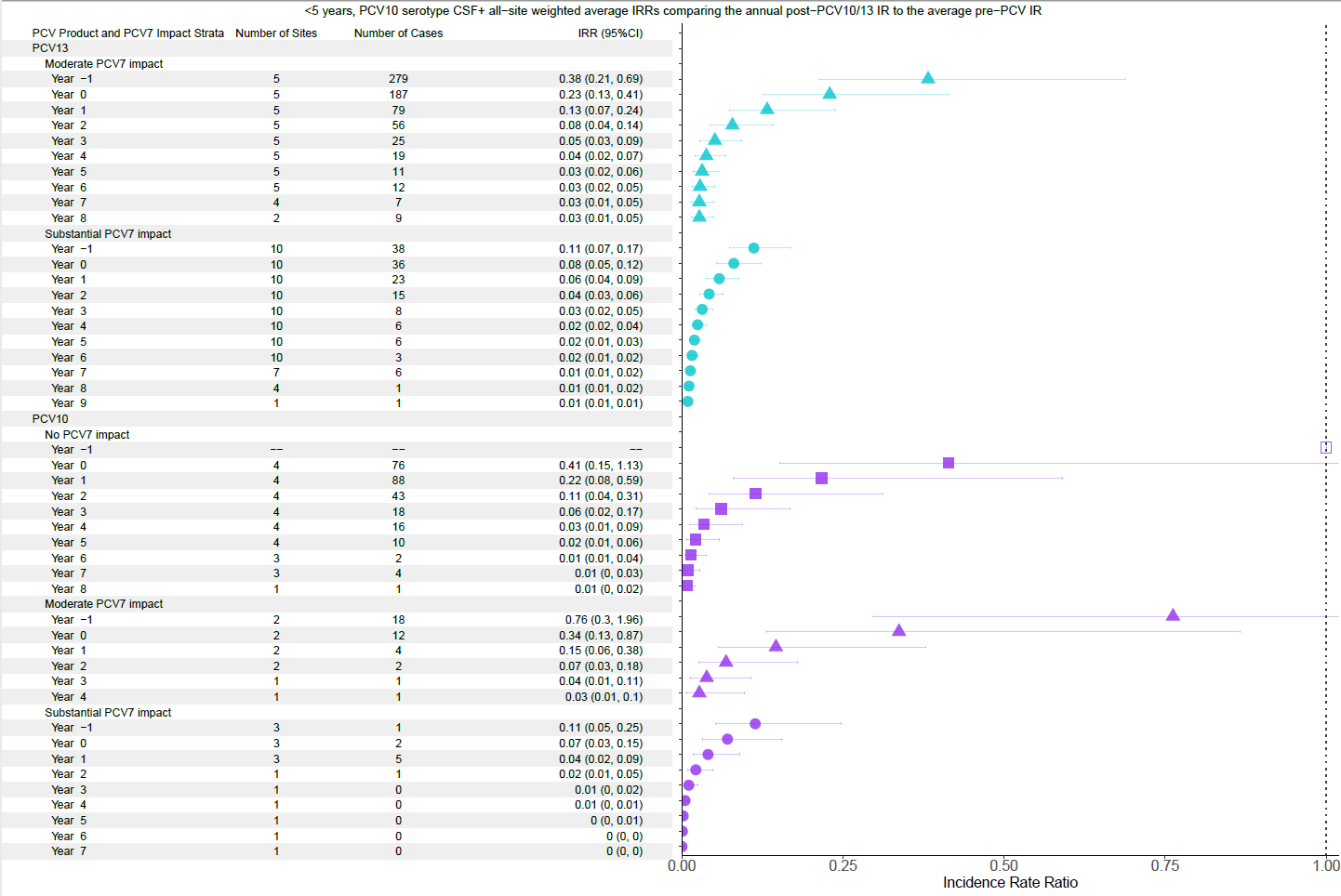


## Supplementary Figure 20. PCV10-type, children 5-17 years.


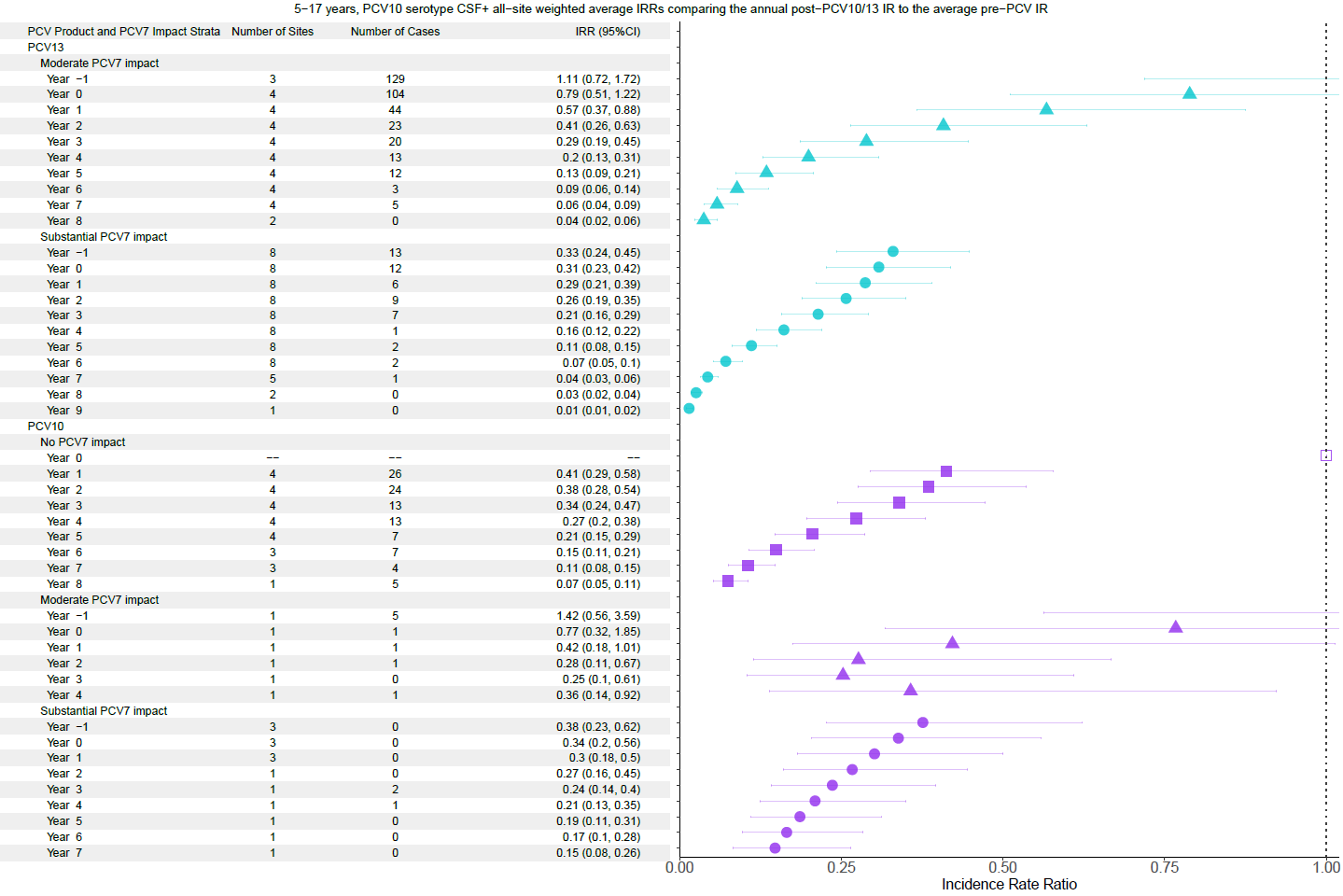


## Supplementary Figure 21. PCV10-type all, adults >18 years.


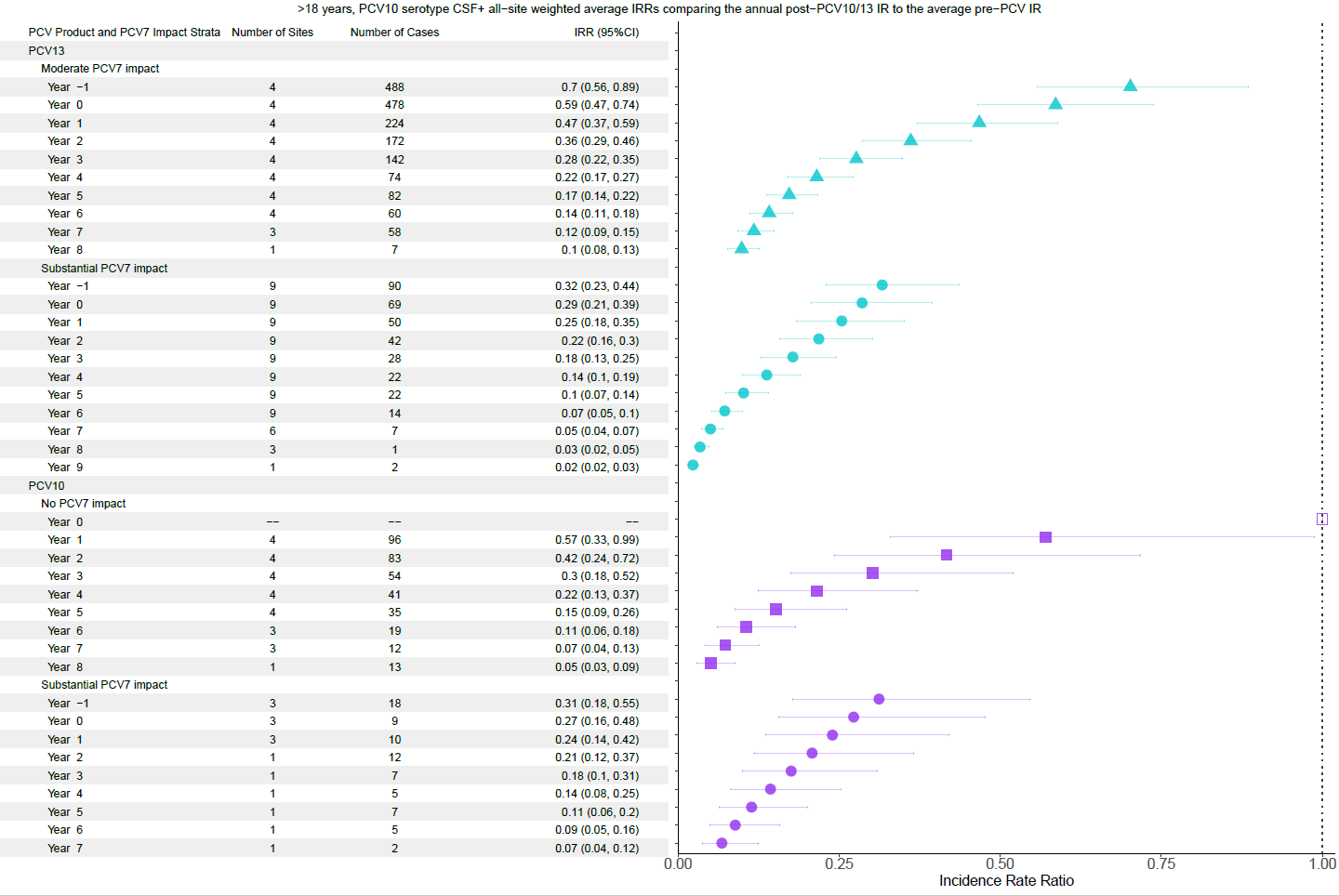


## Supplementary Figure 22. ST6A, children <5 years.


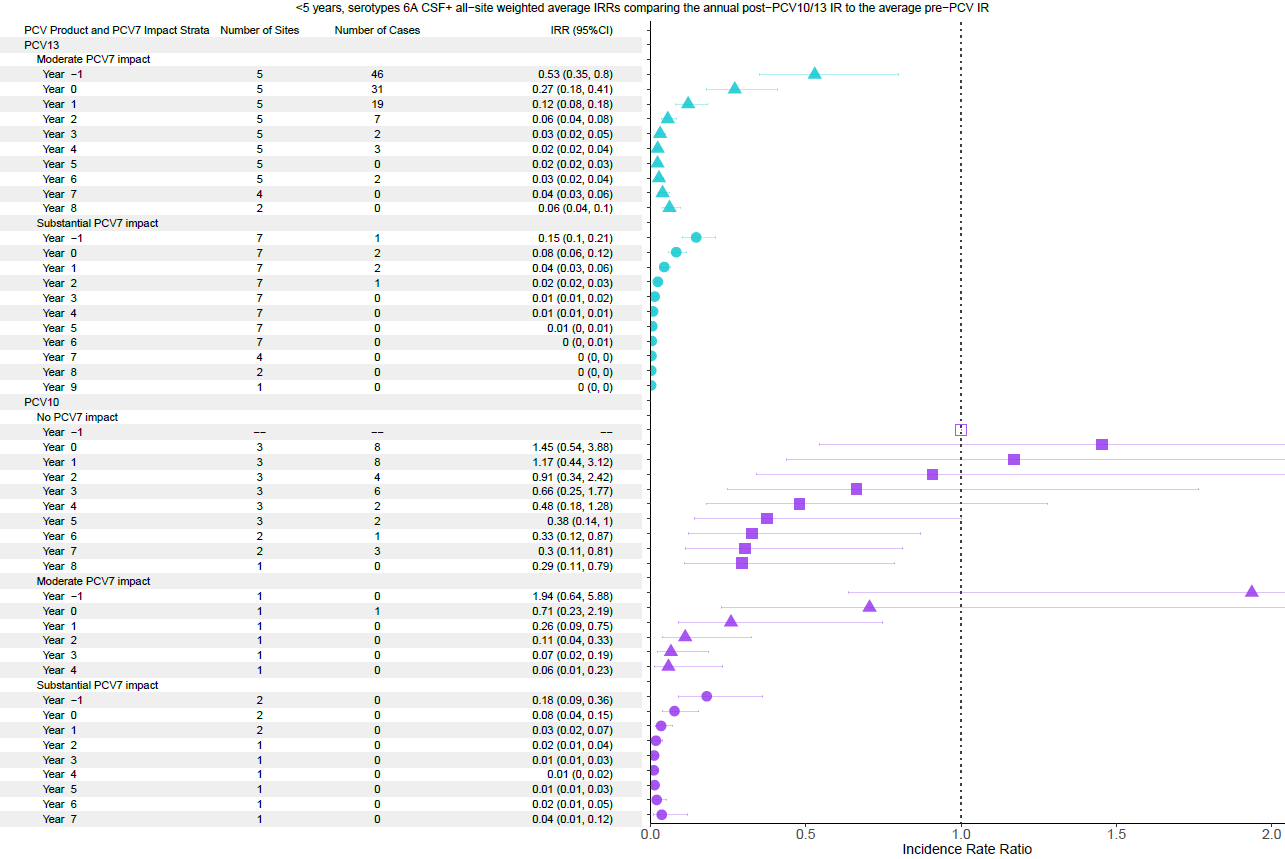


## Supplementary Figure 23. ST6A, children 5-17 years.


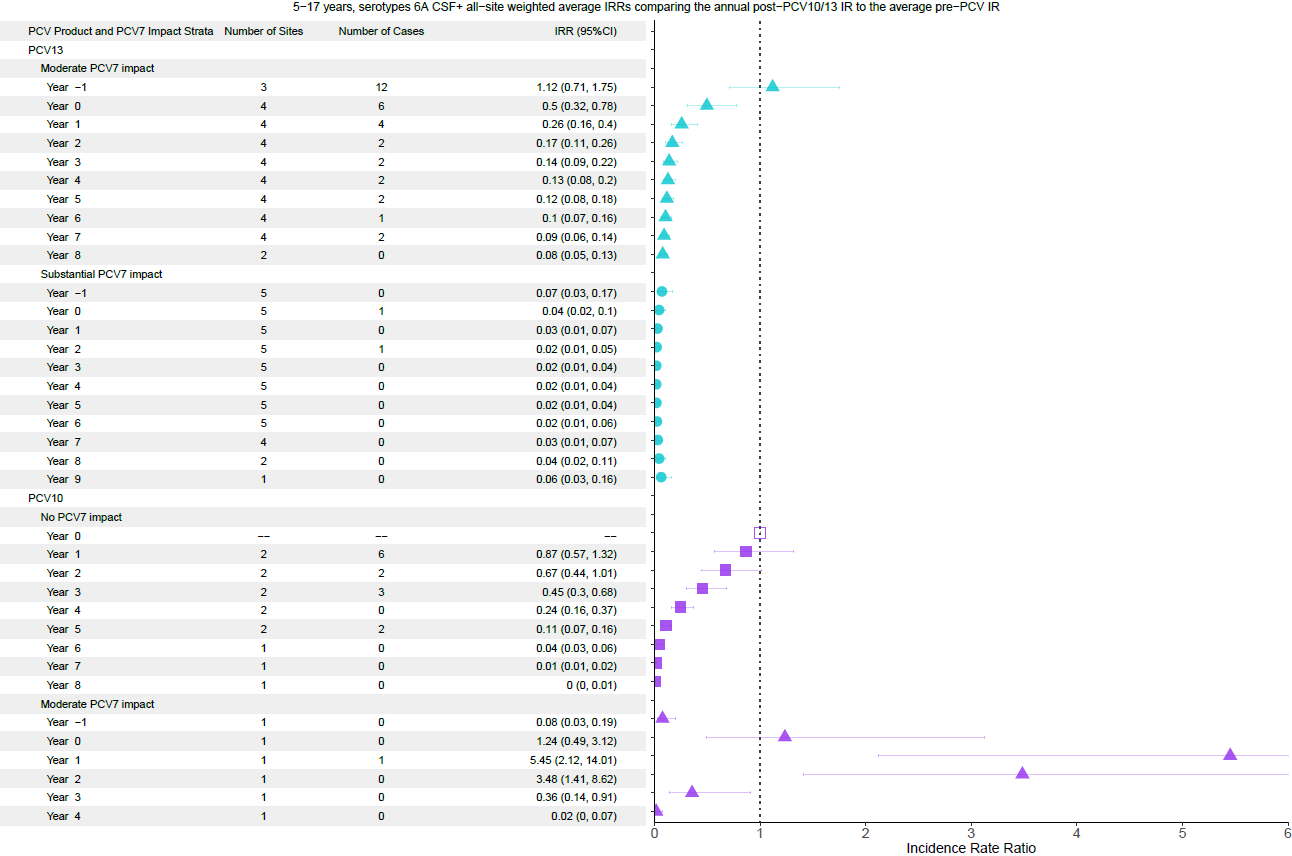


## Supplementary Figure 24. ST6A, adults >18 years.


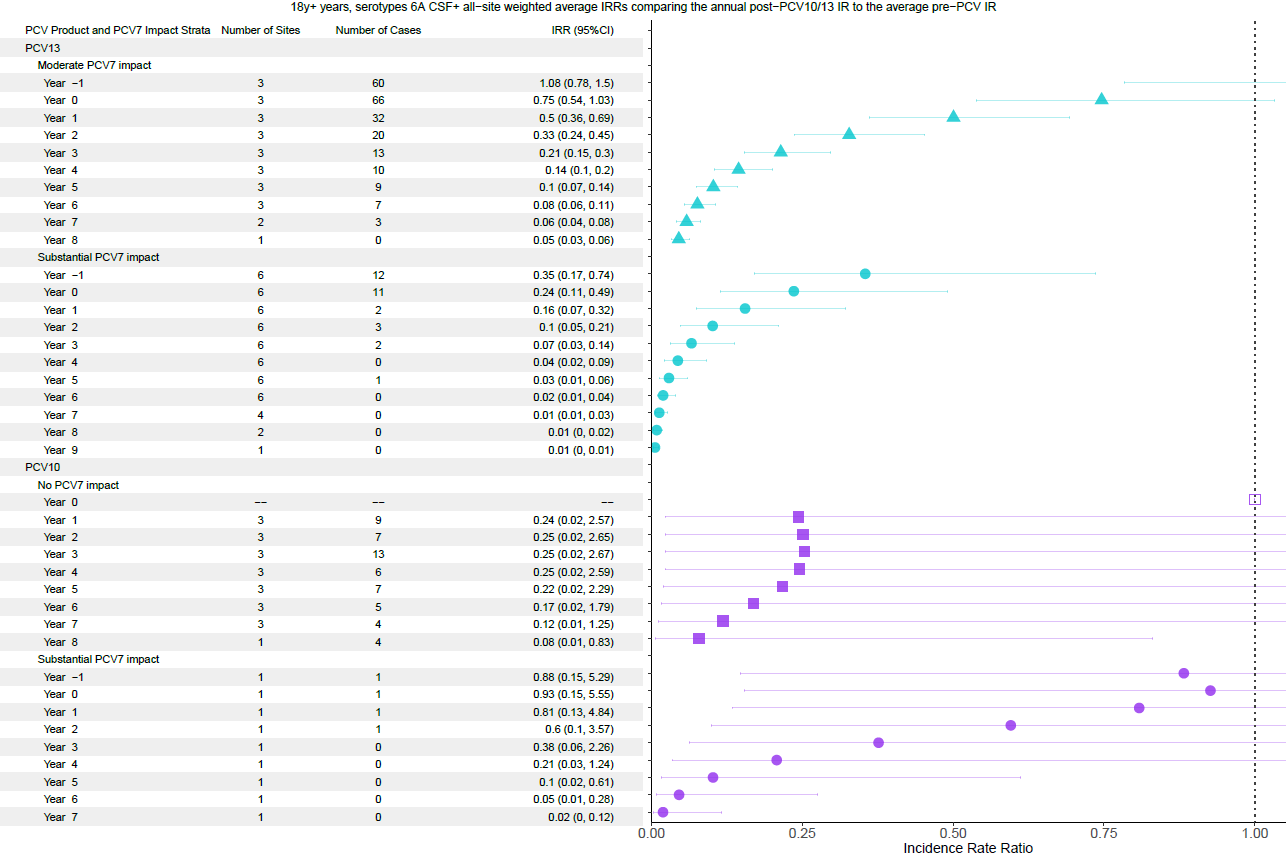


## Supplementary Figure 25. ST19A, children <5 years.


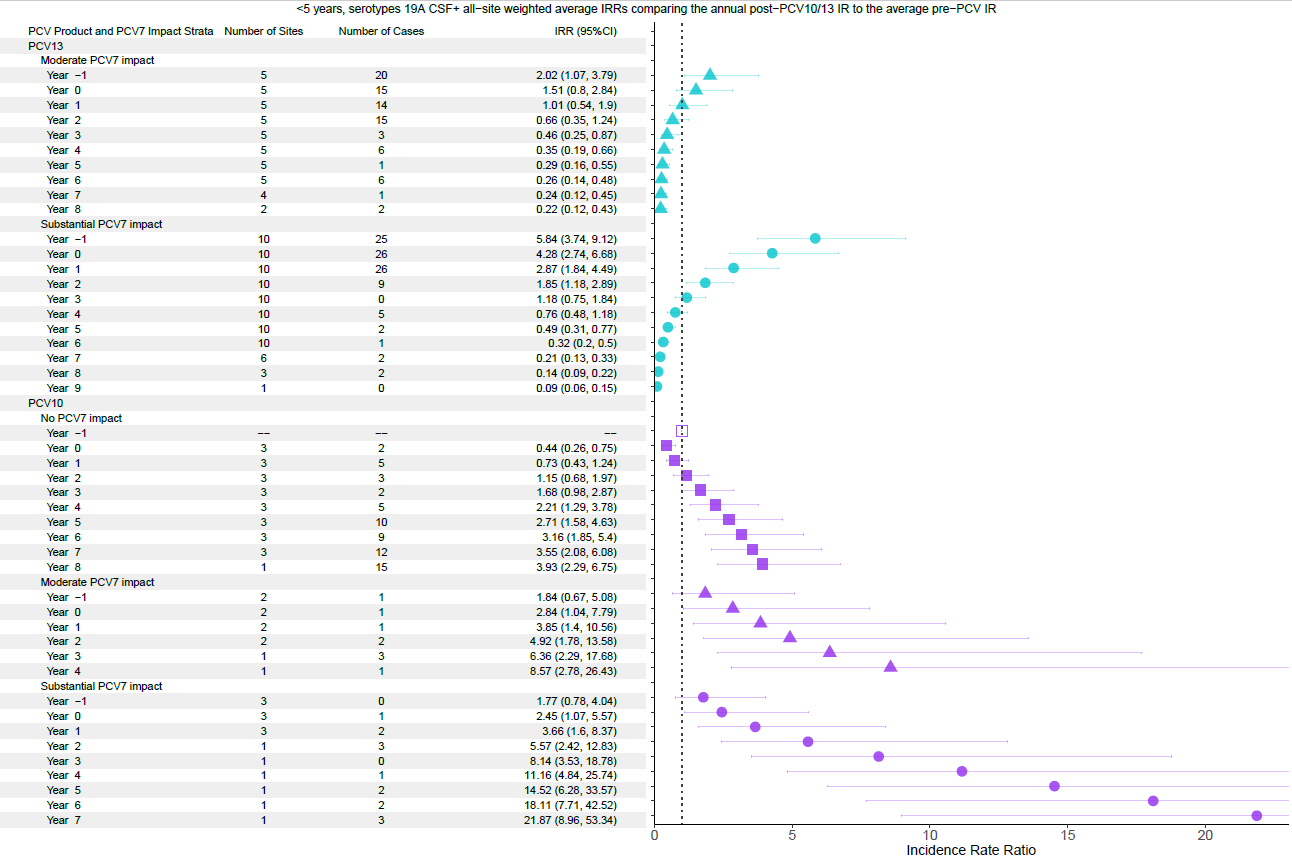


## Supplementary Figure 26. ST19A, children 5-17 years.


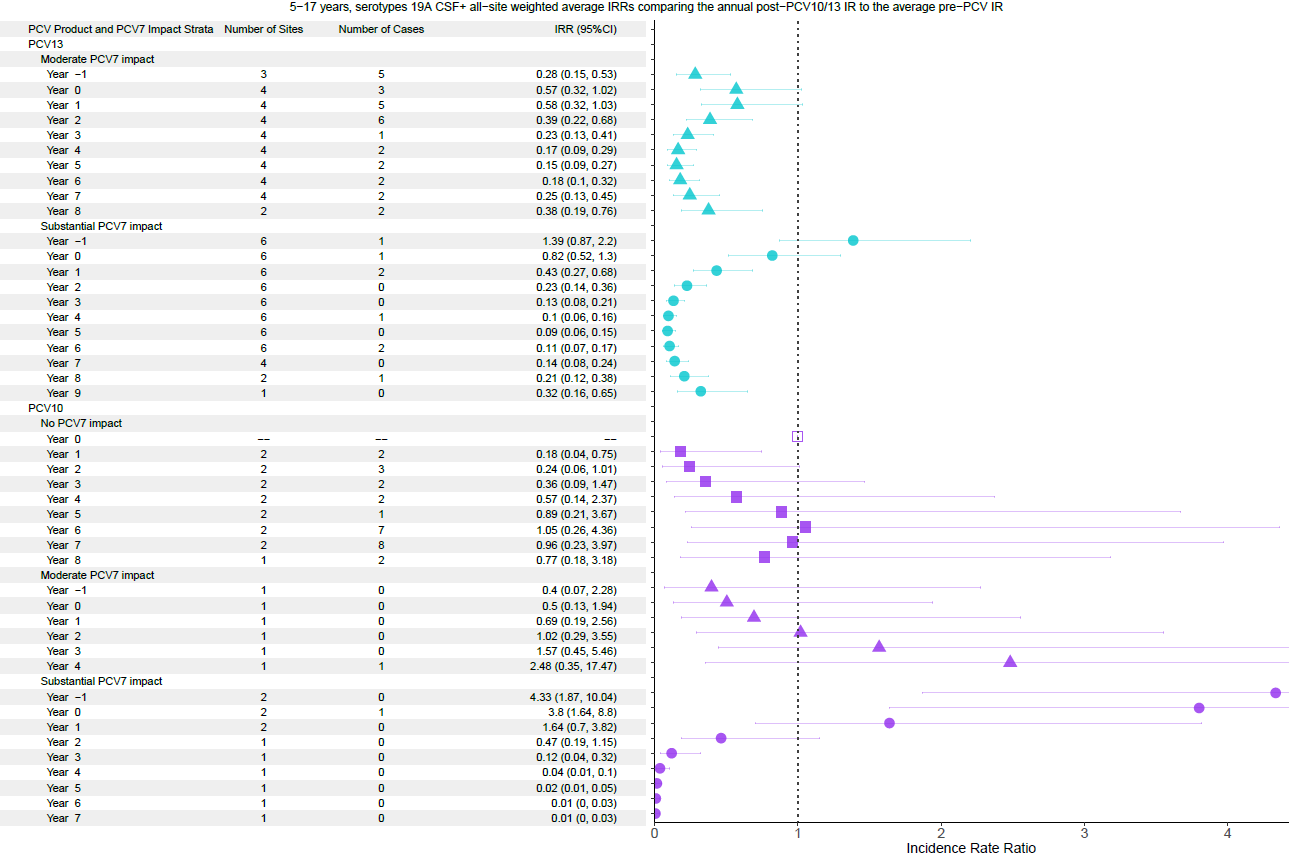


## Supplementary Figure 27. ST19A, adults >18 years.


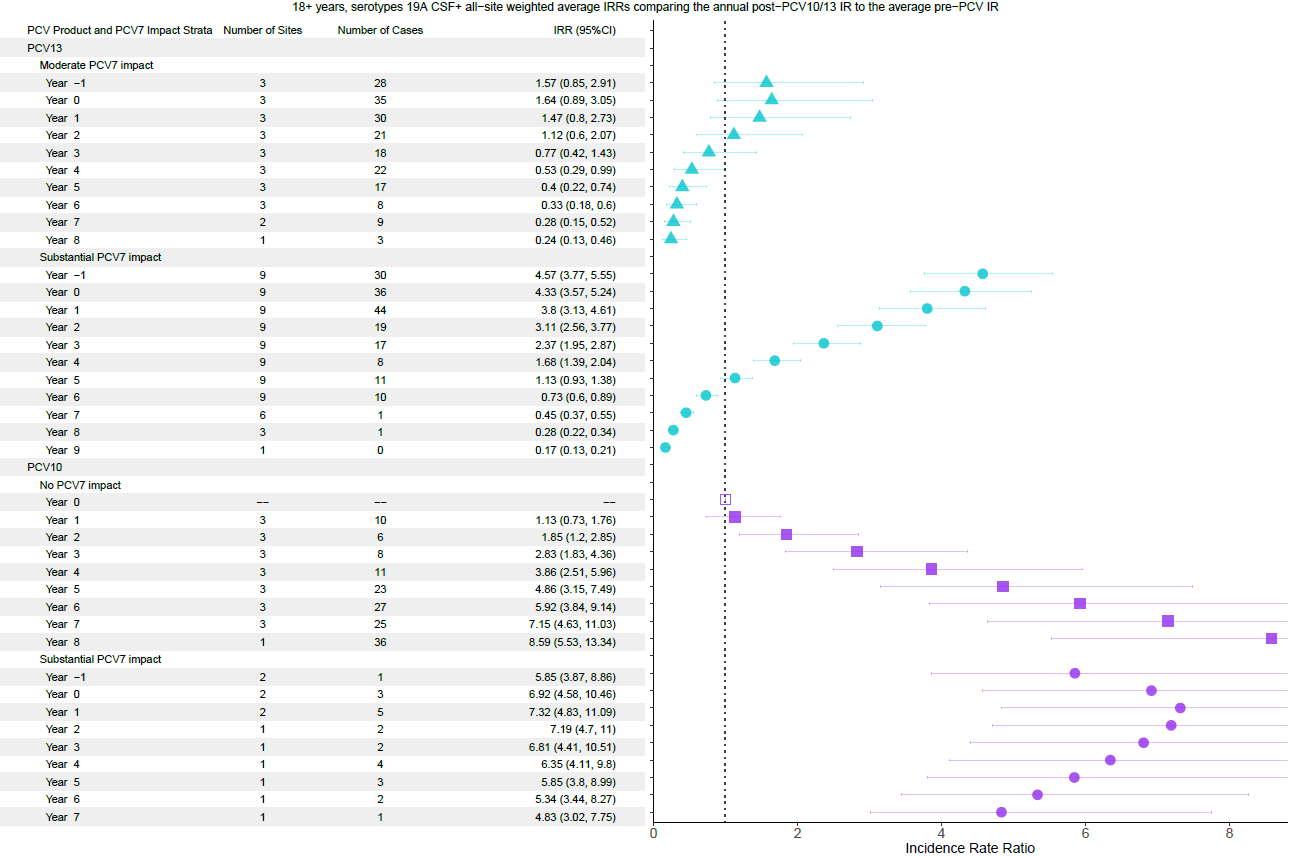


## Supplementary Figure 28. ST3, children <5 years.


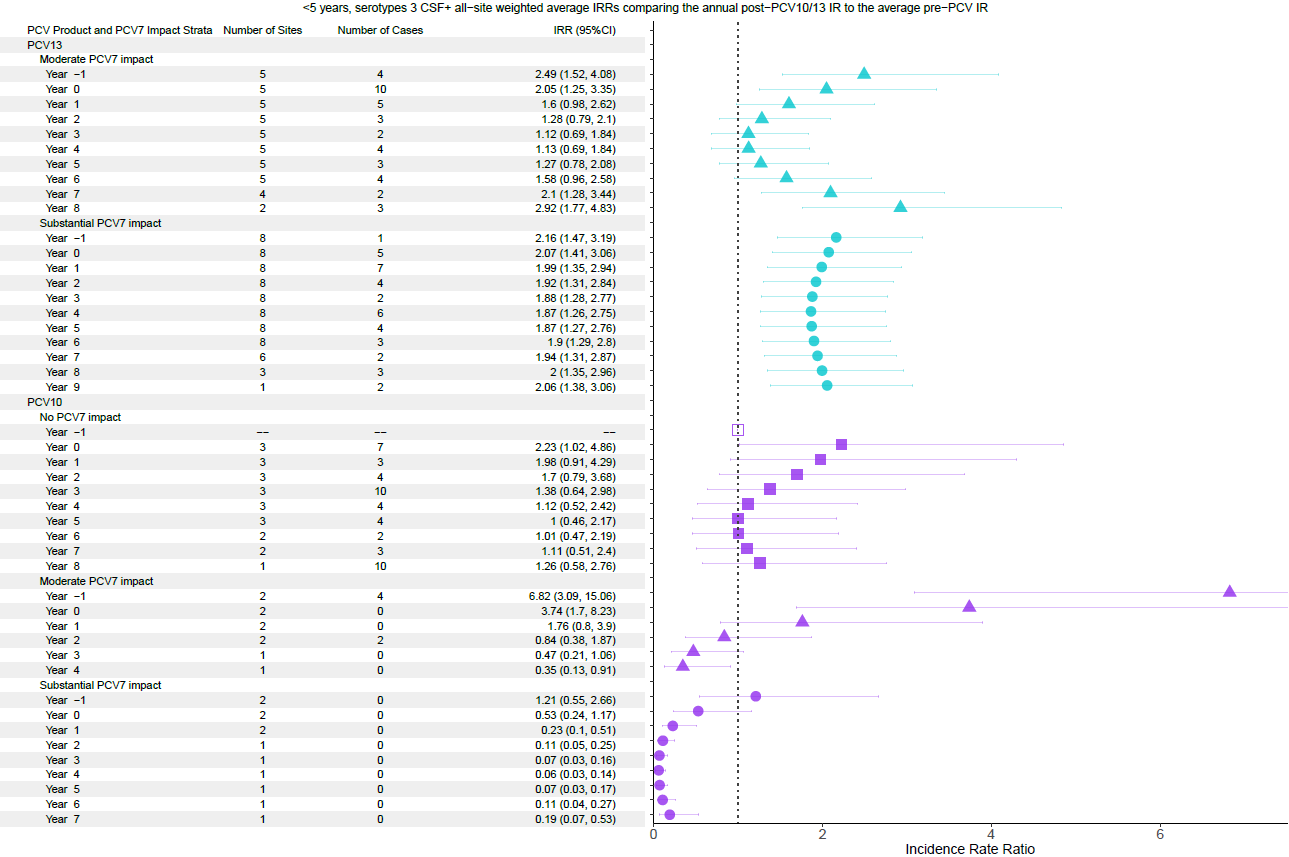


## Supplementary Figure 29. ST3, children 5-17 years.


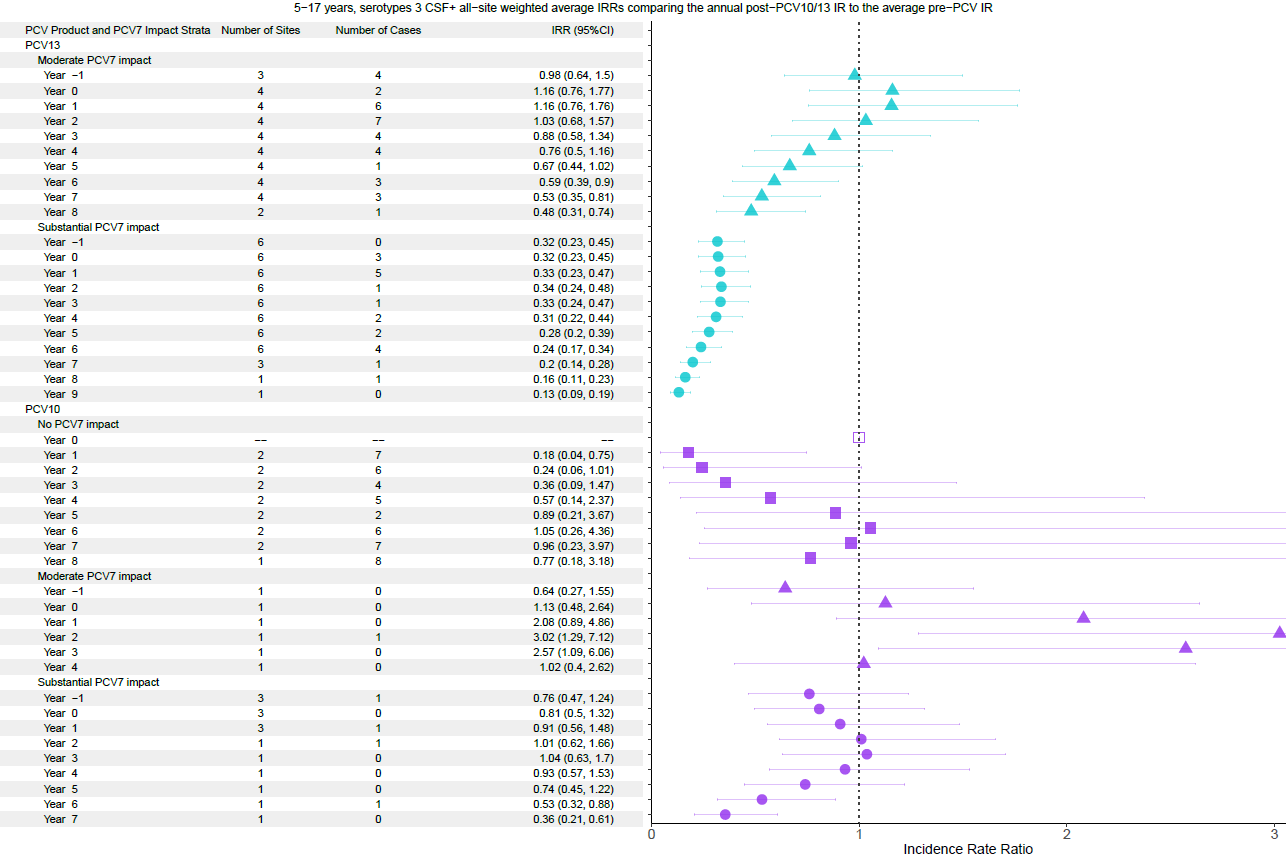


## Supplementary Figure 30. ST3, adults >18 years.


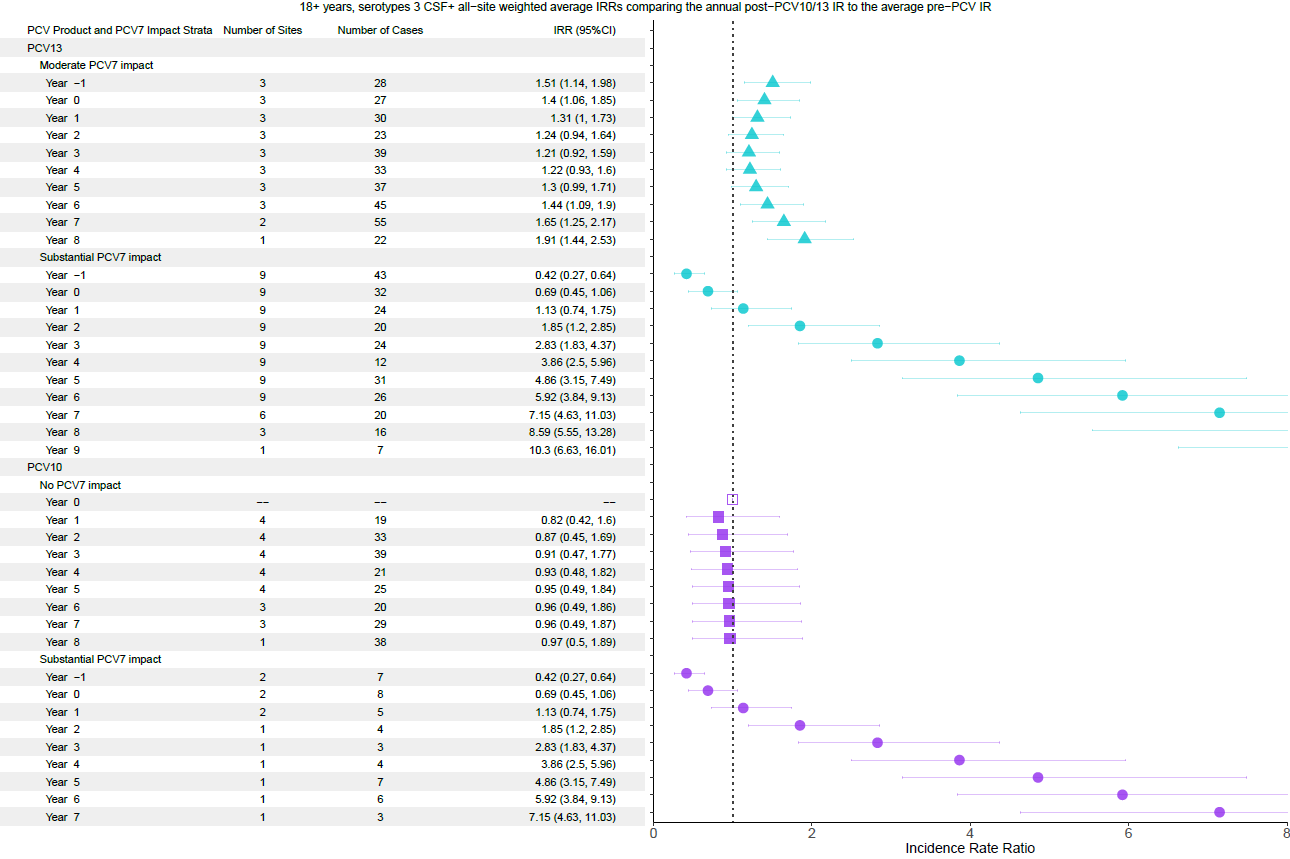


## Supplementary Figure 31. PCV13-type, children <5 years.


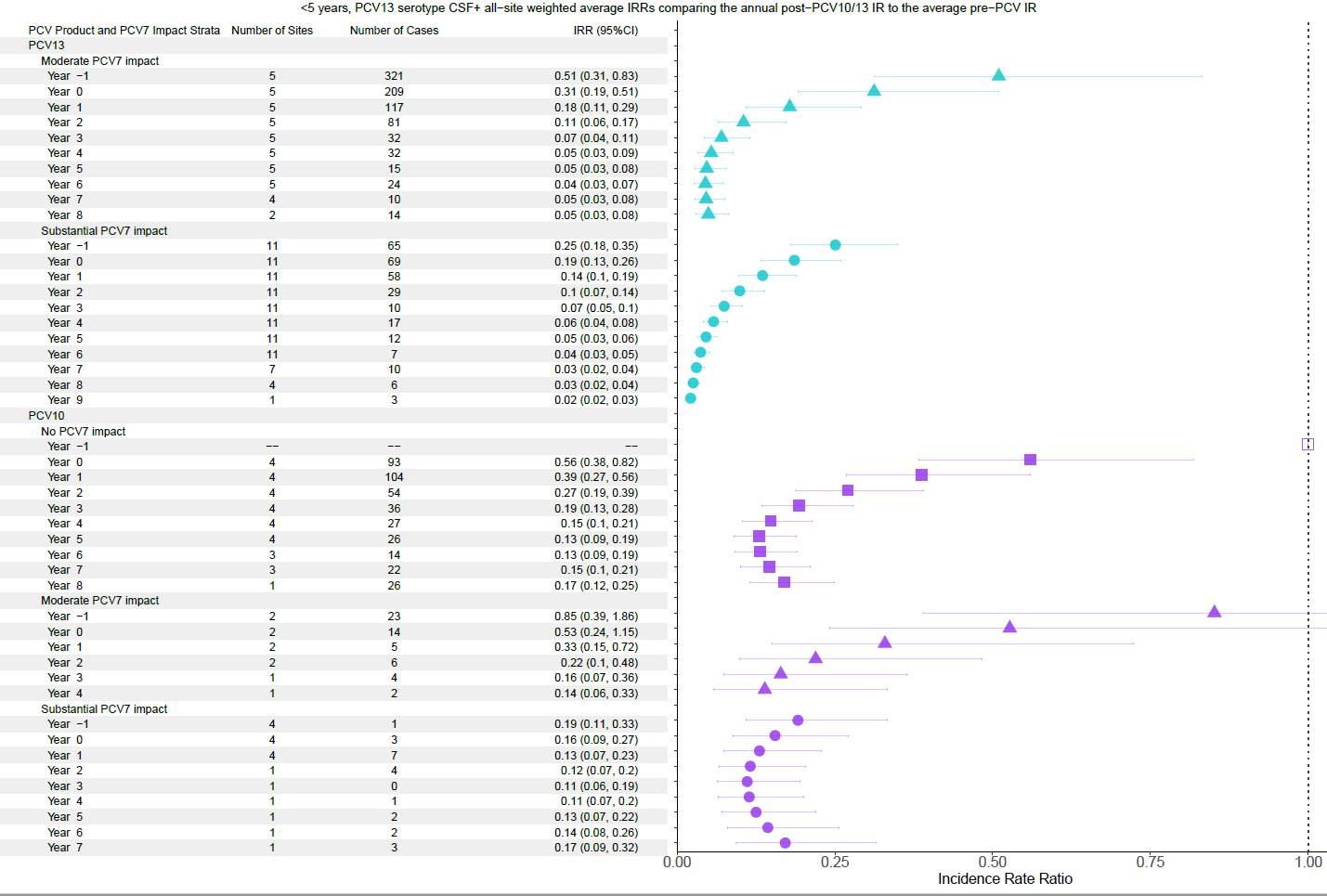


## Supplementary Figure 32. PCV13-type, children 5-17 years.


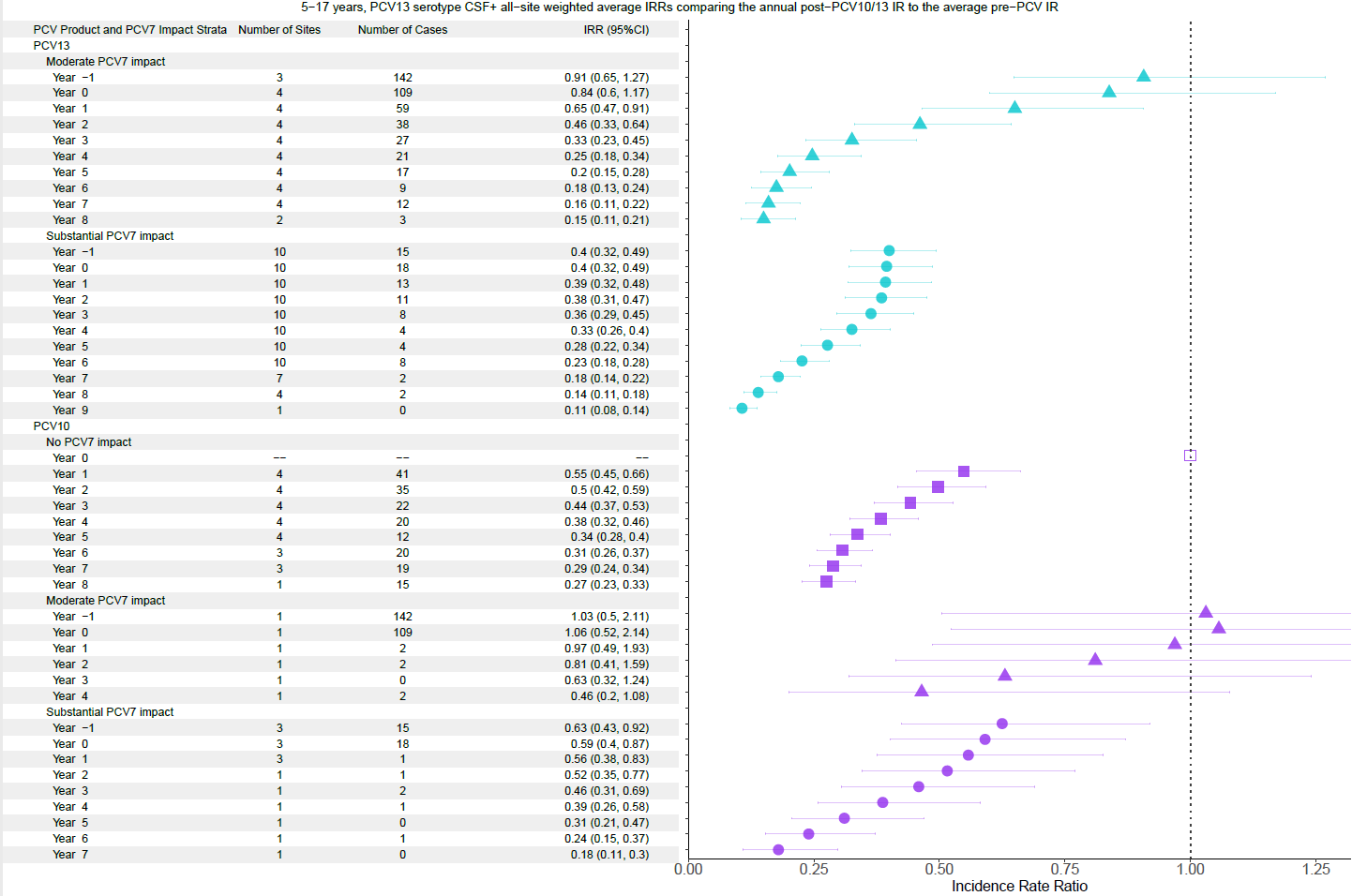


## Supplementary Figure 33. PCV13-type, adults >18 years.


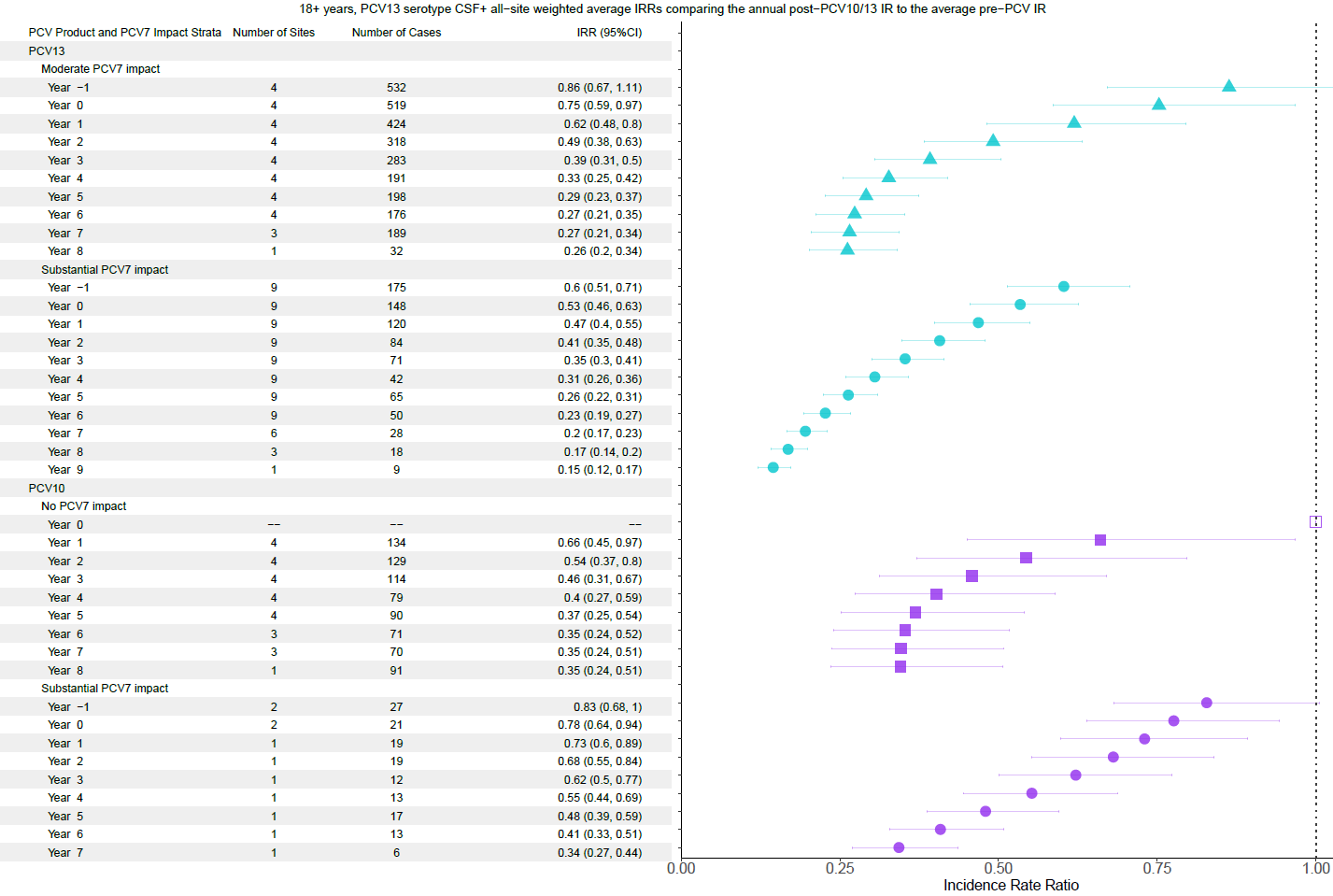


## Supplementary Figure 34. Non-PCV13 ST, children <5 years.


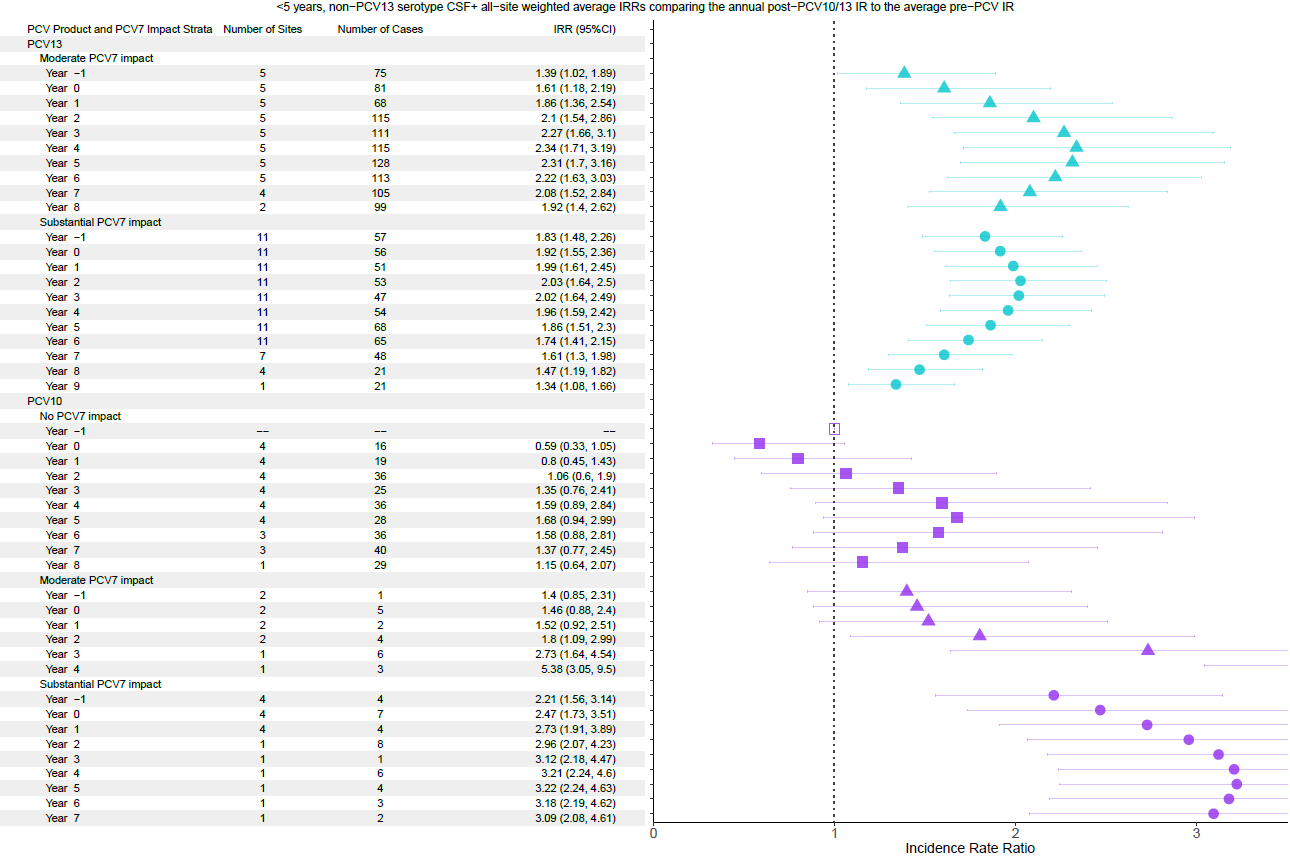


## Supplementary Figure 35. Non-PCV13 ST, children 5-17 years.


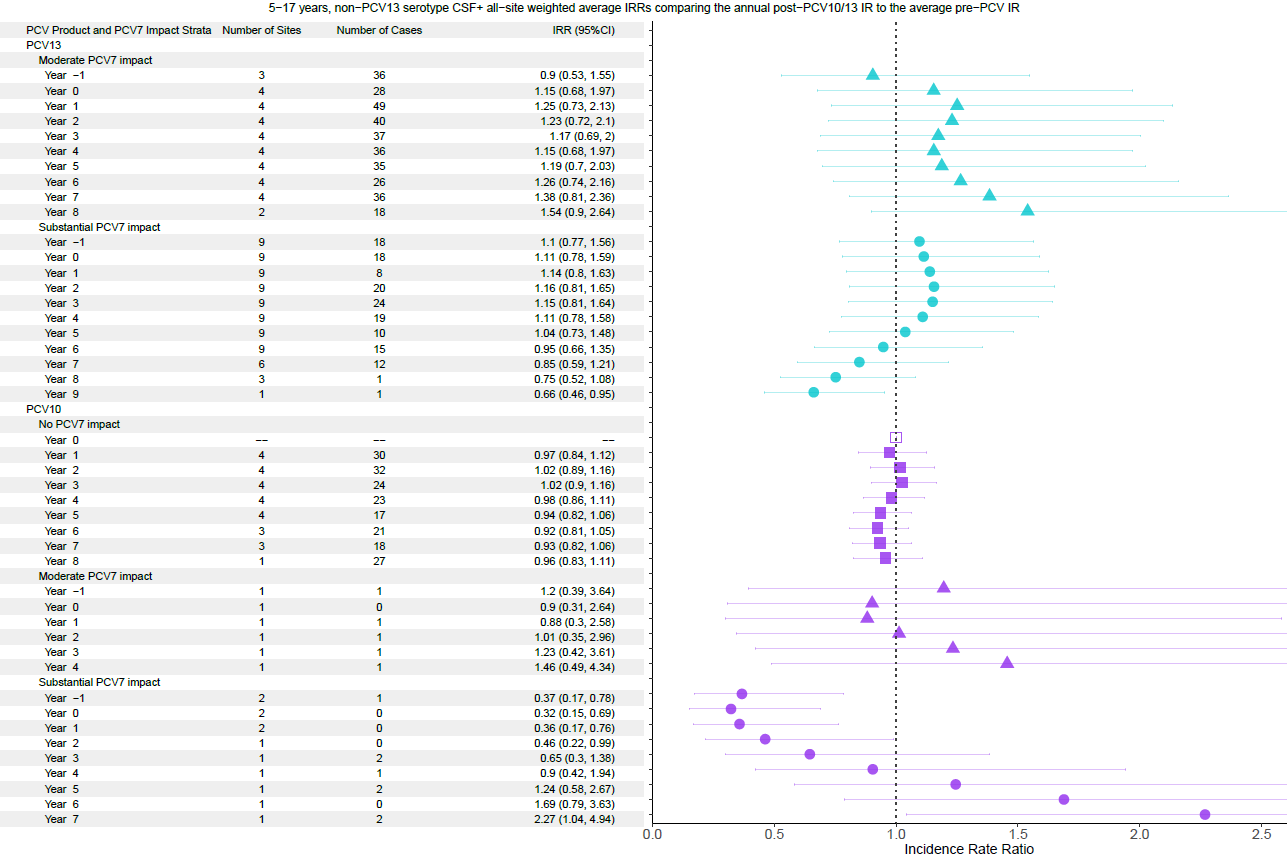


## Supplementary Figure 36. Non-PCV13 ST, adults >18 years.


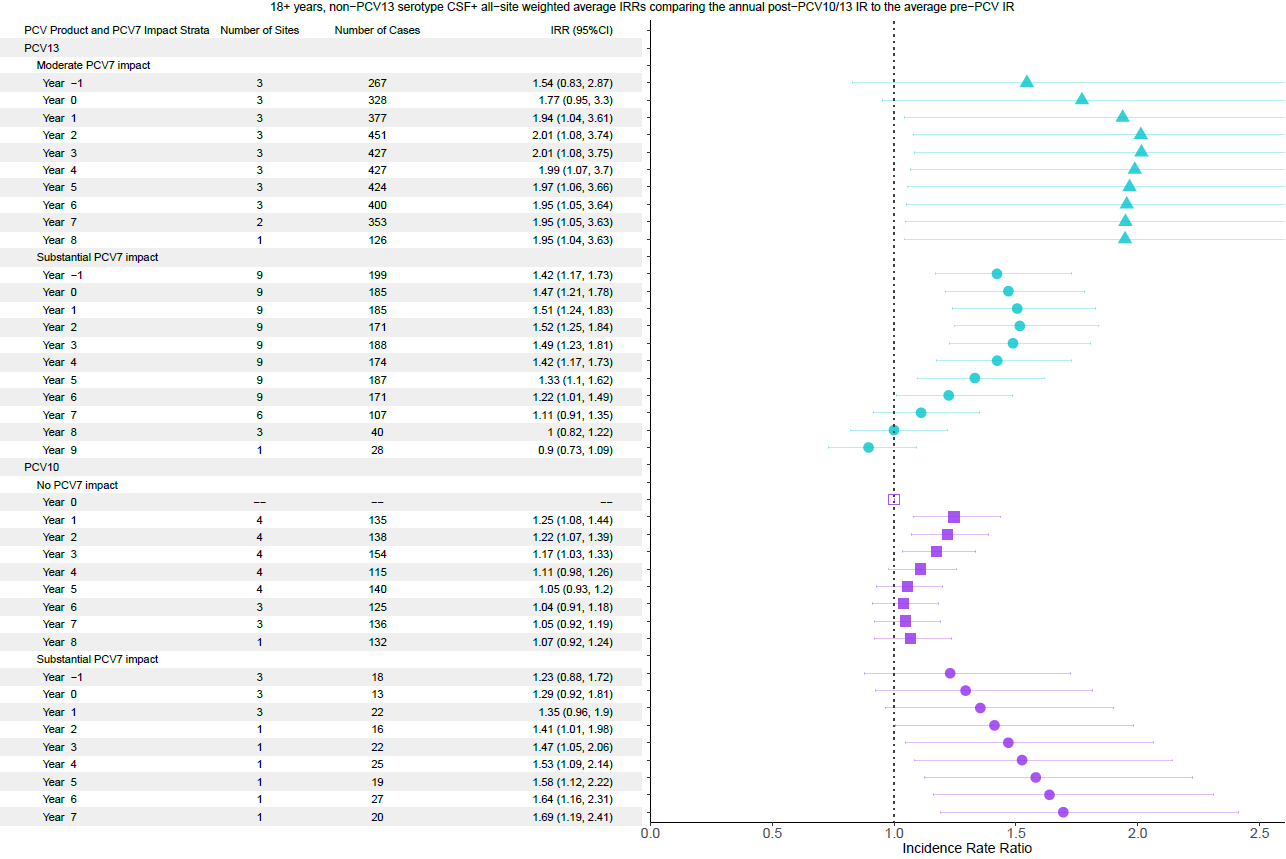


# Appendix 3: All-site CSF+ meningitis weighted average incidence rate ratios comparing the annual post-PCV10/13 incidence rate to the average pre-PCV incidence rate

## Supplementary Figure 37. All serotype with PCV13 use and substantial PCV7 impact for children <5 years.


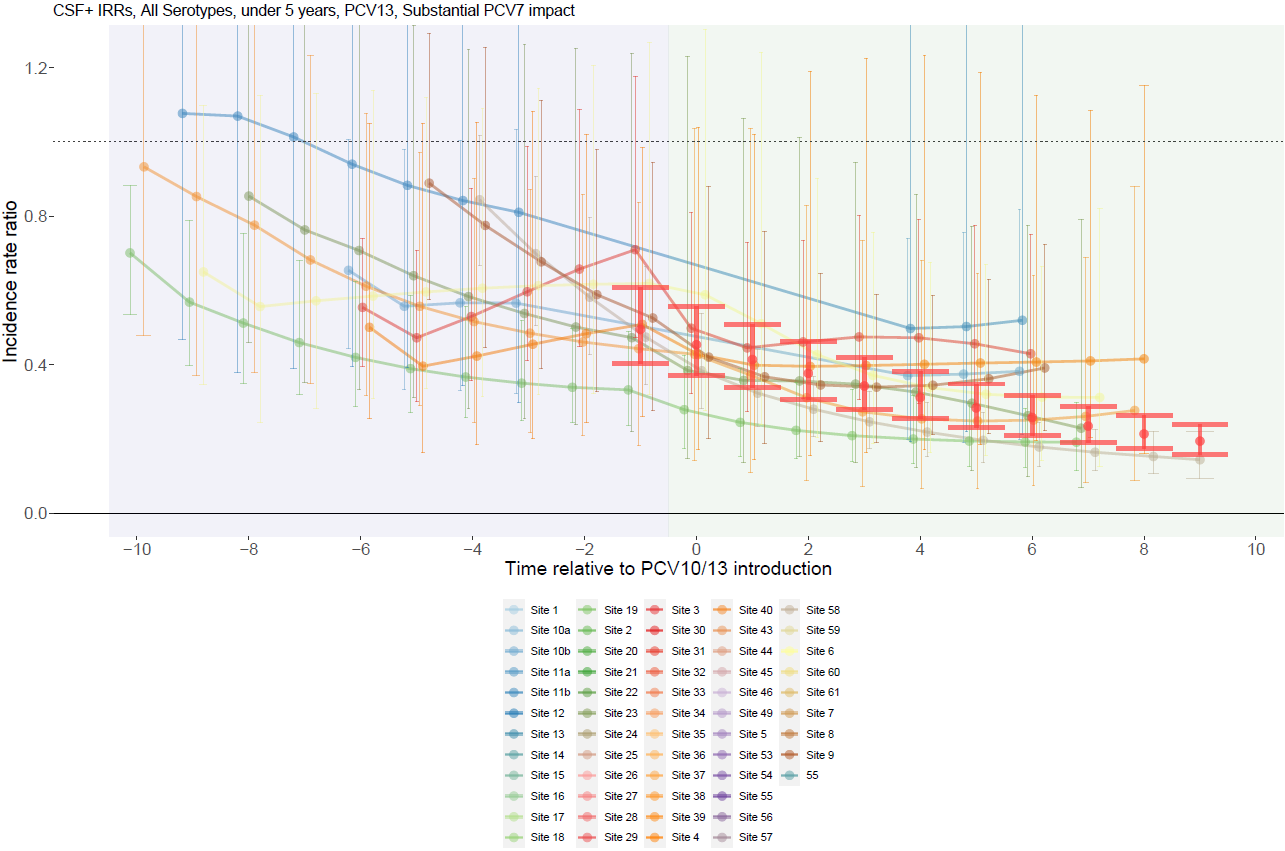


## Supplementary Figure 38. All serotype with PCV13 use and moderate PCV7 impact for children <5 years.


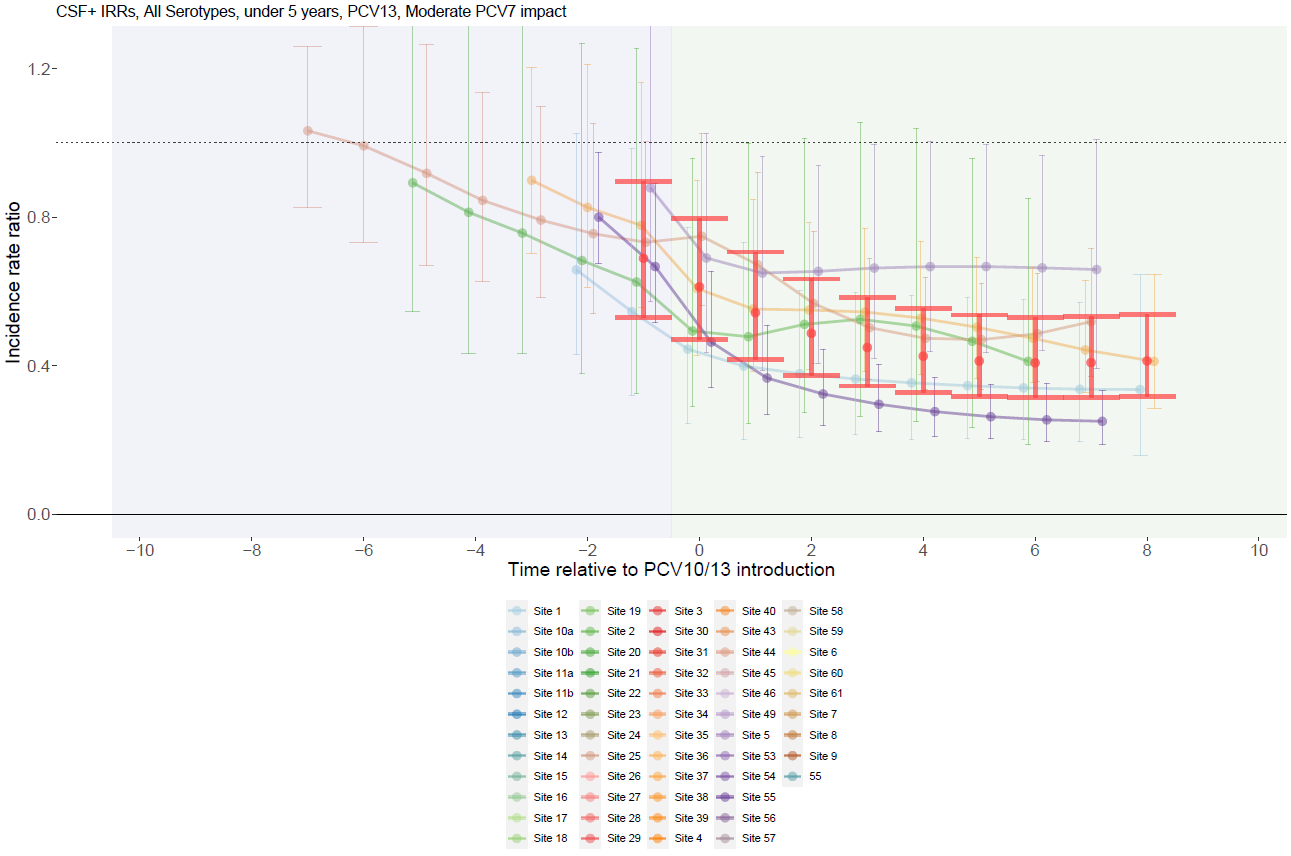


## Supplementary Figure 39. All serotype with PCV13 use and no PCV7 impact for children <5 years.


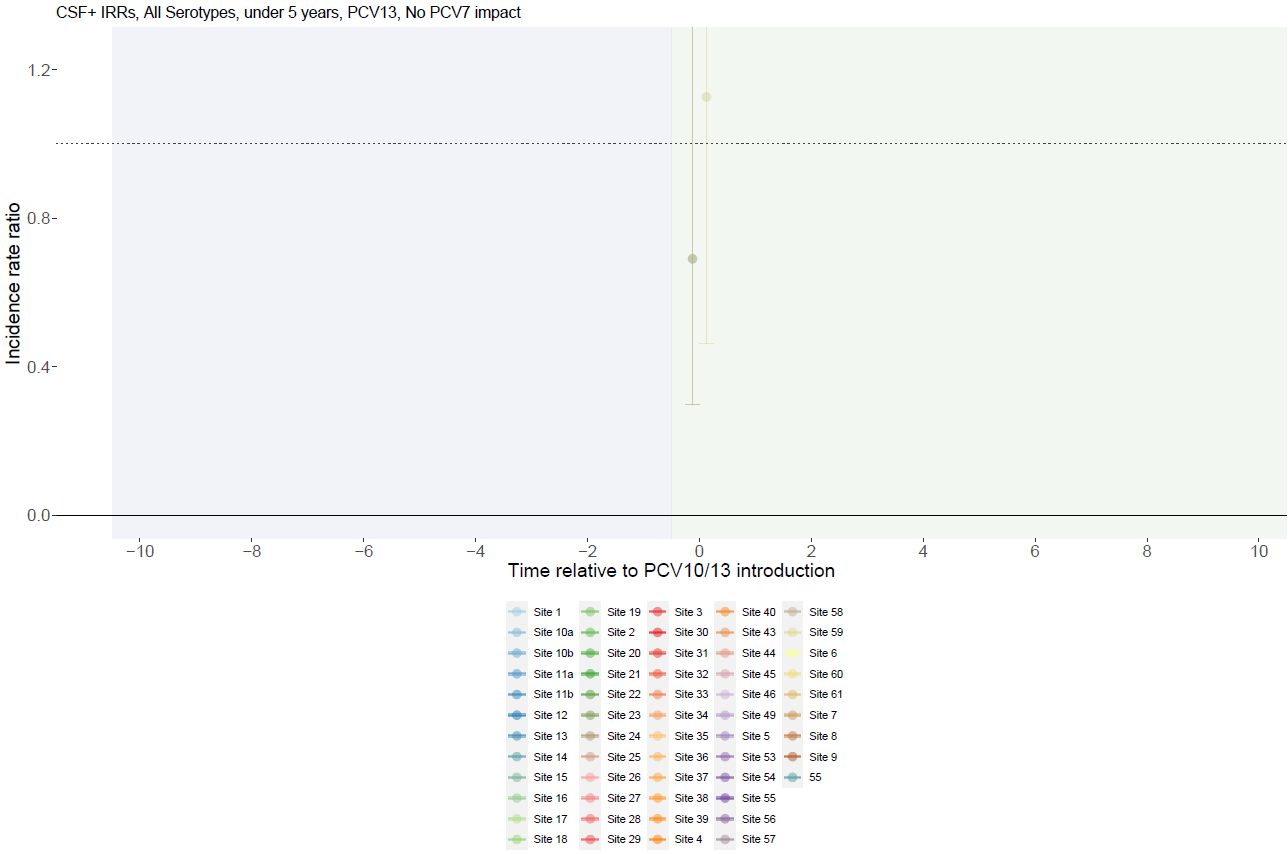


## Supplementary Figure 40. All serotype with PCV10 use and substantial PCV7 impact for children <5 years.


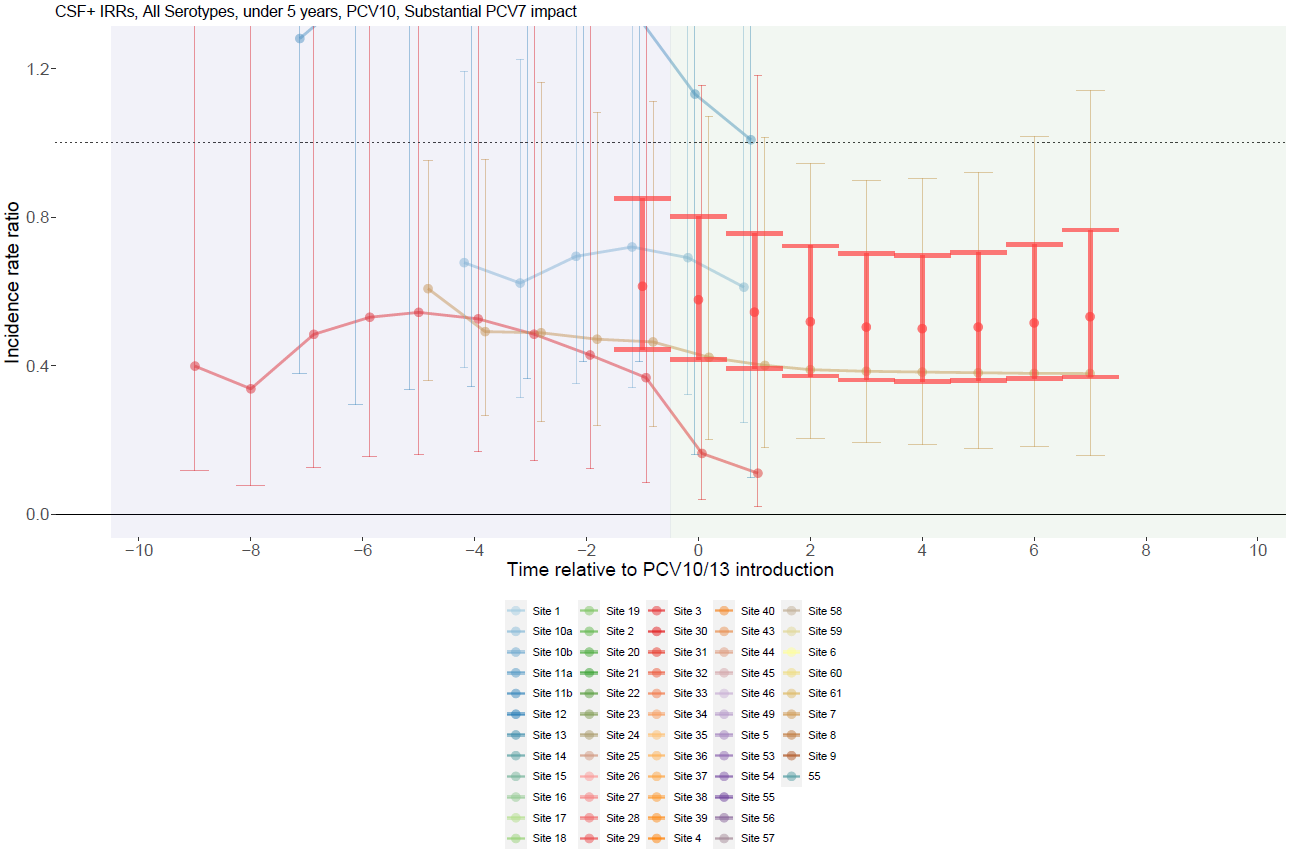


## Supplementary Figure 41. All serotype with PCV10 use and moderate PCV7 impact for children <5 years.


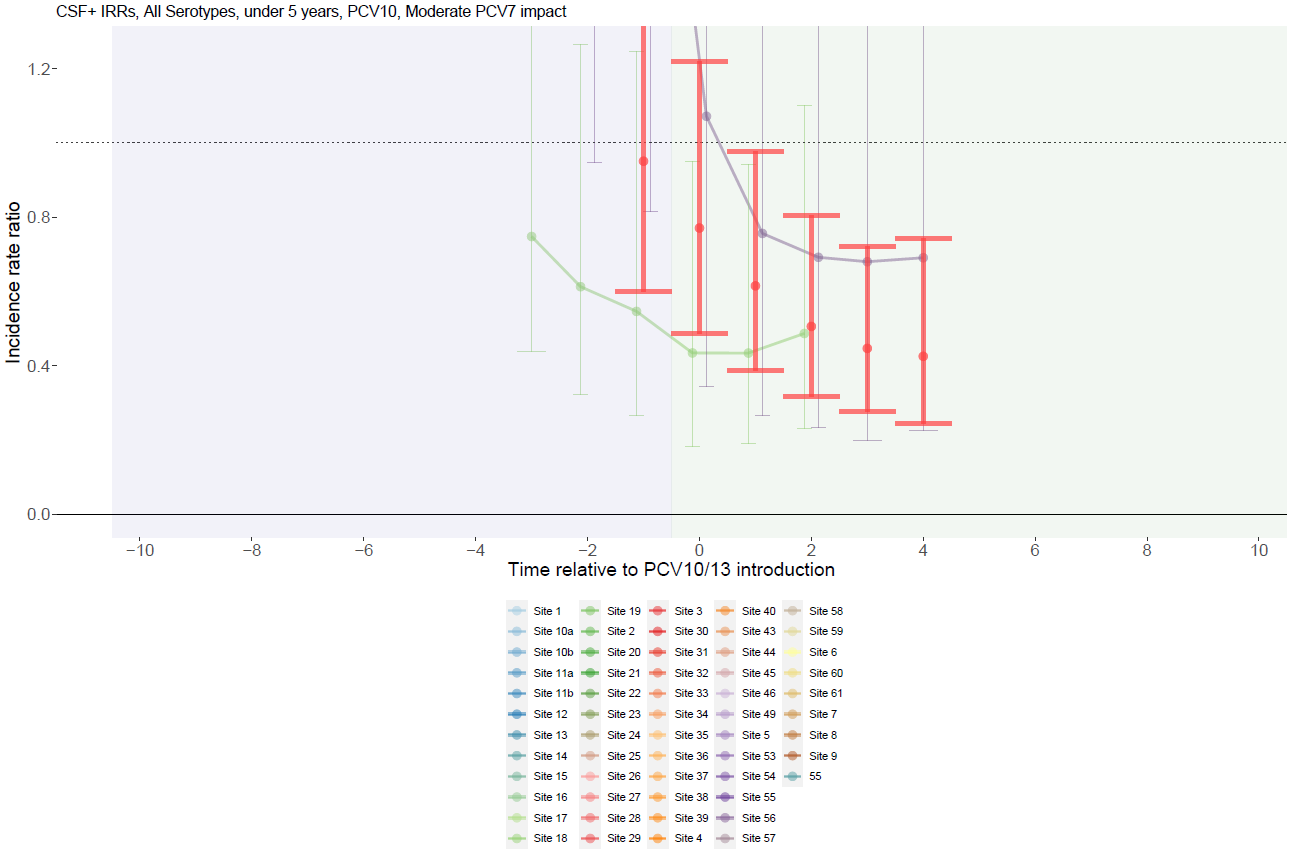


## Supplementary Figure 42. All serotype with PCV10 use and no PCV7 impact for children <5 years.


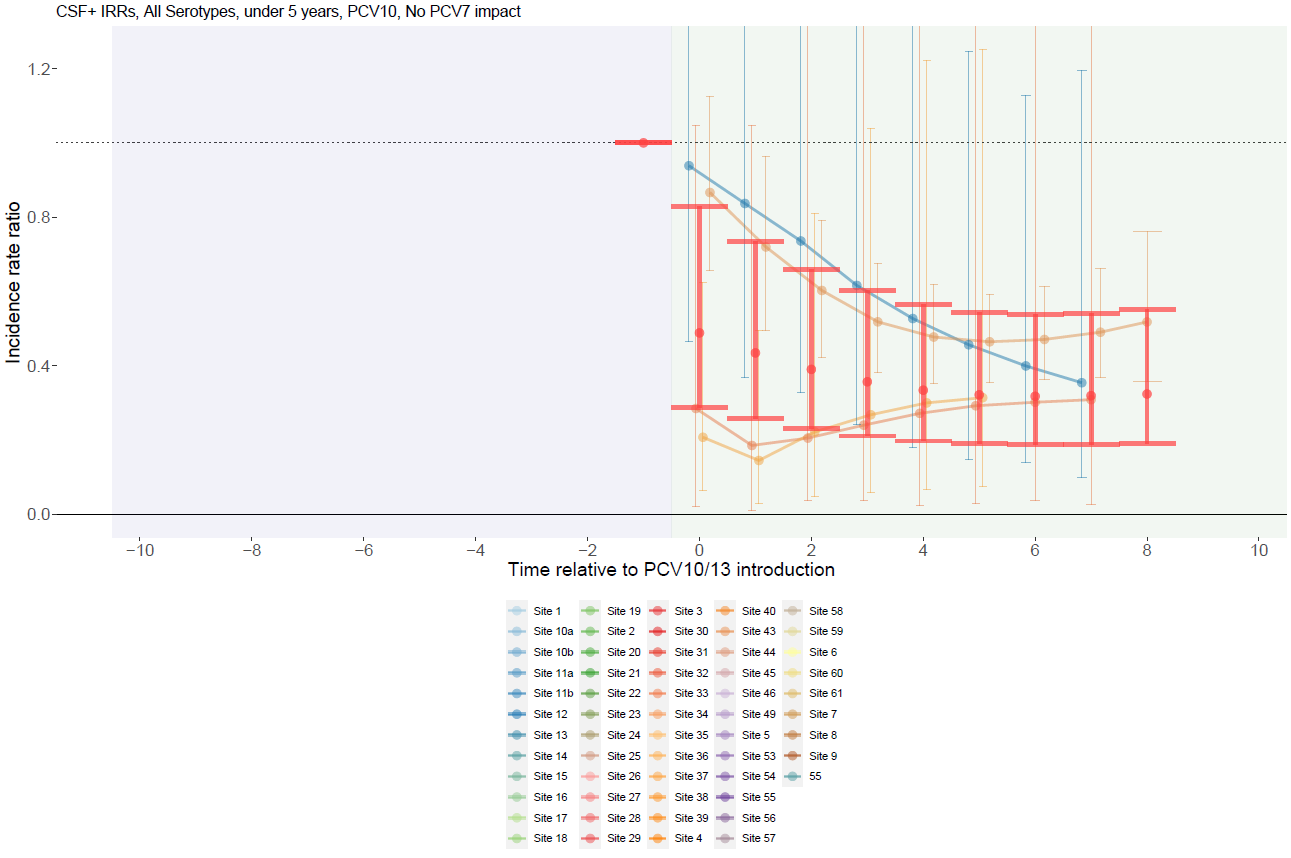


## Supplementary Figure 43. All serotype with PCV13 use and substantial PCV7 impact for individuals 5-17 years.


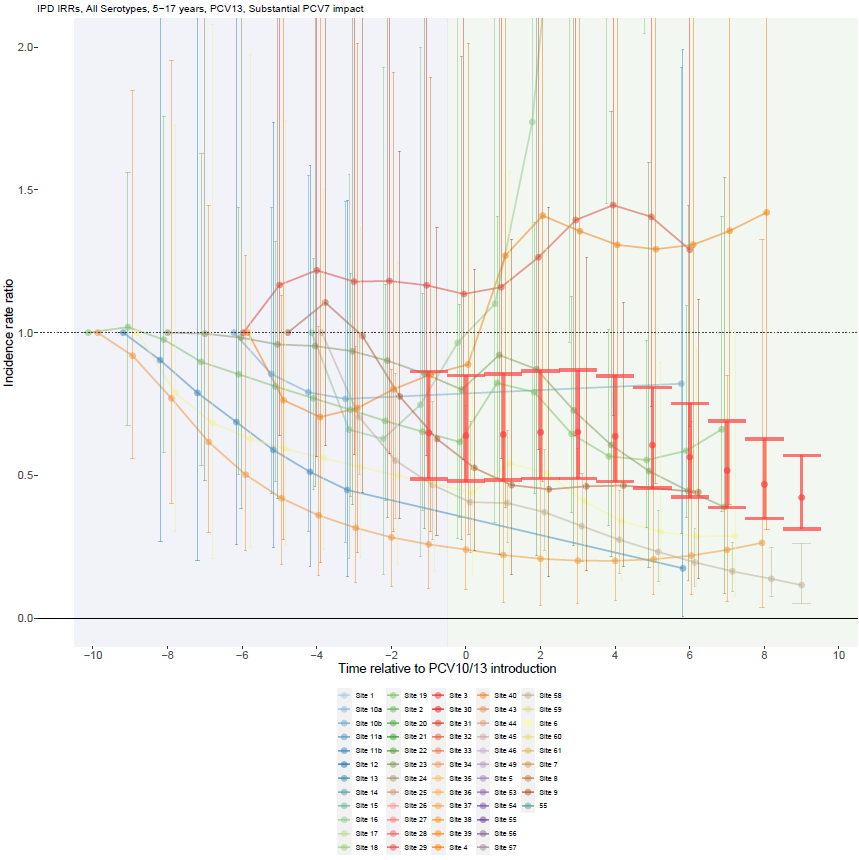


## Supplementary Figure 44. All serotype with PCV13 use and moderate PCV7 impact for individuals 5-17 years.


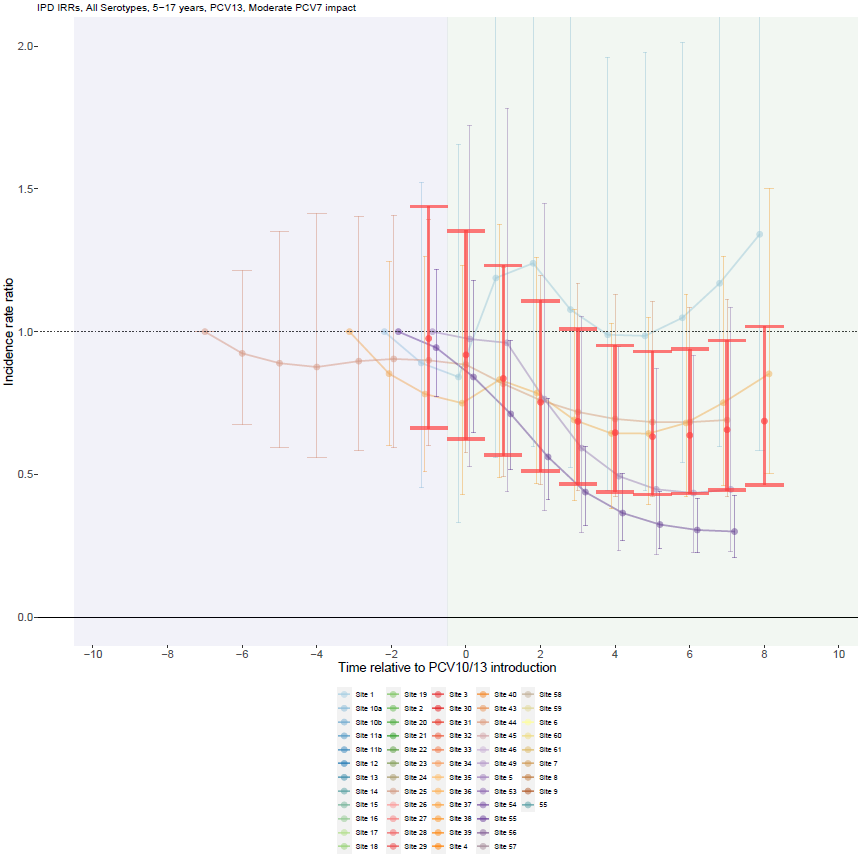


## Supplementary Figure 45. All serotype with PCV13 use and no PCV7 impact for individuals 5-17 years.


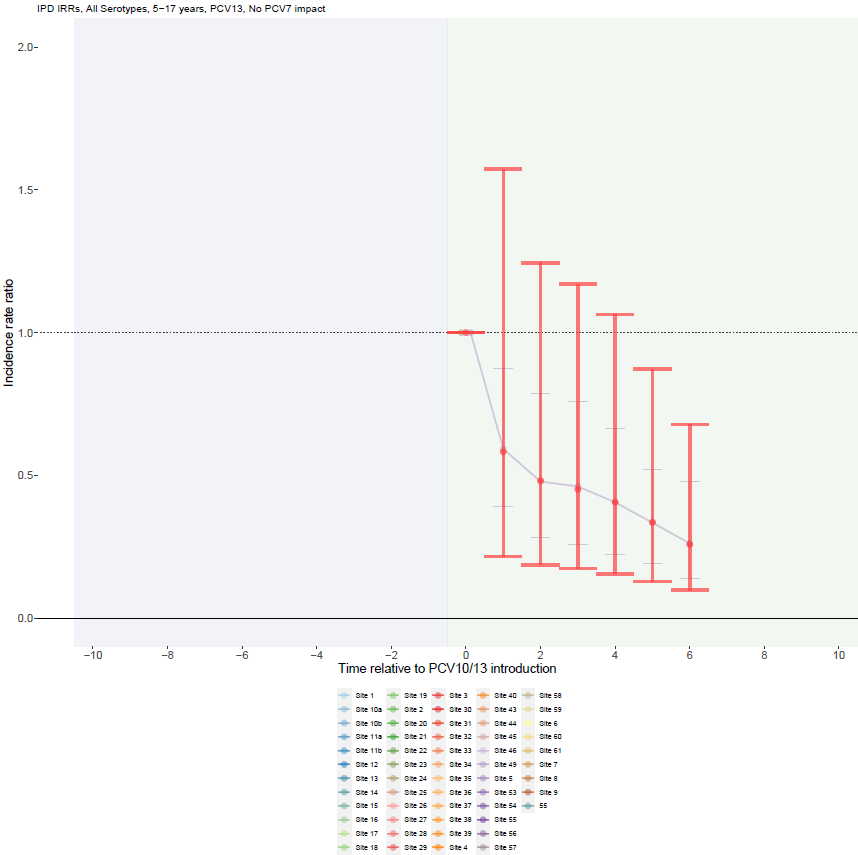


## Supplementary Figure 46. All serotype with PCV10 use and substantial PCV7 impact for individuals 5-17 years.


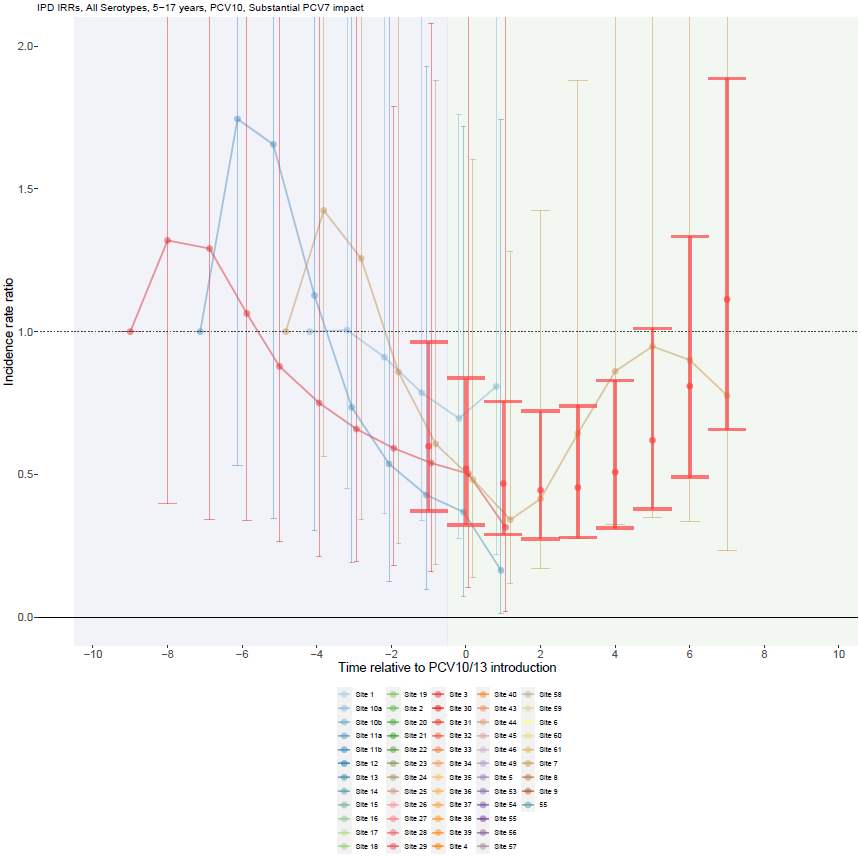


## Supplementary Figure 47. All serotype with PCV10 use and moderate PCV7 impact for individuals 5-17 years.


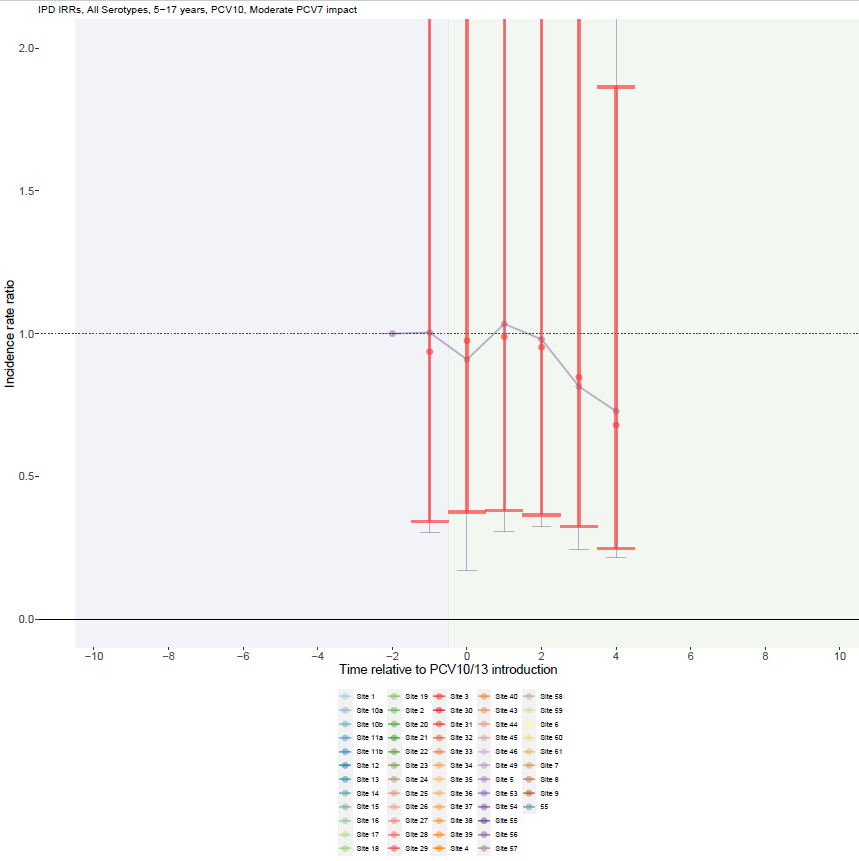


## Supplementary Figure 48. All serotype with PCV10 use and no PCV7 impact for individuals 5-17 years.


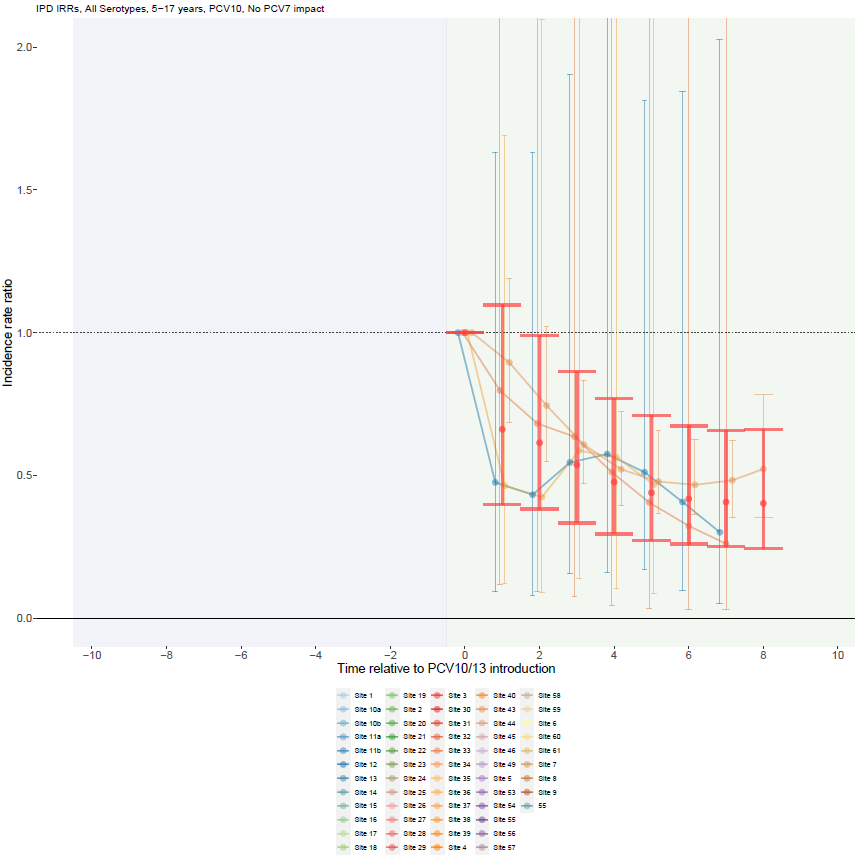


## Supplementary Figure 49. All serotype with PCV13 use and substantial PCV7 impact for adults >18 years.


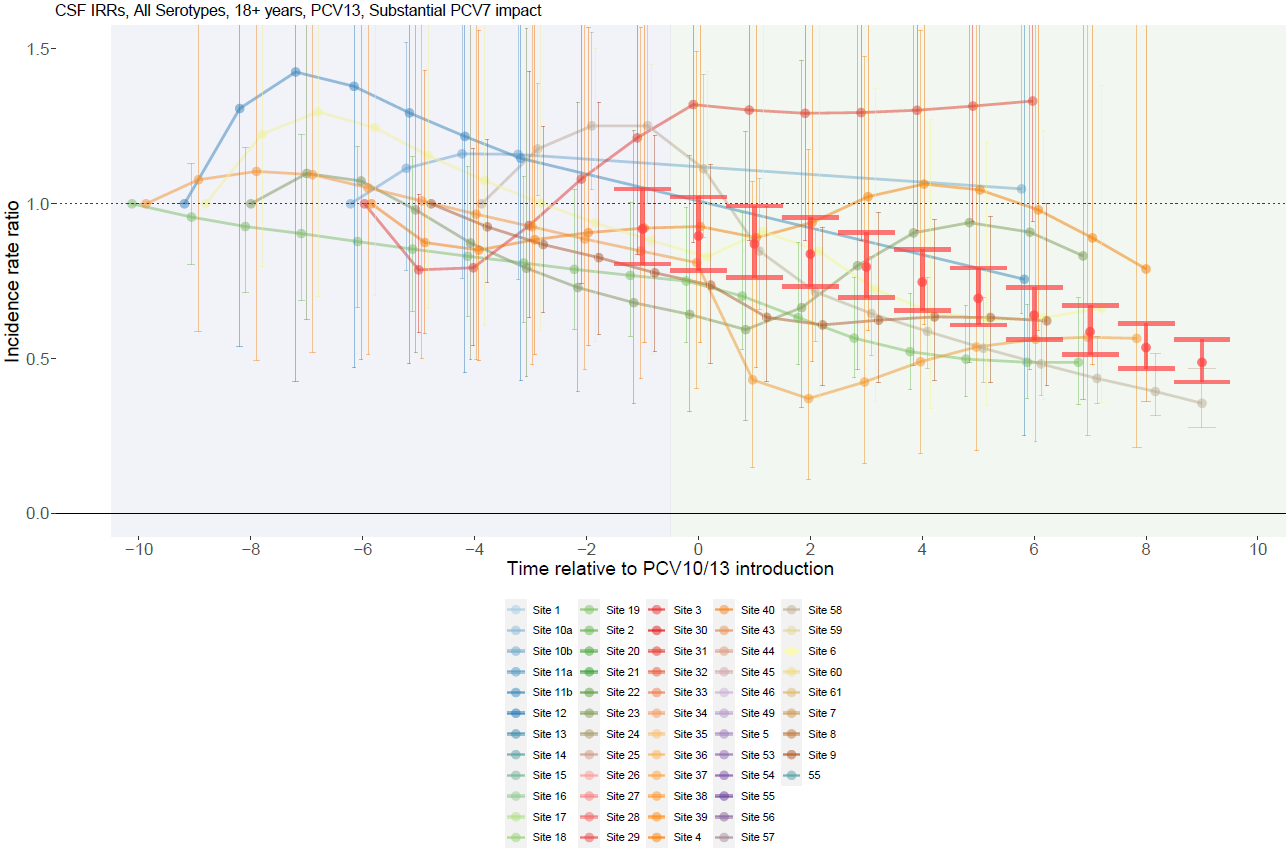


## Supplementary Figure 50. All serotype with PCV13 use and moderate PCV7 impact for adults >18 years.


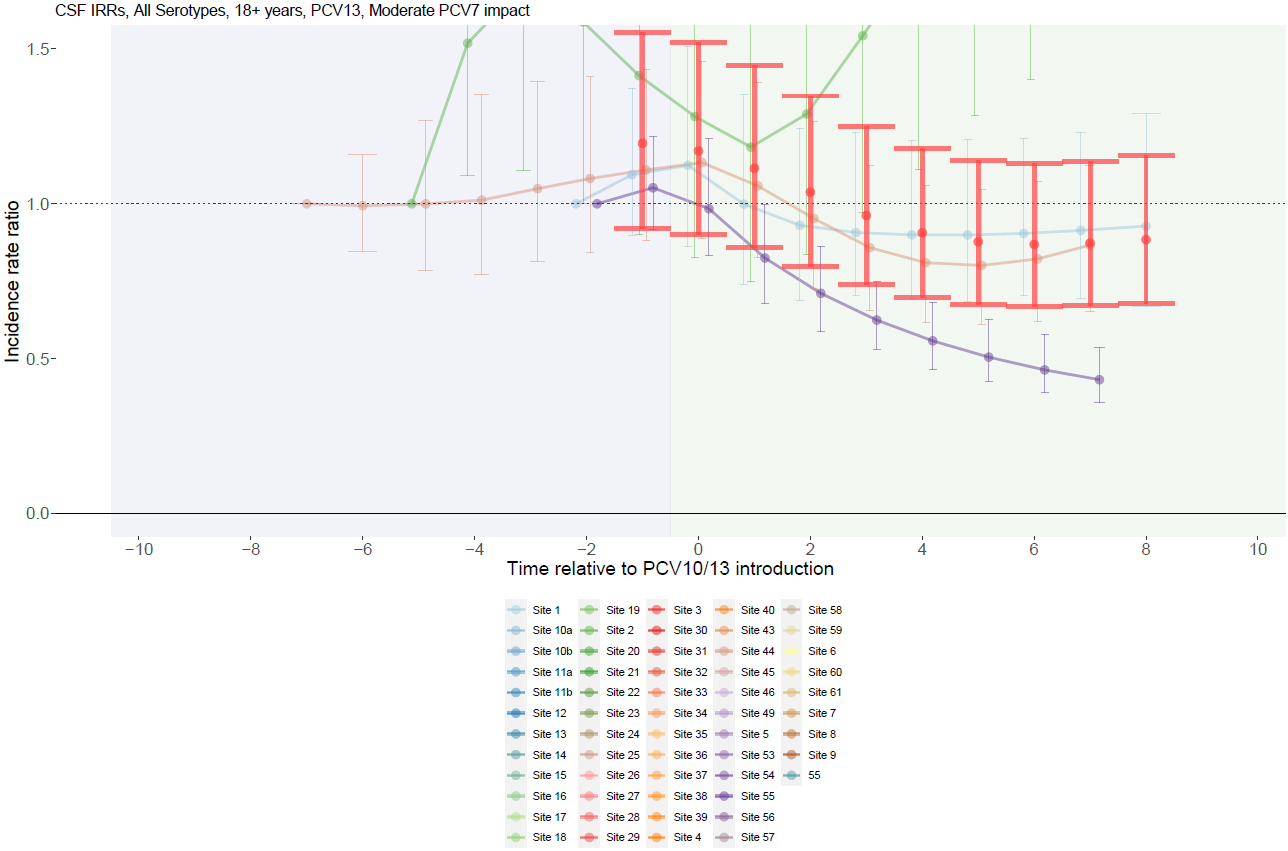


## Supplementary Figure 51. All serotype with PCV10 use and substantial PCV7 impact for adults >18 years.


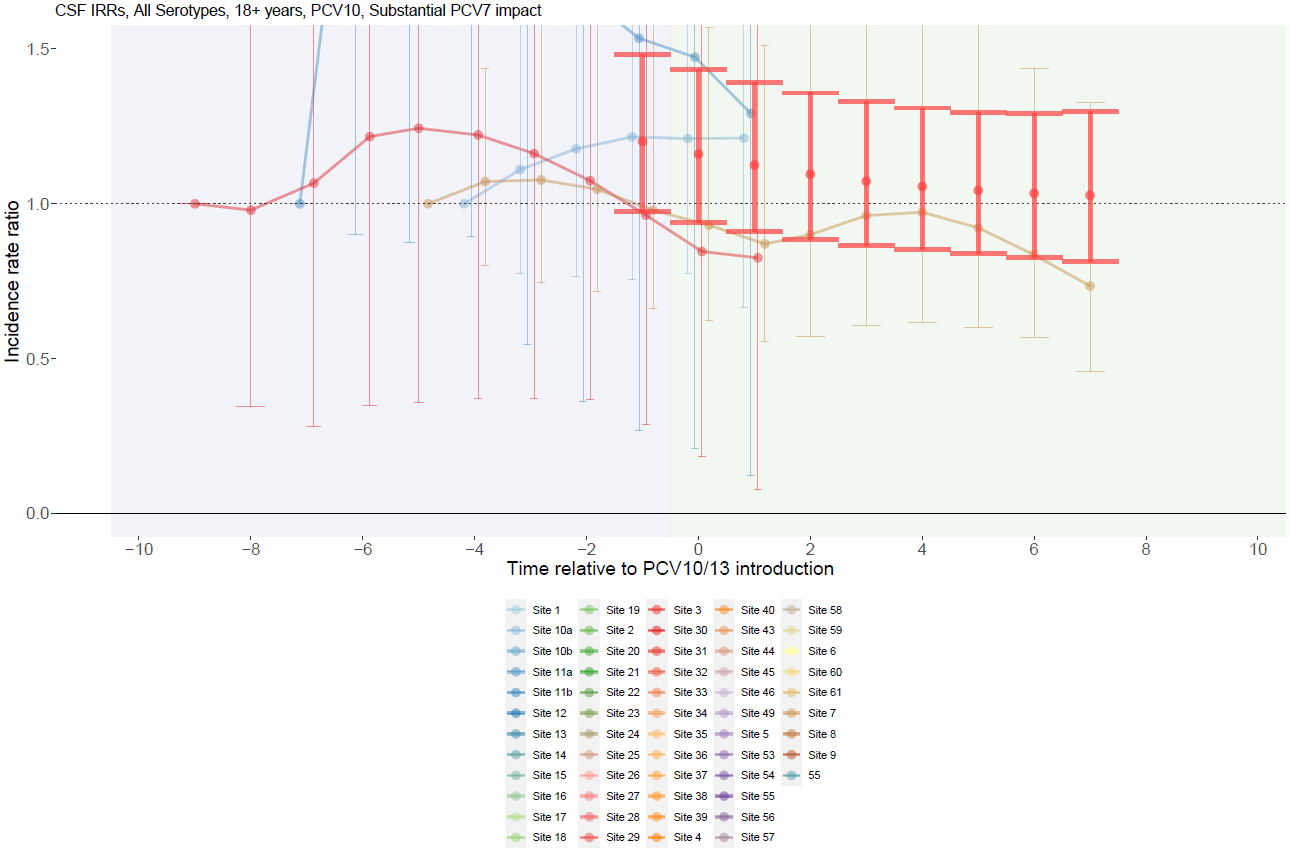


## Supplementary Figure 52. All serotype with PCV13 use and no PCV7 impact for adults >18 years.


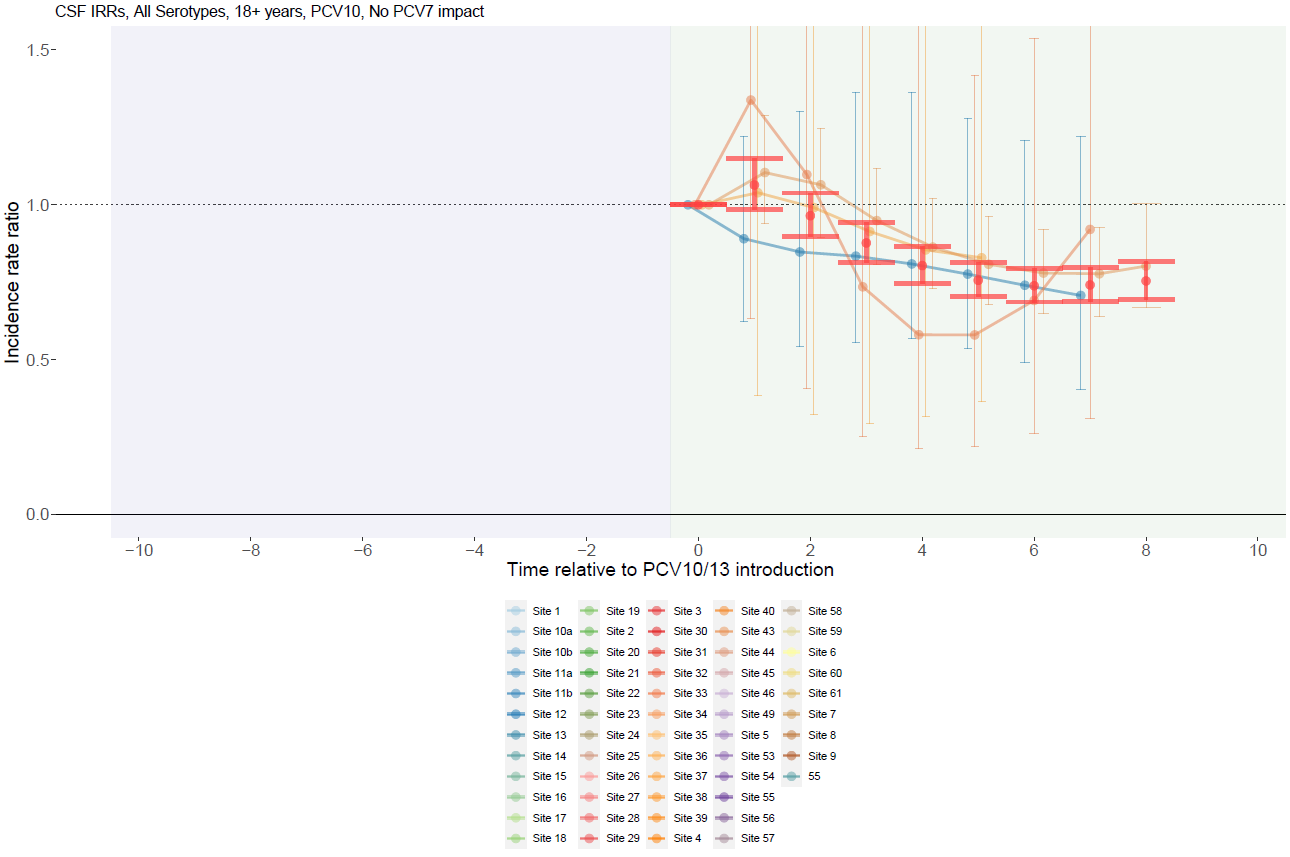


## Supplementary Figure 53. PCV7-type with PCV13 use and substantial PCV7 impact for children <5 years.


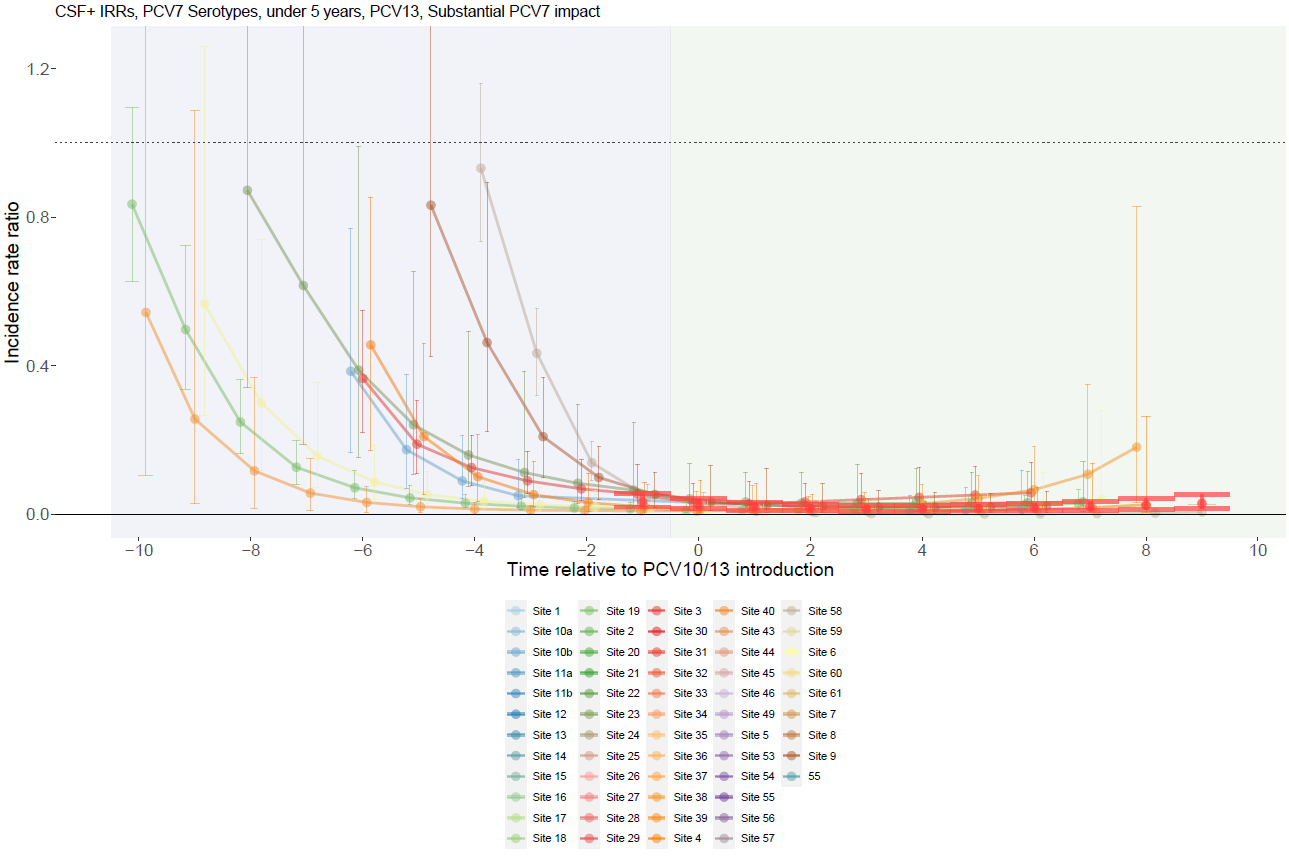


## Supplementary Figure 54. PCV7-type with PCV13 use and moderate PCV7 impact for children <5 years.


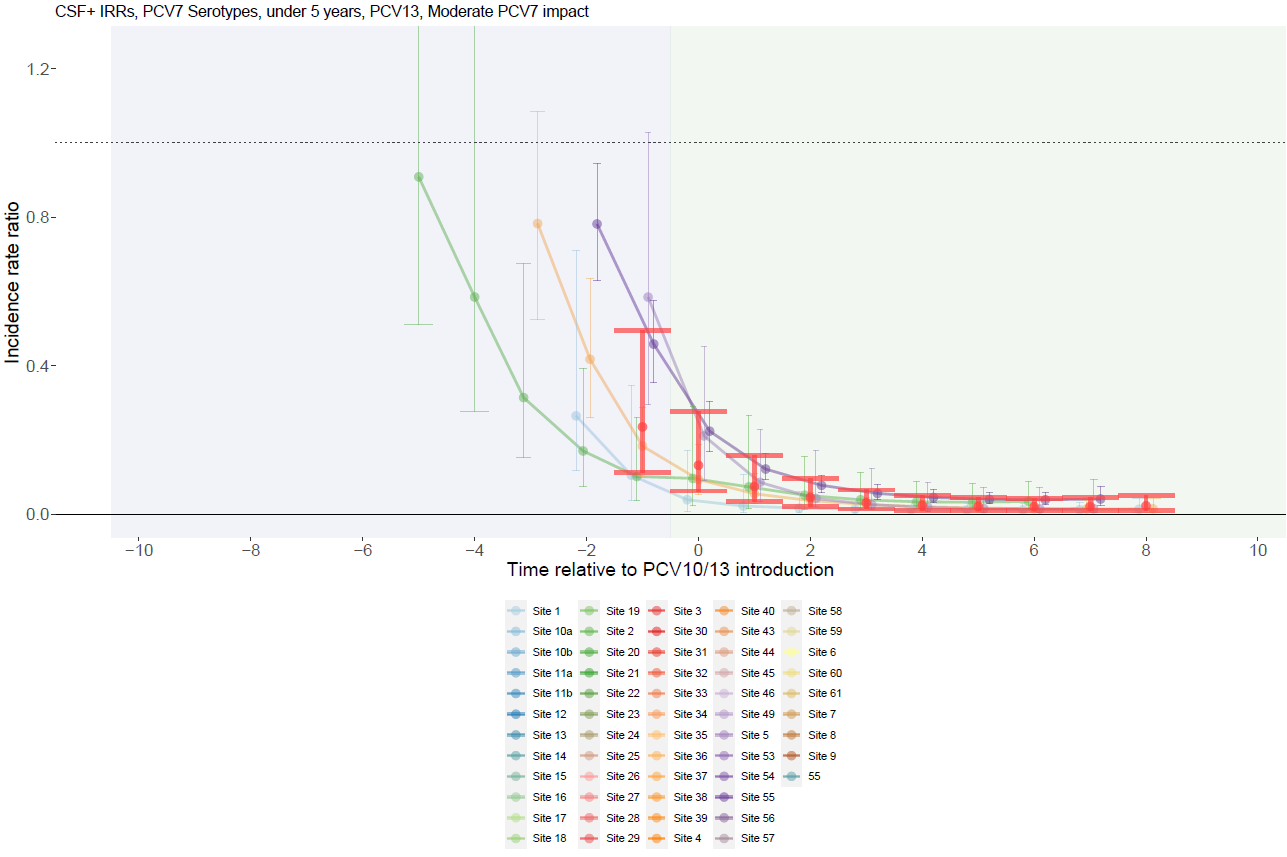


## Supplementary Figure 55. PCV7-type with PCV13 use and no PCV7 impact for children <5 years.


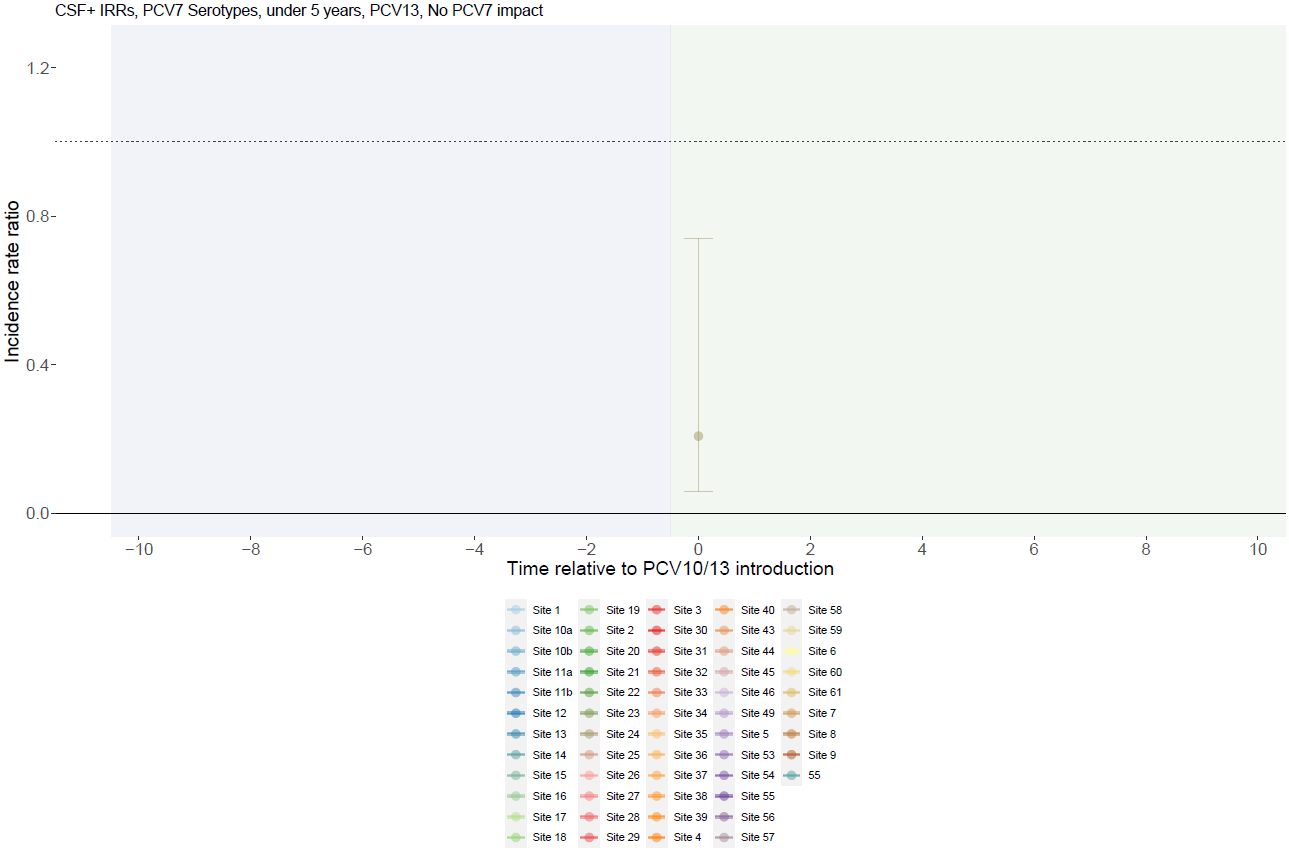


## Supplementary Figure 56. PCV7-type with PCV10 use and substantial PCV7 impact for children <5 years.


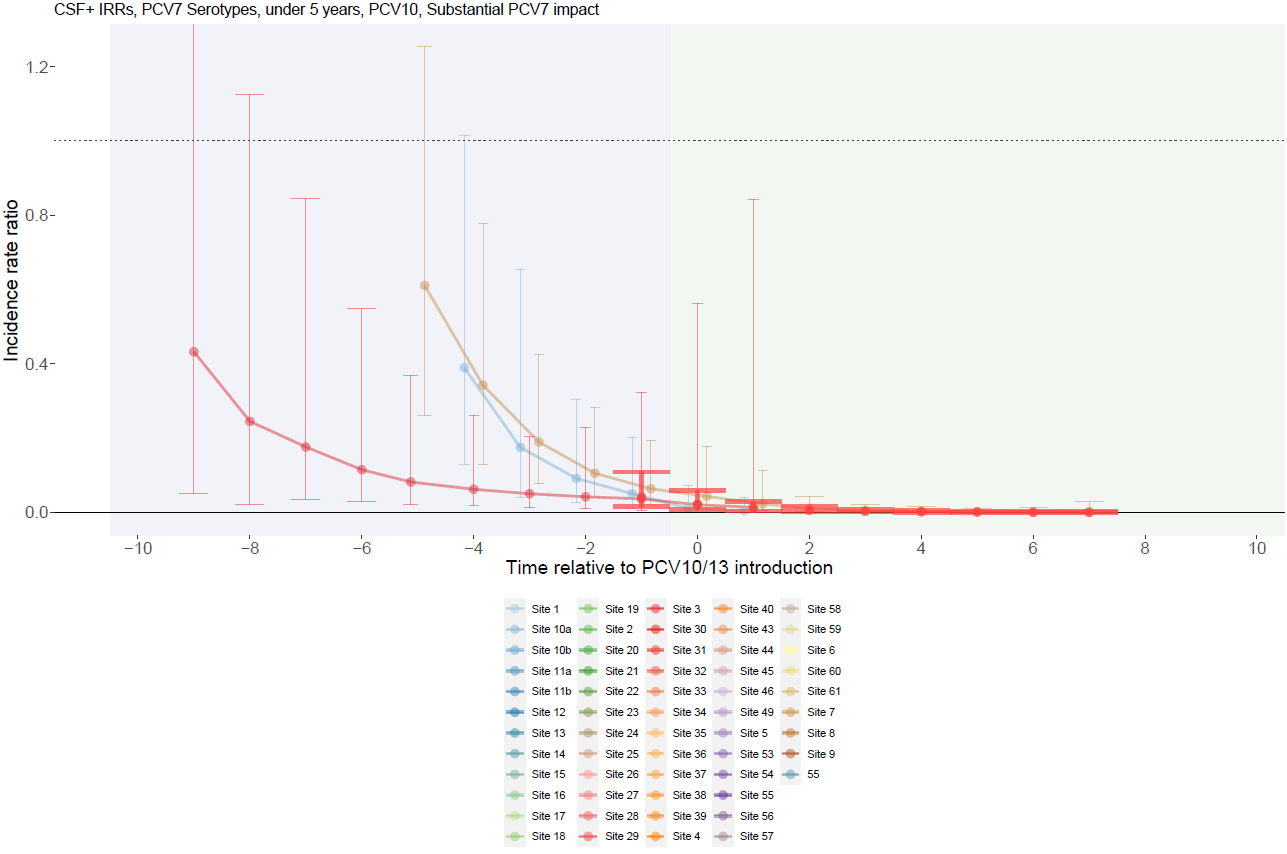


## Supplementary Figure 57. PCV7-type with PCV10 use and moderate PCV7 impact for children <5 years.


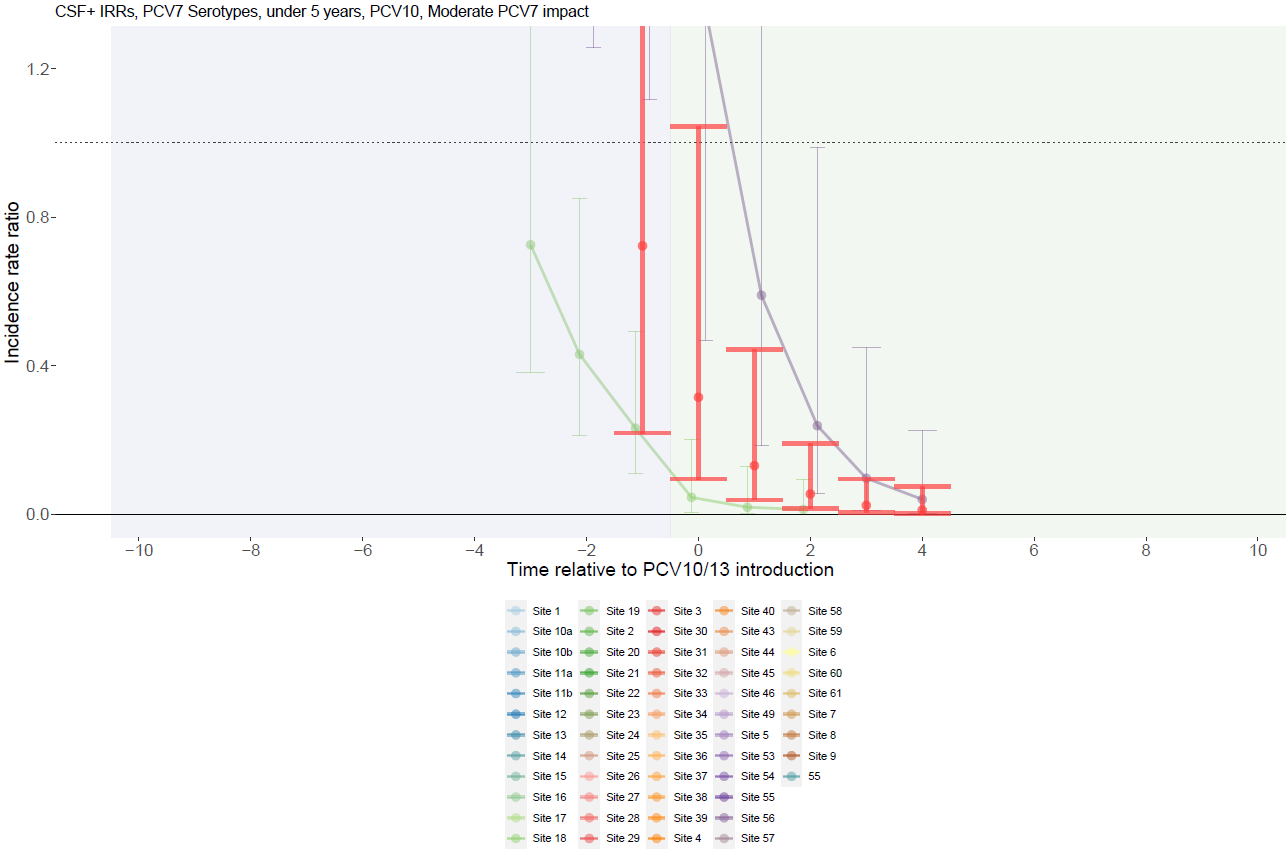


## Supplementary Figure 58. PCV7-type with PCV10 use and no PCV7 impact for children <5 years.


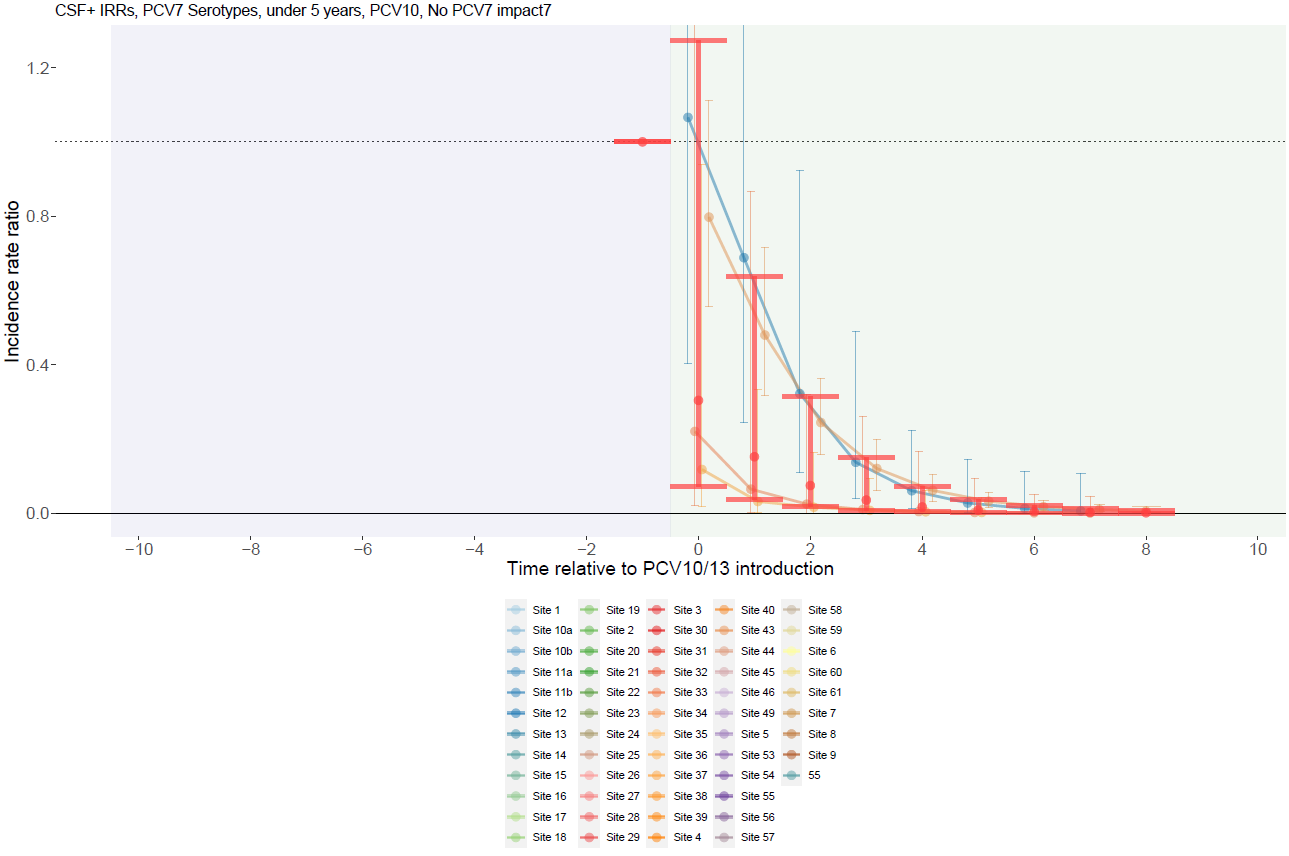


## Supplementary Figure 59. PCV7-type with PCV13 use and substantial PCV7 impact for individuals 5-17 years.


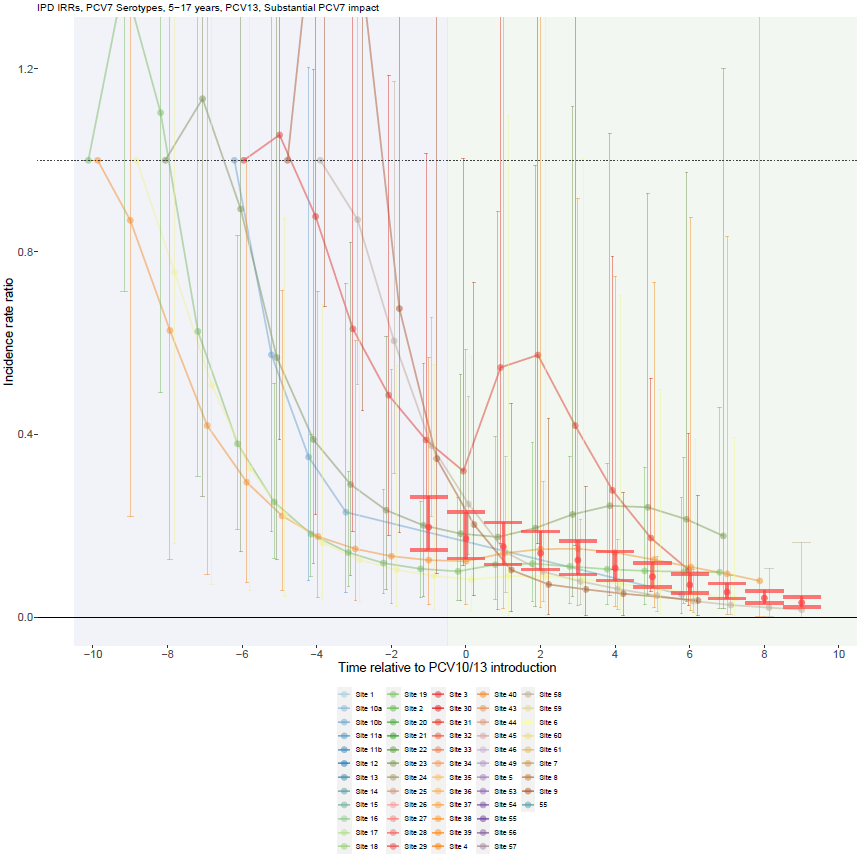


## Supplementary Figure 60. PCV7-type with PCV13 use and moderate PCV7 impact for individuals 5-17 years.


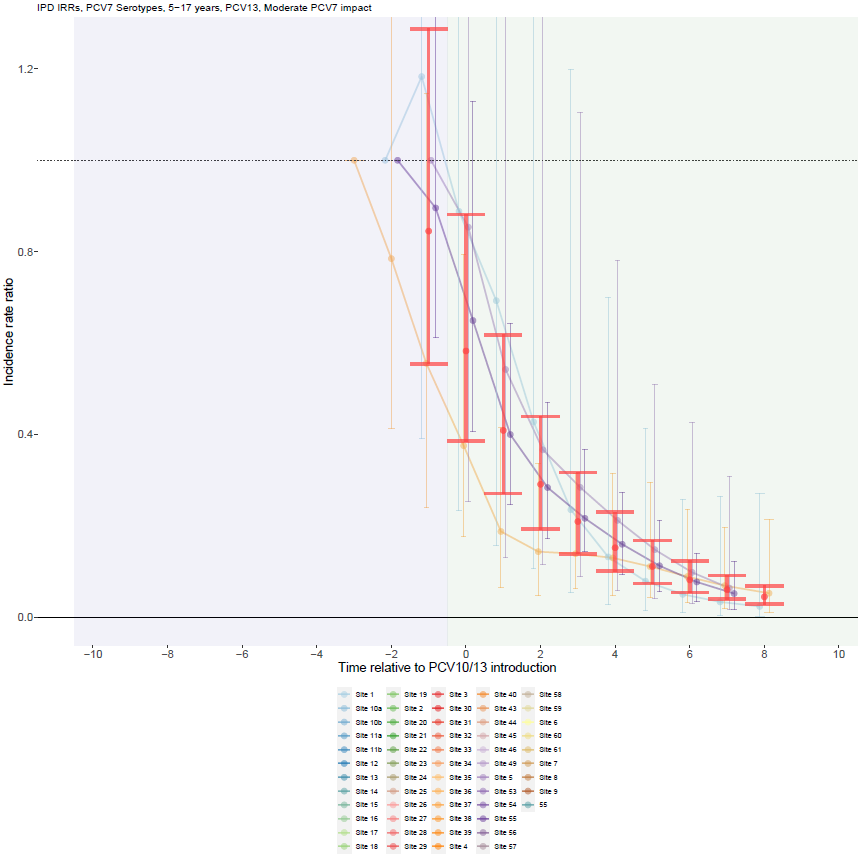


## Supplementary Figure 61. PCV7-type with PCV13 use and no PCV7 impact for individuals 5-17 years.


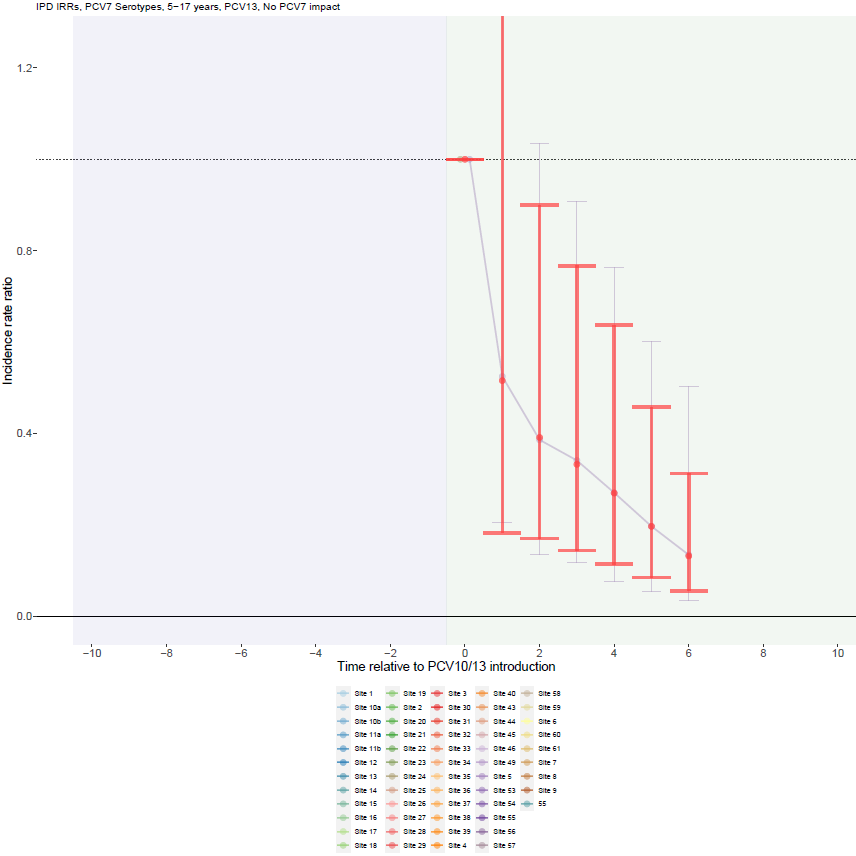


## Supplementary Figure 62. PCV7-type with PCV10 use and substantial PCV7 impact for individuals 5-17 years.


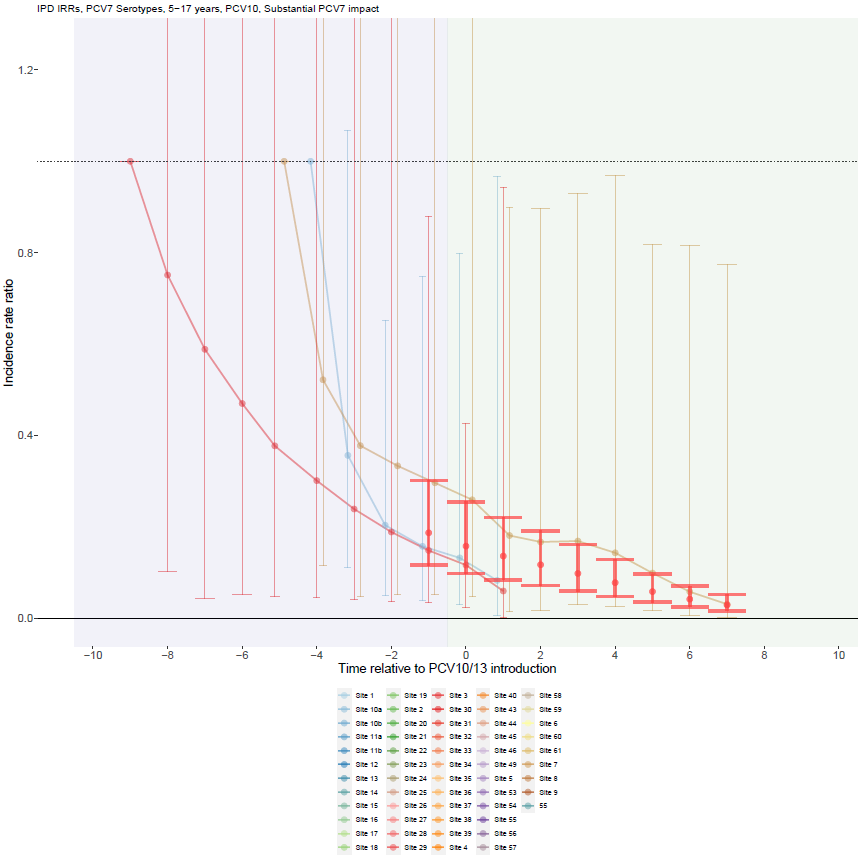


## Supplementary Figure 63. PCV7-type with PCV10 use and moderate PCV7 impact for individuals 5-17 years.


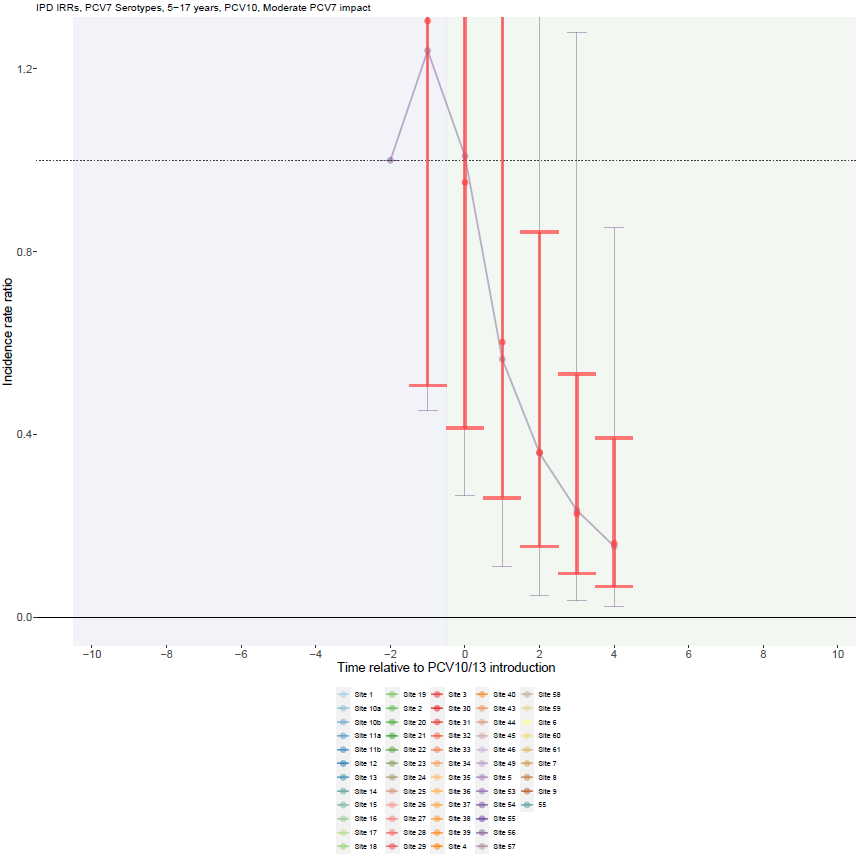


## Supplementary Figure 64. PCV7-type with PCV10 use and no PCV7 impact for individuals 5-17 years.


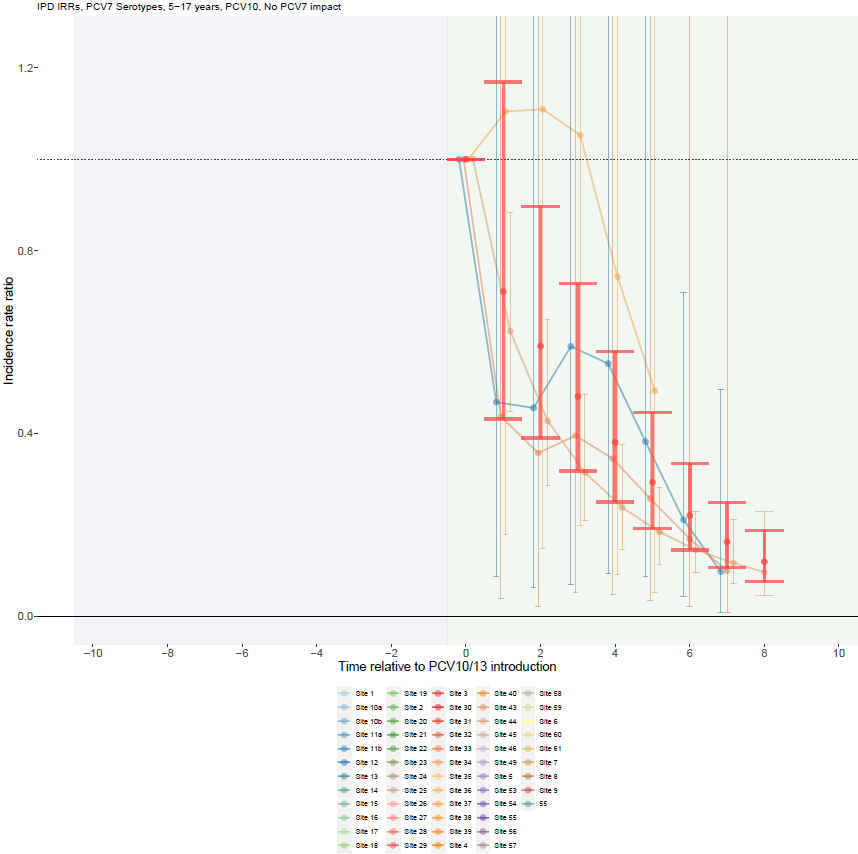


## Supplementary Figure 65. PCV7-type with PCV13 use and substantial PCV7 impact for adults >18 years.


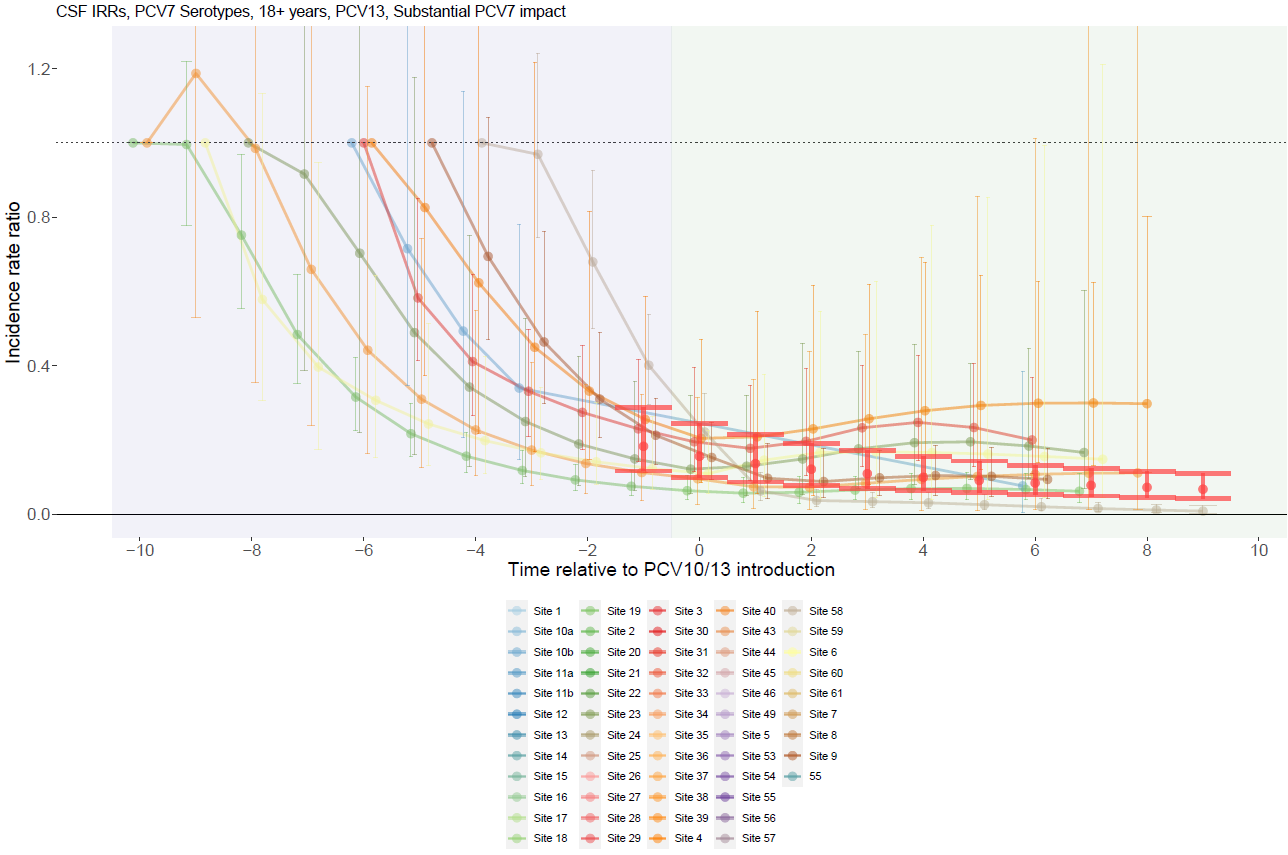


## Supplementary Figure 66. PCV7-type with PCV13 use and moderate PCV7 impact for adults >18 years.


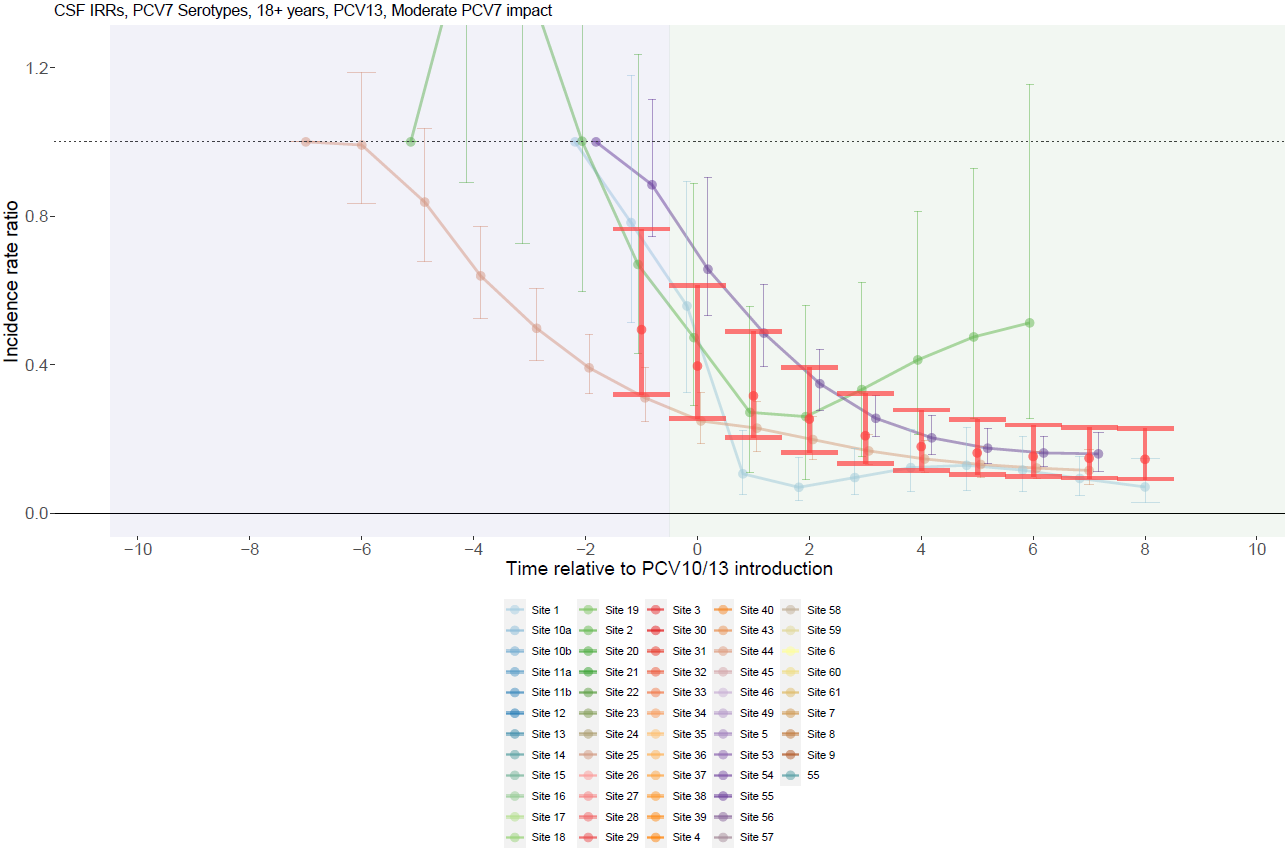


## Supplementary Figure 67. PCV7-type with PCV10 use and substantial PCV7 impact for adults >18 years.


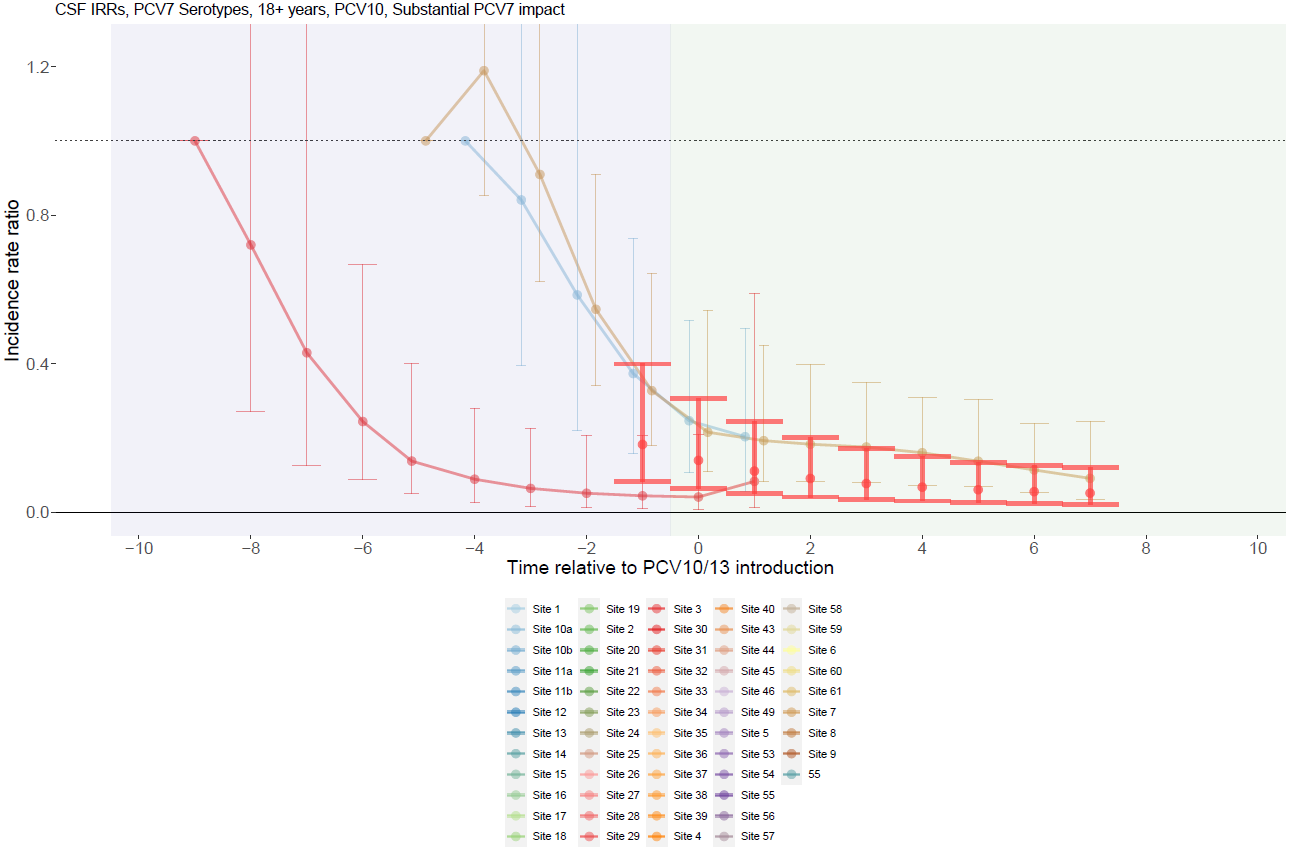


## Supplementary Figure 68. PCV7-type with PCV10 use and no PCV7 impact for adults >18 years.


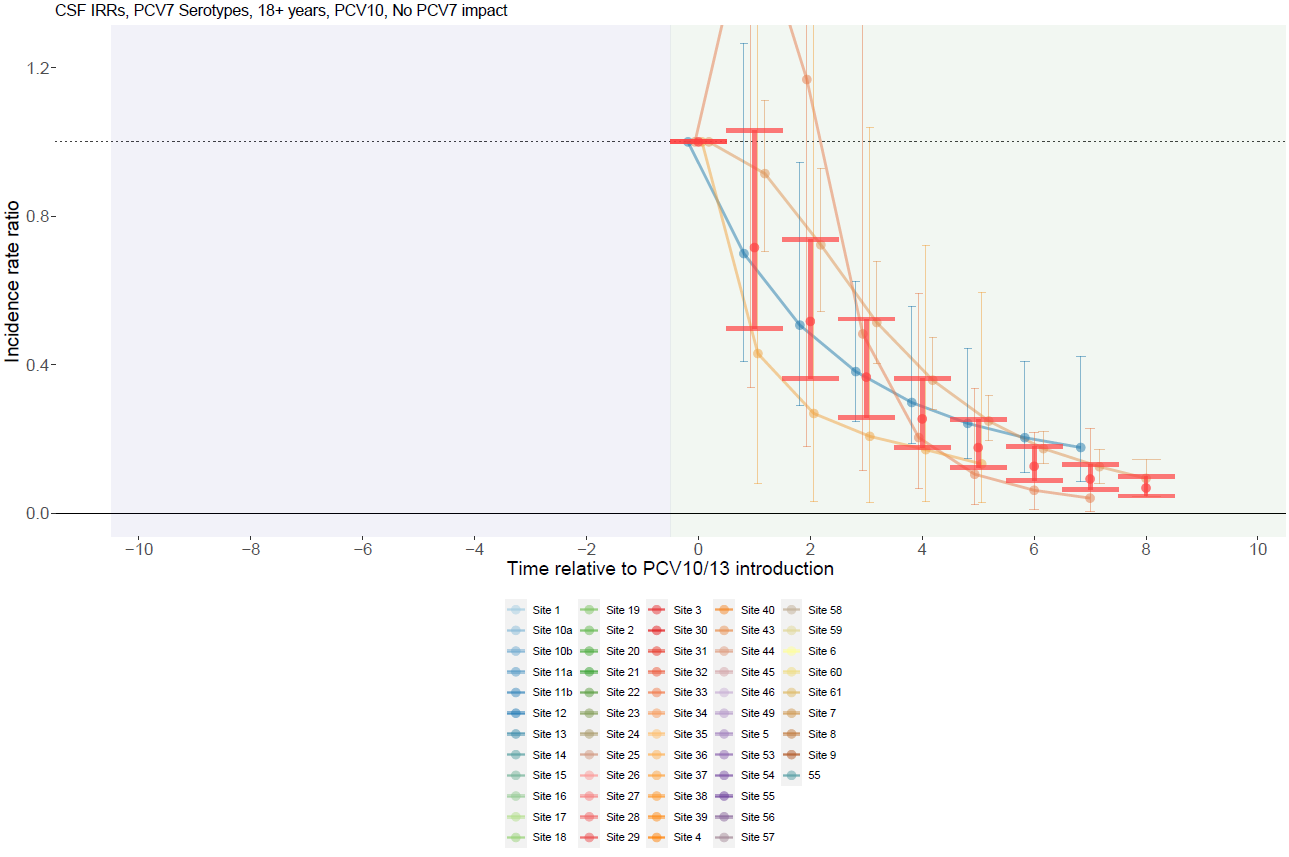


## Supplementary Figure 69. ST1, 5, 7F with PCV13 use and substantial PCV7 impact for children <5 years.


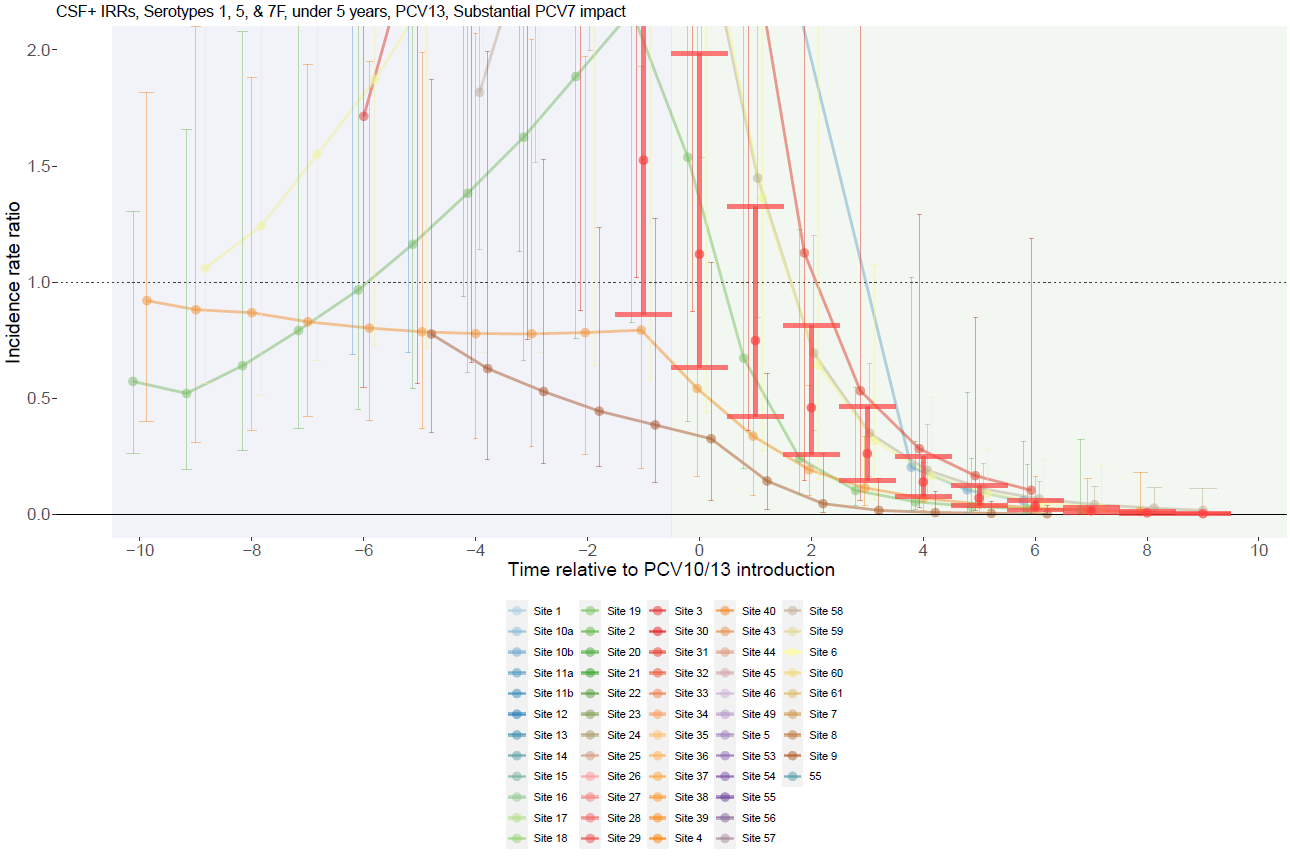


## Supplementary Figure 70. ST1, 5, 7F with PCV13 use and moderate PCV7 impact for children <5 years.


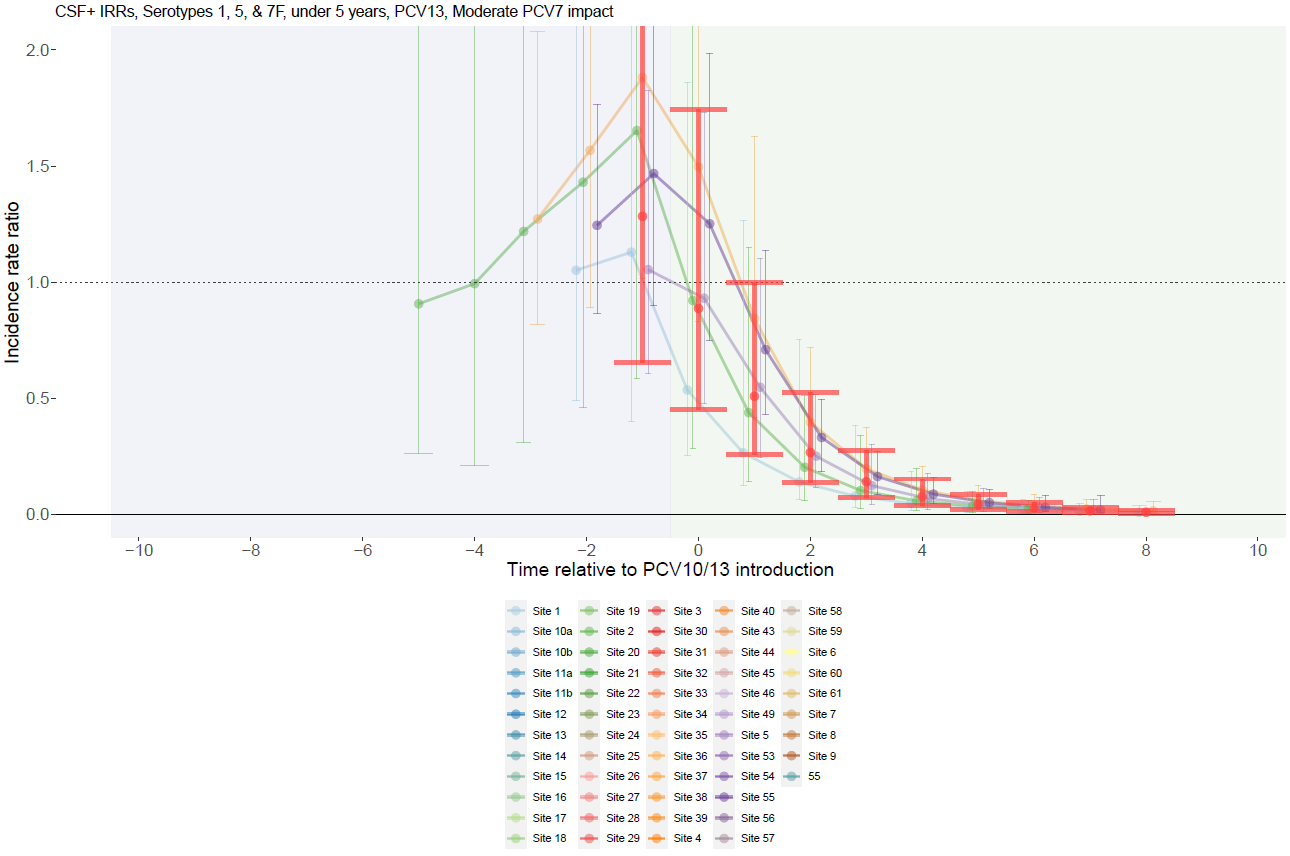


## Supplementary Figure 71. ST1, 5, 7F with PCV13 use and no PCV7 impact for children <5 years.


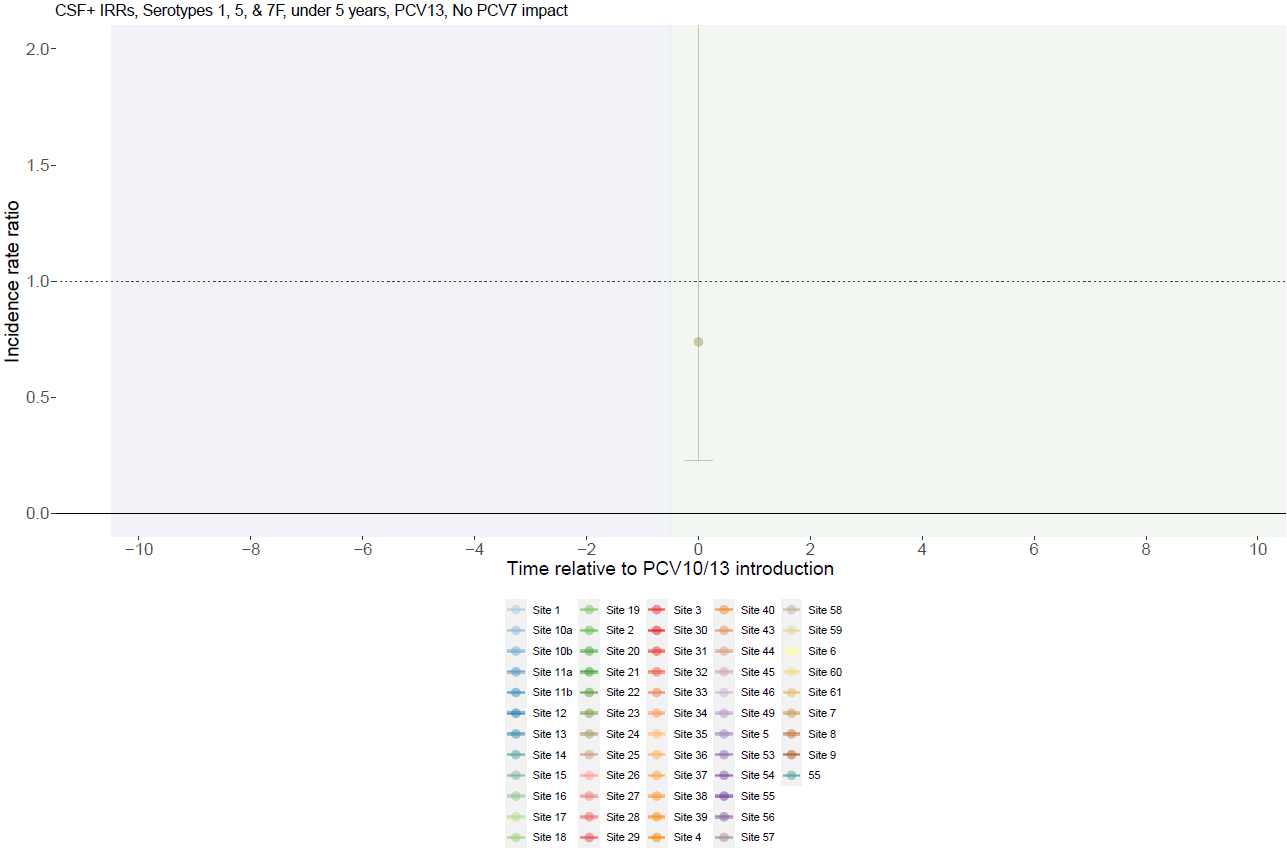


## Supplementary Figure 72. ST1, 5, 7F with PCV10 use and substantial PCV7 impact for children <5 years.


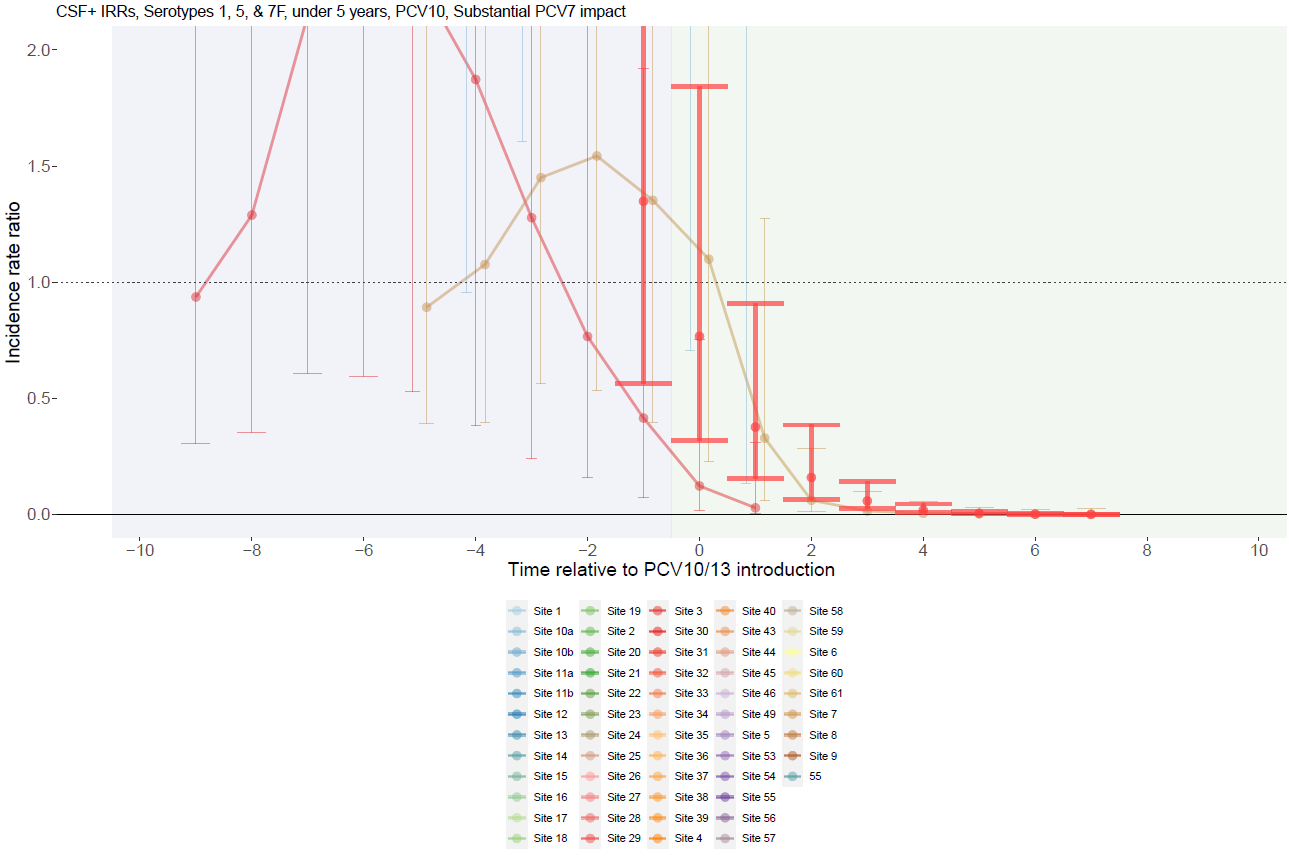


## Supplementary Figure 73. ST1, 5, 7F with PCV10 use and moderate PCV7 impact for children <5 years.


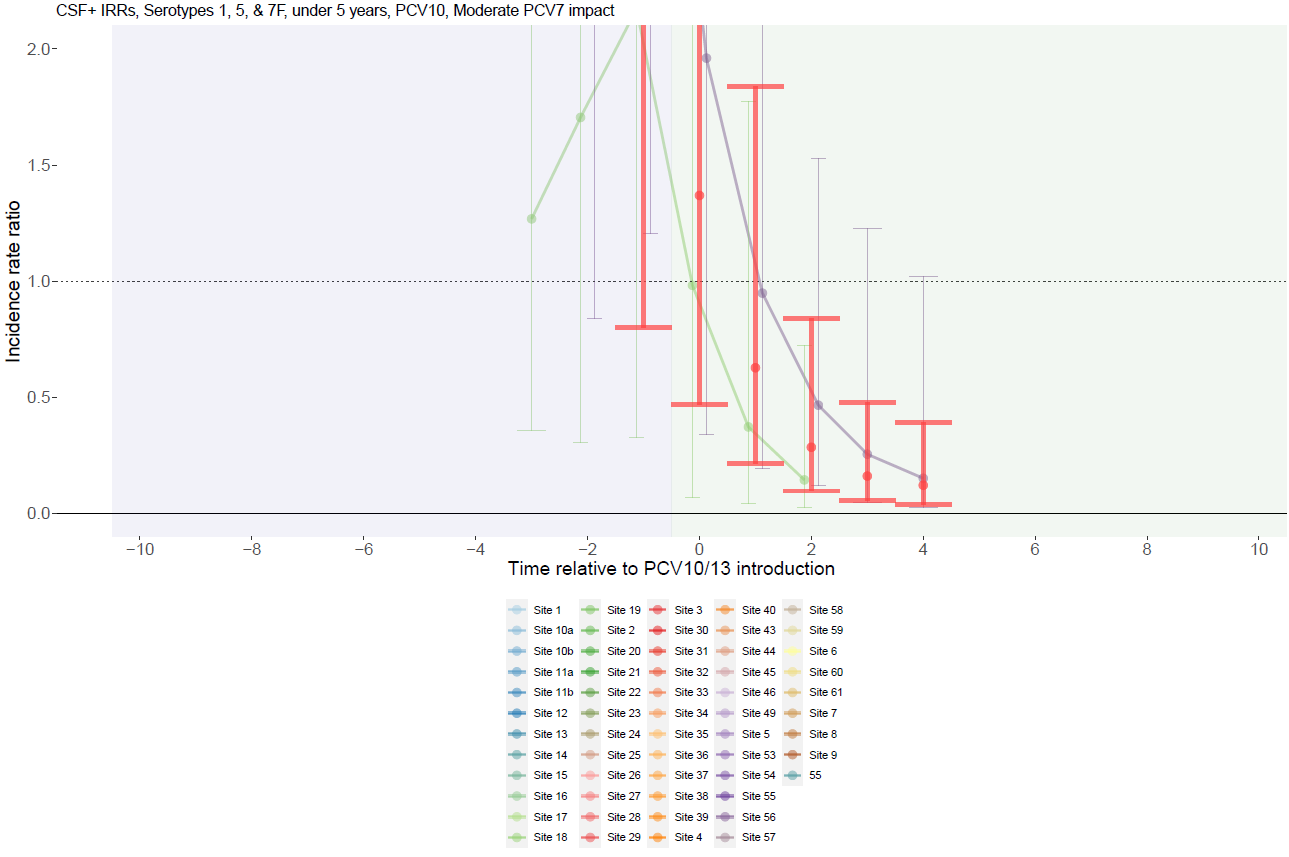


## Supplementary Figure 74. ST1, 5, 7F with PCV10 use and no PCV7 impact for children <5 years.


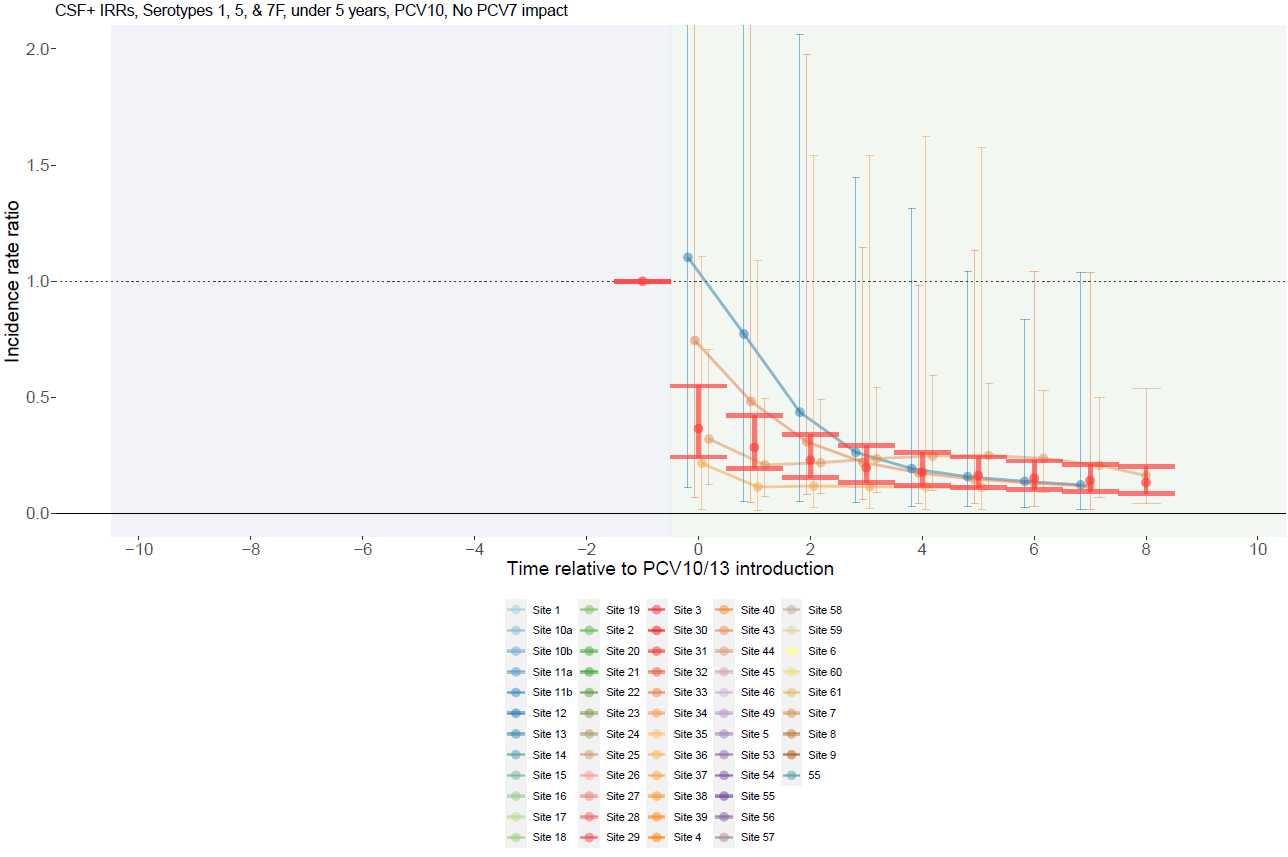


## Supplementary Figure 75. ST1, 5, 7F with PCV13 use and substantial PCV7 impact for individuals 5-17 years.


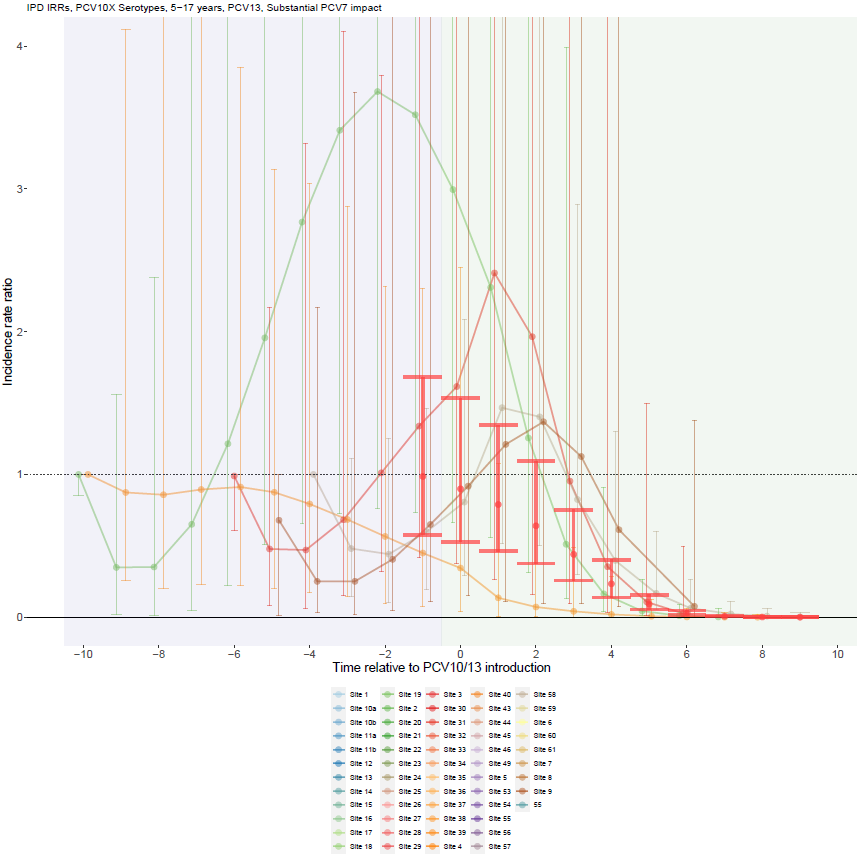


## Supplementary Figure 76. ST1, 5, 7F with PCV13 use and moderate PCV7 impact for individuals 5-17 years.


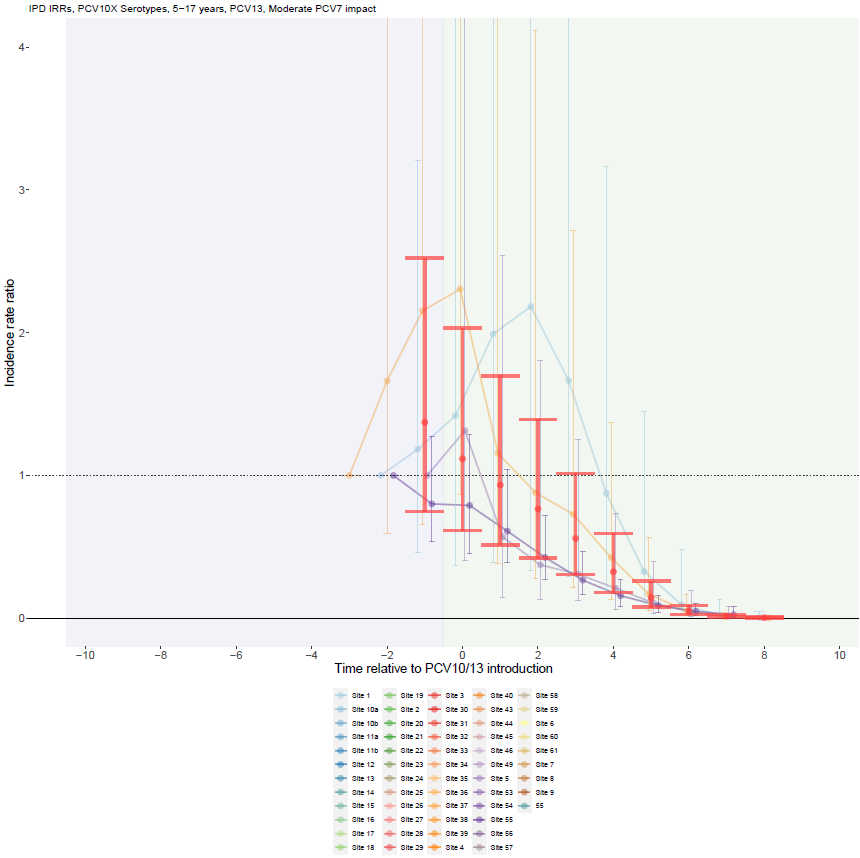


## Supplementary Figure 77. ST1, 5, 7F with PCV13 use and no PCV7 impact for individuals 5-17 years.


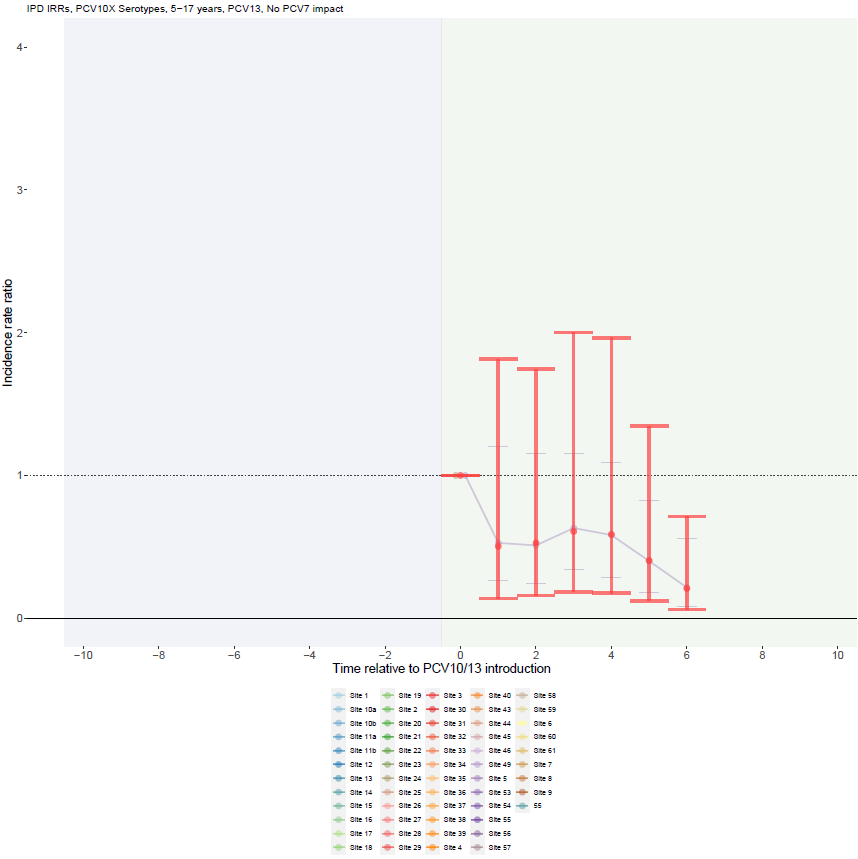


## Supplementary Figure 78. ST1, 5, 7F with PCV10 use and substantial PCV7 impact for individuals 5-17 years.


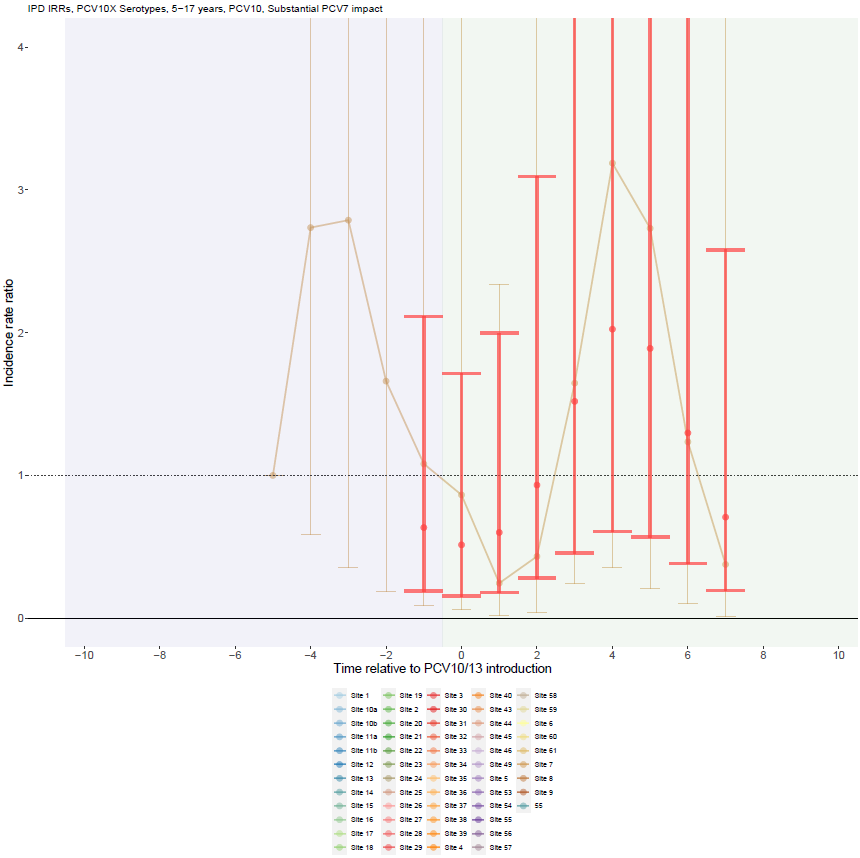


## Supplementary Figure 79. ST1, 5, 7F with PCV10 use and moderate PCV7 impact for individuals 5-17 years.


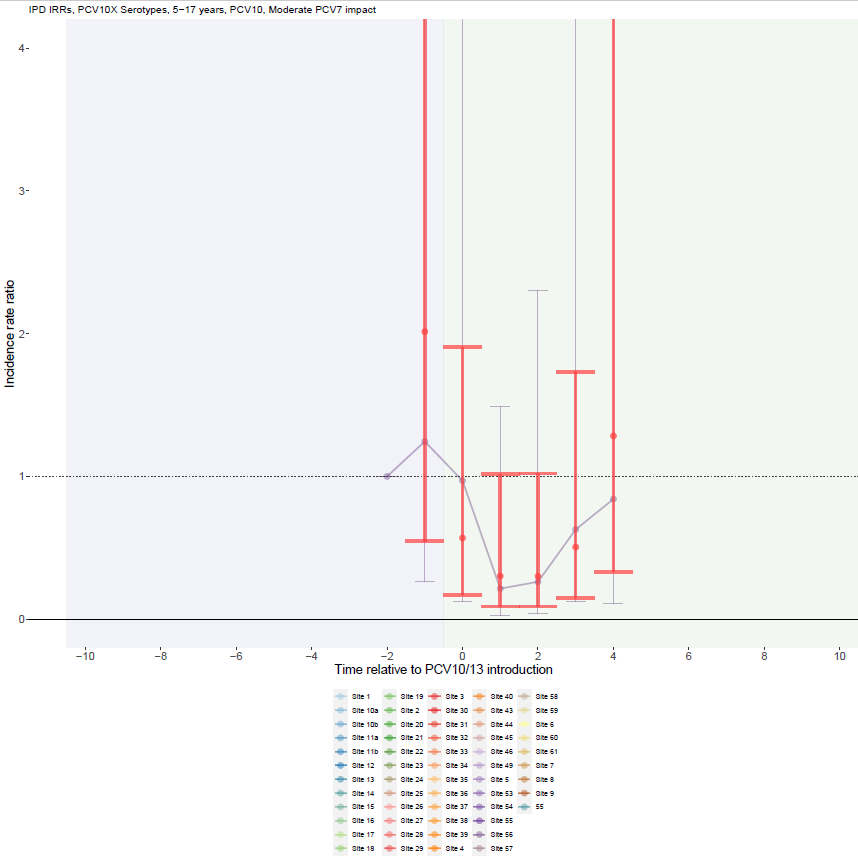


## Supplementary Figure 80. ST1, 5, 7F with PCV10 use and no PCV7 impact for individuals 5-17 years.


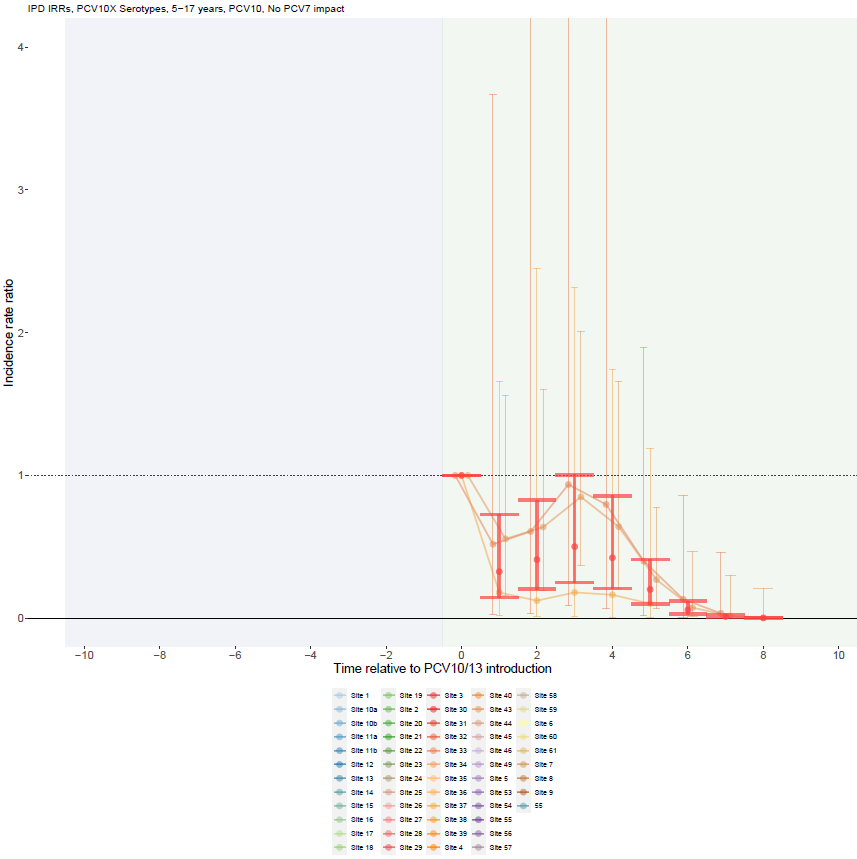


## Supplementary Figure 81. ST1, 5, 7F with PCV13 use and substantial PCV7 impact for adults >18 years.


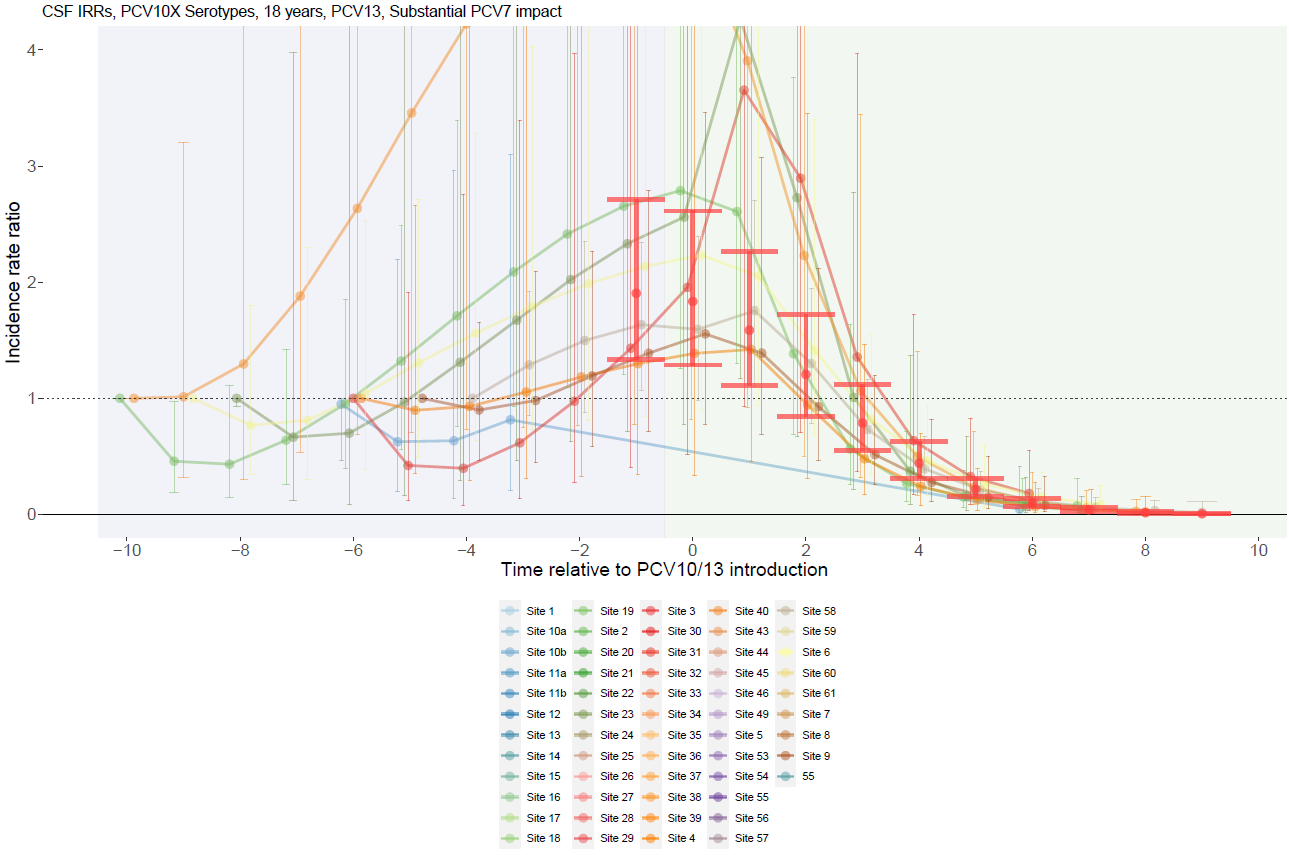


## Supplementary Figure 82. ST1, 5, 7F with PCV13 use and moderate PCV7 impact for adults >18 years.


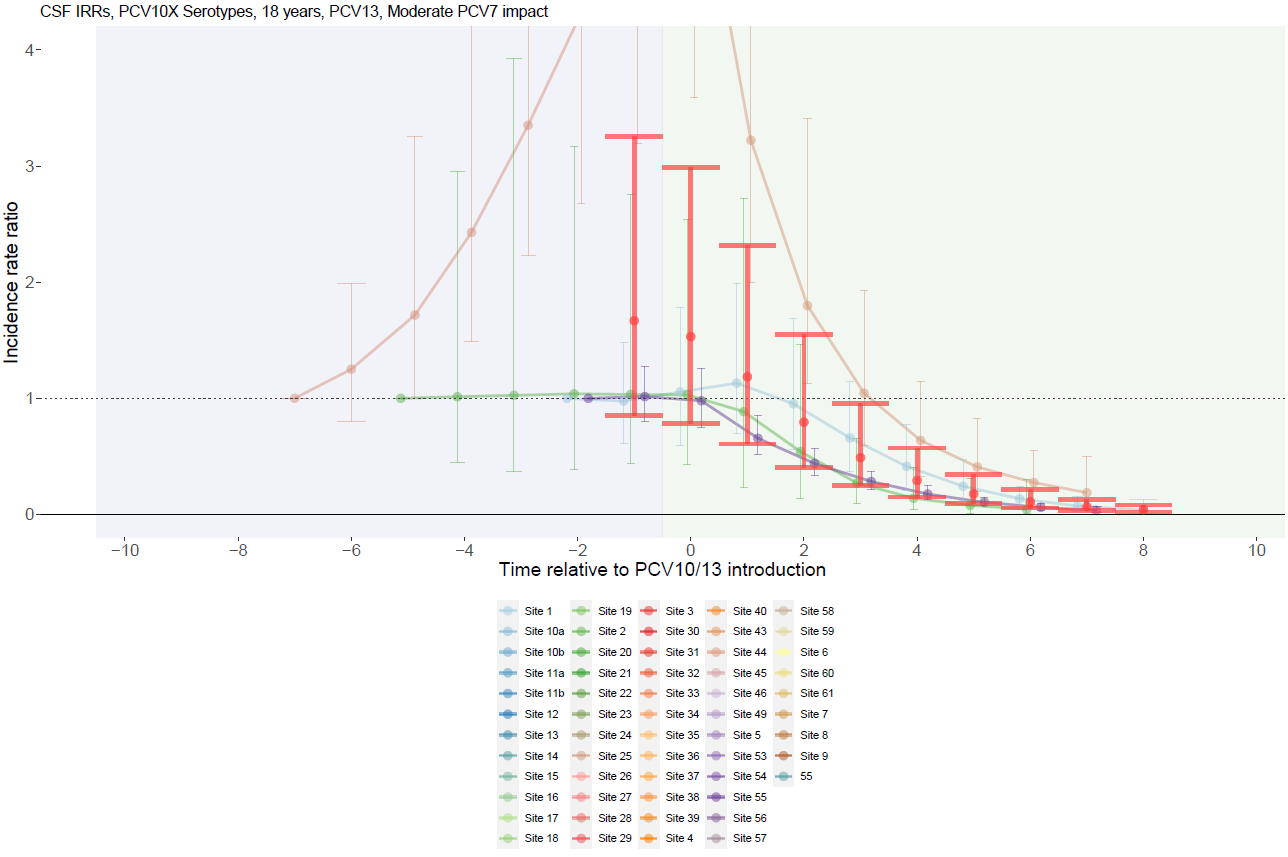


## Supplementary Figure 83. ST1, 5, 7F with PCV10 use and substantial PCV7 impact for adults >18 years.


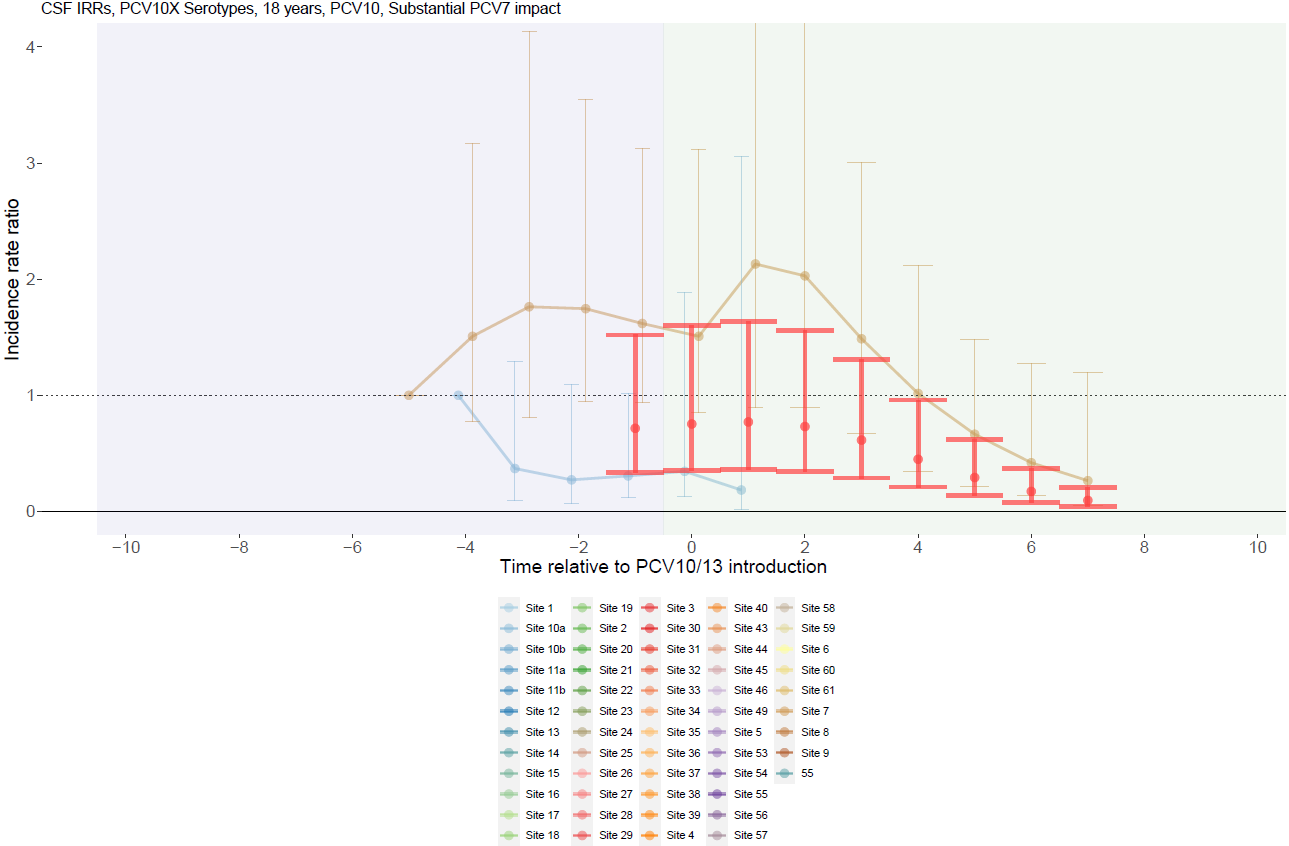


## Supplementary Figure 84. ST1, 5, 7F with PCV10 use and no PCV7 impact for adults >18 years.


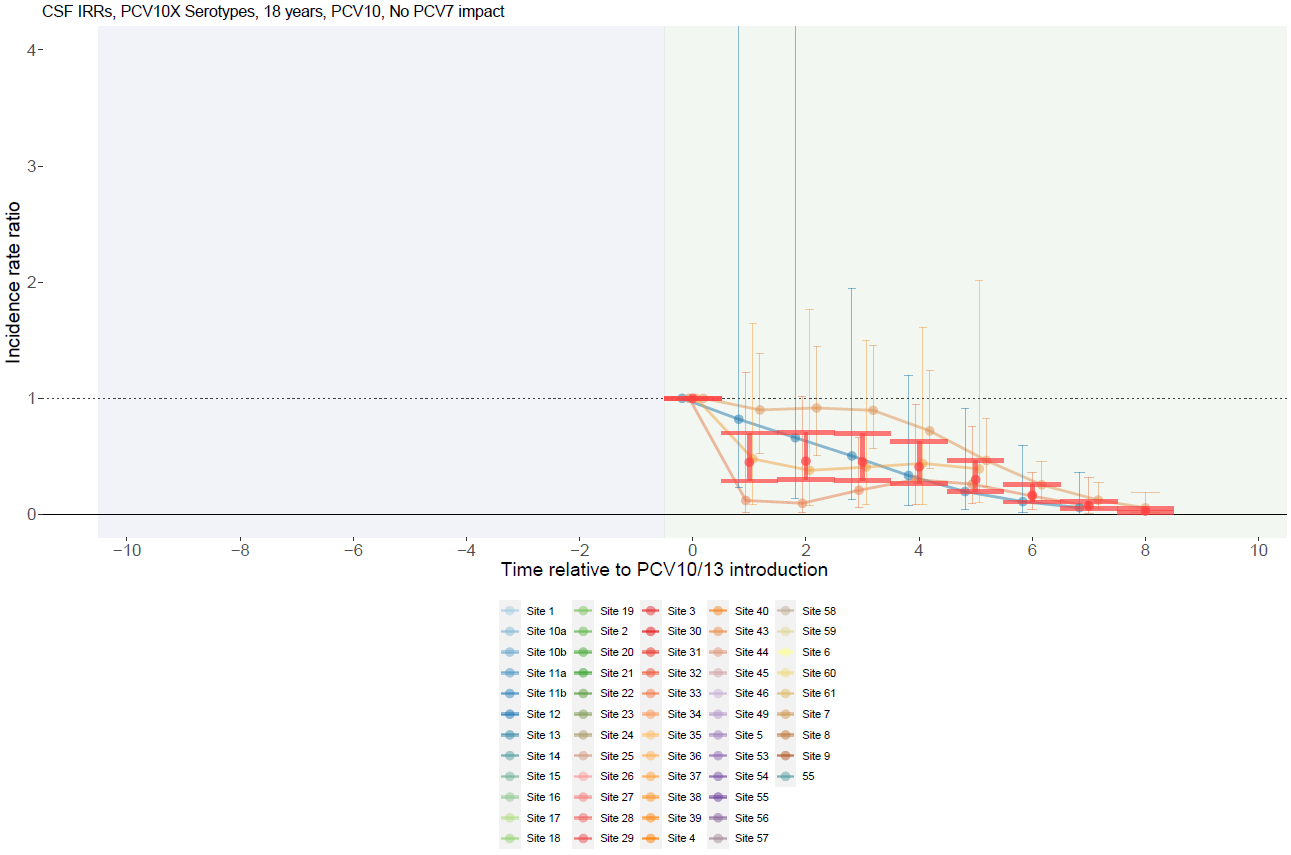


## Supplementary Figure 85. Serotype 6A with PCV13 use and substantial PCV7 impact for children <5 years.


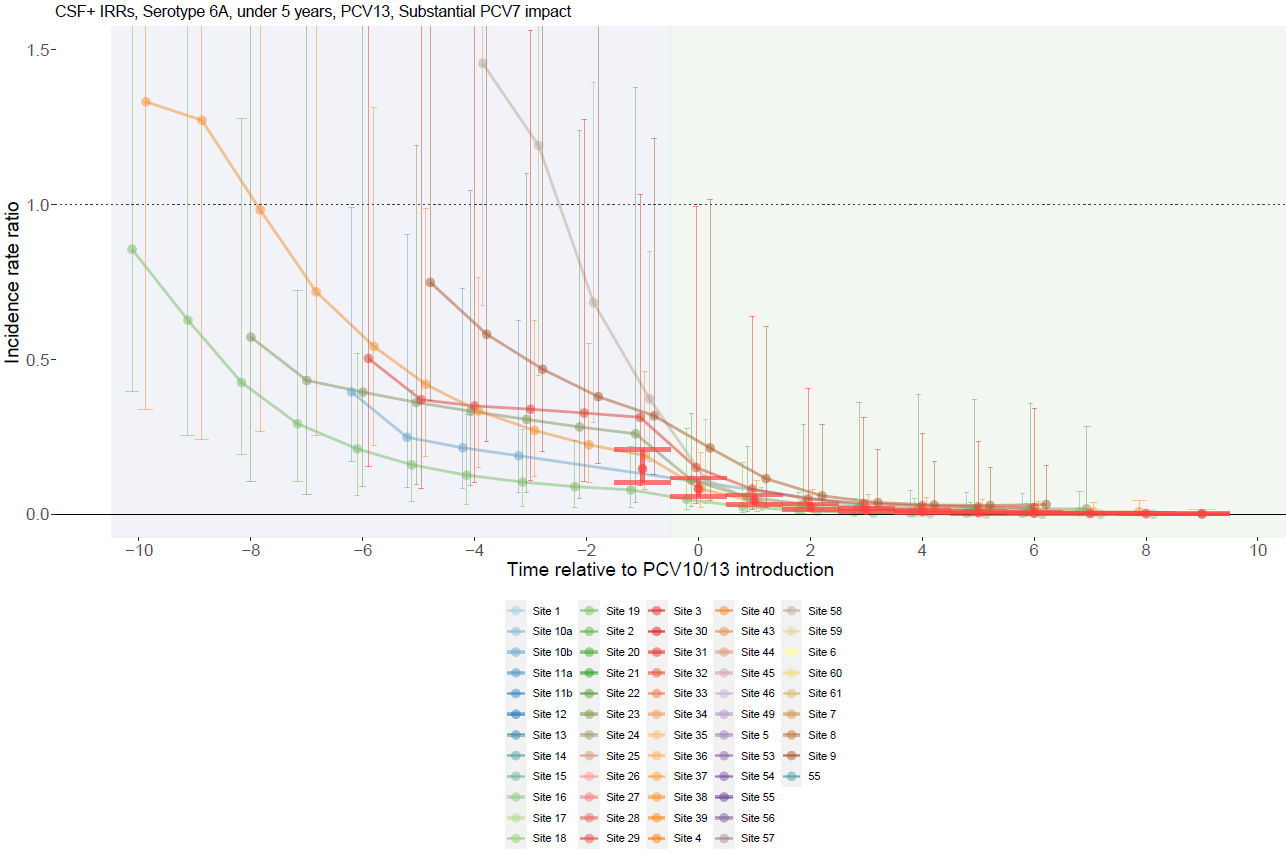


## Supplementary Figure 86. Serotype 6A with PCV13 use and moderate PCV7 impact for children <5 years.


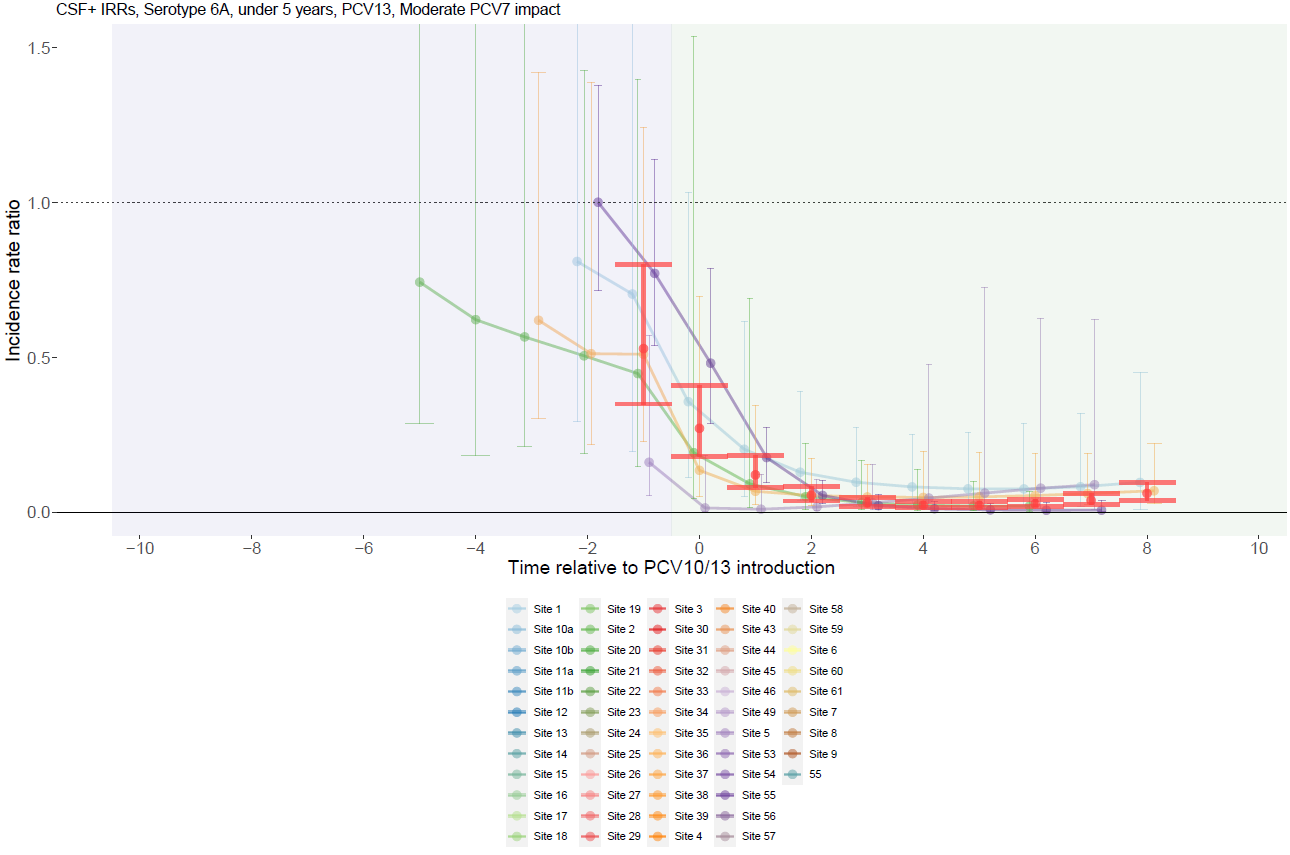


## Supplementary Figure 87. Serotype 6A with PCV13 use and no PCV7 impact for children <5 years.


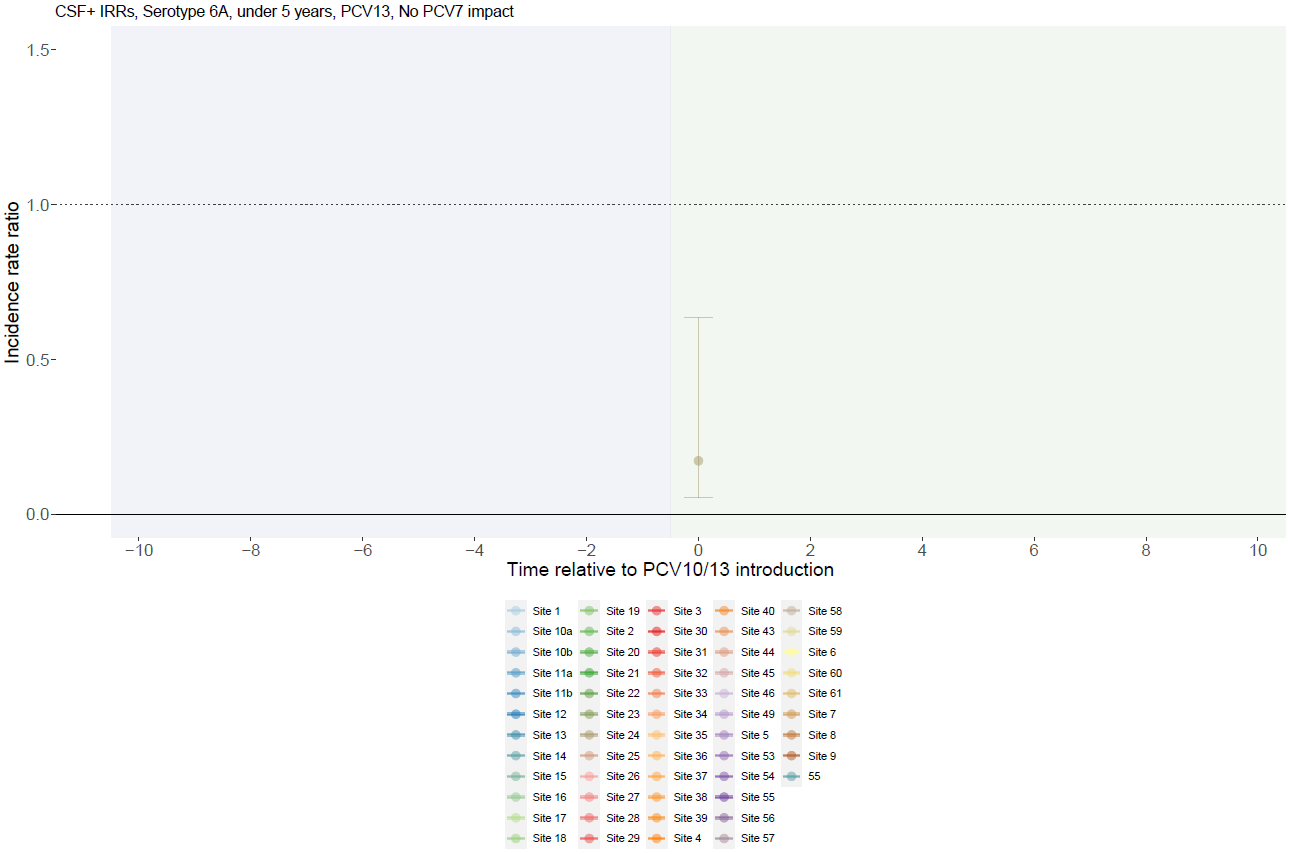


## Supplementary Figure 88. Serotype 6A with PCV10 use and substantial PCV7 impact for children <5 years.


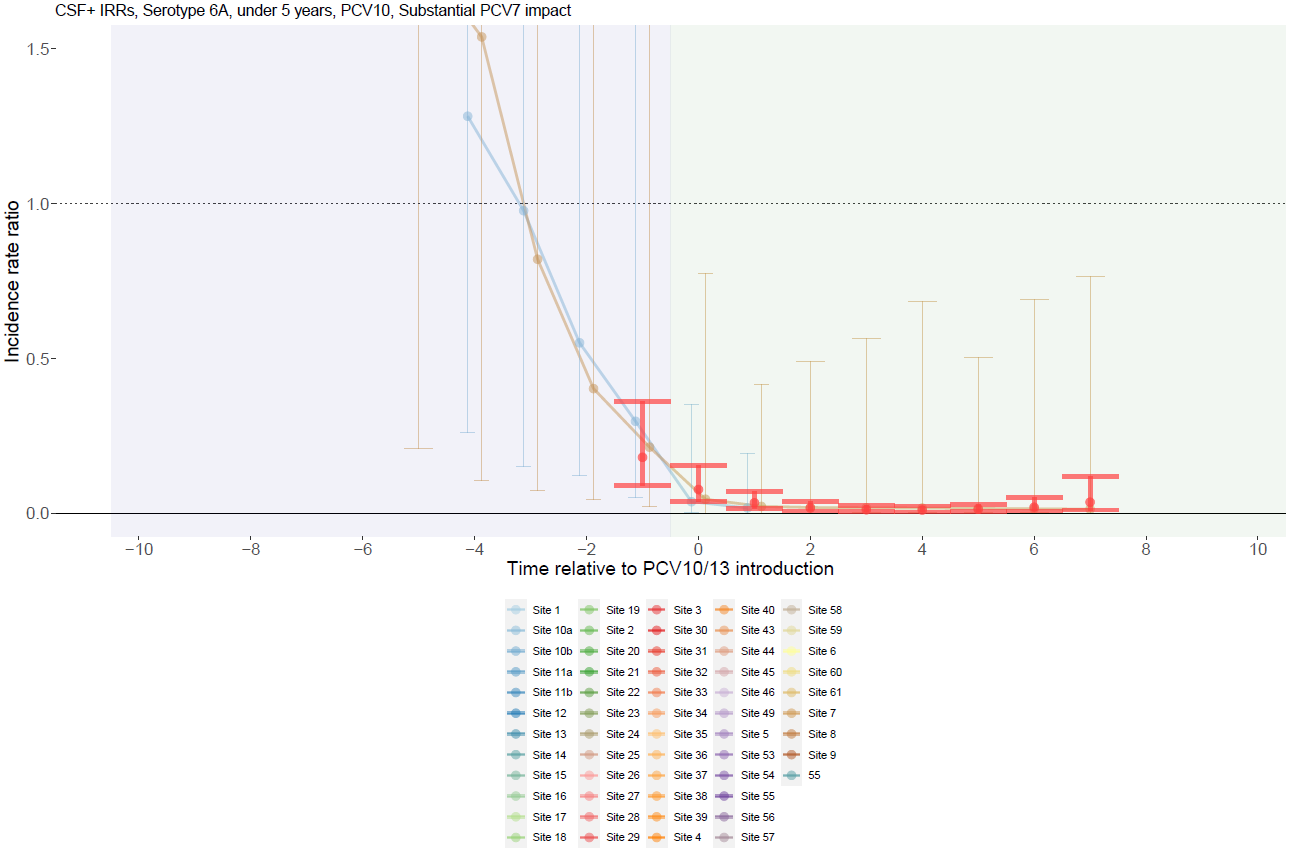


## Supplementary Figure 89. Serotype 6A with PCV10 use and moderate PCV7 impact for children <5 years.


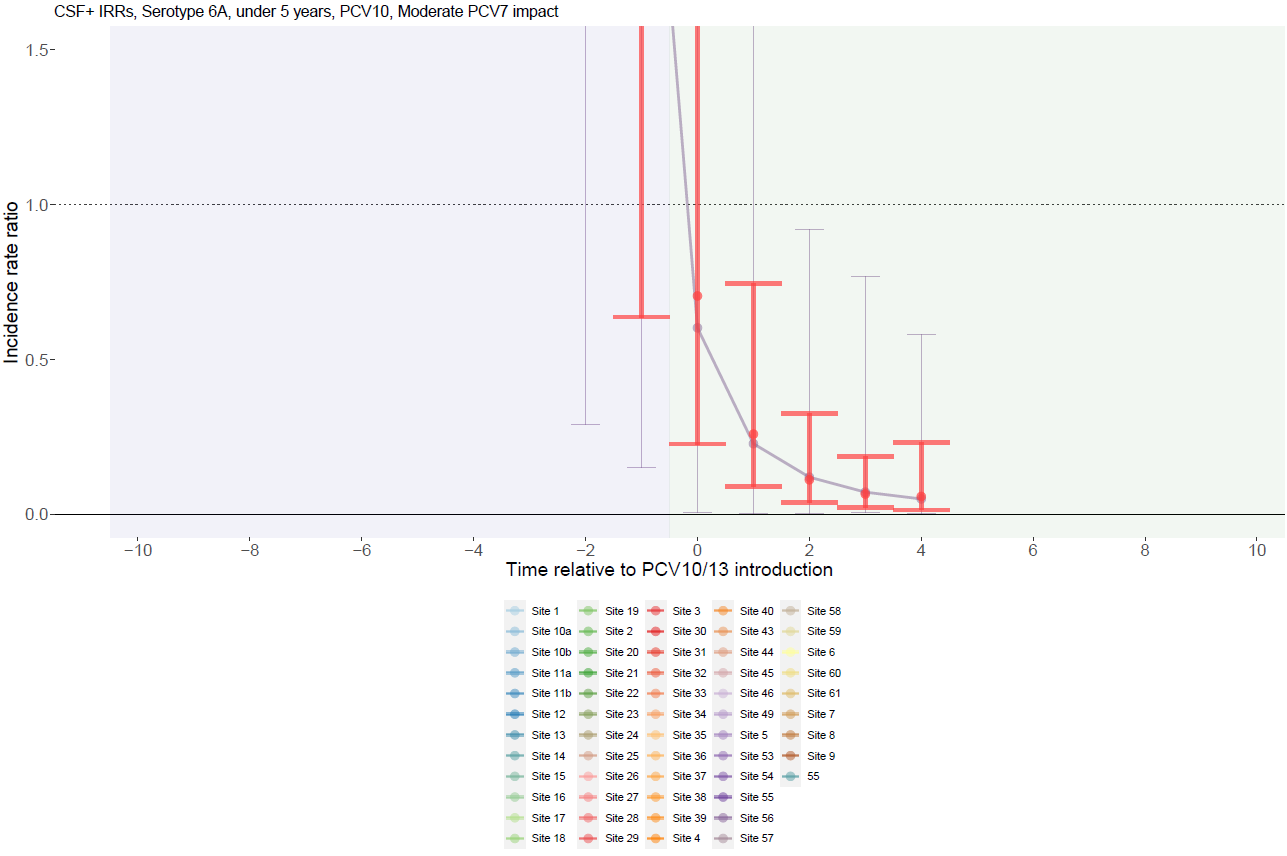


## Supplementary Figure 90. Serotype 6A with PCV10 use and no PCV7 impact for children <5 years.


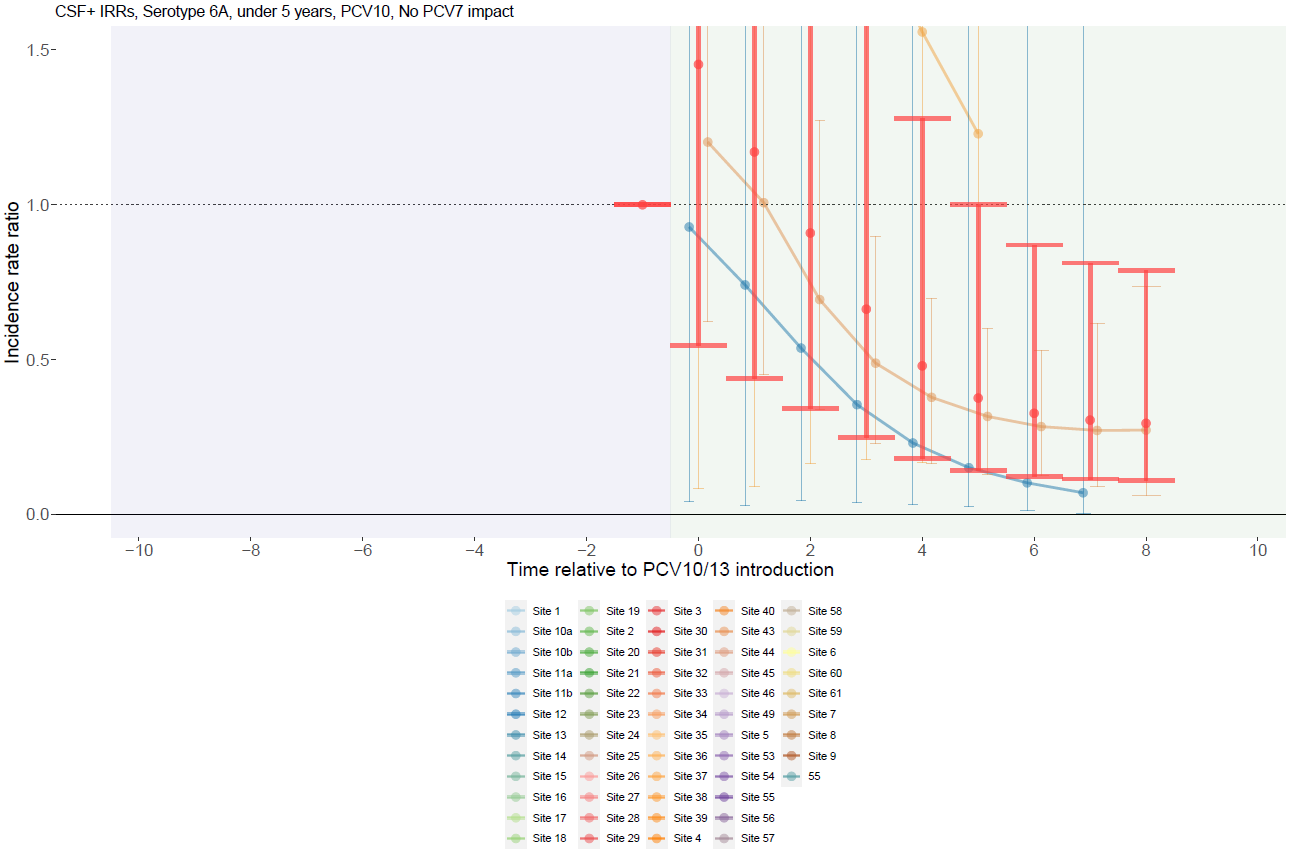


## Supplementary Figure 91. Serotype 6A with PCV13 use and substantial PCV7 impact for individuals 5-17 years.


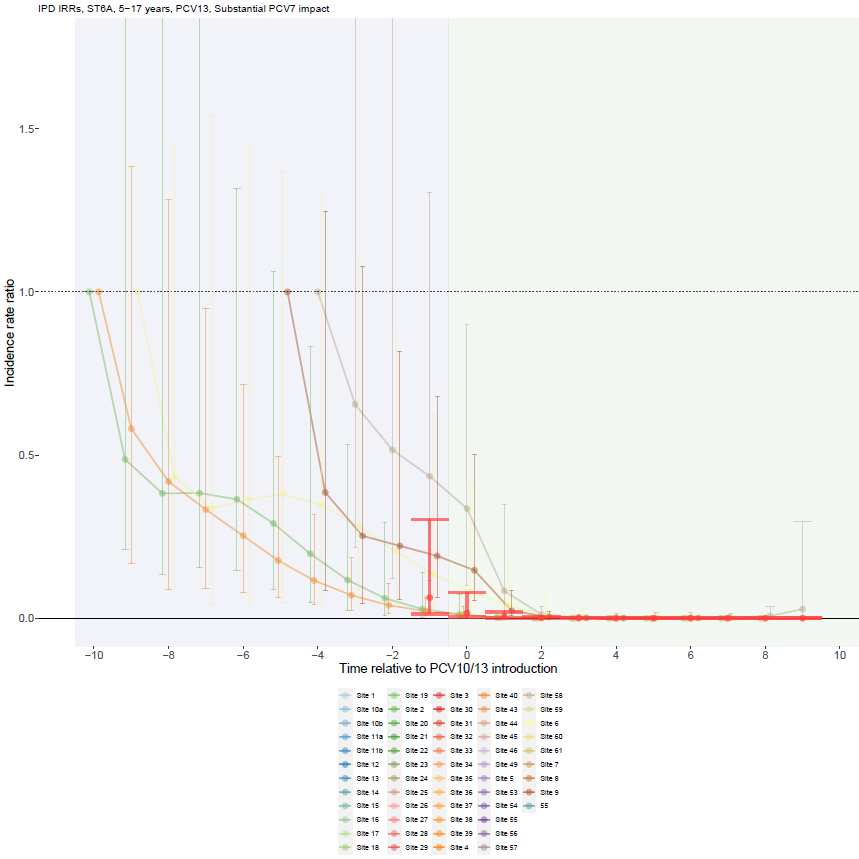


## Supplementary Figure 92. Serotype 6A with PCV13 use and moderate PCV7 impact for individuals 5-17 years.


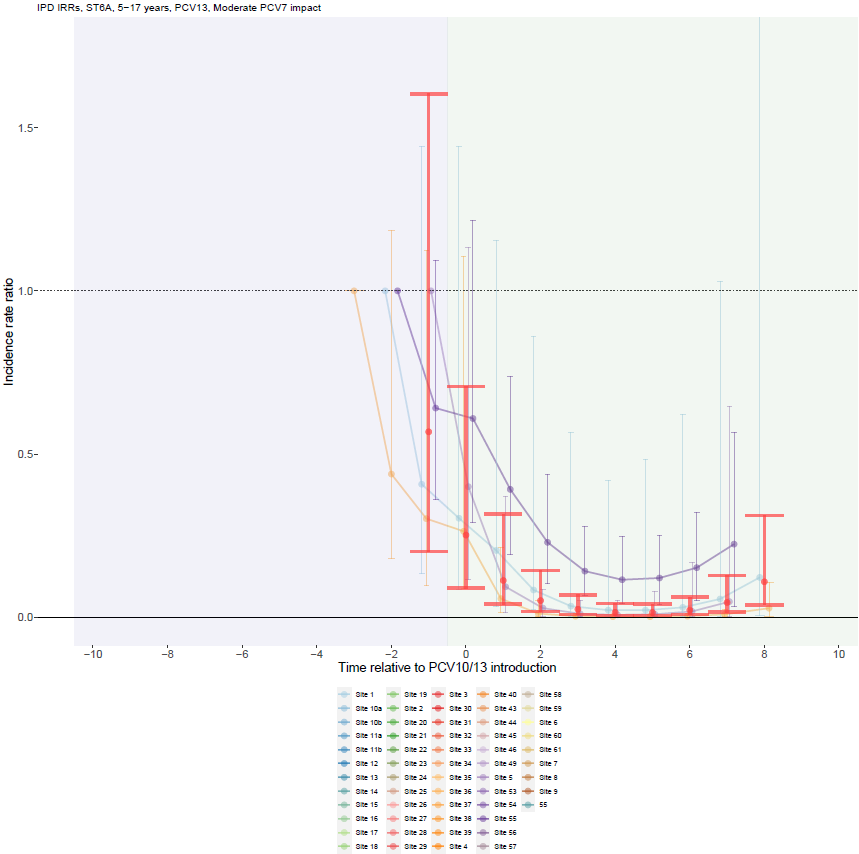


## Supplementary Figure 93. Serotype 6A with PCV13 use and no PCV7 impact for individuals 5-17 years.


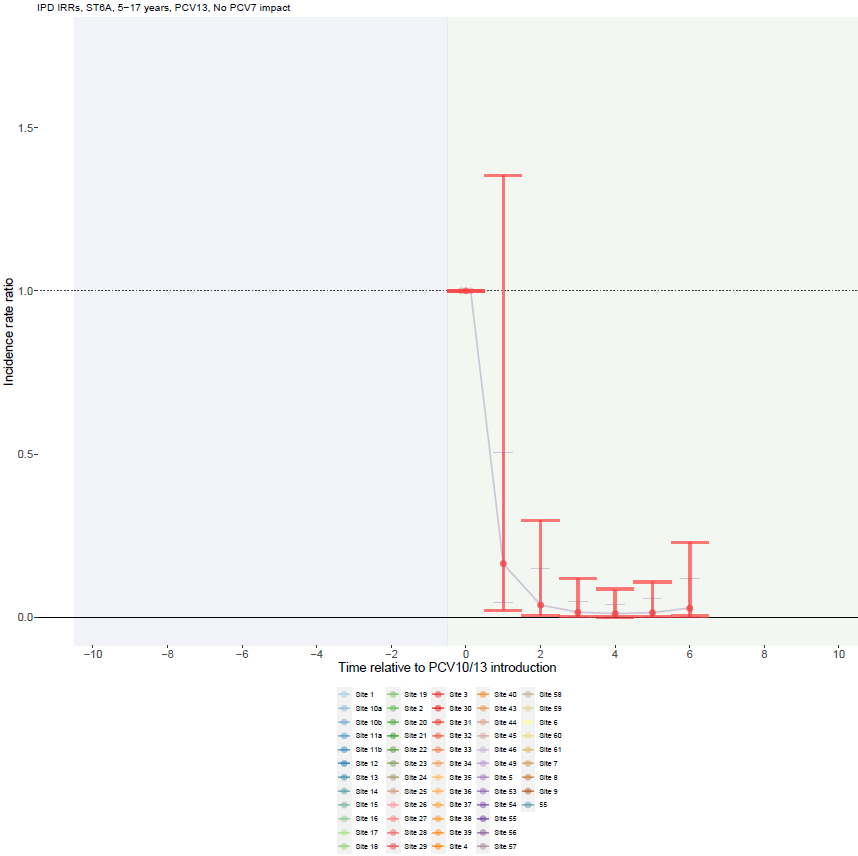


## Supplementary Figure 94. Serotype 6A with PCV10 use and moderate PCV7 impact for individuals 5-17 years.


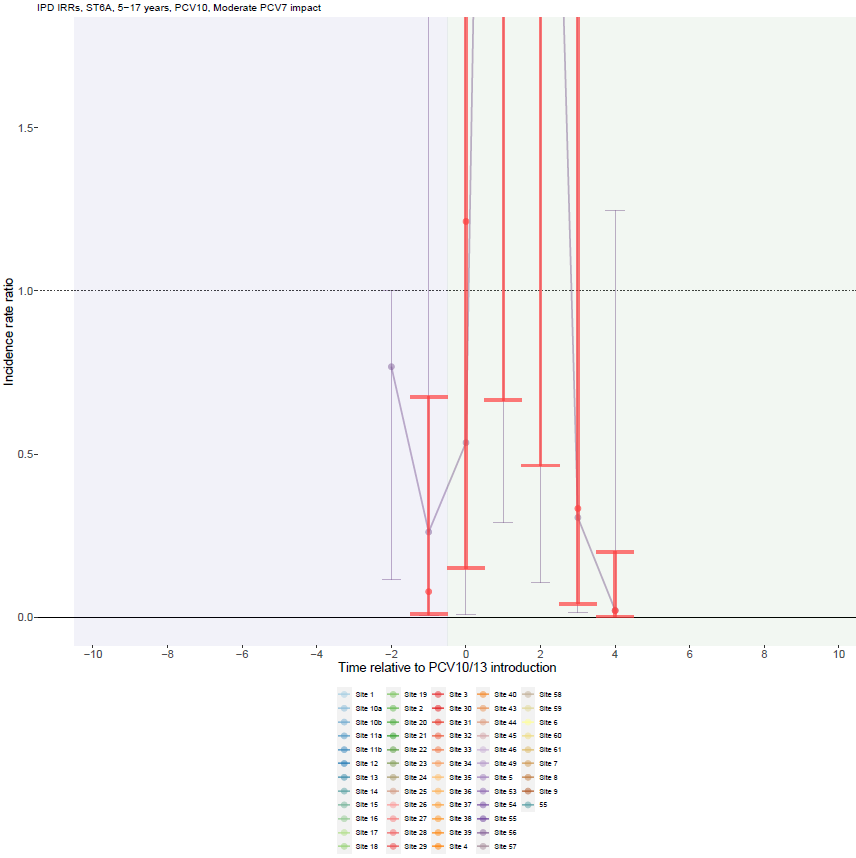


## Supplementary Figure 95. Serotype 6A with PCV10 use and no PCV7 impact for individuals 5-17 years.


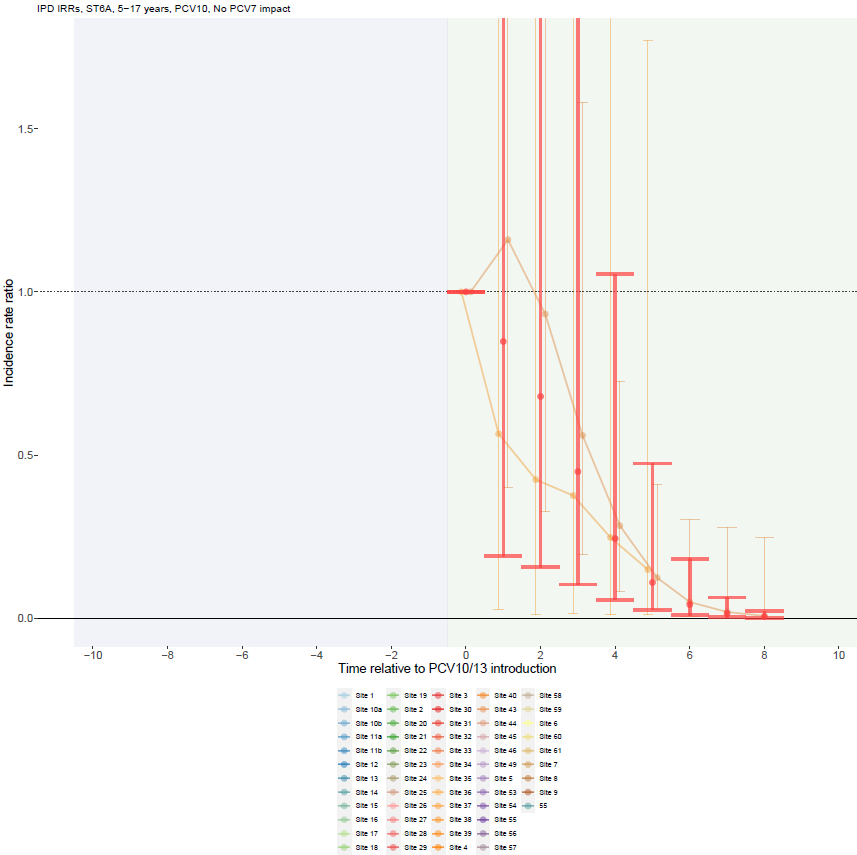


## Supplementary Figure 96. Serotype 6A with PCV13 use and substantial PCV7 impact for adults >18 years.


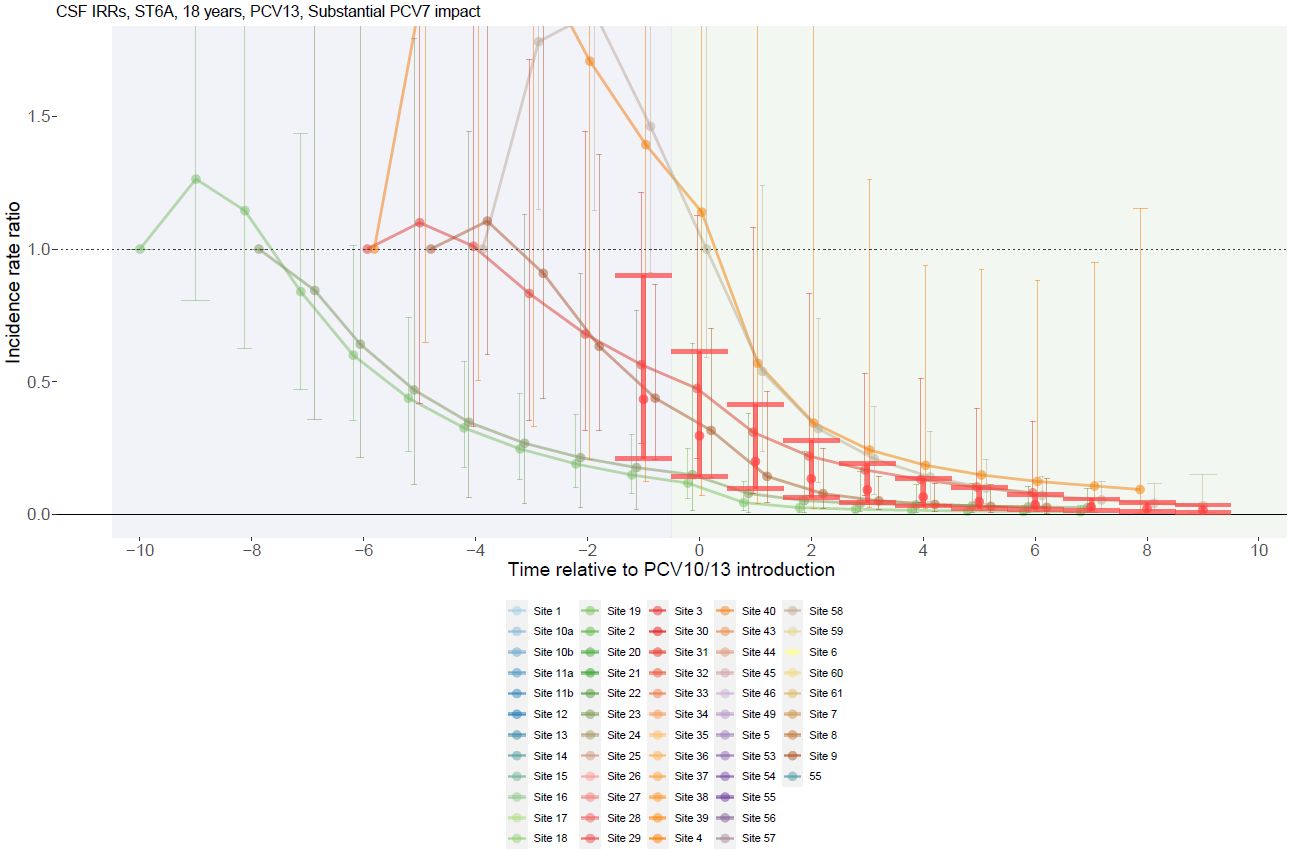


## Supplementary Figure 97. Serotype 6A with PCV13 use and moderate PCV7 impact for adults >18 years.


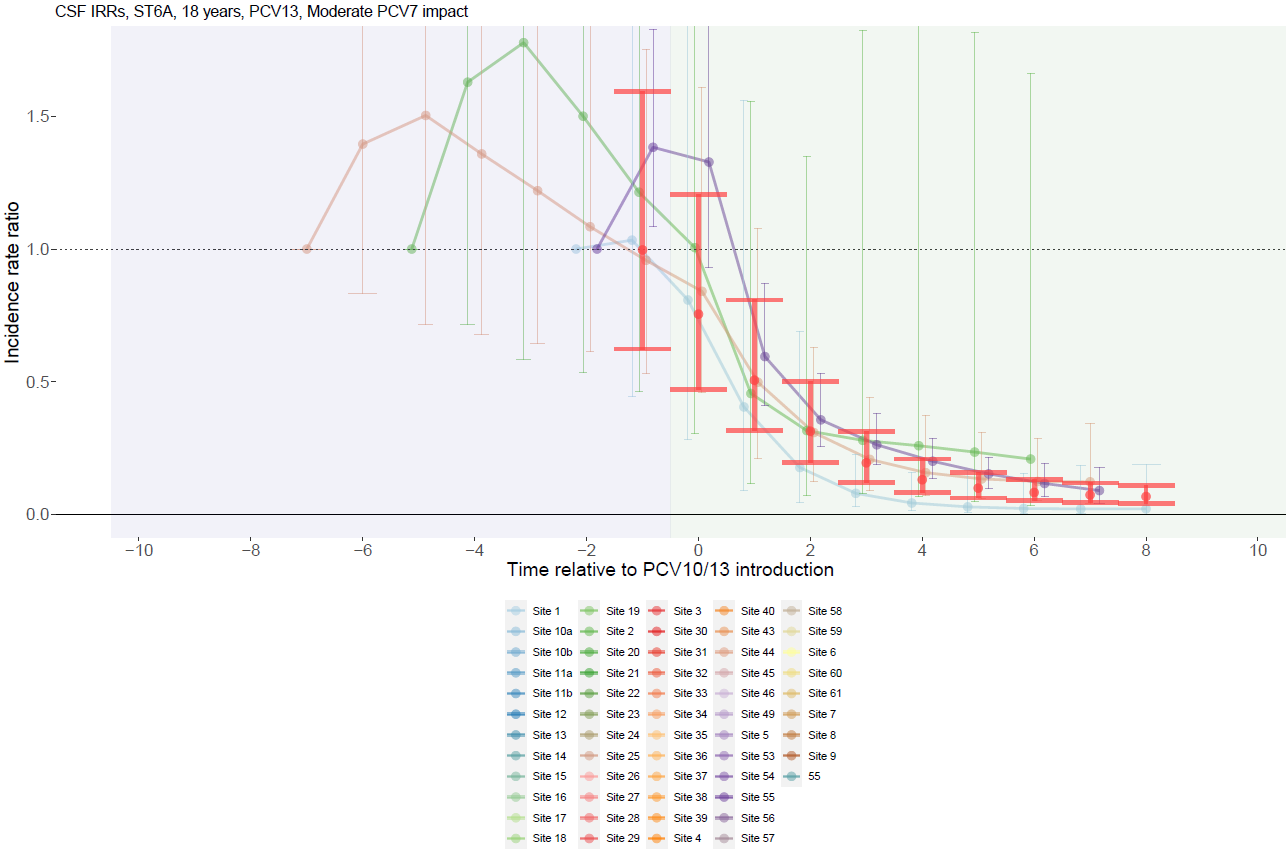


## Supplementary Figure 98. Serotype 6A with PCV10 use and substantial PCV7 impact for adults >18 years.


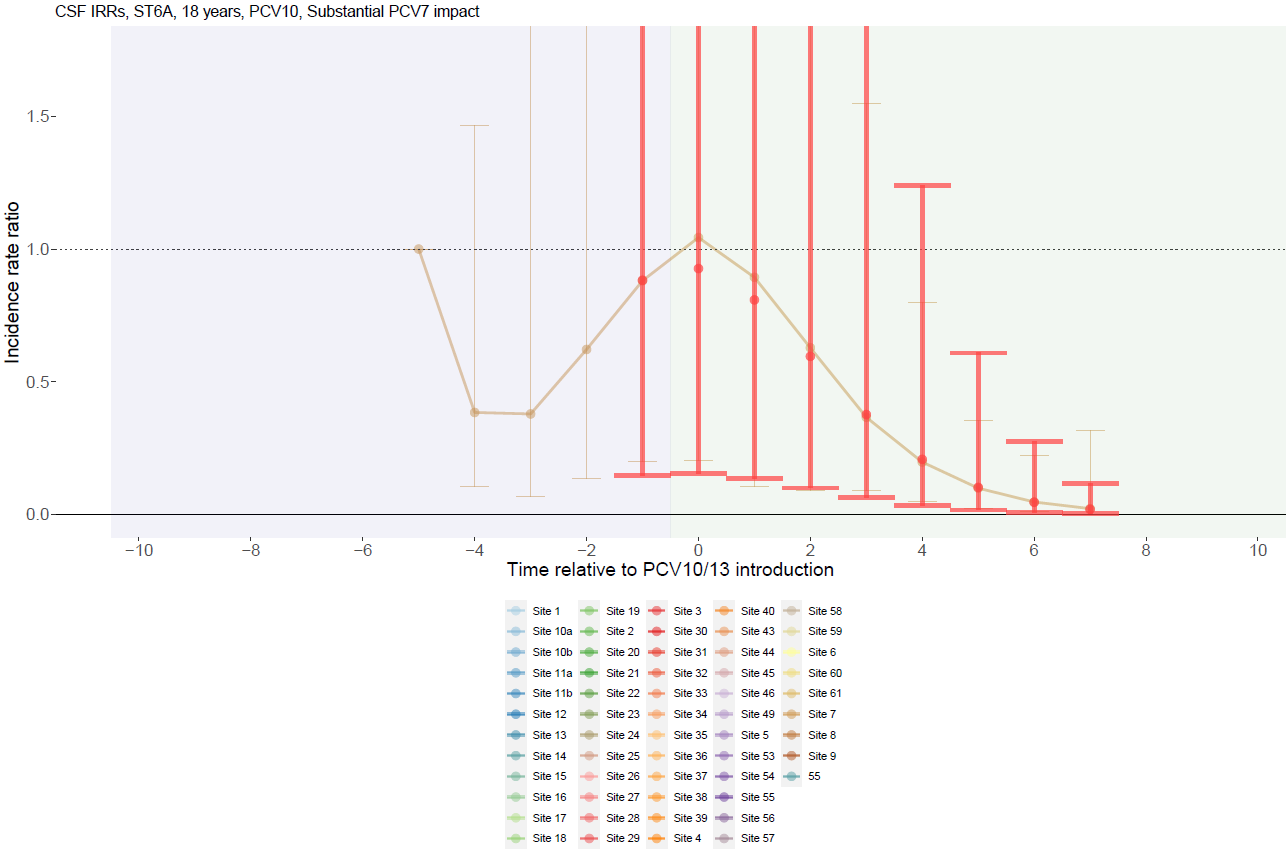


## Supplementary Figure 99. Serotype 6A with PCV10 use and no PCV7 impact for adults >18 years.

## Supplementary Figure 100. Serotype 19A with PCV13 use and substantial PCV7 impact for children <5 years.

## Supplementary Figure 101. Serotype 19A with PCV13 use and moderate PCV7 impact for children <5 years.

## Supplementary Figure 102. Serotype 19A with PCV13 use and no PCV7 impact for children <5 years.

## Supplementary Figure 103. Serotype 19A with PCV10 use and substantial PCV7 impact for children <5 years.

## Supplementary Figure 104. Serotype 19A with PCV10 use and moderate PCV7 impact for children <5 years.

## Supplementary Figure 105. Serotype 19A with PCV10 use and no PCV7 impact for children <5 years.

## Supplementary Figure 106. Serotype 19A with PCV13 use and substantial PCV7 impact for individuals 5-17 years.

## Supplementary Figure 107. Serotype 19A with PCV13 use and moderate PCV7 impact for individuals 5-17 years.

## Supplementary Figure 108. Serotype 19A with PCV13 use and no PCV7 impact for individuals 5-17 years.

## Supplementary Figure 109. Serotype 19A with PCV10 use and substantial PCV7 impact for individuals 5-17 years.

## Supplementary Figure 110. Serotype 19A with PCV10 use and moderate PCV7 impact for individuals 5-17 years.

## Supplementary Figure 111. Serotype 19A with PCV10 use and no PCV7 impact for individuals 5-17 years.

## Supplementary Figure 112. Serotype 19A with PCV13 use and substantial PCV7 impact for adults >18 years.

## Supplementary Figure 113. Serotype 19A with PCV13 use and moderate PCV7 impact for adults >18 years.

## Supplementary Figure 114. Serotype 19A with PCV10 use and substantial PCV7 impact for adults >18 years.

## Supplementary Figure 115. Serotype 19A with PCV10 use and no PCV7 impact for adults >18 years.

## Supplementary Figure 116. Serotype 3 with PCV13 use and substantial PCV7 impact for children <5 years.

## Supplementary Figure 117. Serotype 3 with PCV13 use and moderate PCV7 impact for children <5 years.

## Supplementary Figure 118. Serotype 3 with PCV13 use and no PCV7 impact for children <5 years.

## Supplementary Figure 119. Serotype 3 with PCV10 use and substantial PCV7 impact for children <5 years.

## Supplementary Figure 120. Serotype 3 with PCV10 use and moderate PCV7 impact for children <5 years.

## Supplementary Figure 121. Serotype 3 with PCV10 use and no PCV7 impact for children <5 years.

## Supplementary Figure 122. Serotype 3 with PCV13 use and substantial PCV7 impact for individuals 5-17 years.

## Supplementary Figure 123. Serotype 3 with PCV13 use and moderate PCV7 impact for individuals 5-17 years.

## Supplementary Figure 124. Serotype 3 with PCV13 use and no PCV7 impact for individuals 5-17 years.

## Supplementary Figure 125. Serotype 3 with PCV10 use and substantial PCV7 impact for individuals 5-17 years.

## Supplementary Figure 126. Serotype 3 with PCV10 use and moderate PCV7 impact for individuals 5-17 years.

## Supplementary Figure 127. Serotype 3 with PCV10 use and no PCV7 impact for individuals 5-17 years.

## Supplementary Figure 128. Serotype 3 with PCV13 use and substantial PCV7 impact for adults >18 years.

## Supplementary Figure 129. Serotype 3 with PCV13 use and moderate PCV7 impact for adults >18 years.

## Supplementary Figure 130. Serotype 3 with PCV10 use and substantial PCV7 impact for adults >18 years.

## Supplementary Figure 131. Serotype 3 with PCV10 use and no PCV7 impact for adults >18 years.

## Supplementary Figure 132. Non-PCV13 serotype with PCV13 use and substantial PCV7 impact for children <5 years.

## Supplementary Figure 133. Non-PCV13 serotype with PCV13 use and moderate PCV7 impact for children <5 years.

## Supplementary Figure 134. Non-PCV13 serotype with PCV13 use and no PCV7 impact for children <5 years.

## Supplementary Figure 135. Non-PCV13 serotype with PCV10 use and substantial PCV7 impact for children <5 years.

## Supplementary Figure 136. Non-PCV13 serotype with PCV10 use and moderate PCV7 impact for children <5 years.

## Supplementary Figure 137. Non-PCV13 serotype with PCV10 use and no PCV7 impact for children <5 years.

## Supplementary Figure 138. Non-PCV13 serotype with PCV13 use and substantial PCV7 impact for individuals 5-17 years.

## Supplementary Figure 139. Non-PCV13 serotype with PCV13 use and moderate PCV7 impact for individuals 5-17 years.

## Supplementary Figure 140. Non-PCV13 serotype with PCV13 use and no PCV7 impact for individuals 5-17 years.

## Supplementary Figure 141. Non-PCV13 serotype with PCV10 use and substantial PCV7 impact for individuals 5-17 years.

## Supplementary Figure 142. Non-PCV13 serotype with PCV10 use and moderate PCV7 impact for individuals 5-17 years.

## Supplementary Figure 143. Non-PCV13 serotype with PCV10 use and no PCV7 impact for individuals 5-17 years.

## Supplementary Figure 144. Non-PCV13 serotype with PCV13 use and substantial PCV7 impact for adults >18 years.

## Supplementary Figure 145. Non-PCV13 serotype with PCV13 use and moderate PCV7 impact for adults >18 years.

## Supplementary Figure 146. Non-PCV13 serotype with PCV10 use and substantial PCV7 impact for adults >18 years.

## Supplementary Figure 147. Non-PCV13 serotype with PCV10 use and no PCV7 impact for adults >18 years.

## Supplementary Figure 148. PCV10-type with PCV13 use and substantial PCV7 impact for children <5 years.

## Supplementary Figure 149. PCV10-type with PCV13 use and moderate PCV7 impact for children <5 years.

## Supplementary Figure 150. PCV10-type with PCV13 use and no PCV7 impact for children <5 years.

## Supplementary Figure 151. PCV10-type with PCV10 use and substantial PCV7 impact for children <5 years.

## Supplementary Figure 152. PCV10-type with PCV10 use and moderate PCV7 impact for children <5 years.

## Supplementary Figure 153. PCV10-type with PCV10 use and no PCV7 impact for children <5 years.

## Supplementary Figure 154. PCV13-type with PCV13 use and substantial PCV7 impact for children <5 years.

## Supplementary Figure 155. PCV13-type with PCV13 use and moderate PCV7 impact for children <5 years.

## Supplementary Figure 156. PCV13-type with PCV13 use and no PCV7 impact for children <5 years.

## Supplementary Figure 157. PCV13-type with PCV10 use and substantial PCV7 impact for children <5 years.

## Supplementary Figure 158. PCV13-type with PCV10 use and moderate PCV7 impact for children <5 years.

Supplementary Figure 159. PCV13-type with PCV10 use and no PCV7 impact for children <5 years.
